# Identification of miR-30b-3p and miR-30d-5p as direct regulators of androgen receptor signaling in prostate cancer by complementary functional microRNA library screening

**DOI:** 10.18632/oncotarget.12241

**Published:** 2016-09-24

**Authors:** Binod Kumar, Salar Khaleghzadegan, Brian Mears, Koji Hatano, Tarana A. Kudrolli, Wasim H. Chowdhury, David B. Yeater, Charles M. Ewing, Jun Luo, William B. Isaacs, Luigi Marchionni, Shawn E. Lupold

**Affiliations:** ^1^ The James Buchanan Brady Urologic Institute and Department of Urology, Johns Hopkins School of Medicine, Baltimore, MD, USA; ^2^ The department of Oncology, Sidney Kimmel Comprehensive Cancer Center, School of Medicine, Johns Hopkins University, Baltimore, MD, USA; ^3^ Current Address: University of Texas at San Antonio, San Antonio, Texas, USA

**Keywords:** prostate cancer, castration resistant prostate cancer, androgen receptor, microRNA, miRNA, miR-30

## Abstract

The Androgen Receptor (AR) plays a key role in prostate biology and in the progression of prostate cancer (PCa) to castration resistance. The role of microRNAs (miRNAs) in aberrant AR signaling have not been fully characterized. Here we screened a library of 810 miRNA mimics to identify miRNAs that alter AR activity in complementary functional assays including protein lysate microarray (LMA) quantification of AR and PSA protein levels, AR transcriptional reporter activity, and AR-positive PCa cell viability. Candidate AR-regulating miRNAs were verified through AR transcriptional reporter and cell viability assays. MiRNA binding sites were found within the AR 3′-untranslated region (UTR) and within the AR and AR-V7 coding regions. MiRNA activity was characterized by western blotting, 3′-UTR reporter assay, and AR-GFP and AR-V7-GFP reporter assays. Results uncovered miR-30 family members as direct AR inhibitors. Inhibition of endogenous miR-30b-3p and miR-30d-5p enhanced AR expression and androgen-independent cell growth. Droplet digital RT-PCR quantification of miR-30c-5p and miR-30d-5p revealed significantly reduced levels in metastatic castration resistant PCa (CRPC), when compared to healthy prostate tissues. MiR-30d-5p levels were inversely correlated with AR activity, as measured by PSA mRNA, in metastatic CRPC. Collectively, these studies provide a comprehensive evaluation of AR-regulating miRNAs in PCa.

## INTRODUCTION

The Androgen Receptor (AR) is a ligand-activated nuclear receptor and transcription factor that is required for the natural development of the prostate [1]. The seminal discovery by Huggins and Hodges that PCa is dependent upon androgens led to the use of androgen deprivation therapy (ADT) as a standard treatment for locally advanced, metastatic, and first-line castration resistant PCa (CRPC) [2-4]. Unfortunately, nearly all metastatic PCa cases develop resistance, often through the re-activation of AR signaling by AR gene mutation, amplification, over-expression, alternative splicing, post-translational modification, alteration of co-factor expression, or intratumoral androgen production [5-7]. One mechanism for aberrant AR protein expression or activity, that has not been as well characterized, is though microRNA (miRNA) regulation.

MiRNAs belong to a class of non-coding RNAs that post-transcriptionally regulate gene expression through complementary seed-pairing interactions within target mRNAs [8]. MiRNA expression is widely deregulated in human cancers, implicating their role in cancer etiology, progression and therapeutic resistance [9]. Several miRNAs have been reported to be deregulated in human PCa, and there is potential for their use as biomarkers or therapeutic targets [10]. A number of miRNAs have been found to target AR expression [11-16]; however, the full array of AR-regulating miRNAs has yet to be uncovered. Further, there is little knowledge regarding the levels of AR-regulating miRNAs in the most relevant tissue, metastatic CRPC, and correlative expression analyses between AR-targeting miRNAs and AR activity are lacking in these important tissues. In light of this we sought to identify AR-regulating miRNAs, through a series of complementary functional screens, and to study their potential role in advanced disease through gene expression analyses in localized hormone sensitive PCa and metastatic CRPC.

A library of 810 human miRNA mimics was transfected into three AR-positive PCa cell lines (LNCaP, LAPC4, and VCaP) and miRNA-induced changes in AR protein expression were interrogated by protein LMA. Replicate protein LMAs were also probed for PSA protein expression, to detect potential miRNA-regulation of AR transcriptional activity. The mimic library was further used to screen for miRNA-induced changes in AR transcriptional activity, by using an AR-responsive promoter and reporter assay [17]. Finally, a screen for miRNA-induced changes in AR-positive PCa cell growth and survival was completed, through a bioluminescent cell viability reporter assay [18]. The results identify novel AR-regulating miRNAs, validate previously identified AR-regulating miRNAs, and evaluate miRNA expression and AR-responsive transcripts in PCa specimens.

## RESULTS

### Functional screening identifies miRNAs that modulate the AR signaling axis

**Figure 1:**
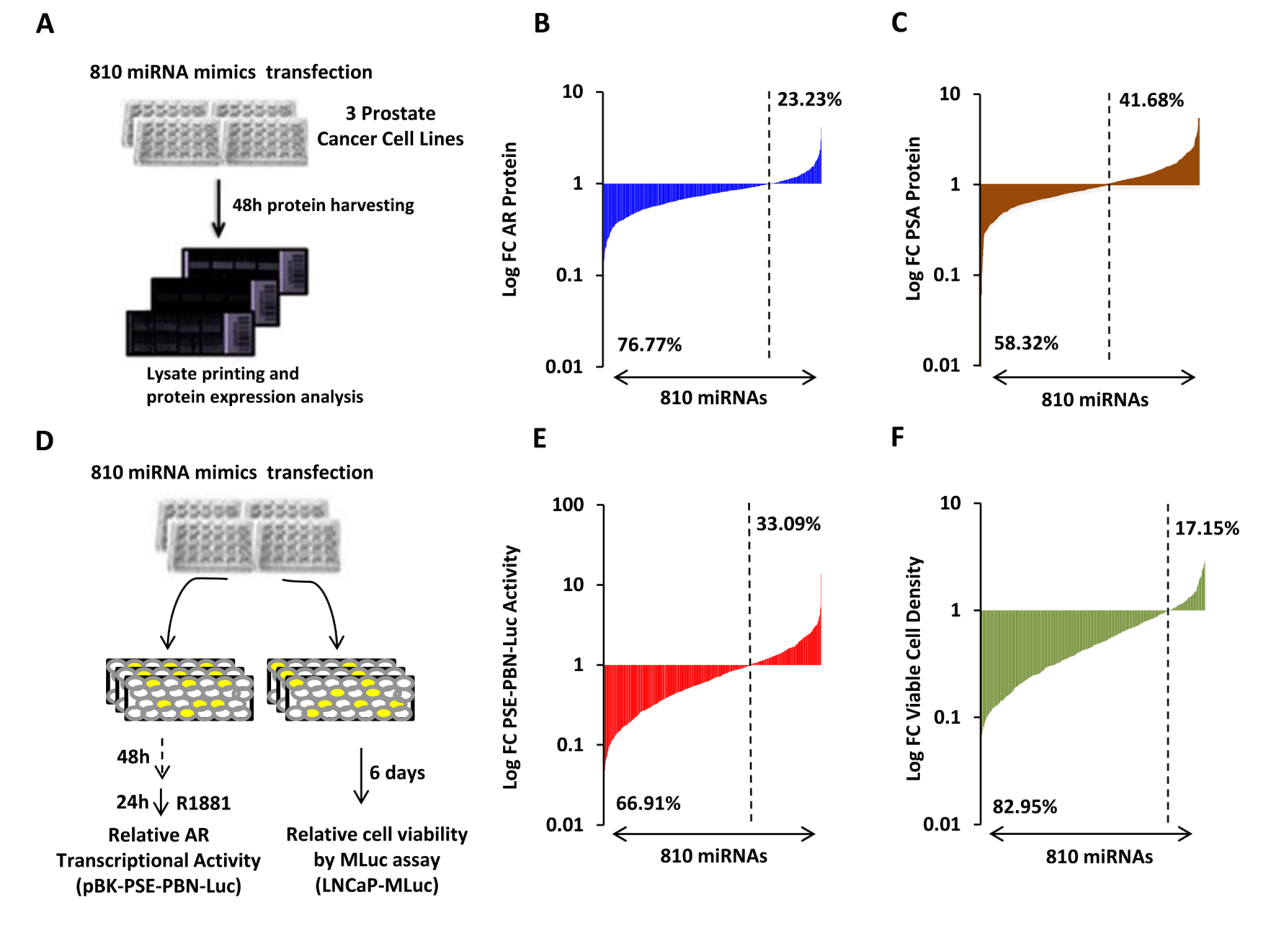
High-throughput miRNA mimic library screen for miRNAs that modulate the AR Signaling Axis A, Schematic of miRNA mimic screen for AR and PSA protein level by LMA. B, Log transformed FC in AR protein level, relative to control miRNA, in LNCaP cells 48 hours post transfection. AR, normalized to GAPDH, is organized as a waterfall plot for each mimic, ranked by FC. C, Log transformed FC in PSA protein level, relative to control miRNA, in LNCaP cells 48 hours post transfection. D, Schematic of miRNA mimic screens for AR transcriptional activity and cell viability. AR was stimulated 48 hours post transfection by 5 nM R1881, and activity detected 24 hours later. Viable cell density was quantified by MLuc Viability Assay 6 days post transfection. E, Log transformed FC in AR transcriptional activity as measured by PSE-PBN-Luc reporter, relative to control miRNA and normalized to *Renilla* luciferase, in LNCaP cells. F, Log transformed FC in LNCaP-MLuc viable cell density as measured by MLuc Cell Viability Assay, relative to control miRNA, 6 days post transfection. Waterfall plots: Dashed line indicates negative control mimic. Percentage of miRNAs with signal above and below control miRNA are indicated.

Four complementary screens were performed to identify miRNAs capable of altering AR expression and activity. First, protein lysates were generated from three androgen-responsive PCa cell lines (LNCaP, LAPC4 and VCaP) that had been separately transfected with each of 810 different miRNA mimics (Figure 1A, Schematic). Three dilutions of lysates were spotted on replicate nitrocellulose-coated slides in triplicate. The resulting LMAs were probed for AR and GAPDH protein by near-infrared immunoassay. The normalized fold change (FC) in AR protein level for each mimic, relative to a negative control miRNA, is presented for LNCaP cells in Figure 1B. The results are organized as a waterfall plot with the leftmost signal corresponding to the most suppressive mimic. The dashed line indicates relative AR protein level in cells transfected with a control miRNA. Notably, AR protein levels were reduced, to some degree, by the majority of the mimics in the library (76%). However, only a small percentage of mimics demonstrated potent suppression or stimulation of AR protein levels. These are apparent at the extreme left and right ends of each waterfall plot. A similar pattern was observed in waterfall plots for VCaP and LAPC4 cells, where 75% and 83% of mimics reduced AR protein levels (Supplementary Figure S2A-B). PSA expression is transcriptionally regulated by AR [19]. We therefore hypothesized that many of the AR suppressive mimics may also suppress PSA protein levels. A second screen investigated this, by probing replicate LMAs for PSA protein. The log transformed FC in PSA protein level, for each mimic, is presented for LNCaP cells in Figure 1C. PSA protein levels were reduced, to some degree, by more than half of the mimics. This was also observed in VCaP and LAPC4 cells (Supplementary Figure S2C-D). A moderate, but statistically significant, correlation was observed between AR and PSA protein levels in LNCaP cells (Pearson r = 0.497, p < 0.001), VCaP cells (Pearson r = 0.301, p < 0.001), and LAPC4 cells (Pearson r = 0.536, p < 0.001) from this screen, suggesting that some of the PSA regulating mimics may act through AR suppression (Supplementary Figure S3). However, it is notable that miRNAs may also reduce PSA protein levels by directly targeting the 3′UTR of the PSA mRNA [20].

Two additional functional screens were performed using LNCaP cells and the same library of 810 miRNA mimics (Figure 1D, Schematic). First, miRNA-mediated changes in AR transcriptional activity were analyzed by a probasin (PBN) promoter and PSA enhancer (PSE) driven luciferase reporter [17]. Endogenous AR was activated 48 hours after miRNA and reporter transfection, by treatment with the synthetic androgen, R1881. Normalized luciferase activity was detected 24 hours later. Similar to AR protein, AR transcriptional activity was reduced to some degree by more than half of the mimics (67%) (Figure 1E). The fourth, and final screen, analyzed miRNA-induced changes in AR-positive PCa cell viability. Viability was analyzed 6 days after mimic transfection in LNCaP-MLuc cells, which secrete *Metridia* luciferase as a bioluminescent reporter that is representative of viable cell density [18]. An endpoint of 6 days was selected for analysis, based on previous results of androgen-induced cell growth in LNCaP cells [21]. Notably, 83% of mimics were found to be growth suppressive, to some degree (Figure 1F). The normalized data from these four screens is available in Supplementary Tables S1-S3. Collectively these results reflect a broad and general suppressive nature of miRNA mimics that is not necessarily representative of specific or endogenous activity. At the same time, a small number of miRNAs were found to be potent inhibitors of AR and AR activity in multiple assays.

### Identification of candidate AR-regulating miRNA mimics

**Figure 2:**
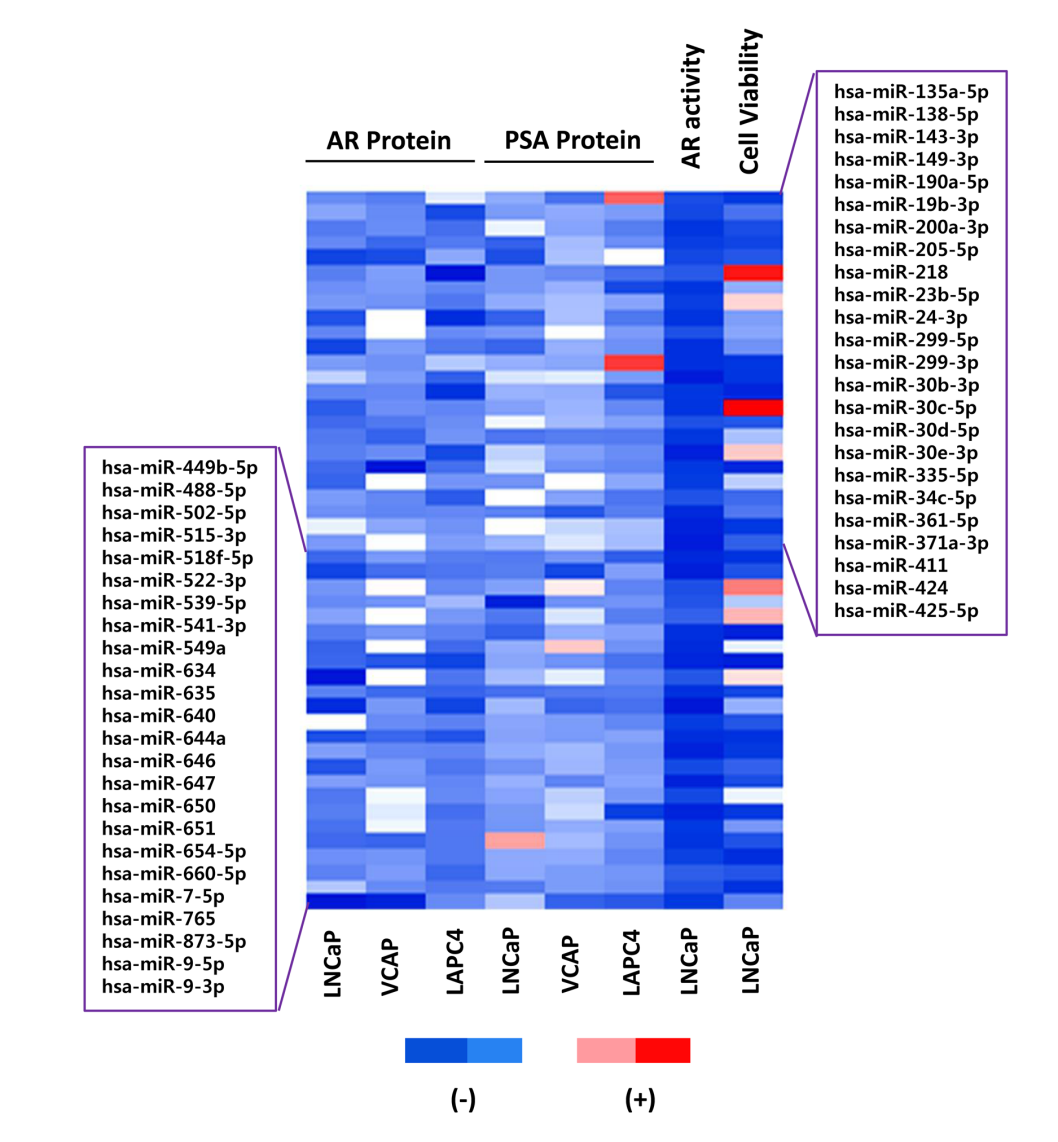
Candidate AR regulating miRNA mimics 48 candidate mimics. The top 25% of inhibitory miRNA mimics from each screen and cell line were compared to identify 43 candidates which inhibited AR transcriptional activity, as well as AR and PSA protein level, in at least two cell lines. The five most potent inhibitors of AR transcription activity were also included. For visualization, a heat map of linear FC in signal for each mimic, assay, and cell line is presented with red indicating an increase in signal and blue indicating a decrease in signal.

The results from the AR protein LMA, AR transcriptional activity screen, and PSA protein LMA screens were analyzed to identify candidate mimics that inhibited AR expression and activity in multiple assays and cell lines. The top 25% of inhibitory mimics, based on fold signal change, were chosen from each screen and cell line. The resulting data was compared to identify 43 candidates which inhibited AR transcriptional activity, as well as AR and PSA protein level in at least two cell lines. Five additional candidates were considered from the most potent inhibitors of AR transcription activity. A heat map, in Figure 2, displays the resulting 48 candidate mimics and associated FC in AR protein level, PSA protein level, AR transcriptional activity, and cell viability.

### Verification of AR-regulating miRNAs

**Figure 3:**
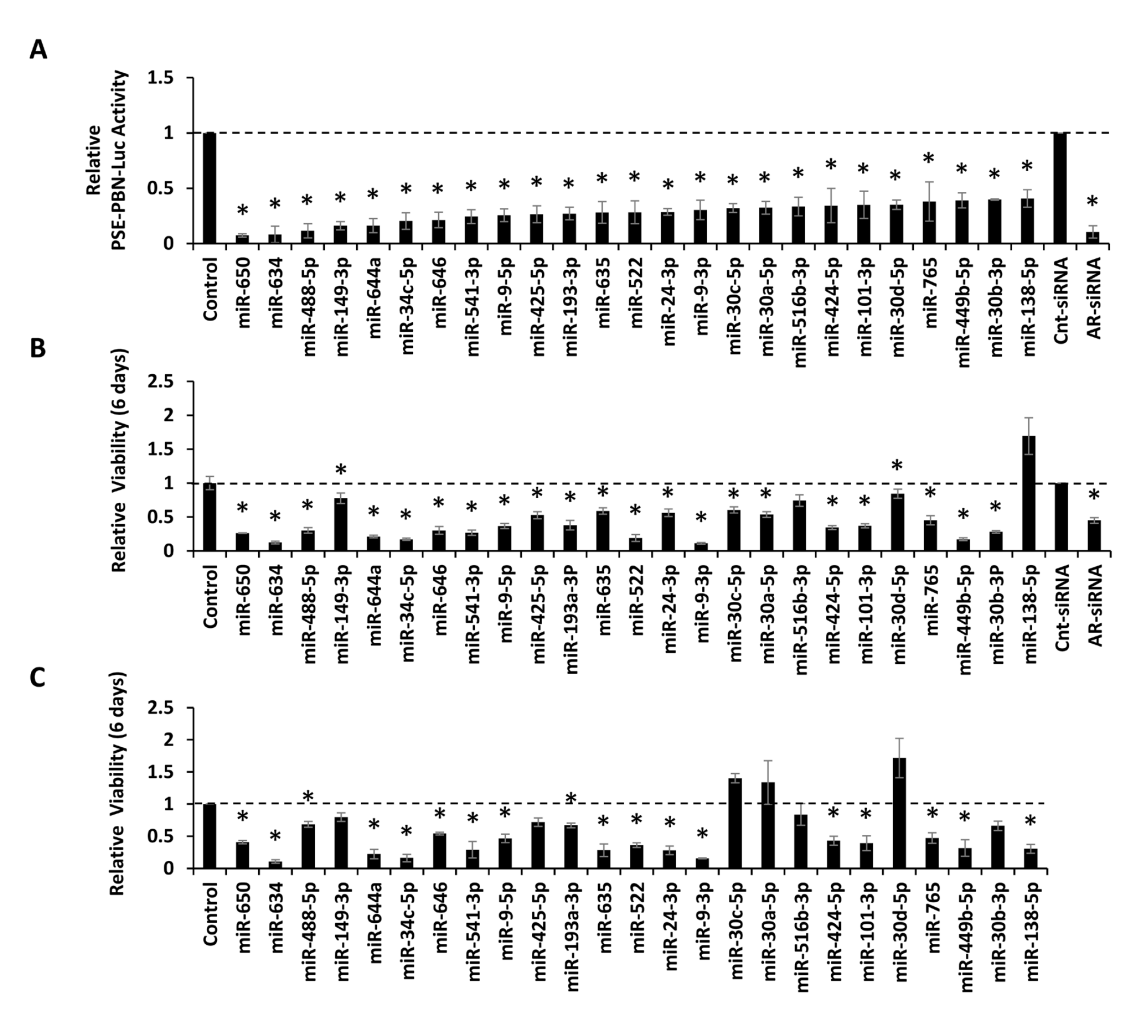
Verification of candidate miRNAs by AR Transcriptional Activity analysis and effects of verified mimics on cell viability A, AR transcriptional activity of the top 25 verified candidates as measured by PSE-PBN-Luc reporter, relative to control miRNA and normalized to *Renilla* luciferase, in LNCaP cells. AR was stimulated 48 hours post transfection by 5 nM R1881, and activity detected 24 hours later. B, Relative viability of LNCaP-MLuc cells, as measured by MLuc Cell Viability Assay, 6 days post transfection with the top 25 verified candidates. C, Relative viability of PC3-MLuc cells, as measured by MLuc Cell Viability Assay, 6 days post transfection with the top 25 verified candidates. Graphs represents average ± Standard Error (SE) from at least two biological replicates performed in triplicate. Dashed line represent control miRNA treated cell activity or viability. Cnt-siRNA, control siRNA, AR-siRNA. Asterisk indicates significant suppression of transcriptional activity or viability. *, p < 0.05.

MiRNA mimics from 39 candidates were separately obtained for verification analyses. In addition, 5 independent mimics from a previous DNA repair screen [22] were analyzed because they were found to inhibit AR protein level or AR activity in at least one AR functional screen. These are specifically miR-101-5p, miR-193a-5p, miR-199a-3p, miR-30a-5p, and miR-516-3p. The resulting 44 mimics were evaluated in AR transcriptional reporter assays in LNCaP cells, utilizing the same AR-responsive PSE-PBN luciferase reporter applied to the initial screen. A positive control AR-targeting siRNA, a negative control siRNA, and a negative control mimic where included for reference. The results verified 39 out of 44 mimics as inhibitors of AR transcriptional activity (Supplementary Figure S4A). The activity of the 25 most potent mimics is provided in Figure 3A. To further evaluate these miRNAs, mimics were transfected into AR-positive LNCaP-MLuc and AR-negative PC3-MLuc cells to determine their influence on cell growth and viability. Of the 25 verified mimics, 23 were found to reduce LNCaP-MLuc cell viability (Figure 3B). Interestingly, 18 out of 25 mimics also significantly reduced viability of the AR-negative PC3-MLuc cells (Figure 3C), reflecting their ability to target additional pathways. 11 of 25 mimics also reduced the viability of a castration resistant, but AR-dependent, derivative of LNCaP cells [23] (Supplementary Figure S4B). The expression level of these 25 miRNAs were evaluated in a publically available miRNA gene expression dataset (GSE21036) for association with PCa progression [24]. After multiple testing correction (FDR < 0.05, minimum fold-change > 1.2), 8 out of the 25 miRNAs were found to be significantly associated with progression to primary and metastatic PCa in this data set (Supplementary Table S5 and Figure S5). Although, these miRNAs were found to be both increased and decreased in this specific dataset.

### Regulation of AR expression by 3′UTR-targeting miRNAs

**Figure 4:**
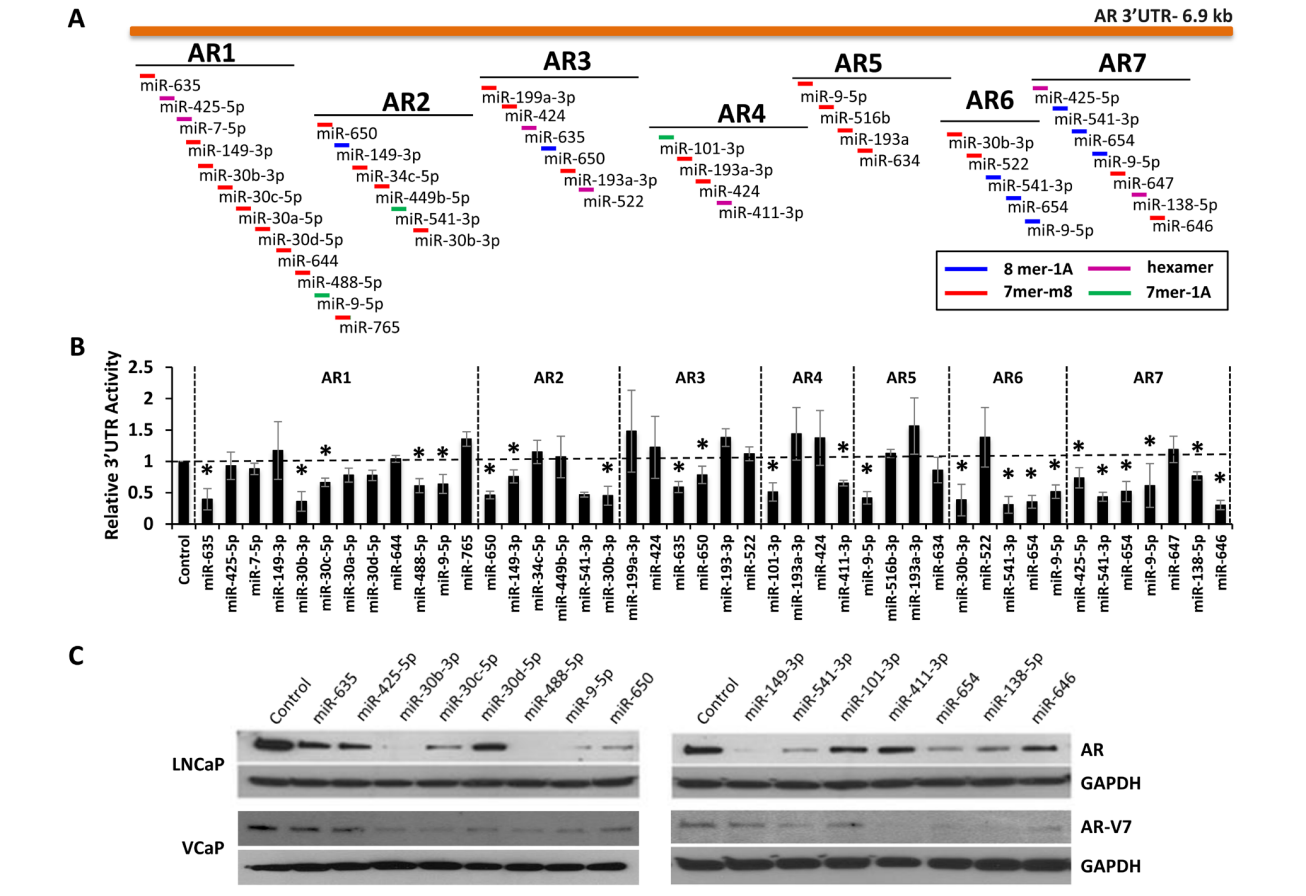
Regulation of AR expression by 3′UTR-targeting miRNAs A, Map of the extended AR 3′UTR (ENST00000396044.7) and corresponding AR 3′UTR reporter amplicons AR1-AR7. MiRNA binding site and type are indicated by color according to legend. B, AR 3′UTR luciferase activity for each amplicon (AR1-AR7), relative to control miRNA mimic and normalized to *Renilla* luciferase, 48 hours post miRNA mimic transfection. Data represents average ± SE, from at least two biologic replicates for each miRNA in triplicate. Dashed line indicates control miRNA. Asterisk indicates significant miRNA suppression of luciferase expression, * p < 0.05. C, Western blot analysis for AR (AR) in LNCaP cells, AR-V7 in VCaP cells, and GAPDH 48 hours post mimic transfection (20 nM).

AR can be alternatively polyadenylated to produce extended 3′UTRs [16]. Complementary seed binding sites for 29 of the 44 analyzed miRNAs were found within a 6.9 Kb AR 3′UTR (ENST00000396044.7). To investigate whether these sites were functional, seven different overlapping regions (AR1-AR7) from the AR 3′UTR were amplified and subcloned downstream of firefly luciferase in the pMIR-REPORT vector (Figure 4A). The resulting vectors were co-transfected into LNCaP cells, with each of the predicted mimics, as well as a constitutively active *Renilla* luciferase vector for normalization. 15 of the 29 mimics significantly suppressed AR 3′UTR reporter activity, when compared to a negative control miRNA (Figure 4B). It is notable that miR-9-5p, miR-30b-3p and miR-541-3p significantly inhibit three or more AR-3′UTR reporters.

Each 3′-UTR-targeting mimic was also evaluated for the ability to knock-down endogenous AR protein expression (Figure 4C). All 15 miRNAs were verified to reduce AR protein levels in LNCaP cells to various degrees. The most potent AR-suppressing miRNAs included miR-9-5p, miR-30b-3p and miR-541-3p. Recently, alternatively spliced isoforms of AR have been discovered in PCa [25-28]. 10 of the 15 miRNAs were also capable of reducing expression of the AR-V7 splice variant in VCaP cells (Figure 4C). The effects of additional AR modifying miRNAs on AR, PSA, and AR-V7 protein expression are provided in Supplementary Figure S6.

### Regulation of AR by miRNAs targeting the coding region

**Figure 5:**
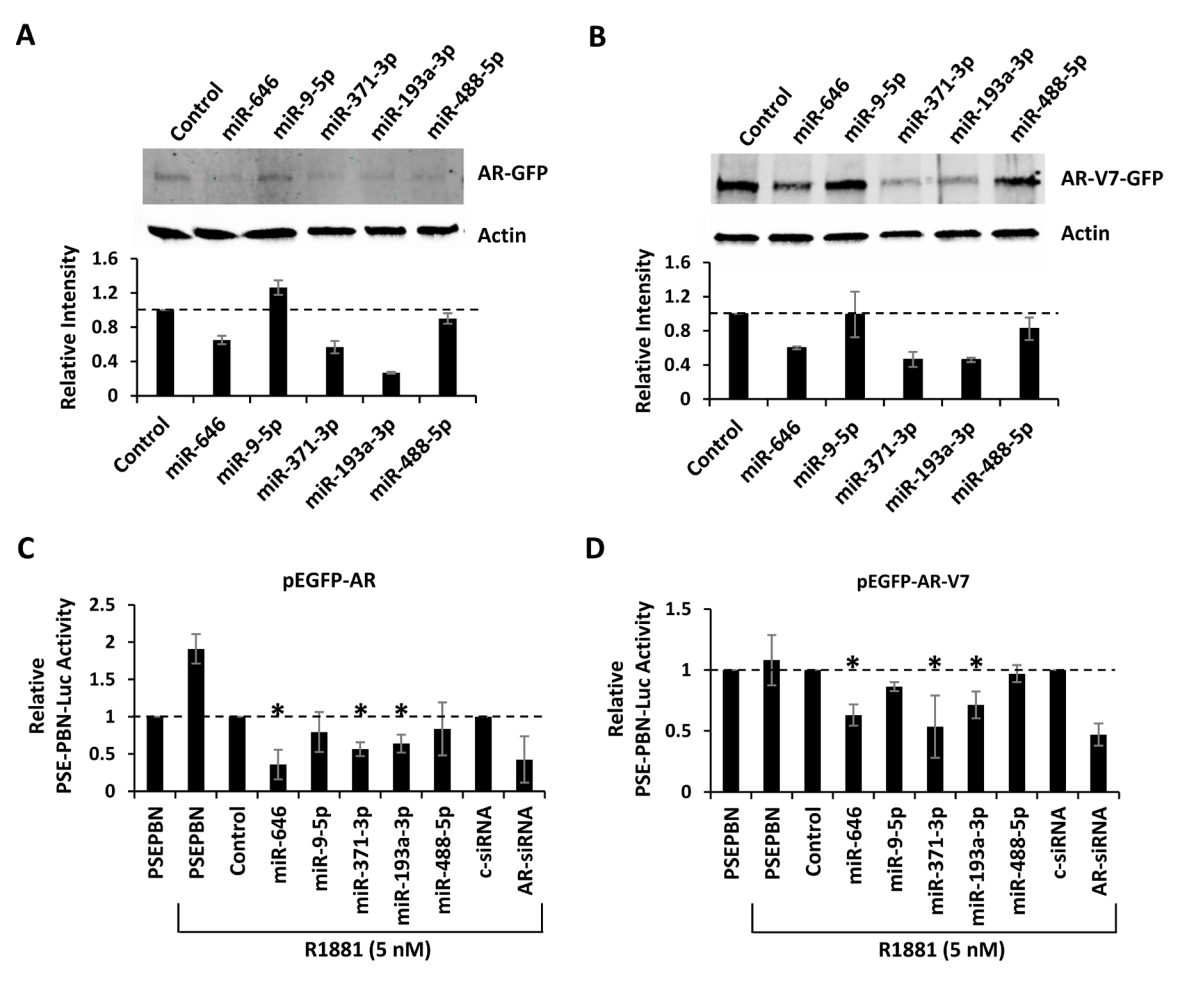
Regulation of AR by miRNAs targeting the coding region A, PC3 cells were co-transfection with pEGFP-AR and miRNA mimics predicted to bind sites within the AR coding region. AR-GFP was detected by western blot 48 hours post transfection. Bar graphs represent β-actin-normalized intensity, relative to control, from two biologic replicates. B, PC3 anti-GFP western blot 48 hours after co-transfection with pEGFP-AR-V7 and miRNA mimics predicted to bind the AR coding region. Bar graphs represents β-actin-normalized intensity, as above. C, AR transcriptional activity, as measured by *Renilla* normalized PSE-PBN-Luc activity, after PC3 transfection with pEGFP-AR and mimics. AR was activated 48 hours after transfection with 5 nM R1881 and activity measured 24 hours later. D, AR transcriptional activity, as measured by *Renilla* normalized PSE-PBN-Luc activity, after PC3 transfection with pEGFP-AR-V7 and mimics. AR activity was stimulated with 5 nM R1881 and activity measured 24 hours later. Bar graphs represent average ± standard deviation (SD) from two biologic replicates. Asterisks indicate significant reduction in AR transcriptional activity, * p < 0.05.

MiRNA-mediated gene suppression is traditionally studied within the 3′UTR of target mRNAs. However, there is evidence that miRNAs can also regulate gene expression through the coding region [29]. Complementary seed sequence binding sites for 5 of the 44 analyzed miRNAs (miR-646, miR-9-5p, miR-371-3p, miR-193a-3p, and miR-488-5p) were found within the coding regions of AR and AR-V7. To study this potential mechanism of regulation, GFP-tagged AR and GFP-tagged AR-V7 expression vectors, which lack the natural AR 3′UTR, were co-transfected with each miRNA mimic into AR-negative PC3 cells. Changes in AR protein expression were quantified by western blot detection of the GFP fusion tag. Three miRNAs (miR-646, miR-371-3p, and miR-193a-3p) notably decreased AR-GFP and AR-V7-GFP protein levels (Figure 5A-5B). To further study this mechanism, the AR expression vectors were co-transfected into PC3 cells with the AR-responsive PSE-PBN luciferase reporter vector, a constitutively active *Renilla* luciferase vector for normalization, and each miRNA mimic. After 48 hours, the full-length AR-GFP was stimulated by addition of the synthetic androgen, R1881. Androgen-induced AR-GFP transcriptional activity was detected 24 hours later (Figure 5C). The ligand-independent AR-V7-GFP variant was active in the presence or absence of R1881 (Figure 5D). The same three AR-targeting miRNAs (miR-646, miR-371-3p, and miR-193a-3p) were found to significantly suppress AR and AR-V7 transcriptional activity. These results indicate that miR-371-3p, miR-193-3p, and miR-646 can suppress AR and AR-V7 protein expression through binding sites within the coding region.

### miR-30b-3p and miR-30d-5p directly suppress AR and PCa cell proliferation

A previously reported high throughput mimic screen, by Östling and colleagues, applied 5 PCa cell lines to an AR protein LMA analysis, and identified 71 AR-regulating miRNAs and 21 verified AR-regulating miRNAs [16]. Comparison of our 44 candidates to this previous study reveals 12 common AR-inhibitors. Of these, 10 miRNAs were validated by both studies: miR-34c, miR-371, miR-449, miR-488-5p, miR-541, miR-634, miR-644, miR-654, miR-9-5p, and miR-9-3p. Eight validated miRNAs from our study were unique: miR-101-3p, miR-149, miR-30b-3p, miR-30c-5p, miR-30d-5p, miR-635, miR-646, and miR-650. It is notable that multiple miR-30 family members are present in this group. MiRanda analysis found that each miR-30 candidate was predicted to target the AR 3′UTR with mirSVR scores < −0.5, indicating a high probability for AR regulation [30]. The 4 candidate miR-30 family members were selected for further detailed studies.

**Figure 6:**
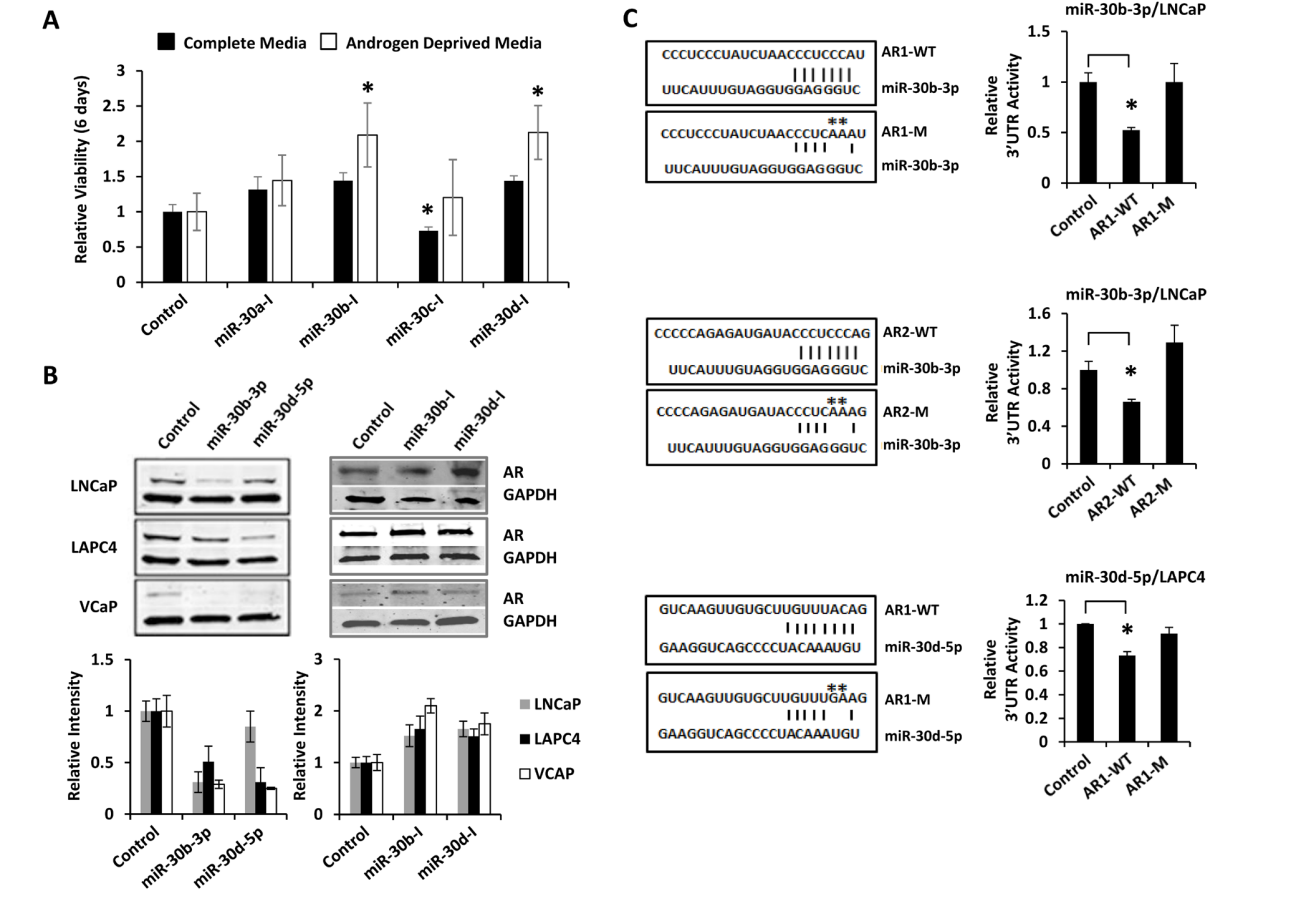
miR-30b-3p and miR-30d-5p directly regulate AR protein expression and PCa cell growth A, LNCaP-MLuc cells were transfected with control or miR-30 antagomirs. Cell viability was determined 6 days post transfection in complete media (with androgens) or in androgen depleted media by MLuc Cell Viability Assay. Antagomirs: miR-30a-I: miR-30a-3p; miR-30b-I: miR-30b-3p; miR-30c-I: miR-30c-5p; miR-30d-I: miR-30d-5p. Asterisk indicated significant increase in viable cell density, *, p < 0.05. B, AR western blot 48 hours post transfection with control, miR-30 mimics, or miR-30 antagomirs (20 nM) in LNCaP, LAPC4, and VCaP cells. Bar graph represents average GAPDH normalized AR intensity average, relative to control. Error bars = SD. C, AR 3′UTR luciferase activity for amplicons AR1 and AR2, before and after site-directed mutagenesis of miR-30 binding sites. Schematic indicates miRNA seed sequence binding sites and mutations (−M) are indicated by asterisks. Top, miR-30b-3p mimics with AR1-WT and AR1-M in LNCaP; Middle, miR-30b-3p mimics with AR2-WT and AR2-M in LNCaP; Bottom, miR-30d-5p mimics with AR1-WT and AR1-M in LAPC4 cells. Bar graphs represent average 3′UTR activity ± SD, normalized to control miRNA mimics from two biologic replicates. Asterisk indicates significant suppression of 3′UTR activity by miRNA mimics, * p < 0.05.

LNCaP cells were transfected with miR-30a-5p, miR-30b-3p, miR-30c-5p, and miR-30d-5p antagomirs to inhibit endogenous miRNA levels. Cell viability was measured after 6 days of growth in complete media (RPMI 1640 + 10% FBS) or in androgen deprived media (Phenol-red free RPMI 1640 + 10% Charcoal-Stripped FBS). Inhibition of miR-30b-3p and miR-30d-5p induced LNCaP cell growth under androgen deprived conditions (Figure 6A). Quantitative RT-PCR confirmed that miR-30b-I and miR-30d-I significantly inhibit miRNA levels in LNCaP cells (Supplementary Figure S7A and S7B). To further interrogate miR-30b-3p and miR-30d-5p as AR-targeting miRNAs, three PCa cell lines (LNCaP, LAPC4, and VCAP) were transfected with miRNA mimics or miRNA antagomirs. The results verified that miR-30b-3p and miR-30d-5p mimics reduced AR protein levels, while miR-30b-3p and miR-30d-5p inhibitors elevated AR protein levels (Figure 6B). These changes in AR protein expression were not associated with significant changes in AR mRNA level, as quantified by qRT-PCR (Supplementary Figure S7C). Repeat transfection studies were then performed with the 3′UTR reporters AR1 and AR2. MiR-30b-3p was verified to directly suppress both 3′UTR reporters. Importantly, this suppression was lost following mutation of miR-30b-3p seed sequence binding sites (Figure 6C). Repeat transfection studies with miR-30d-5p replicated mild, but non-significant, suppression of the AR1 3′UTR reporter in LNCaP cells (Supplementary Figure S8). When these studies were repeated in LAPC4 cells, where miR-30d-5p more potently inhibited AR protein expression, AR1 3′UTR reporter activity was significantly reduced by miR-30d-5p transfection. This suppression was ablated with mutation of the miR-30d-5p seed sequence binding site (Figure 6C). Collectively, these data support direct and endogenous regulation of AR gene expression by miR-30b-3p and miR-30d-5p.

### miR-30 expression in primary PCa, metastatic CRPC, and relationship to AR activity

**Figure 7:**
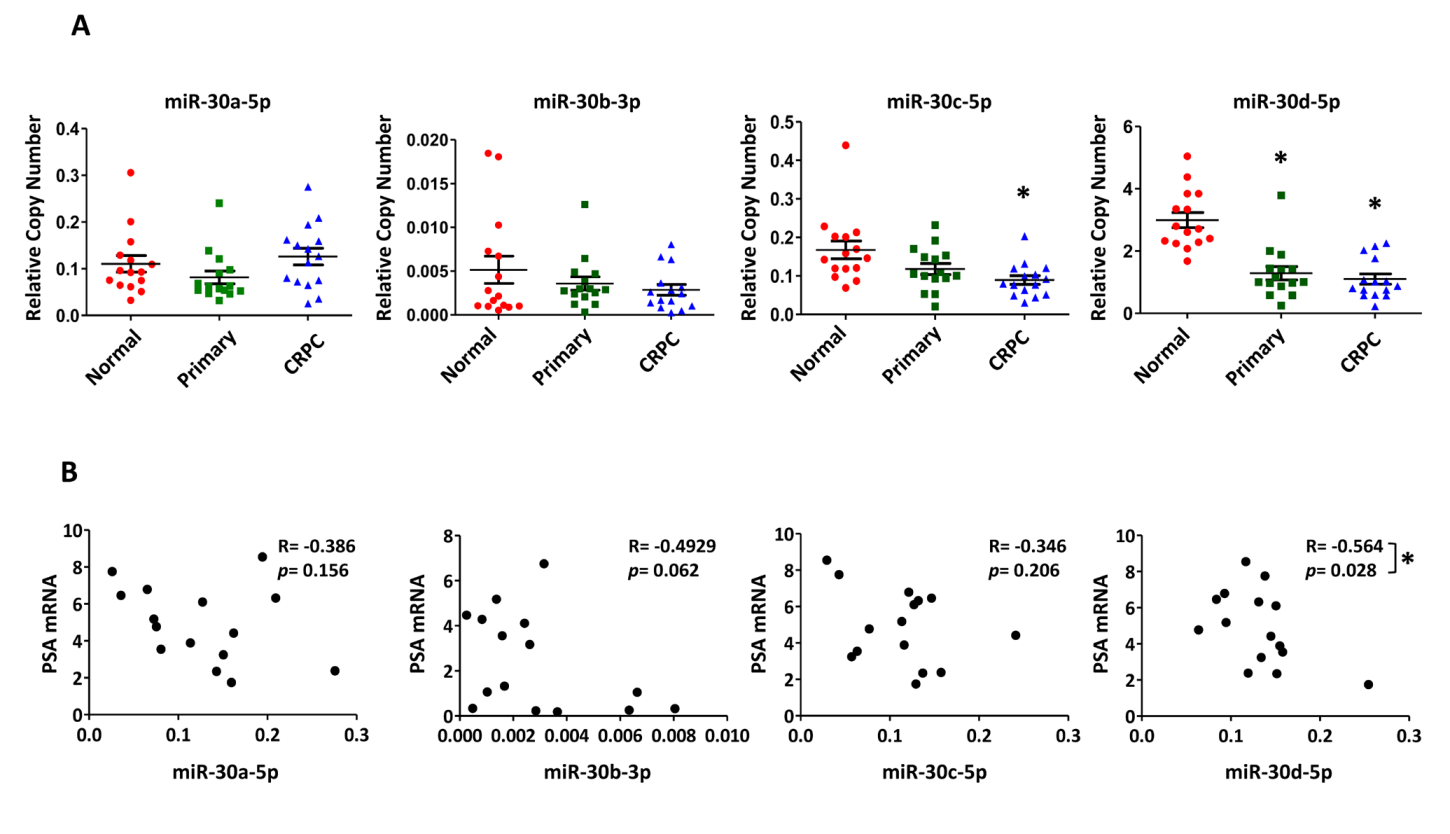
Differential expression of miR-30c-5p and miR-30d-5p in human PCa and correlation with PSA mRNA levels A, Relative miR-30 expression quantified by ddPCR of reverse transcribed miRNA from normal prostate (N=15); primary prostate cancer (N=15) and metastatic CRPC (N=15). Mean ± SEM. Asterisk indicates significantly reduced miR-30 expression, relative to normal prostate, * p < 0.05. B, Correlation analysis of PSA mRNA level, as a surrogate for AR transcriptional activity, and miR-30 miRNA levels in CRPC samples (N = 15). PSA mRNA level determined by qRT-PCR and normalized to GAPDH. Correlation determined by Pearson correlation calculation. R – Pearson r. Asterisk indicates a significant correlation between PSA mRNA and miR-30, * p < 0.05.

To investigate the four candidate miR-30 family members in human disease, miR-30a-5p, miR-30b-3p, miR-30c-5p and miR-30d-5p levels were measured in 15 primary PCa samples, 15 adjacent normal prostate samples, and 15 metastatic CRPC samples by droplet digital RT-PCR. MiRNA expression levels were normalized to RNU6B for each sample. Notably, miR-30d-5p levels were significantly lower in primary and metastatic CRPC, when compared to normal healthy tissue (Figure 7A). MiR-30c-5p levels were also significantly lower in metastatic CRPC.

Levels of PSA mRNA, an AR-induced gene, were quantified by qRT-PCR as a surrogate marker for AR transcriptional activity in the metastatic CRPC samples. A statistically significant inverse correlation (Pearson r = −0.564, p=0.028) was found between miR-30d-5p and PSA mRNA levels (Figure 7B). Similarly, an inverse trend could be observed for PSA mRNA and miR-30a-5p, miR-30b-3p, and miR-30c-5p levels in these same samples. Purified protein was not available from these tissues for direct correlation analyses between miRNA and AR protein levels; however, previous immunohistochemical studies had quantified nuclear AR protein levels from 10 of the 15 metastatic CRPC cases. When nuclear AR expression was compared to PSA mRNA level in these 10 samples, a significant correlation was observed (Pearson r = 0.6442, p = 0.049), indicating that PSA mRNA level was a reasonable surrogate marker for nuclear AR protein (Supplementary Figure S9A). This was supported by a complementary inverse trend in AR nuclear protein levels, for all of the studied miR-30 family members (Supplementary Figure S9B-E).

## DISCUSSION

Aberrant AR expression and activity are associated with PCa development and its progression through all stages of the disease [31]. Altered AR signaling can be detected early in primary PCa [24], and its transcriptional activity can directly contribute to chromosomal breaks and rearrangements [32, 33]. As the disease progresses to metastasis and castration resistance, following ADT, the AR gene is commonly mutated or amplified [24, 34]. In addition to these genetic aberrations, elevated AR activity can be caused by anomalous androgen production, signal transduction cross-talk, altered AR co-factor expression or activity, or alternative AR splicing [5-7]. The potential role of miRNAs as regulators of AR protein expression and signaling in metastatic CRPC have not yet been fully characterized.

Previous studies have reported direct miRNA suppression of the AR. One of the first reported was miR-488* (miR-488-5p), which targets the AR through its 3′UTR [11]. Our studies, as well as those by Östling et al. [16], validate miR-488* as a direct AR-targeting miRNA by 3′UTR reporter assay and western blotting. Further, our data support that miR-488* suppresses AR-V7 protein expression. Hagman and colleagues found miR-205 to be an AR-targeting miRNA with reduced expression in PCa that later failed ADT [12]. MiR-205 was identified as a candidate AR-targeting miRNA in screens (Figure 2), and it was verified to inhibit AR transcriptional activity (Supplementary Figure S4A). Both miR-185 and miR-124 have also been reported as AR-regulating miRNAs [14, 35]. These were not identified as top candidates in our study; nonetheless, both reduced AR protein expression in the three applied PCa cell lines and they reduced AR transcriptional reporter activity in the initial screens (Supplementary Tables 1 and 2). MiR-185 was also found to be an AR-targeting miRNA in the LMA screen by Östling et al. [16]. MiR-145 has also been reported to reduce AR mRNA and protein levels, to influence PCa cell growth, and to be significantly down-regulated in aggressive PCa [36]. MiR-145 was not considered to be a candidate in our screens, or those from Östling and colleagues, however it was found to moderately reduce AR protein levels in LNCaP, VCaP, and LAPC4 cells (Supplementary Table S1).

Östling and colleagues demonstrated the value of protein LMAs for the identification of functional miRNA mimics [16]. Our screen complements this initial discovery by evaluating AR protein levels in LNCaP and LAPC4 cells, and it brings additional analyses from VCaP protein lysates, PSA protein LMAs, AR transcriptional reporter activity, and LNCaP-MLuc cell viability. While these two studies applied some different cell lines and different criteria for normalization and candidate selection, 12 common AR-regulating candidates were identified and 10 were validated by at least one functional assay in both studies. Additional high throughput miRNA mimic studies have been recently reported in PCa models. Larne et al. screened a library of mimics for PSA protein level changes in LNCaP and LAPC4 cells. Their results identified 30 PSA-suppressing miRNAs. Five miRNAs were common with our candidate AR-regulating miRNAs: miR-30b-3p, miR-34c, miR-644, miR-654, and miR-765. MiRanda target prediction analysis did not identify any PSA 3′UTR binding sites for miR-30b-3p, miR-34c, or miR-765, suggesting that these miRNAs likely suppress PSA expression through AR regulation. Seed binding sites for miR-644 and miR-654 were identified in the PSA 3′UTR, but mirSVR scores were > −0.5 (data not shown). Binding sites for other miR-30-5p family members were also absent in miRanda analysis of the PSA 3′UTR. Finally, the high throughput analysis of miRNA-mediated effects on LNCaP cell viability provide some complementary data to a recent study by Aakula et al., which measured the effects of mimics on the proliferation and survival of 5 PCa cell lines by CellTiter Glo viability assay, as well as Ki67 and cPARP reverse phase protein arrays [37]. Both studies found miR-191-3p and miR-381 to be potent growth stimulators, and miR-193a, miR-708, miR-885-3p, and miR-876 to be potent growth inhibitors (Supplementary Table S3).

In addition to these reported miRNAs, our high throughput analyses have uncovered some novel AR-regulating miRNAs. MiR-101 is a tumor suppressive miRNA that is commonly lost during PCa progression and it is associated with over-expression of its target gene, EZH2 [38]. Here we found the more commonly expressed isoform of miR-101, miR-101-3p, to target and mildly suppress AR and AR-V7 protein expression, AR transcriptional reporter activity, and PCa cell viability. Several members of the miR-30 family were also identified as AR-suppressive miRNAs. MiR-30b-3p and miR-30d-5p were verified as direct AR targeting miRNAs through 3′UTR reporter analyses and site directed mutagenesis (Figure 6C, 6D). MiR-30b and miR-30d are located in close proximity on the long arm of Chromosome 8, although it is not clear whether they share a common primary transcript or if they are separately regulated. MiR-30b expression is negatively regulated by EGFR and Src signaling in PCa [39]. MiR-30b post-transcriptional processing has further been found to be inhibited by growth suppressing signals at epithelial apical zonula adherens [40]. There have been conflicting reports of miR-30 expression in PCa. Some studies have found miR-30b-3p and miR-30d-5p to be elevated in PCa and CRPC [41, 42]. This was observed in our analysis of the GSE21036 dataset (Supplementary Figure S5). At the same time, others studies have found miR-30b-3p and miR-30d-5p to be reduced in PCa [39, 43]. Our focused ddPCR analyses found miR-30c-5p and miR-30d-5p levels to be significantly reduced in primary PCa, and miR-30d-5p levels were significantly reduced in metastatic CPRC (Figure 7A). These differences in quantification may be associated with tissue processing, RNA isolation methods, miRNA quantification, or miRNA normalization techniques [44].

There have been relatively few studies analyzing miRNA expression in advanced PCa, such as metastatic CRPC, where altered AR expression and activity would be most expected. Analysis of primary tumors isolated by surgery or transurethral resection of the prostate (TURP) have found reduced expression of AR-targeting miRNAs and corresponding elevated AR protein expression or AR transcriptional activity [12, 14, 36, 45]. However, to our knowledge, correlative studies of AR-targeting miRNAs and AR activity have not yet been studied in metastatic CRPC tissue. Here we observed significant down-regulation of miR-30c-5p and miR-30d-5p in metastatic CRPC tissue by droplet digital qRT-PCR. Moreover, miR-30d-5p expression was inversely correlated with expression of the AR-regulated transcript, PSA (Figure 6B). Inverse trends between PSA mRNA levels and miR-30a, miR-30b and miR-30c expression were also observed, and these were consistent with inverse levels of nuclear AR protein staining. Further studies of AR regulating miRNAs in larger sample sets of metastatic CRPC may shed better light on their relevance in human disease and AR signaling.

High throughput gene expression studies have also evaluated miRNA expression in CRPC tissues [42, 46, 47]. Porkka and colleagues reported fifteen miRNAs that were selectively decreased in hormone refractory PCa specimens isolated by TURP [47]. Five of these fifteen miRNAs were considered to be AR-suppressive in our study (miR-19b, miR-30a, miR-30b, miR-30c and miR-205). Another report by Goto et al., specially focused on the miRNA expression signature of CRPC samples obtained at autopsy [46]. Nine of the AR suppressive miRNAs identified our study (miR-143, miR-149, miR-152, miR-205, miR-23b, miR-24, miR-30a, miR-30e, and miR-660) were found to be down regulated in CRPC samples, when compared to normal prostate. Three AR-suppressing miRNAs (miR-101, miR-30d, and miR-650) were found to be down-regulated in CRPC when compared to primary PCa [46]. These results support our observation of reduced miR-30d-5p in CRPC.

In summary, we have applied a series of complementary functional miRNA mimic screens to identify multiple miRNAs capable of inhibiting or activating the AR signaling axis. The results demonstrated a common suppressive nature for miRNAs across many assays. At the same time, potent and specific AR regulating miRNAs were identified and verified in multiple models, assays and clinical samples. These results provide a comprehensive evaluation of AR-regulating miRNAs and AR function in PCa cell models. Importantly, these results implicate miR-30 family members as possible regulators of the AR signaling axis in CRPC. Additional and detailed studies are required to further determine the clinical significance of these miRNAs in PCa development and progression.

## MATERIALS AND METHODS

Materials. Material details and catalog numbers are provided in Supplementary Information.

### Cell culture

Human PCa cell lines LNCaP, DU145 and PC3 were purchased from ATCC (Manassas, VA). LAPC4 and VCaP cells were provided by John Isaacs (Johns Hopkins; Baltimore, MD). Cells were obtained between 2006-2010. LNCaP-MLuc and PC3-MLuc cells stably express *Metridia* Luciferase (MLuc) driven by the β-actin promoter and enhancer [18]. Conditions: LNCaP and PC3 - RPMI 1640, VCaP - DMEM, LAPC4 - IMDM plus 1 nM R1881, LNCaP-MLuc and PC3-MLuc - RPMI 1640 plus 5 μg/ml Blasticidin, each supplemented with 10% FBS and 5 μg/ml Ciprofloxacin and maintained at 37°C and 5% CO_2_. Cells verified mycoplasma free annually by MycoAlert Mycoplasma Detection Kit (Lonza; Anaheim, CA). LNCaP, LAPC4, DU145 and PC3 were authenticated by DDC medical (Fairfield, OH), November of 2014.

### Protein lysate microarray screens

The Dharmacon miRIDIAN® microRNA Mimic Library was purchased from the Johns Hopkins High Throughput Biology - HIT Center (Baltimore, MD). Cells were Lipofectamine (Life Technologies; Grand Island, NY) transfected with each mimic (20 nM) in 24 well plates. Protein lysates were harvested 48 hours later and transferred to 384 plates. Dilutions (1, 1:4, and 1:16) were generated by Microlab STARlet liquid handling robotics system (Hamilton, Reno, NV). Triplicate spots were printed on nitrocellulose-coated Fast slides (Main Manufacturing; Sanford, ME) by a 4-tip GeSim Nano-Plotter 2 robotics system (Grosserkmannsdorf, Germany). Each LMA included control mimic transfection and PC3 and DU145 as AR and PSA negative controls. LMAs were immunoblotted for AR (AR441), PSA (A0562), and GAPDH (G9545) overnight at 4°C and detected by LI-COR Odyssey imaging system (Lincoln, NE). Antibody dilutions were established in pilot studies (Supplementary Figure S1). For each protein, spots from a single dilution (within the linear range) were quantified in triplicate using LI-COR Odyssey spot selection and quantification software. GAPDH-normalized signal was recorded as fold-change relative to control miRNAs (Supplementary Table S1). Mimics with negative values were removed from analyses.

### AR transcriptional activity screen and verification

LNCaP cells were Lipofectamine reverse-transfected in triplicate with the miRNA library (20 nM), an AR-responsive firefly luciferase reporter (pBK-PSE-PBN-Luc) driven by the probasin (PBN) promoter and the PSA enhancer (PSE), and pRL-CMV *Renilla* luciferase (100 ng DNA total) in 96 well plate format in phenol-red-free medium supplemented with 10% charcoal stripped serum. Anti-AR and control FlexiTube siRNA (Qiagen; Gaithersburg, MD) were included (20 nM) in each plate. 48 hours later, cells were treated with 5 nM R1881 and firefly luciferase activity determined 24 hours later, an established endpoint for androgen-induced transcriptional activity [48]. *Renilla* normalized activity was quantified by a non-commercial dual luciferase assay [49], on a Wallac Microbeta luminometer (Perkin Elmer; Waltham, MA). Activity is relative to negative control siRNA (Supplementary Table S2). For verification mimics were separately purchased and analyzed for miRNA-induced changes in AR transcriptional activity, as described above, in LNCaP cells.

### Cell viability screen and verification

Cell viability was determined by MLuc Viability Assay as previously described [18]. Briefly, 2,000 LNCaP-MLuc cells were previously reverse-transfected with 810 mimics (20 nM) in 96 well plate format (quadruplicate) [22]. MLuc activity was measured in 50 μl of supernatant 6 days post transfection, an established time point for androgen-induced growth or ADT response [21], in *Renilla* buffer by Wallac Microbeta luminometer. Cell viability 11 days post transfection has been previously reported [22]. Fold change (FC) in viability is relative to control miRNA (Supplementary Table S3). For verification candidate mimics or antagomirs were separately purchased and LNCaP-MLuc cell viability performed as described above (20 nM). Anti-AR siRNA and control siRNA were included.

### Immunoblotting

SDS-PAGE and immunoblotting were performed as previously described [50]. Anti-AR (AR-N20), GFP (sc-8334), PSA (A0562), β-actin (A5442), and GAPDH (G9545) primary antibodies were applied and detected with IRdye 800CW, IRdye 680LT, or Anti-mouse IgG HRP (A4416) secondary antibodies. Bands were imaged by ECL chemiluminescence or on the LI-COR Odyssey imaging system. Quantification was performed by LI-COR Odyssey under non-saturating conditions, relative to β-actin or GAPDH. Mimics and antagomirs (20 nM) were lipofectamine transfected.

### AR 3′UTR reporter constructs and luciferase assay

AR 3′UTR regions were PCR amplified (Supplementary Table S4) from LNCaP genomic DNA and subcloned downstream of firefly luciferase in pMIR-REPORT (AddGene; Cambridge, MA). Site directed mutagenesis, by the QuickChange II kit (Agilent Technologies; Santa Clara, CA) was applied for binding site mutations. LNCaP cells were Lipofectamine co-transfected with mimics (20 nM), AR 3′UTR reporters, and pRL-CMV luciferase vectors (100 ng total). After 48 hours, reporter activity was assessed by dual luciferase enzyme assay and normalized to *Renilla* luciferase [22, 49].

### AR coding region analysis

pEGFP-AR and pEGFP-AR-V7 have been previously described [26]. Plasmid DNA (100 ng) and mimics (20 nM) were Lipofectamine co-transfected into PC3 cells and AR detected by anti-GFP Western blot. AR transcriptional activity was determined by *Renilla* normalized firefly luciferase activity in cells Lipofectamine co-transfected with mimics or siRNAs, pBK-PSE-PBN-Luc, and pRL-CMV in RPMI 1640 plus charcoal stripped FBS. 48 hours later, cells were treated with 5 nM R1881 and AR transcriptional activity determined 24h later by dual luciferase enzyme assay [22, 49], as described above.

### qRT-PCR analysis

Total RNA from normal prostate (n=15), primary PCa (n=15) and metastatic CRPC (n=15) was provided by the Prostate Cancer Biorepository Network (University of Washington; Seattle, WA). CRPC (n=15) were isolated from adrenal (n=1), lymph node (n=4), diaphragm (n=2), lung (n=1) and liver (n=7) metastases. TaqMan MicroRNA RT (ThermoFisher Scientific; Waltham, MA) for miRNAs and RNU6B was followed by ddPCR on Bio-Rad's QX200 ddPCR System, according to manufacturer protocol. Expression was RNU6B normalized. For PSA, cDNA was generated using QuantiTect Reverse Transcription Kit (Qiagen; Gaithersburg, MD) and qPCR was performed using Invitrogen SYBR GreenER qPCR SuperMix (ThermoFisher Scientific; Waltham, MA) on the ABI 7900 HT Real Time PCR System. β-actin was used for normalization. Primers are included in Supplementary Table S4.

### Statistical analyses

Differences between groups were evaluated by Student's t-test, relative to negative or untreated control. Spearman correlation calculations were applied to gene expression studies. Statistical significance was assigned with p < 0.05.

## SUPPLEMENTARY MATERIALS METHODS, FIGURES AND TABLES


