# Supplementary material for: Identification of miR-30b-3p and miR-30d-5p as direct regulators of androgen receptor signaling in prostate cancer by complementary functional microRNA library screening
Source: Oncotarget. 2016 Sep 24;7(45):72593–607. doi: 10.18632/oncotarget.12241 (PMC5341930; doi:10.18632/oncotarget.12241)
Supplement: Supplementary file 1 [file oncotarget-07-72593-s001.docx]

Identification of miR-30b-3p and miR-30d-5p as direct regulators of Androgen Receptor Signaling in Prostate Cancer by complementary functional microRNA library screening

**Supplementary Information**

**Supplementary Materials and Methods**

**Additional material details not provided in manuscript.**

Antibodies:

AR441- sc-7305-Santa Cruz Biotechnology, USA

AR-N20-sc-816- Santa Cruz Biotechnology, USA

Anti-GFP-sc-8334- Santa Cruz Biotechnology, USA

Anti-actin-A5441- Sigma Aldrich, USA

Anti-GAPDH-G9545- Sigma Aldrich, USA

Anti- Mouse IgG secondary antibody-A4416-Sigma, USA

PSA-A0562-DAKO, Denmark

IRdye 800CW secondary antibodies- 926-322210- LI-COR Biosciences-USA

IRdye 680LT secondary antibodies- 926-68021- LI-COR Biosciences–USA.

Cell culture and transfection materials:

RPMI1640- Mediatech, VA, USA

IMDM-Gibco, NY, USA

DMEM-Glutamax- Gibco, NY, USA

Ciprofloxacin Hydrochloride -C5075, US Biological; Salem, MA

Flexi Tube AR siRNA- SI02757258- Qiagen-USA

Flexi Tube control siRNA-1022076-Qiagen-USA

Lipofectamine 2000-11668-019- Invitrogen, USA

R1881- NLP005005MG- Perkin Elmer-USA

Fetal Bovine Serum-F4135- Sigma Aldrich, USA

Fetal Bovine Serum Charcoal/Dextran Treated – SH30068 – GE Healthcare Life Sciences- USA

Cell Titer 96 aqueous one solution Cell Proliferation Assay-G358B-Promega, USA

Passive Lysis Buffer-E194A- Promega, USA

ECL prime Western Blotting Detection Reagent, RPN2232, GE Healthcare, USA

Lysate Microarray

FAST Slide- Maine Manufacturing, Sanford, ME, USA

Dharmacon miRIDIAN® microRNA Mimic Library - CS-001005, Lot 08123

miRNAs mimics for verification- GE Dharmacon, USA:

miRNA negative control mimic #2 CN-00200-01-05

hsa-miR-138 MI0000476

hsa-miR-143 MI0000459

hsa-miR-149* MI0000478

hsa-miR-19b MI0000075

hsa-miR-200a MI0000737

hsa-miR-205 MI0000285

hsa-miR-218 MI0000294

hsa-miR-24 MI0000080

hsa-miR-299-5p MI0000744

hsa-miR-30b* MI0000441

hsa-miR-30c MI0000736

hsa-miR-30d MI0000255

hsa-miR-30e-3p MI0000749

hsa-miR-335 MI0000816

hsa-miR-34c MI0000743

hsa-miR-371 MI0000779

hsa-miR-411 MI0003675

hsa-miR-424 MI0001446

hsa-miR-425-5p MI0001448

hsa-miR-449b MI0003673

hsa-miR-488* MI0003123

hsa-miR-515-3p MI0003147

hsa-miR-522 MI0003177

hsa-miR-539 MI0003514

hsa-miR-541 MI0005539

hsa-miR-634 MI0003649

hsa-miR-635 MI0003650

hsa-miR-640 MI0003655

hsa-miR-644 MI0003659

hsa-miR-646 MI0003661

hsa-miR-647 MI0003662

hsa-miR-650 MI0003665

hsa-miR-651 MI0003666

hsa-miR-654 MI0003676

hsa-miR-7 MI0000265

hsa-miR-765 MI0005116

hsa-miR-873 MI0005564

hsa-miR-9 MI0000467

hsa-miR-9* MI0000466

miRNAs inhibitors- GE Dharmacon, USA:

IH-300505-05-mir-30a-5p

IH-301045-05-mir-30b-3p

IH-300542-05-mir-30c-5p

IH-300543-05-mir-30d-5p

IN-001005-05-control

Cloning:

pBK-PSE-PBN-Luc - provided by Ronald Rodriguez (Johns Hopkins; Baltimore, MD).

pRL-CMV - provided by Ronald Rodriguez (Johns Hopkins; Baltimore, MD).

pMIR-REPORT vector- AddGene, -USA

Quick change II xL site directed mutagenesis kit-200521-5-Agilient Technologies-USA. Restriction Enzymes, New England Biolabs, USA

***Renilla* Luciferase Buffer.** 1.1 M NaCl, 2.2 mM Na_2_EDTA, 0.22 M K_x_PO_4_ (pH 5.1), 0.44 mg/mL BSA, 1.3 mM NaN_3_, 1.43 µM coelenterazine.

**miRNA candidate selection**. Normalized data from three complementary miRNA library screens (AR Transcriptional Activity, AR LMA, and PSA LMA) were analyzed to identify common functional miRNA mimics. Values from each screen were sorted from smallest to largest value for each individual miRNA mimic. Any miRNA mimic with a negative value in any assay was eliminated from further analysis. The top 25% of inhibitory miRNA mimics were identified in each category, and common miRNA mimics which inhibited AR transcriptional activity, as well as AR and PSA protein level in at least two cell lines, were considered candidates. In addition the top five miRNA mimics that inhibited AR transcriptional activity, but were not already considered candidates, were included for further analysis.

**Analysis of differential miRNA expression.** The dataset from Taylor et al ([1](#_ENREF_1)) was used to study miRNA expression in clinical phenotypes. Raw expression data files were downloaded from the NCBI Gene Expression Omnibus database (series GSE21036, part of the GSE21032 super series, also accounting for paired mRNA expression and copy number data), while complete phenotype was obtained from the supplementary information associated to the original manuscript ([1](#_ENREF_1)). This miRNA expression dataset contains 142 samples (cell line=1, Metastasis=14, Primary=99, and adjacent normal=28) and expression levels for 723 unique human miRNA. Pre-processing and normalization were performed essentially as previously described ([2-4](#_ENREF_2)) using statistical packages from the R/Bioconductor project ([5](#_ENREF_5)). Briefly, after quantile normalization, a fixed effects model was fit to each miRNA to estimate expression differences between groups (primary tumors *versus* adjacent normal and metastasis *versus* primary tumors), and an empirical Bayesian approach was applied to moderate log2 fold-change standard errors. Out of 142 samples, the cell line and 13 patients who received neo-adjuvant hormonal or radiotherapy treatment were excluded from differential miRNA expression analysis. Multiple testing corrections were performed within and across comparisons using the Benjamini–Hochberg method ([6](#_ENREF_6)). Statistical significant candidates were observed only at FDR of 0.05 or less.

**LNCaP-95 Cell Viability Studies.** LNCaP-95 cells were provided by Dr. Alan Meeker (Johns Hopkins, Baltimore, MD). LNCaP-95 cells are androgen-independent, but AR-dependent, derivative of LNCaP cells (7). Cells were Lipofectamine co-transfected with mimics (5 nM) in 96 well plate format. Six days post transfection, cell viability was determined by Cell Titer 96 aqueous one solution Cell Proliferation Assay according to manufacturer instructions (4 hour incubation, absorbance at 570 nm).

**Supplementary References**

1. Taylor BS, Schultz N, Hieronymus H, Gopalan A, Xiao Y, Carver BS, et al. Integrative genomic profiling of human prostate cancer. Cancer cell 2010;18(1):11-22.

2. Kortenhorst MS, Wissing MD, Rodriguez R, Kachhap SK, Jans JJ, Van der Groep P, et al. Analysis of the genomic response of human prostate cancer cells to histone deacetylase inhibitors. Epigenetics : official journal of the DNA Methylation Society 2013;8(9):907-20.

3. Ross AE, Marchionni L, Phillips TM, Miller RM, Hurley PJ, Simons BW, et al. Molecular effects of genistein on male urethral development. The Journal of urology 2011;185(5):1894-8.

4. Munari E, Marchionni L, Chitre A, Hayashi M, Martignoni G, Brunelli M, et al. Clear cell papillary renal cell carcinoma: micro-RNA expression profiling and comparison with clear cell renal cell carcinoma and papillary renal cell carcinoma. Human pathology 2014;45(6):1130-8.

5. Gentleman RC, Carey VJ, Bates DM, Bolstad B, Dettling M, Dudoit S, et al. Bioconductor: open software development for computational biology and bioinformatics. Genome biology 2004;5(10):R80.

6. Benjamini Y, Hochberg Y. Controlling the False Discovery Rate - a Practical and Powerful Approach to Multiple Testing. J Roy Stat Soc B Met 1995;57(1):289-300.

7. Sprenger CC, Plymate SR. The link between androgen receptor splice variants and castration-resistant prostate cancer. Hormones & cancer 2014;5(4):207-17.

| Supplementary table S1. Relative AR and PSA protein levels 48h post miRNA transfection as determined by protein lysate microarray immunoblotting. Data represent GAPDH-normalized protein levels, relative to control miRNA transfection. Cell lines are indicated in parentheses. | | | | | | | | |
| --- | --- | --- | --- | --- | --- | --- | --- | --- |
|  |  |  |  |  |  |  |  |  |
|  |  |  |  |  |  |  |  |  |
| **miRNA** | **miR Base** | **Sequence** | **AR** | **PSA** | **AR** | **PSA** | **AR** | **PSA** |
|  | **accession** |  | **(LNCAP)** | **(LNCAP)** | **(VCAP)** | **(VCAP)** | **(LAPC4)** | **(LAPC4)** |
|  | **number** |  |  |  |  |  |  |  |
| hsa-let-7a | MI0000061 | UGAGGUAGUAGGUUGUAUAGUU | 0.4565 | 0.6596 | 1.0175 | 1.2044 | 0.0894 | 0.4414 |
| hsa-let-7a | MI0000062 | UGAGGUAGUAGGUUGUAUAGUU | 0.7814 | 0.9182 | 1.0727 | 1.3615 | 0.4313 | 0.7327 |
| hsa-let-7a | MI0000060 | UGAGGUAGUAGGUUGUAUAGUU | 1.1109 | 0.9208 | 1.2255 | 1.4563 | 0.464 | 0.7862 |
| hsa-let-7a* | MI0000062 | CUAUACAAUCUACUGUCUUUC | 0.6909 | 0.3246 | 0.4816 | 0.331 | 0.5974 | 0.2661 |
| hsa-let-7a* | MI0000060 | CUAUACAAUCUACUGUCUUUC | 0.733 | 0.397 | 0.5769 | 0.5513 | 0.8361 | 0.5692 |
| hsa-let-7b | MI0000063 | UGAGGUAGUAGGUUGUGUGGUU | 1.0406 | 1.033 | 1.0722 | 1.205 | 1.0443 | 1.545 |
| hsa-let-7b* | MI0000063 | CUAUACAACCUACUGCCUUCCC | 0.4278 | 0.5087 | 0.6024 | 0.8237 | 1.4922 | 0.4204 |
| hsa-let-7c | MI0000064 | UGAGGUAGUAGGUUGUAUGGUU | 0.6999 | 0.9211 | 0.8635 | 0.7969 | 0.0273 | 0.5917 |
| hsa-let-7c* | MI0000064 | UAGAGUUACACCCUGGGAGUUA | 0.7237 | 0.6795 | 0.463 | 0.8846 | 0.6691 | 0.6162 |
| hsa-let-7d | MI0000065 | AGAGGUAGUAGGUUGCAUAGUU | 1.0338 | 2.3173 | 2.2233 | 0.9008 | 0.4731 | 0.951 |
| hsa-let-7d* | MI0000065 | CUAUACGACCUGCUGCCUUUCU | 0.6688 | 0.7009 | 0.6757 | 0.7772 | 0.9513 | 0.8764 |
| hsa-let-7e | MI0000066 | UGAGGUAGGAGGUUGUAUAGUU | 1.5199 | 2.6111 | 0.8299 | 0.9069 | 1.1722 | 2.073 |
| hsa-let-7e* | MI0000066 | CUAUACGGCCUCCUAGCUUUCC | 1.0817 | 0.9348 | 1.3356 | 0.9688 | 1.094 | 0.9713 |
| hsa-let-7f | MI0000068 | UGAGGUAGUAGAUUGUAUAGUU | 0.345 | 0.6702 | 0.4242 | 0.7364 | 0.4162 | 1.1041 |
| hsa-let-7f | MI0000067 | UGAGGUAGUAGAUUGUAUAGUU | 0.7858 | 1.5184 | 0.5782 | 0.9337 | 1.0362 | 1.7518 |
| hsa-let-7f-1* | MI0000067 | CUAUACAAUCUAUUGCCUUCCC | 0.6756 | 0.8283 | 0.6225 | 0.7371 | 0.4467 | 0.3589 |
| hsa-let-7f-2* | MI0000068 | CUAUACAGUCUACUGUCUUUCC | 0.7588 | 0.6178 | 0.4703 | -0.6699 | 0.7283 | 0.5157 |
| hsa-let-7g | MI0000433 | UGAGGUAGUAGUUUGUACAGUU | 0.922 | 1.2182 | 0.5731 | 0.7877 | 0.9294 | 1.0406 |
| hsa-let-7g* | MI0000433 | CUGUACAGGCCACUGCCUUGC | 0.7088 | 0.3615 | 0.5775 | 0.928 | 0.4569 | 0.7373 |
| hsa-let-7i | MI0000434 | UGAGGUAGUAGUUUGUGCUGUU | 1.3857 | 2.5042 | 0.6754 | 0.9859 | 0.7544 | 1.1319 |
| hsa-let-7i* | MI0000434 | CUGCGCAAGCUACUGCCUUGCU | 0.833 | 1.9004 | 0.7069 | 2.0536 | -250.332 | 56.804 |
| hsa-miR-1 | MI0000437 | UGGAAUGUAAAGAAGUAUGUAU | 0.783 | 0.5475 | 0.4832 | 0.6911 | 0.4075 | 0.5367 |
| hsa-miR-1 | MI0000651 | UGGAAUGUAAAGAAGUAUGUAU | 0.9025 | 1.9675 | 0.5302 | 0.7039 | 0.5471 | 0.7568 |
| hsa-miR-100 | MI0000102 | AACCCGUAGAUCCGAACUUGUG | 0.6312 | 1.4834 | 0.733 | 0.5288 | 0.5999 | 0.9837 |
| hsa-miR-100* | MI0000102 | CAAGCUUGUAUCUAUAGGUAUG | 1.8285 | 2.004 | 1.3727 | 0.9195 | 0.6018 | 0.4985 |
| hsa-miR-101 | MI0000739 | UACAGUACUGUGAUAACUGAA | 0.3954 | 0.9605 | 0.3719 | 0.5431 | 0.5149 | 0.6982 |
| hsa-miR-101 | MI0000103 | UACAGUACUGUGAUAACUGAA | 0.5774 | 1.0846 | 0.4126 | 0.677 | 0.7024 | 1.1305 |
| hsa-miR-101* | MI0000103 | CAGUUAUCACAGUGCUGAUGCU | 0.2501 | 0.3144 | 0.4198 | 0.2671 | 0.2965 | 0.4782 |
| hsa-miR-103 | MI0000108 | AGCAGCAUUGUACAGGGCUAUGA | 0.972 | 0.6293 | 0.9076 | 0.6285 | 0.874 | 0.66 |
| hsa-miR-103 | MI0000109 | AGCAGCAUUGUACAGGGCUAUGA | 2.8925 | 0.7174 | 1.0467 | 0.6974 | 1.0014 | 1.2269 |
| hsa-miR-105 | MI0000111 | UCAAAUGCUCAGACUCCUGUGGU | 0.8656 | 0.9707 | 0.6051 | 0.7236 | 0.6717 | 0.9232 |
| hsa-miR-105 | MI0000112 | UCAAAUGCUCAGACUCCUGUGGU | 1.3623 | 1.1787 | 0.9536 | 0.8219 | 0.6758 | 0.9258 |
| hsa-miR-105* | MI0000111 | ACGGAUGUUUGAGCAUGUGCUA | 0.7364 | 1.1981 | 0.6126 | 0.7806 | 0.5968 | 0.4998 |
| hsa-miR-105* | MI0000112 | ACGGAUGUUUGAGCAUGUGCUA | 0.7631 | 1.3981 | 0.6588 | 0.8843 | 0.6255 | 0.8018 |
| hsa-miR-106a | MI0000113 | AAAAGUGCUUACAGUGCAGGUAG | 1.1307 | 1.4703 | 1.4018 | 0.8218 | 0.8199 | 0.9467 |
| hsa-miR-106a* | MI0000113 | CUGCAAUGUAAGCACUUCUUAC | 0.4328 | 0.355 | 0.6697 | 0.3835 | 0.6345 | 0.4077 |
| hsa-miR-106b | MI0000734 | UAAAGUGCUGACAGUGCAGAU | 0.484 | 0.9794 | 0.7497 | 0.8059 | 0.9687 | 1.0471 |
| hsa-miR-106b* | MI0000734 | CCGCACUGUGGGUACUUGCUGC | 0.5304 | 0.4437 | 0.5693 | 0.7965 | 0.8004 | 0.5984 |
| hsa-miR-107 | MI0000114 | AGCAGCAUUGUACAGGGCUAUCA | 1.9603 | 0.7123 | 1.2223 | 1.0662 | 0.7562 | 0.6387 |
| hsa-miR-10a | MI0000266 | UACCCUGUAGAUCCGAAUUUGUG | 0.8118 | 2.3465 | 0.7804 | 0.7873 | 1.1689 | 1.5241 |
| hsa-miR-10a* | MI0000266 | CAAAUUCGUAUCUAGGGGAAUA | 1.1055 | 0.9651 | 1.0693 | 0.7419 | 0.4875 | 0.8615 |
| hsa-miR-10b | MI0000267 | UACCCUGUAGAACCGAAUUUGUG | 0.8172 | 0.7858 | 1.9542 | 0.7992 | 1.1974 | 1.2173 |
| hsa-miR-10b* | MI0000267 | ACAGAUUCGAUUCUAGGGGAAU | 0.5249 | 1.3346 | 0.587 | 0.9565 | 0.8391 | 0.5235 |
| hsa-miR-122* | MI0000442 | AACGCCAUUAUCACACUAAAUA | 0.7357 | 1.1534 | 0.5671 | 1.2007 | 0.7827 | 0.7154 |
| hsa-miR-1224-3p | MI0003764 | CCCCACCUCCUCUCUCCUCAG | 1.2276 | 1.2742 | 1.8113 | 0.7728 | 0.8615 | 1.3963 |
| hsa-miR-1224-5p | MI0003764 | GUGAGGACUCGGGAGGUGG | 0.9093 | 0.8682 | 1.9688 | 0.2436 | 1.2003 | 1.465 |
| hsa-miR-1225-3p | MI0006311 | UGAGCCCCUGUGCCGCCCCCAG | 0.9371 | 0.9942 | 1.6662 | 0.6665 | 0.3165 | 0.8796 |
| hsa-miR-1225-5p | MI0006311 | GUGGGUACGGCCCAGUGGGGGG | 0.9519 | 1.225 | 6.1376 | 2.3901 | 1.7905 | 1.3237 |
| hsa-miR-1226 | MI0006313 | UCACCAGCCCUGUGUUCCCUAG | 0.7869 | 0.7049 | 1.5497 | 0.6579 | 1.2952 | 0.9976 |
| hsa-miR-1226* | MI0006313 | GUGAGGGCAUGCAGGCCUGGAUGGGG | 0.8927 | 0.9897 | 1.7317 | 0.5347 | 1.0064 | 1.5367 |
| hsa-miR-1227 | MI0006316 | CGUGCCACCCUUUUCCCCAG | 0.8615 | 0.5673 | 1.3166 | 0.5725 | 0.8254 | 1.1612 |
| hsa-miR-1228 | MI0006318 | UCACACCUGCCUCGCCCCCC | 0.6692 | 0.7618 | 0.9073 | 0.6854 | 0.4359 | 0.6896 |
| hsa-miR-1228* | MI0006318 | GUGGGCGGGGGCAGGUGUGUG | 0.9431 | 0.7393 | 2.0105 | 0.6673 | 0.3521 | 0.8669 |
| hsa-miR-1229 | MI0006319 | CUCUCACCACUGCCCUCCCACAG | 1.4096 | 0.3723 | 0.427 | 1.2924 | 1.7844 | 2.0352 |
| hsa-miR-122a | MI0000442 | UGGAGUGUGACAAUGGUGUUUG | 0.5828 | 2.0569 | 0.6242 | 1.0294 | 0.6929 | 0.5508 |
| hsa-miR-1231 | MI0006321 | GUGUCUGGGCGGACAGCUGC | 1.0974 | 0.3052 | 0.4718 | 1.0179 | 1.2655 | 1.5848 |
| hsa-miR-1233 | MI0006323 | UGAGCCCUGUCCUCCCGCAG | 1.0169 | 0.6688 | 0.3704 | 1.0648 | 0.9671 | 1.5979 |
| hsa-miR-1234 | MI0006324 | UCGGCCUGACCACCCACCCCAC | 0.8745 | 0.5132 | 0.6369 | 1.1174 | 0.6094 | 1.6227 |
| hsa-miR-1236 | MI0006326 | CCUCUUCCCCUUGUCUCUCCAG | 0.7387 | 0.5144 | 0.3994 | 0.9943 | 0.3934 | 0.4196 |
| hsa-miR-1237 | MI0006327 | UCCUUCUGCUCCGUCCCCCAG | 0.7619 | 0.4681 | 0.6538 | 1.041 | 2.217 | 1.12 |
| hsa-miR-1238 | MI0006328 | CUUCCUCGUCUGUCUGCCCC | 0.5921 | 0.399 | 0.5425 | 1.1794 | 1.1468 | 0.606 |
| hsa-miR-124* | MI0000443 | CGUGUUCACAGCGGACCUUGAU | 0.862 | 0.5126 | 0.3932 | 0.4644 | 0.6274 | 0.7668 |
| hsa-miR-124* | MI0000445 | CGUGUUCACAGCGGACCUUGAU | 0.8774 | 1.1241 | 0.4584 | 0.613 | 1.1105 | 0.7792 |
| hsa-miR-124* | MI0000444 | CGUGUUCACAGCGGACCUUGAU | 1.2916 | 1.2267 | 0.7543 | 0.9755 | 2.2494 | 2.5272 |
| hsa-miR-124a | MI0000443 | UAAGGCACGCGGUGAAUGCC | 0.6412 | 2.0184 | 0.6132 | 0.8246 | 0.4599 | 0.6558 |
| hsa-miR-124a | MI0000444 | UAAGGCACGCGGUGAAUGCC | 0.8197 | 2.0706 | 0.6191 | 0.916 | 0.5655 | 0.6633 |
| hsa-miR-124a | MI0000445 | UAAGGCACGCGGUGAAUGCC | 0.8537 | 2.6697 | 0.6517 | 1.1207 | 0.6234 | 1.2942 |
| hsa-miR-125a | MI0000469 | UCCCUGAGACCCUUUAACCUGUGA | 1.2787 | 3.1687 | 0.6902 | 0.8688 | 0.5322 | 0.9918 |
| hsa-miR-125a-3p | MI0000469 | ACAGGUGAGGUUCUUGGGAGCC | 0.9717 | 1.2012 | 0.898 | 1.5722 | 0.6996 | 0.6049 |
| hsa-miR-125b | MI0000446 | UCCCUGAGACCCUAACUUGUGA | 1.0023 | 0.6429 | 1.0353 | 0.6622 | 0.1773 | 0.6765 |
| hsa-miR-125b | MI0000470 | UCCCUGAGACCCUAACUUGUGA | 1.0268 | 0.7128 | -8.6227 | -4.135 | 0.2513 | 3.3876 |
| hsa-miR-125b-1* | MI0000446 | ACGGGUUAGGCUCUUGGGAGCU | 0.7805 | 0.6154 | 0.6188 | 1.3138 | 0.6031 | 0.934 |
| hsa-miR-125b-2* | MI0000470 | UCACAAGUCAGGCUCUUGGGAC | -26.6893 | -17.6874 | 0.8406 | 0.7111 | 3.2518 | 3.0511 |
| hsa-miR-126 | MI0000471 | UCGUACCGUGAGUAAUAAUGCG | 0.5731 | 0.3415 | 0.5045 | 0.5859 | 0.7979 | 0.6012 |
| hsa-miR-126* | MI0000471 | CAUUAUUACUUUUGGUACGCG | 0.4321 | 0.6817 | 0.9331 | 1.0618 | 0.5289 | 0.9711 |
| hsa-miR-127 | MI0000472 | UCGGAUCCGUCUGAGCUUGGCU | 0.9406 | 1.1635 | 0.8386 | 1.2788 | 0.7872 | 1.1651 |
| hsa-miR-127-5p | MI0000472 | CUGAAGCUCAGAGGGCUCUGAU | 0.301 | 0.786 | 0.4696 | 1.2564 | 1.4635 | 0.8859 |
| hsa-miR-128 | MI0000727 | UCACAGUGAACCGGUCUCUUU | 1.6585 | 0.3849 | 0.2803 | 0.2718 | 1.8234 | 2.0956 |
| hsa-miR-128a | MI0000447 | UCACAGUGAACCGGUCUCUUU | 1.0252 | 0.7947 | 1.3339 | 0.7498 | 0.579 | 0.474 |
| hsa-miR-129 | MI0000473 | CUUUUUGCGGUCUGGGCUUGC | 0.998 | 0.9396 | 0.6173 | 0.6749 | 0.0288 | 0.5249 |
| hsa-miR-129 | MI0000252 | CUUUUUGCGGUCUGGGCUUGC | 1.5223 | 1.88 | 0.8264 | 0.9903 | 1.345 | 1.5943 |
| hsa-miR-129-3p | MI0000252 | AAGCCCUUACCCCAAAAAGUAU | 0.7052 | 1.0168 | 0.6151 | 0.5787 | 2.3527 | 1.9729 |
| hsa-miR-129* | MI0000473 | AAGCCCUUACCCCAAAAAGCAU | 0.9704 | 1.8602 | 0.7779 | 0.8582 | 0.6443 | 0.5966 |
| hsa-miR-130a | MI0000448 | CAGUGCAAUGUUAAAAGGGCAU | 0.2074 | 0.262 | 0.5281 | 0.8402 | 0.8756 | 1.7301 |
| hsa-miR-130a* | MI0000448 | UUCACAUUGUGCUACUGUCUGC | 0.735 | 1.002 | 0.6018 | 1.0706 | 0.8947 | 0.7551 |
| hsa-miR-130b | MI0000748 | CAGUGCAAUGAUGAAAGGGCAU | 0.5932 | 0.6021 | -11.3912 | 3.4506 | 1.7075 | 1.9619 |
| hsa-miR-130b* | MI0000748 | ACUCUUUCCCUGUUGCACUAC | 0.4409 | 1.6398 | 0.5955 | 0.7935 | 0.548 | 0.5069 |
| hsa-miR-132 | MI0000449 | UAACAGUCUACAGCCAUGGUCG | 0.5882 | 0.6321 | 0.4023 | 0.6034 | 0.6115 | 0.8116 |
| hsa-miR-132* | MI0000449 | ACCGUGGCUUUCGAUUGUUACU | 0.7599 | 1.2034 | 0.5713 | 1.2041 | 0.5104 | 1.0101 |
| hsa-miR-133a | MI0000450 | UUUGGUCCCCUUCAACCAGCUG | 0.7671 | 0.8116 | 0.5933 | 0.8101 | 0.4475 | 0.445 |
| hsa-miR-133a | MI0000451 | UUUGGUCCCCUUCAACCAGCUG | 0.8512 | 0.9992 | 0.6512 | 0.9414 | 0.7377 | 0.8263 |
| hsa-miR-133b | MI0000822 | UUUGGUCCCCUUCAACCAGCUA | 0.8456 | 0.9958 | 0.6979 | 1.0926 | 0.2398 | 0.4305 |
| hsa-miR-134 | MI0000474 | UGUGACUGGUUGACCAGAGGGG | 1.3437 | 3.266 | 0.7476 | 1.3791 | 0.6218 | 0.5692 |
| hsa-miR-135a | MI0000452 | UAUGGCUUUUUAUUCCUAUGUGA | 0.4681 | 0.5844 | 0.4136 | 0.3696 | 0.8224 | 1.54 |
| hsa-miR-135a | MI0000453 | UAUGGCUUUUUAUUCCUAUGUGA | 0.508 | 0.6436 | 0.6308 | 0.7404 | 1.5822 | 1.8875 |
| hsa-miR-135a* | MI0000452 | UAUAGGGAUUGGAGCCGUGGCG | 0.8409 | 0.752 | 0.2443 | 0.315 | 0.9533 | 0.7127 |
| hsa-miR-135b | MI0000810 | UAUGGCUUUUCAUUCCUAUGUGA | 2.0563 | 1.6662 | 0.9015 | 0.6142 | 0.3936 | 0.8604 |
| hsa-miR-135b* | MI0000810 | AUGUAGGGCUAAAAGCCAUGGG | 0.7131 | 2.2217 | 0.6184 | 1.0924 | 0.796 | 0.472 |
| hsa-miR-136 | MI0000475 | ACUCCAUUUGUUUUGAUGAUGGA | 0.6379 | 1.404 | 0.6418 | 1.034 | 1.1403 | 1.2865 |
| hsa-miR-136* | MI0000475 | CAUCAUCGUCUCAAAUGAGUCU | 0.6675 | 0.8527 | 0.8978 | 1.1832 | 0.4343 | 0.4113 |
| hsa-miR-137 | MI0000454 | UUAUUGCUUAAGAAUACGCGUAG | 1.5528 | 1.8241 | 1.4113 | 0.5763 | 0.5495 | 0.8456 |
| hsa-miR-138 | MI0000455 | AGCUGGUGUUGUGAAUCAGGCCG | 0.5613 | 0.5256 | 0.4596 | 0.5773 | 0.2211 | 0.5293 |
| hsa-miR-138 | MI0000476 | AGCUGGUGUUGUGAAUCAGGCCG | 0.7828 | 1.0197 | 0.5385 | 0.8338 | 0.6233 | 1.1958 |
| hsa-miR-138-1* | MI0000476 | GCUACUUCACAACACCAGGGCC | 0.9144 | 1.1529 | 0.8294 | 1.687 | 1.0001 | 1.2118 |
| hsa-miR-138-2* | MI0000455 | GCUAUUUCACGACACCAGGGUU | 0.5751 | 1.2135 | 0.4462 | 1.3792 | 0.7394 | 0.7758 |
| hsa-miR-139 | MI0000261 | UCUACAGUGCACGUGUCUCCAG | 0.4258 | 0.7257 | 0.6437 | 0.6086 | 1.0749 | 1.0152 |
| hsa-miR-139-3p | MI0000261 | GGAGACGCGGCCCUGUUGGAGU | 1.1409 | 1.1547 | 0.9402 | 1.1156 | 0.623 | 1.1818 |
| hsa-miR-140 | MI0000456 | CAGUGGUUUUACCCUAUGGUAG | 0.6219 | 0.6929 | 0.582 | 0.7911 | 0.5461 | 0.6918 |
| hsa-miR-140-3p | MI0000456 | UACCACAGGGUAGAACCACGG | 0.7253 | 1.0643 | 0.5629 | 1.9112 | 0.9514 | 0.8773 |
| hsa-miR-141 | MI0000457 | UAACACUGUCUGGUAAAGAUGG | 1.0845 | 0.96 | 1.1501 | 0.7462 | 0.9308 | 1.4163 |
| hsa-miR-141* | MI0000457 | CAUCUUCCAGUACAGUGUUGGA | 0.848 | 0.8718 | 0.6751 | 1.3817 | 0.8284 | 0.9287 |
| hsa-miR-142-3p | MI0000458 | UGUAGUGUUUCCUACUUUAUGGA | 0.4355 | 0.6032 | -2.6086 | 0.36 | 0.8426 | 0.8992 |
| hsa-miR-142-5p | MI0000458 | CAUAAAGUAGAAAGCACUACU | 0.6752 | 0.9177 | 0.6493 | 0.8342 | 0.4521 | 0.6705 |
| hsa-miR-143 | MI0000459 | UGAGAUGAAGCACUGUAGCUC | 0.3995 | 0.8611 | 0.467 | 0.5559 | 0.3525 | 0.4132 |
| hsa-miR-143* | MI0000459 | GGUGCAGUGCUGCAUCUCUGGU | 0.7311 | 0.7259 | 0.6377 | 2.3702 | 0.8211 | 0.7534 |
| hsa-miR-144 | MI0000460 | UACAGUAUAGAUGAUGUACU | 0.6228 | 0.9352 | 0.5695 | 0.7809 | 0.5253 | 0.533 |
| hsa-miR-144* | MI0000460 | GGAUAUCAUCAUAUACUGUAAG | 0.8149 | 0.7408 | 0.5221 | -5.4639 | 1.0909 | 0.9648 |
| hsa-miR-145 | MI0000461 | GUCCAGUUUUCCCAGGAAUCCCU | 0.7681 | 1.6561 | 0.636 | 0.8824 | 0.7242 | 0.8233 |
| hsa-miR-145* | MI0000461 | GGAUUCCUGGAAAUACUGUUCU | 0.7301 | 1.3222 | 0.9418 | 1.2749 | 4.1613 | 1.873 |
| hsa-miR-146a | MI0000477 | UGAGAACUGAAUUCCAUGGGUU | 0.3718 | 1.3422 | 0.1946 | 0.5247 | 0.2539 | 1.3266 |
| hsa-miR-146a* | MI0000477 | CCUCUGAAAUUCAGUUCUUCAG | 0.8323 | 0.6664 | 0.8627 | 0.9817 | 1.1121 | 0.6244 |
| hsa-miR-146b | MI0003129 | UGAGAACUGAAUUCCAUAGGCU | 0.7866 | 0.9298 | 1.0244 | 0.7082 | 1.0763 | 1.6284 |
| hsa-miR-146b-3p | MI0003129 | UGCCCUGUGGACUCAGUUCUGG | 0.84 | 0.8185 | 0.6329 | 0.8384 | 0.57 | 0.8738 |
| hsa-miR-147 | MI0000262 | GUGUGUGGAAAUGCUUCUGC | 0.3928 | 0.4888 | 0.3971 | 0.7406 | 0.518 | 1.7628 |
| hsa-miR-147b | MI0005544 | GUGUGCGGAAAUGCUUCUGCUA | 0.6309 | 1.5605 | 0.4177 | 0.8151 | 0.5538 | 0.4269 |
| hsa-miR-148a | MI0000253 | UCAGUGCACUACAGAACUUUGU | 0.186 | 0.1939 | 7.4271 | 4.5916 | 1.4803 | 1.0194 |
| hsa-miR-148a* | MI0000253 | AAAGUUCUGAGACACUCCGACU | 0.5001 | 0.9106 | 0.5025 | 0.8677 | 0.9534 | 0.8361 |
| hsa-miR-148b | MI0000811 | UCAGUGCAUCACAGAACUUUGU | 0.3872 | 2.3534 | 0.524 | 0.9011 | 0.3352 | 0.638 |
| hsa-miR-148b* | MI0000811 | AAGUUCUGUUAUACACUCAGGC | 0.7675 | 0.8668 | 0.3432 | 1.0249 | -0.0795 | 1.5527 |
| hsa-miR-149 | MI0000478 | UCUGGCUCCGUGUCUUCACUCCC | 1.35 | 0.7299 | 0.4856 | 0.7248 | 0.43 | 0.86 |
| hsa-miR-149* | MI0000478 | AGGGAGGGACGGGGGCUGUGC | 0.4509 | 0.309 | 0.3263 | 0.6505 | 0.4012 | 0.4767 |
| hsa-miR-150 | MI0000479 | UCUCCCAACCCUUGUACCAGUG | 0.678 | 1.9986 | 0.2503 | 0.7258 | 0.4843 | 1.3084 |
| hsa-miR-150* | MI0000479 | CUGGUACAGGCCUGGGGGACAG | 0.8472 | 1.1578 | 0.9029 | 1.8519 | 1.257 | 0.5918 |
| hsa-miR-151 | MI0000809 | CUAGACUGAAGCUCCUUGAGG | 0.882 | 1.1123 | 0.714 | 1.1978 | 0.5251 | 0.7969 |
| hsa-miR-151-5p | MI0000809 | UCGAGGAGCUCACAGUCUAGU | 0.5795 | 0.9429 | 0.613 | 1.2173 | 3.6057 | 5.3895 |
| hsa-miR-152 | MI0000462 | UCAGUGCAUGACAGAACUUGG | 0.3738 | 1.0569 | 0.5426 | 0.6005 | 0.3686 | 0.4705 |
| hsa-miR-153 | MI0000463 | UUGCAUAGUCACAAAAGUGAUC | 0.9949 | 1.2156 | 3.1165 | 3.056 | 0.7761 | 0.8187 |
| hsa-miR-153 | MI0000464 | UUGCAUAGUCACAAAAGUGAUC | 1.3826 | 1.25 | -19.2022 | -3.056 | 1.0516 | 0.8812 |
| hsa-miR-154 | MI0000480 | UAGGUUAUCCGUGUUGCCUUCG | 0.9328 | 1.0996 | 0.8587 | 0.9003 | 0.6267 | 0.9395 |
| hsa-miR-154* | MI0000480 | AAUCAUACACGGUUGACCUAUU | 0.9597 | 1.9334 | 0.3469 | 1.1064 | 0.848 | 1.231 |
| hsa-miR-155 | MI0000681 | UUAAUGCUAAUCGUGAUAGGGGU | 0.7287 | 1.414 | 0.7569 | 0.8915 | 0.3983 | 0.5535 |
| hsa-miR-155* | MI0000681 | CUCCUACAUAUUAGCAUUAACA | 1.4323 | 1.2789 | 0.7737 | 0.8824 | 0.8052 | 0.5035 |
| hsa-miR-15a | MI0000069 | UAGCAGCACAUAAUGGUUUGUG | 1.8741 | 0.9124 | 1.1925 | 1.1588 | 0.5124 | 0.2511 |
| hsa-miR-15a* | MI0000069 | CAGGCCAUAUUGUGCUGCCUCA | 1.1506 | 1.1686 | 0.7592 | 0.9803 | 0.9011 | 1.0678 |
| hsa-miR-15b | MI0000438 | UAGCAGCACAUCAUGGUUUACA | 0.2023 | 0.3785 | 1.0893 | 1.3698 | 0.9015 | 1.5081 |
| hsa-miR-15b* | MI0000438 | CGAAUCAUUAUUUGCUGCUCUA | 0.4292 | 0.7108 | 0.5718 | 0.786 | 0.4614 | 0.7797 |
| hsa-miR-16 | MI0000070 | UAGCAGCACGUAAAUAUUGGCG | 0.8265 | 1.1036 | 0.709 | 0.6173 | 0.3893 | 0.8544 |
| hsa-miR-16 | MI0000115 | UAGCAGCACGUAAAUAUUGGCG | 1.2843 | 1.5911 | 0.8293 | 0.7465 | 0.6372 | 1.23 |
| hsa-miR-16-1* | MI0000070 | CCAGUAUUAACUGUGCUGCUGA | 0.8669 | 0.87 | 0.7274 | 0.7627 | 0.6505 | 0.7847 |
| hsa-miR-16-2* | MI0000115 | CCAAUAUUACUGUGCUGCUUUA | -3.8991 | -10.1282 | 0.611 | 1.0638 | 0.8525 | 0.7149 |
| hsa-miR-17-3p | MI0000071 | ACUGCAGUGAAGGCACUUGUAG | 0.6941 | -18.3877 | 0.3457 | 0.2852 | 0.8819 | 3.8064 |
| hsa-miR-17-5p | MI0000071 | CAAAGUGCUUACAGUGCAGGUAG | 1.1114 | 1.9157 | 0.8651 | 0.8572 | 0.5832 | 0.7879 |
| hsa-miR-181a | MI0000269 | AACAUUCAACGCUGUCGGUGAGU | -4.3359 | -3.7601 | 0.598 | 0.4996 | 2.5936 | 1.7417 |
| hsa-miR-181a | MI0000289 | AACAUUCAACGCUGUCGGUGAGU | 0.1738 | 0.2131 | 0.6146 | 0.5388 | 2.8864 | 1.7998 |
| hsa-miR-181a-2* | MI0000269 | ACCACUGACCGUUGACUGUACC | 0.81 | 1.6124 | 0.539 | 0.7897 | 0.5774 | 0.3404 |
| hsa-miR-181b | MI0000270 | AACAUUCAUUGCUGUCGGUGGGU | 0.7175 | 0.6984 | 0.6512 | 0.4101 | 0.7387 | 0.6046 |
| hsa-miR-181b | MI0000683 | AACAUUCAUUGCUGUCGGUGGGU | 0.9256 | 0.8246 | 1.0098 | 0.5067 | 0.7549 | 0.6616 |
| hsa-miR-181c | MI0000271 | AACAUUCAACCUGUCGGUGAGU | 0.4262 | 0.4004 | 0.7747 | 0.7256 | 0.2666 | 0.3897 |
| hsa-miR-181c* | MI0000271 | AACCAUCGACCGUUGAGUGGAC | 0.5791 | 1.1166 | 0.8717 | 1.0143 | 0.7206 | 0.795 |
| hsa-miR-181d | MI0003139 | AACAUUCAUUGUUGUCGGUGGGU | 0.3341 | 0.1137 | 0.495 | 0.5782 | 0.453 | 0.43 |
| hsa-miR-182 | MI0000272 | UUUGGCAAUGGUAGAACUCACACU | 0.7441 | 1.0751 | 1.8426 | 0.659 | 0.8147 | 0.7625 |
| hsa-miR-182* | MI0000272 | UGGUUCUAGACUUGCCAACUA | 0.5707 | 0.6304 | 0.8195 | 0.8273 | 0.8063 | 1.113 |
| hsa-miR-183 | MI0000273 | UAUGGCACUGGUAGAAUUCACU | 1.1612 | 1.9219 | 1.447 | 0.8269 | 0.5692 | 1.1124 |
| hsa-miR-183* | MI0000273 | GUGAAUUACCGAAGGGCCAUAA | 0.7133 | 1.6697 | 0.4786 | 0.8717 | 0.8701 | 0.8414 |
| hsa-miR-184 | MI0000481 | UGGACGGAGAACUGAUAAGGGU | 1.3719 | 1.2967 | 0.7441 | 1.3033 | 0.8228 | 1.1721 |
| hsa-miR-185 | MI0000482 | UGGAGAGAAAGGCAGUUCCUGA | 0.5283 | 1.2538 | 0.4797 | 0.5891 | 0.3568 | 0.6219 |
| hsa-miR-185* | MI0000482 | AGGGGCUGGCUUUCCUCUGGUC | 0.3066 | 1.1659 | 0.595 | 0.9866 | 0.82 | 1.0824 |
| hsa-miR-186 | MI0000483 | CAAAGAAUUCUCCUUUUGGGCU | 1.1788 | 1.2886 | 1.9094 | 0.908 | 0.4275 | 0.7954 |
| hsa-miR-186* | MI0000483 | GCCCAAAGGUGAAUUUUUUGGG | 0.6062 | 1.1434 | 0.6432 | 0.8373 | 0.6223 | 0.6735 |
| hsa-miR-187 | MI0000274 | UCGUGUCUUGUGUUGCAGCCGG | 0.6273 | 0.8596 | 0.6165 | 0.9947 | 0.9574 | 0.8413 |
| hsa-miR-187* | MI0000274 | GGCUACAACACAGGACCCGGGC | 1.6163 | 2.3282 | 0.9064 | 1.0262 | 2.5686 | 1.5665 |
| hsa-miR-188 | MI0000484 | CAUCCCUUGCAUGGUGGAGGG | 1.2362 | 1.8714 | 0.705 | 0.7767 | 0.5994 | 0.7344 |
| hsa-miR-188-3p | MI0000484 | CUCCCACAUGCAGGGUUUGCA | 0.4502 | 0.9453 | 0.6121 | 0.6475 | 0.6323 | 0.5114 |
| hsa-miR-189 | MI0000080 | UGCCUACUGAGCUGAUAUCAGU | 1.1731 | 1.4833 | 0.5486 | 0.8513 | 0.4358 | 1.1388 |
| hsa-miR-18a | MI0000072 | UAAGGUGCAUCUAGUGCAGAUAG | 2.135 | 2.5723 | 0.5564 | 0.7088 | 0.3848 | 0.6909 |
| hsa-miR-18a* | MI0000072 | ACUGCCCUAAGUGCUCCUUCUGG | 0.8888 | 1.1789 | 0.8151 | 1.0327 | 1.7434 | 1.354 |
| hsa-miR-18b | MI0001518 | UAAGGUGCAUCUAGUGCAGUUAG | 0.6685 | 0.7512 | 2.5439 | 1.9318 | 0.4541 | 0.6532 |
| hsa-miR-18b* | MI0001518 | UGCCCUAAAUGCCCCUUCUGGC | 0.5868 | 0.8263 | 0.5573 | 0.7782 | 0.6065 | 0.5829 |
| hsa-miR-190 | MI0000486 | UGAUAUGUUUGAUAUAUUAGGU | 0.1973 | 0.237 | 0.2256 | 0.6664 | 0.5693 | 1.0324 |
| hsa-miR-190b | MI0005545 | UGAUAUGUUUGAUAUUGGGUU | 0.3619 | 0.2946 | 0.6041 | 0.9977 | 0.3618 | 0.4515 |
| hsa-miR-191 | MI0000465 | CAACGGAAUCCCAAAAGCAGCUG | 0.6933 | 1.2709 | 0.5651 | 0.965 | 0.8147 | 0.7985 |
| hsa-miR-191* | MI0000465 | GCUGCGCUUGGAUUUCGUCCCC | 0.6369 | 0.4203 | 0.9138 | 0.7393 | 0.4507 | 1.3776 |
| hsa-miR-192 | MI0000234 | CUGACCUAUGAAUUGACAGCC | 1.0515 | 0.8799 | 0.1682 | 0.2841 | 0.612 | 0.7908 |
| hsa-miR-192* | MI0000234 | CUGCCAAUUCCAUAGGUCACAG | 1.0017 | 0.966 | 1.5194 | 0.8082 | 0.3257 | 0.5403 |
| hsa-miR-193a | MI0000487 | AACUGGCCUACAAAGUCCCAGU | 0.5085 | 1.865 | 0.5265 | 1.6862 | 0.4642 | 1.2804 |
| hsa-miR-193a-5p | MI0000487 | UGGGUCUUUGCGGGCGAGAUGA | 0.8394 | 1.0417 | 0.8161 | 1.3525 | 0.6526 | 0.8757 |
| hsa-miR-193b | MI0003137 | AACUGGCCCUCAAAGUCCCGCU | 0.9033 | 1.3264 | 0.9249 | 0.8279 | 0.5508 | 2.2889 |
| hsa-miR-193b* | MI0003137 | CGGGGUUUUGAGGGCGAGAUGA | 0.8874 | 0.3852 | 0.3587 | 0.1821 | 0.5483 | 1.3478 |
| hsa-miR-194 | MI0000732 | UGUAACAGCAACUCCAUGUGGA | 0.6445 | 0.966 | 0.6099 | 0.8285 | 0.9833 | 1.3149 |
| hsa-miR-194 | MI0000488 | UGUAACAGCAACUCCAUGUGGA | 1.1227 | 1.041 | 0.9106 | 1.0196 | 1.5888 | 1.648 |
| hsa-miR-194* | MI0000732 | CCAGUGGGGCUGCUGUUAUCUG | 1.4346 | 1.3042 | 0.6372 | 1.1344 | 1.5244 | 1.7703 |
| hsa-miR-195 | MI0000489 | UAGCAGCACAGAAAUAUUGGC | 1.1744 | 1.1009 | 0.7488 | 0.8539 | 1.106 | 1.5986 |
| hsa-miR-195* | MI0000489 | CCAAUAUUGGCUGUGCUGCUCC | 0.9082 | 0.9788 | 0.788 | 1.0187 | 1.3974 | 1.4042 |
| hsa-miR-196a | MI0000238 | UAGGUAGUUUCAUGUUGUUGGG | 0.6846 | 1.1513 | 0.6388 | 0.7483 | 0.49 | 0.5064 |
| hsa-miR-196a | MI0000279 | UAGGUAGUUUCAUGUUGUUGGG | 1.0108 | 4.3903 | 1.8739 | 1.2387 | 0.5919 | 0.6882 |
| hsa-miR-196a* | MI0000279 | CGGCAACAAGAAACUGCCUGAG | 1.7878 | 3.512 | 1.12 | 1.49 | 1.4153 | 1.8162 |
| hsa-miR-196b | MI0001150 | UAGGUAGUUUCCUGUUGUUGGG | 0.9932 | 1.3291 | 1.4782 | 0.7892 | 0.6133 | 0.7977 |
| hsa-miR-197 | MI0000239 | UUCACCACCUUCUCCACCCAGC | 0.5951 | 0.7699 | 0.7918 | 0.7335 | 0.9947 | 1.3836 |
| hsa-miR-198 | MI0000240 | GGUCCAGAGGGGAGAUAGGUUC | 0.7614 | 1.4573 | 0.7352 | 0.7304 | 0.8224 | 1.2791 |
| hsa-miR-199a | MI0000242 | CCCAGUGUUCAGACUACCUGUUC | 0.9146 | 0.8516 | 1.1555 | 1.1041 | 0.7862 | 0.9014 |
| hsa-miR-199a | MI0000281 | CCCAGUGUUCAGACUACCUGUUC | 1.742 | 0.8796 | 7.2636 | 2.1956 | 1.3583 | 1.4413 |
| hsa-miR-199a* | MI0000242 | ACAGUAGUCUGCACAUUGGUUA | 0.618 | 0.9685 | 0.6097 | 0.5157 | 0.7199 | 0.4476 |
| hsa-miR-199a* | MI0000281 | ACAGUAGUCUGCACAUUGGUUA | 1.3002 | 1.034 | 1.522 | 0.6168 | 1.1503 | 0.8659 |
| hsa-miR-199b | MI0000282 | CCCAGUGUUUAGACUAUCUGUUC | 0.5507 | 0.5244 | 0.7519 | 0.5924 | 2.633 | 1.7175 |
| hsa-miR-199b-3p | MI0000282 | ACAGUAGUCUGCACAUUGGUUA | 0.3965 | 1.0995 | 0.4844 | 0.6832 | 0.7111 | 0.3221 |
| hsa-miR-19a | MI0000073 | UGUGCAAAUCUAUGCAAAACUGA | 1.5958 | 0.7475 | -13.8056 | -7.706 | 0.5942 | 0.5601 |
| hsa-miR-19a* | MI0000073 | AGUUUUGCAUAGUUGCACUACA | 1.0818 | 1.1959 | 0.1162 | 0.9253 | 0.6546 | 0.743 |
| hsa-miR-19b | MI0000074 | UGUGCAAAUCCAUGCAAAACUGA | 0.4176 | 0.5047 | 0.5329 | 0.4619 | 0.0101 | 0.3569 |
| hsa-miR-19b | MI0000075 | UGUGCAAAUCCAUGCAAAACUGA | 0.7958 | 0.5741 | 0.6734 | 0.7612 | 0.4743 | 0.4901 |
| hsa-miR-19b-1* | MI0000074 | AGUUUUGCAGGUUUGCAUCCAGC | 0.9044 | 1.1928 | 0.7742 | 0.9985 | 0.8019 | 0.7765 |
| hsa-miR-19b-2* | MI0000075 | AGUUUUGCAGGUUUGCAUUUCA | 1.1766 | 1.7105 | 0.7952 | 1.8504 | 0.841 | 1.3592 |
| hsa-miR-200a | MI0000737 | UAACACUGUCUGGUAACGAUGU | 0.4762 | 0.5131 | 0.5218 | 0.6052 | 0.4426 | 0.217 |
| hsa-miR-200a* | MI0000737 | CAUCUUACCGGACAGUGCUGGA | 0.5596 | 0.8215 | 0.9825 | 1.0733 | 0.4553 | 0.3128 |
| hsa-miR-200b | MI0000342 | UAAUACUGCCUGGUAAUGAUGA | 1.0114 | 1.3012 | 0.6281 | 0.8438 | 0.8471 | 0.9304 |
| hsa-miR-200b* | MI0000342 | CAUCUUACUGGGCAGCAUUGGA | 1.1009 | 0.4585 | 0.7001 | 0.8076 | 0.9509 | 0.6816 |
| hsa-miR-200c | MI0000650 | UAAUACUGCCGGGUAAUGAUGGA | 1.1508 | 2.1054 | 0.6831 | 0.9884 | 0.4679 | 0.5485 |
| hsa-miR-200c* | MI0000650 | CGUCUUACCCAGCAGUGUUUGG | 0.7938 | 1.171 | 1.0628 | 3.4472 | 3.1666 | -0.2631 |
| hsa-miR-202 | MI0003130 | AGAGGUAUAGGGCAUGGGAA | 1.008 | 1.3799 | 0.6416 | 0.7375 | 0.6523 | 1.0914 |
| hsa-miR-202* | MI0003130 | UUCCUAUGCAUAUACUUCUUUG | 0.6497 | 0.7409 | 0.6426 | 0.714 | 1.1822 | 0.9846 |
| hsa-miR-203 | MI0000283 | GUGAAAUGUUUAGGACCACUAG | 0.4693 | 1.1114 | 0.6986 | 0.8363 | 0.6544 | 1.1726 |
| hsa-miR-204 | MI0000284 | UUCCCUUUGUCAUCCUAUGCCU | 0.3386 | 0.6817 | 0.9687 | 1.0451 | 0.4442 | 1.641 |
| hsa-miR-205 | MI0000285 | UCCUUCAUUCCACCGGAGUCUG | 0.5145 | 0.5835 | 0.4949 | 0.6598 | 0.3874 | 0.5544 |
| hsa-miR-206 | MI0000490 | UGGAAUGUAAGGAAGUGUGUGG | 0.8741 | 0.91 | 0.8691 | 1.0379 | 0.8978 | 1.3038 |
| hsa-miR-208 | MI0000251 | AUAAGACGAGCAAAAAGCUUGU | 1.2938 | 1.4872 | 1.2189 | 0.9484 | 1.207 | 1.7989 |
| hsa-miR-208b | MI0005570 | AUAAGACGAACAAAAGGUUUGU | 1.0261 | 2.2686 | 1.6828 | 0.7573 | 0.8024 | 1.0273 |
| hsa-miR-20a | MI0000076 | UAAAGUGCUUAUAGUGCAGGUAG | 1.5134 | 0.9407 | 0.8024 | 0.6891 | 0.1968 | 2.1882 |
| hsa-miR-20a* | MI0000076 | ACUGCAUUAUGAGCACUUAAAG | 0.6827 | 0.7699 | 1.1802 | 1.2161 | 1.0673 | 1.3582 |
| hsa-miR-20b | MI0001519 | CAAAGUGCUCAUAGUGCAGGUAG | 0.7464 | 0.9059 | 0.7777 | 0.6933 | 1.2571 | 1.221 |
| hsa-miR-20b* | MI0001519 | ACUGUAGUAUGGGCACUUCCAG | 0.615 | 1.021 | 0.5921 | 0.6973 | 0.993 | 0.7818 |
| hsa-miR-21 | MI0000077 | UAGCUUAUCAGACUGAUGUUGA | 0.9298 | 0.4205 | 1.1188 | 0.7345 | 1.1926 | 0.7218 |
| hsa-miR-21* | MI0000077 | CAACACCAGUCGAUGGGCUGU | 0.4736 | 0.5942 | 0.6551 | 0.6733 | 0.6995 | 0.6112 |
| hsa-miR-210 | MI0000286 | CUGUGCGUGUGACAGCGGCUGA | 1.1491 | 0.3325 | 0.6448 | 0.5491 | 0.2264 | 0.2714 |
| hsa-miR-211 | MI0000287 | UUCCCUUUGUCAUCCUUCGCCU | 0.5291 | 1.3708 | -9.8297 | -6.1667 | 0.674 | 1.4286 |
| hsa-miR-212 | MI0000288 | UAACAGUCUCCAGUCACGGCC | 1.3833 | 0.8032 | 0.9771 | 0.7395 | 0.7411 | 0.9971 |
| hsa-miR-213 | MI0000289 | ACCAUCGACCGUUGAUUGUACC | 2.2899 | 3.7309 | 0.7498 | 0.6546 | 2.0295 | 2.1761 |
| hsa-miR-214 | MI0000290 | ACAGCAGGCACAGACAGGCAGU | 0.7731 | 0.9276 | 0.5094 | 0.5686 | 0.5523 | 0.6883 |
| hsa-miR-214* | MI0000290 | UGCCUGUCUACACUUGCUGUGC | 0.6604 | 1.3473 | 0.5995 | 1.5176 | 0.5847 | 1.3376 |
| hsa-miR-215 | MI0000291 | AUGACCUAUGAAUUGACAGAC | 0.4912 | 0.6023 | 1.0321 | 0.9743 | 0.4 | 2.5489 |
| hsa-miR-216 | MI0000292 | UAAUCUCAGCUGGCAACUGUGA | 1.2108 | 1.6611 | 1.4302 | 0.7632 | 0.851 | 1.0352 |
| hsa-miR-216b | MI0005569 | AAAUCUCUGCAGGCAAAUGUGA | 0.6197 | 1.5586 | 1.9097 | 0.7825 | 0.6985 | 0.7413 |
| hsa-miR-217 | MI0000293 | UACUGCAUCAGGAACUGAUUGGA | 0.8059 | 0.7598 | 0.5225 | 0.7075 | 0.6568 | 0.5797 |
| hsa-miR-218 | MI0000295 | UUGUGCUUGAUCUAACCAUGU | 0.2504 | 0.3089 | 0.9526 | 0.6641 | 0.1102 | 0.3895 |
| hsa-miR-218 | MI0000294 | UUGUGCUUGAUCUAACCAUGU | 0.2582 | 0.7527 | 1.2043 | 0.7992 | 0.3461 | 0.7713 |
| hsa-miR-218-1* | MI0000294 | AUGGUUCCGUCAAGCACCAUGG | 0.4828 | 1.1028 | 0.4748 | 1.344 | 0.6159 | 1.1628 |
| hsa-miR-218-2* | MI0000295 | CAUGGUUCUGUCAAGCACCGCG | 0.9277 | 2.2758 | 0.5344 | 1.3159 | 1.0485 | 0.8844 |
| hsa-miR-219 | MI0000740 | UGAUUGUCCAAACGCAAUUCU | 0.5548 | 0.8454 | 0.8348 | 0.9676 | 0.4843 | 0.5316 |
| hsa-miR-219 | MI0000296 | UGAUUGUCCAAACGCAAUUCU | 0.727 | 0.8489 | 1.3073 | 1.4083 | 1.4922 | 1.761 |
| hsa-miR-219-1-3p | MI0000296 | AGAGUUGAGUCUGGACGUCCCG | 1.0593 | 1.1402 | 0.6377 | 0.9022 | 0.6915 | 0.6085 |
| hsa-miR-219-2-3p | MI0000740 | AGAAUUGUGGCUGGACAUCUGU | 0.6712 | 0.8009 | 0.6248 | 0.8703 | 0.5825 | 0.5427 |
| hsa-miR-22 | MI0000078 | AAGCUGCCAGUUGAAGAACUGU | 0.7133 | 0.6957 | 0.7854 | 0.7819 | -0.0088 | 0.7822 |
| hsa-miR-22* | MI0000078 | AGUUCUUCAGUGGCAAGCUUUA | 0.4412 | 0.6265 | 0.8746 | 1.0119 | 0.6611 | 0.3854 |
| hsa-miR-220 | MI0000297 | CCACACCGUAUCUGACACUUU | 0.7507 | 1.0109 | 0.7229 | 0.8403 | 0.4443 | 0.9488 |
| hsa-miR-220b | MI0005529 | CCACCACCGUGUCUGACACUU | 0.764 | 1.076 | 0.649 | 0.8774 | 0.6177 | 0.6639 |
| hsa-miR-220c | MI0005536 | ACACAGGGCUGUUGUGAAGACU | 0.6834 | 1.5079 | 0.8444 | 1.3768 | 0.5384 | 0.3094 |
| hsa-miR-221 | MI0000298 | AGCUACAUUGUCUGCUGGGUUUC | 0.6662 | 0.378 | 1.0379 | 0.4288 | 8.3032 | 1.715 |
| hsa-miR-221* | MI0000298 | ACCUGGCAUACAAUGUAGAUUU | 0.4704 | 1.0325 | 0.7024 | 1.548 | 0.79 | 1.6444 |
| hsa-miR-222 | MI0000299 | AGCUACAUCUGGCUACUGGGU | 1.0577 | 0.5334 | 1.1777 | 0.4032 | 0.6221 | 0.4161 |
| hsa-miR-222* | MI0000299 | CUCAGUAGCCAGUGUAGAUCCU | 0.5695 | 0.6674 | 0.4835 | 0.6235 | 0.5553 | 0.2687 |
| hsa-miR-223 | MI0000300 | UGUCAGUUUGUCAAAUACCCCA | 1.2208 | 1.4189 | 2.9205 | 0.7814 | 0.6338 | 0.7946 |
| hsa-miR-223* | MI0000300 | CGUGUAUUUGACAAGCUGAGUU | 0.5589 | 1.2914 | 0.5685 | 1.334 | 0.5025 | 0.6732 |
| hsa-miR-224 | MI0000301 | CAAGUCACUAGUGGUUCCGUU | 0.675 | 0.8022 | 1.2489 | 0.5165 | 0.7182 | 0.7766 |
| hsa-miR-23a | MI0000079 | AUCACAUUGCCAGGGAUUUCC | 0.3875 | 1.1539 | -16.6707 | -11.855 | -0.0289 | 0.3463 |
| hsa-miR-23a* | MI0000079 | GGGGUUCCUGGGGAUGGGAUUU | 0.3955 | 0.5907 | 1.2525 | 1.3736 | 0.9037 | 1.087 |
| hsa-miR-23b | MI0000439 | AUCACAUUGCCAGGGAUUACC | 0.4377 | 0.5085 | 0.919 | 0.9319 | 0.4566 | 0.5237 |
| hsa-miR-23b* | MI0000439 | UGGGUUCCUGGCAUGCUGAUUU | 0.6876 | 1.2878 | 0.7223 | 2.112 | 0.562 | 1.7155 |
| hsa-miR-24 | MI0000081 | UGGCUCAGUUCAGCAGGAACAG | 0.2007 | 0.3218 | 0.5296 | 0.6044 | 0.3894 | 0.4842 |
| hsa-miR-24 | MI0000080 | UGGCUCAGUUCAGCAGGAACAG | 0.8864 | 0.6182 | 0.6879 | 0.6184 | 1.2022 | 0.8101 |
| hsa-miR-24-2* | MI0000081 | UGCCUACUGAGCUGAAACACAG | 0.5335 | 0.691 | 0.7287 | 1.351 | 0.7841 | 0.8988 |
| hsa-miR-25 | MI0000082 | CAUUGCACUUGUCUCGGUCUGA | 0.495 | 0.4562 | 11.1052 | 2.8473 | 0.7132 | 0.6176 |
| hsa-miR-25* | MI0000082 | AGGCGGAGACUUGGGCAAUUG | 0.7633 | 1.7493 | 0.6967 | 1.131 | 0.5953 | 1.3674 |
| hsa-miR-26a | MI0000083 | UUCAAGUAAUCCAGGAUAGGCU | 0.6112 | 0.643 | 0.6161 | 0.5682 | 0.5264 | 0.4958 |
| hsa-miR-26a | MI0000750 | UUCAAGUAAUCCAGGAUAGGCU | 0.8816 | 1.2655 | 0.6637 | 0.7814 | 0.8062 | 0.624 |
| hsa-miR-26a-1* | MI0000083 | CCUAUUCUUGGUUACUUGCACG | 1.0893 | 1.2411 | 1.3212 | 1.2427 | 0.606 | 0.7847 |
| hsa-miR-26a-2* | MI0000750 | CCUAUUCUUGAUUACUUGUUUC | 1.1706 | 0.6449 | 0.6617 | 0.9945 | 0.3908 | 1.1871 |
| hsa-miR-26b | MI0000084 | UUCAAGUAAUUCAGGAUAGGU | 1.4323 | 1.0381 | 1.541 | 0.5239 | 0.732 | 0.5013 |
| hsa-miR-26b* | MI0000084 | CCUGUUCUCCAUUACUUGGCUC | 0.9878 | 0.6779 | 0.5716 | 0.6023 | 0.5679 | 1.0685 |
| hsa-miR-27a | MI0000085 | UUCACAGUGGCUAAGUUCCGC | 0.5017 | 1.0817 | 0.2944 | 0.6085 | -0.3933 | -0.2301 |
| hsa-miR-27a* | MI0000085 | AGGGCUUAGCUGCUUGUGAGCA | 1.076 | 1.7843 | 0.6005 | 0.8746 | 0.4011 | 0.6675 |
| hsa-miR-27b | MI0000440 | UUCACAGUGGCUAAGUUCUGC | 0.56 | 0.5626 | 1.0789 | 0.9782 | 0.3681 | 0.2947 |
| hsa-miR-27b* | MI0000440 | AGAGCUUAGCUGAUUGGUGAAC | 0.7281 | 0.4772 | 0.718 | 1.2605 | 0.3212 | 0.4375 |
| hsa-miR-28 | MI0000086 | AAGGAGCUCACAGUCUAUUGAG | 0.5981 | 0.6424 | 0.6309 | 0.6885 | 0.9898 | 1.3712 |
| hsa-miR-28-3p | MI0000086 | CACUAGAUUGUGAGCUCCUGGA | 0.6439 | 2.4839 | 0.7081 | 0.9282 | 0.6946 | 0.6543 |
| hsa-miR-296 | MI0000747 | AGGGCCCCCCCUCAAUCCUGU | 0.488 | 0.6816 | 0.4495 | 0.9494 | 0.4142 | 0.9338 |
| hsa-miR-296-3p | MI0000747 | GAGGGUUGGGUGGAGGCUCUCC | 0.7316 | 0.4234 | 0.7593 | 0.973 | 0.8325 | 0.5505 |
| hsa-miR-297 | MI0005775 | AUGUAUGUGUGCAUGUGCAUG | 0.5622 | 0.5167 | 0.4919 | 0.5193 | 0.4317 | 0.3347 |
| hsa-miR-298 | MI0005523 | AGCAGAAGCAGGGAGGUUCUCCCA | 0.3957 | 0.6145 | 0.5113 | 0.7299 | 0.6292 | 0.7391 |
| hsa-miR-299-3p | MI0000744 | UAUGUGGGAUGGUAAACCGCUU | 0.5246 | 0.6014 | 0.4809 | 0.5691 | 0.696 | 1.6649 |
| hsa-miR-299-5p | MI0000744 | UGGUUUACCGUCCCACAUACAU | 0.7296 | 0.8043 | 0.5254 | 0.8309 | 0.2984 | 0.5223 |
| hsa-miR-29a | MI0000087 | UAGCACCAUCUGAAAUCGGUUA | 0.9539 | 0.3595 | 1.285 | 0.6198 | 1.3378 | 0.866 |
| hsa-miR-29a* | MI0000087 | ACUGAUUUCUUUUGGUGUUCAG | 0.6046 | 0.8577 | 0.5841 | 0.8317 | 0.7396 | 0.4968 |
| hsa-miR-29b | MI0000107 | UAGCACCAUUUGAAAUCAGUGUU | 0.4064 | 0.6117 | 0.923 | 1.3575 | 0.607 | 0.9195 |
| hsa-miR-29b | MI0000105 | UAGCACCAUUUGAAAUCAGUGUU | 0.5821 | 0.8337 | 1.195 | 1.438 | 0.658 | 0.9237 |
| hsa-miR-29b-1* | MI0000105 | GCUGGUUUCAUAUGGUGGUUUAGA | 0.4578 | 0.9647 | 0.5634 | 1.1849 | 1.0541 | 1.328 |
| hsa-miR-29b-2* | MI0000107 | CUGGUUUCACAUGGUGGCUUAG | 0.6393 | 0.5525 | 0.6602 | 0.8617 | 0.4434 | 0.5791 |
| hsa-miR-29c | MI0000735 | UAGCACCAUUUGAAAUCGGUUA | 1.49 | 1.2459 | 1.4476 | 0.4398 | 0.9366 | 0.8079 |
| hsa-miR-29c* | MI0000735 | UGACCGAUUUCUCCUGGUGUUC | 0.5153 | 0.8813 | 0.7179 | 0.9338 | 0.68 | 0.578 |
| hsa-miR-300 | MI0005525 | UAUACAAGGGCAGACUCUCUCU | 0.8305 | 1.0896 | 0.6191 | 0.8327 | 0.7392 | 0.6016 |
| hsa-miR-301 | MI0000745 | CAGUGCAAUAGUAUUGUCAAAGC | 0.3865 | 0.3428 | 0.6117 | 0.8735 | 1.037 | 1.2144 |
| hsa-miR-301b | MI0005568 | CAGUGCAAUGAUAUUGUCAAAGC | 0.7201 | 1.036 | 0.5774 | 0.9005 | 0.2388 | 0.425 |
| hsa-miR-302a | MI0000738 | UAAGUGCUUCCAUGUUUUGGUGA | 0.5159 | 0.5542 | 0.9465 | 0.5291 | 0.4828 | 0.4447 |
| hsa-miR-302a* | MI0000738 | ACUUAAACGUGGAUGUACUUGCU | 0.8272 | 0.9452 | 1.8613 | 0.9268 | 0.8249 | 0.7268 |
| hsa-miR-302b | MI0000772 | UAAGUGCUUCCAUGUUUUAGUAG | 0.5657 | 0.5536 | 0.9856 | 0.9307 | 0.4667 | 0.503 |
| hsa-miR-302b* | MI0000772 | ACUUUAACAUGGAAGUGCUUUC | 0.6934 | 1.0327 | 1.6923 | 0.7871 | 0.6627 | 0.7467 |
| hsa-miR-302c | MI0000773 | UAAGUGCUUCCAUGUUUCAGUGG | 0.8452 | 0.7251 | 1.1615 | 1.1726 | 0.4621 | 0.3392 |
| hsa-miR-302c* | MI0000773 | UUUAACAUGGGGGUACCUGCUG | 0.5488 | 0.9446 | 1.0433 | 0.9513 | 0.4963 | 2.021 |
| hsa-miR-302d | MI0000774 | UAAGUGCUUCCAUGUUUGAGUGU | 0.4901 | 0.5059 | 1.2377 | 1.365 | 0.525 | 1.4116 |
| hsa-miR-302d* | MI0000774 | ACUUUAACAUGGAGGCACUUGC | 0.6866 | 0.5721 | 0.8379 | 1.3199 | 0.6662 | 0.6725 |
| hsa-miR-30a-3p | MI0000088 | CUUUCAGUCGGAUGUUUGCAGC | 0.534 | 0.5584 | 0.7874 | 0.3997 | -0.0515 | 0.1206 |
| hsa-miR-30a-5p | MI0000088 | UGUAAACAUCCUCGACUGGAAG | 0.1001 | 0.6674 | 0.4144 | 0.6068 | 0.4051 | 0.9215 |
| hsa-miR-30b | MI0000441 | UGUAAACAUCCUACACUCAGCU | 0.9463 | 0.732 | 0.6274 | 0.9185 | 0.1293 | 0.6617 |
| hsa-miR-30b* | MI0000441 | CUGGGAGGUGGAUGUUUACUUC | 0.4377 | 0.6123 | 0.4516 | 0.5972 | 0.1293 | 0.2617 |
| hsa-miR-30c | MI0000254 | UGUAAACAUCCUACACUCUCAGC | 0.2844 | 0.5487 | 0.4788 | 0.6175 | 0.4402 | 0.4524 |
| hsa-miR-30c | MI0000736 | UGUAAACAUCCUACACUCUCAGC | 0.3095 | 1.667 | 0.8713 | 1.0313 | 0.851 | 1.3754 |
| hsa-miR-30c-1* | MI0000736 | CUGGGAGAGGGUUGUUUACUCC | 0.9977 | 1.4566 | 0.6143 | 0.8563 | 0.3123 | 0.159 |
| hsa-miR-30c-2* | MI0000254 | CUGGGAGAAGGCUGUUUACUCU | 1.0911 | 1.1764 | 0.5387 | 0.7798 | 0.5291 | 0.6927 |
| hsa-miR-30d | MI0000255 | UGUAAACAUCCCCGACUGGAAG | 0.339 | 0.8758 | 0.3937 | 0.6395 | 0.4683 | 0.544 |
| hsa-miR-30d* | MI0000255 | CUUUCAGUCAGAUGUUUGCUGC | 0.9208 | 0.4995 | 0.807 | 0.6738 | 0.6368 | 0.5872 |
| hsa-miR-30e-3p | MI0000749 | CUUUCAGUCGGAUGUUUACAGC | 0.3947 | 0.384 | 0.3131 | 0.4138 | 0.4945 | 0.403 |
| hsa-miR-30e-5p | MI0000749 | UGUAAACAUCCUUGACUGGAAG | 1.0335 | 1.7708 | 0.6402 | 1.2229 | 0.364 | 0.9774 |
| hsa-miR-31 | MI0000089 | AGGCAAGAUGCUGGCAUAGCU | 0.7626 | 1.5978 | 1.0238 | 1.0258 | 0.5533 | 1.5535 |
| hsa-miR-31* | MI0000089 | UGCUAUGCCAACAUAUUGCCAU | 0.6346 | 0.6589 | 0.7842 | 0.8532 | 0.4057 | 0.6098 |
| hsa-miR-32 | MI0000090 | UAUUGCACAUUACUAAGUUGCA | 2.353 | 1.0399 | 1.3303 | 0.6378 | 0.5686 | 0.776 |
| hsa-miR-32* | MI0000090 | CAAUUUAGUGUGUGUGAUAUUU | 0.7302 | 1.6274 | 0.6476 | 0.9048 | 0.8634 | 0.6853 |
| hsa-miR-320 | MI0000542 | AAAAGCUGGGUUGAGAGGGCGA | 0.9731 | 1.6485 | 0.7856 | 0.8257 | 0.6267 | 0.5281 |
| hsa-miR-323 | MI0000807 | CACAUUACACGGUCGACCUCU | 1.1047 | 1.2606 | 0.6438 | 0.7201 | 0.5276 | 0.2916 |
| hsa-miR-323-5p | MI0000807 | AGGUGGUCCGUGGCGCGUUCGC | 0.6634 | 1.3872 | 0.663 | 1.9035 | 0.5831 | 2.3499 |
| hsa-miR-324-3p | MI0000813 | ACUGCCCCAGGUGCUGCUGG | 0.8315 | 1.2945 | 0.6624 | 1.1587 | 0.5866 | 0.4999 |
| hsa-miR-324-5p | MI0000813 | CGCAUCCCCUAGGGCAUUGGUGU | 0.5083 | 0.9725 | 0.3651 | 0.8839 | 0.3743 | 1.4553 |
| hsa-miR-325 | MI0000824 | CCUAGUAGGUGUCCAGUAAGUGU | 0.7215 | 0.6279 | 0.7454 | 0.8551 | 1.0154 | 1.043 |
| hsa-miR-326 | MI0000808 | CCUCUGGGCCCUUCCUCCAG | 0.2343 | 0.7429 | 0.642 | 0.8613 | 0.5342 | 0.6468 |
| hsa-miR-328 | MI0000804 | CUGGCCCUCUCUGCCCUUCCGU | 0.3006 | 0.7074 | 0.4426 | 0.8739 | 0.5226 | 1.2446 |
| hsa-miR-329 | MI0001725 | AACACACCUGGUUAACCUCUUU | 0.4606 | 0.5414 | 1.0622 | 0.8795 | 0.4617 | 1.2169 |
| hsa-miR-329 | MI0001726 | AACACACCUGGUUAACCUCUUU | 1.7954 | 0.6611 | 1.405 | 0.9193 | 1.0906 | 1.8914 |
| hsa-miR-33 | MI0000091 | GUGCAUUGUAGUUGCAUUGCA | 1.7567 | 3.1366 | 0.691 | 0.9697 | 0.9066 | 1.2209 |
| hsa-miR-330 | MI0000803 | GCAAAGCACACGGCCUGCAGAGA | 2.0192 | 1.1301 | 0.7161 | 1.0414 | 0.8535 | 0.764 |
| hsa-miR-330-5p | MI0000803 | UCUCUGGGCCUGUGUCUUAGGC | 0.7896 | 0.4726 | 0.4735 | 0.7389 | 0.9317 | 0.8425 |
| hsa-miR-331 | MI0000812 | GCCCCUGGGCCUAUCCUAGAA | 1.0615 | 0.8102 | 1.1579 | 1.2236 | 10.1432 | 1.9871 |
| hsa-miR-331-5p | MI0000812 | CUAGGUAUGGUCCCAGGGAUCC | 0.7027 | 0.655 | 0.5577 | 1.1324 | 0.8562 | 0.5062 |
| hsa-miR-335 | MI0000816 | UCAAGAGCAAUAACGAAAAAUGU | 0.4184 | 0.7291 | 0.4637 | 0.5353 | 0.2155 | 0.4665 |
| hsa-miR-335* | MI0000816 | UUUUUCAUUAUUGCUCCUGACC | 0.6528 | 0.3686 | 0.6484 | 0.9037 | 0.5357 | 0.5006 |
| hsa-miR-337 | MI0000806 | CUCCUAUAUGAUGCCUUUCUUC | 0.9509 | 1.648 | 0.6868 | 1.3498 | 0.6277 | 0.808 |
| hsa-miR-337-5p | MI0000806 | GAACGGCUUCAUACAGGAGUU | 0.8993 | 0.3647 | 0.8315 | 1.4134 | 0.7601 | 1.4937 |
| hsa-miR-338 | MI0000814 | UCCAGCAUCAGUGAUUUUGUUG | 0.8555 | 1.3632 | 0.732 | 0.9975 | 0.6853 | 0.6022 |
| hsa-miR-338-5p | MI0000814 | AACAAUAUCCUGGUGCUGAGUG | 0.6758 | 0.612 | 0.6647 | 0.7441 | 0.7744 | 0.9621 |
| hsa-miR-339 | MI0000815 | UCCCUGUCCUCCAGGAGCUCACG | 0.6775 | 1.5668 | 2.441 | 0.6211 | 0.4943 | 0.646 |
| hsa-miR-339-3p | MI0000815 | UGAGCGCCUCGACGACAGAGCCG | 0.9347 | 0.8281 | 0.5407 | 0.8457 | 0.8769 | 0.7768 |
| hsa-miR-33a* | MI0000091 | CAAUGUUUCCACAGUGCAUCAC | 0.8475 | 0.6189 | 0.5854 | 0.5441 | 0.8652 | 0.528 |
| hsa-miR-33b | MI0003646 | GUGCAUUGCUGUUGCAUUGC | 1.0823 | 1.1664 | 0.4447 | 0.6895 | 0.9259 | 0.8636 |
| hsa-miR-33b* | MI0003646 | CAGUGCCUCGGCAGUGCAGCCC | 0.6009 | 0.7086 | 0.6735 | 0.8939 | 0.4742 | 0.4031 |
| hsa-miR-340 | MI0000802 | UUAUAAAGCAAUGAGACUGAUU | 0.7457 | 0.6562 | 0.7357 | 0.7232 | 0.7405 | 1.1588 |
| hsa-miR-340* | MI0000802 | UCCGUCUCAGUUACUUUAUAGC | 0.91 | 2.0775 | 0.6574 | 1.0298 | 0.7336 | 0.7497 |
| hsa-miR-342 | MI0000805 | UCUCACACAGAAAUCGCACCCGU | 0.9607 | 1.5647 | 0.6681 | 1.0497 | 0.4481 | 0.6888 |
| hsa-miR-342-5p | MI0000805 | AGGGGUGCUAUCUGUGAUUGA | 0.3519 | 0.3731 | 0.691 | 1.3985 | 0.3755 | 0.5391 |
| hsa-miR-345 | MI0000825 | GCUGACUCCUAGUCCAGGGCUC | 0.8193 | 0.6639 | 1.0163 | 0.6574 | 0.8085 | 0.8493 |
| hsa-miR-346 | MI0000826 | UGUCUGCCCGCAUGCCUGCCUCU | 0.5557 | 2.2225 | 0.4312 | 0.7476 | 0.574 | 0.6857 |
| hsa-miR-34a | MI0000268 | UGGCAGUGUCUUAGCUGGUUGU | 0.3642 | 0.8377 | 0.4134 | 0.7687 | 0.9131 | 2.3066 |
| hsa-miR-34a* | MI0000268 | CAAUCAGCAAGUAUACUGCCCU | 0.7124 | 0.3383 | 0.4408 | 0.2547 | 0.4353 | 0.4001 |
| hsa-miR-34b | MI0000742 | CAAUCACUAACUCCACUGCCAU | 0.7589 | -0.4878 | 1.0226 | 1.574 | 0.356 | 0.3851 |
| hsa-miR-34b* | MI0000742 | UAGGCAGUGUCAUUAGCUGAUUG | 0.5939 | 0.8898 | 0.6301 | 1.0121 | 0.8569 | 1.774 |
| hsa-miR-34c | MI0000743 | AGGCAGUGUAGUUAGCUGAUUGC | 0.34 | 0.7914 | 0.0151 | 0.4857 | 0.3565 | 0.4435 |
| hsa-miR-34c-3p | MI0000743 | AAUCACUAACCACACGGCCAGG | 0.8512 | 1.3494 | 0.6991 | 2.2666 | 1.2988 | 1.3768 |
| hsa-miR-361 | MI0000760 | UUAUCAGAAUCUCCAGGGGUAC | 0.3153 | 0.4995 | 0.9757 | 0.9784 | 0.4955 | 0.572 |
| hsa-miR-361-3p | MI0000760 | UCCCCCAGGUGUGAUUCUGAUUU | 0.6208 | 0.489 | 0.5901 | 0.6403 | 0.6957 | 0.657 |
| hsa-miR-362 | MI0000762 | AAUCCUUGGAACCUAGGUGUGAGU | 0.4916 | 0.6019 | 0.8265 | 0.7083 | 2.5679 | 1.9043 |
| hsa-miR-362-3p | MI0000762 | AACACACCUAUUCAAGGAUUCA | 0.4366 | 1.693 | 0.6128 | 0.9958 | 1.0428 | 1.003 |
| hsa-miR-363 | MI0000764 | AAUUGCACGGUAUCCAUCUGUA | 0.3741 | 0.3811 | 8.5822 | 4.5576 | 1.2562 | 1.1256 |
| hsa-miR-363* | MI0000764 | CGGGUGGAUCACGAUGCAAUUU | 0.5264 | 0.8506 | 0.7893 | 0.9792 | 0.4033 | 1.2564 |
| hsa-miR-365 | MI0000767 | UAAUGCCCCUAAAAAUCCUUAU | 0.5625 | 0.705 | 0.4554 | 0.5476 | 0.5308 | 0.7455 |
| hsa-miR-365 | MI0000769 | UAAUGCCCCUAAAAAUCCUUAU | 0.569 | 0.8739 | 0.4733 | 0.5489 | 1.2063 | 1.0873 |
| hsa-miR-367 | MI0000775 | AAUUGCACUUUAGCAAUGGUGA | 0.4597 | 0.5368 | 1.2711 | 1.2774 | 0.527 | 0.4565 |
| hsa-miR-367* | MI0000775 | ACUGUUGCUAAUAUGCAACUCU | 0.8418 | 0.3198 | 0.3306 | 0.5542 | 0.3636 | 0.4509 |
| hsa-miR-368 | MI0000776 | AACAUAGAGGAAAUUCCACGU | 1.1369 | 2.2357 | 0.7322 | 1.1776 | 0.5262 | 1.623 |
| hsa-miR-369-3p | MI0000777 | AAUAAUACAUGGUUGAUCUUU | 0.5668 | 3.7047 | 0.1794 | 0.9591 | 0.6486 | 0.7907 |
| hsa-miR-369-5p | MI0000777 | AGAUCGACCGUGUUAUAUUCGC | 1.5214 | 0.7063 | 1.18 | 0.8496 | 1.3127 | 1.5552 |
| hsa-miR-370 | MI0000778 | GCCUGCUGGGGUGGAACCUGGU | 0.9632 | 2.0638 | 0.7432 | 1.1739 | 0.3781 | 0.7418 |
| hsa-miR-371 | MI0000779 | AAGUGCCGCCAUCUUUUGAGUGU | 0.522 | 0.9722 | 0.4526 | 0.5515 | 0.2849 | 0.3793 |
| hsa-miR-371-5p | MI0000779 | ACUCAAACUGUGGGGGCACU | 0.7961 | 0.6188 | 0.9052 | 1.0485 | 1.0909 | 0.8367 |
| hsa-miR-372 | MI0000780 | AAAGUGCUGCGACAUUUGAGCGU | 0.3698 | 0.4971 | 0.4392 | 0.9335 | 0.0898 | 0.3206 |
| hsa-miR-373 | MI0000781 | GAAGUGCUUCGAUUUUGGGGUGU | 0.3908 | 0.4454 | 0.3819 | 0.6465 | 0.0858 | 0.2568 |
| hsa-miR-373* | MI0000781 | ACUCAAAAUGGGGGCGCUUUCC | 1.0171 | 2.3842 | 0.2224 | 1.3558 | 0.7931 | 0.9748 |
| hsa-miR-374 | MI0000782 | UUAUAAUACAACCUGAUAAGUG | 0.8482 | 0.964 | 0.4305 | 1.1048 | 0.3761 | 0.2308 |
| hsa-miR-374a* | MI0000782 | CUUAUCAGAUUGUAUUGUAAUU | 1.3069 | 0.6418 | 0.6686 | 0.8163 | 0.7243 | 0.7749 |
| hsa-miR-374b | MI0005566 | AUAUAAUACAACCUGCUAAGUG | 0.9627 | 1.4228 | 0.6737 | 1.0792 | 0.5709 | 0.738 |
| hsa-miR-374b* | MI0005566 | CUUAGCAGGUUGUAUUAUCAUU | 0.825 | 5.403 | 0.676 | 1.1265 | 0.6432 | 0.5657 |
| hsa-miR-375 | MI0000783 | UUUGUUCGUUCGGCUCGCGUGA | 0.6695 | 0.5949 | 0.8842 | 1.1832 | 0.5944 | 0.5517 |
| hsa-miR-376a | MI0000784 | AUCAUAGAGGAAAAUCCACGU | 1.235 | 1.369 | 0.5069 | 0.8189 | 0.5908 | 1.1705 |
| hsa-miR-376a | MI0003529 | AUCAUAGAGGAAAAUCCACGU | 2.0483 | 2.2013 | 0.6902 | 1.2358 | 0.6024 | 1.7725 |
| hsa-miR-376a* | MI0000784 | GUAGAUUCUCCUUCUAUGAGUA | 0.7604 | 1.5124 | 0.4362 | 0.5978 | 0.8702 | 0.5179 |
| hsa-miR-376b | MI0002466 | AUCAUAGAGGAAAAUCCAUGUU | 0.5744 | 0.8339 | 0.9788 | 0.7502 | 0.6685 | 1.6763 |
| hsa-miR-377 | MI0000785 | AUCACACAAAGGCAACUUUUGU | 0.7876 | 0.7182 | 0.5036 | 0.7231 | 0.393 | 1.0059 |
| hsa-miR-377* | MI0000785 | AGAGGUUGCCCUUGGUGAAUUC | 0.8385 | 1.2988 | 0.6827 | 0.6243 | 0.6623 | 2.0032 |
| hsa-miR-378 | MI0000786 | CUCCUGACUCCAGGUCCUGUGU | 0.5343 | 0.8579 | 0.6326 | 0.872 | 0.6692 | 1.1593 |
| hsa-miR-379 | MI0000787 | UGGUAGACUAUGGAACGUAGG | 0.7881 | 1.4941 | 0.6388 | 1.0044 | 0.5235 | 1.0587 |
| hsa-miR-379* | MI0000787 | UAUGUAACAUGGUCCACUAACU | 0.7932 | 0.9042 | 0.7738 | 0.617 | 0.6121 | 0.8212 |
| hsa-miR-380-3p | MI0000788 | UAUGUAAUAUGGUCCACAUCUU | -0.0319 | 0.6426 | 0.7994 | 1.13 | 0.5152 | 1.9547 |
| hsa-miR-380-5p | MI0000788 | UGGUUGACCAUAGAACAUGCGC | 0.3579 | 0.4351 | 0.3567 | 0.7723 | 0.8076 | 1.1631 |
| hsa-miR-381 | MI0000789 | UAUACAAGGGCAAGCUCUCUGU | 0.4754 | 0.6033 | 0.5113 | 0.7198 | 0.5505 | 1.1348 |
| hsa-miR-382 | MI0000790 | GAAGUUGUUCGUGGUGGAUUCG | 1.0258 | 1.0623 | 0.7419 | 1.1235 | 0.8577 | 0.4101 |
| hsa-miR-383 | MI0000791 | AGAUCAGAAGGUGAUUGUGGCU | 0.2546 | 0.6813 | 0.5691 | 0.7806 | 0.7409 | 1.0159 |
| hsa-miR-384 | MI0001145 | AUUCCUAGAAAUUGUUCAUA | 1.1666 | 0.984 | 0.7022 | 0.575 | 1.6838 | 1.4028 |
| hsa-miR-409-3p | MI0001735 | GAAUGUUGCUCGGUGAACCCCU | 0.8487 | 0.955 | 1.1136 | 1.1925 | 0.5819 | 0.7643 |
| hsa-miR-409-5p | MI0001735 | AGGUUACCCGAGCAACUUUGCAU | 0.9083 | 0.8977 | 1.0778 | 0.7401 | 0.6814 | 0.7311 |
| hsa-miR-410 | MI0002465 | AAUAUAACACAGAUGGCCUGU | 1.6818 | 0.6664 | 0.8257 | 0.7729 | 1.0765 | 1.269 |
| hsa-miR-411 | MI0003675 | UAGUAGACCGUAUAGCGUACG | 0.4854 | 0.4354 | 0.4408 | 0.2734 | 0.45 | 0.4172 |
| hsa-miR-411* | MI0003675 | UAUGUAACACGGUCCACUAACC | 0.8681 | 0.8835 | 0.3975 | 0.2701 | 0.8431 | 1.1635 |
| hsa-miR-412 | MI0002464 | ACUUCACCUGGUCCACUAGCCGU | 0.5738 | 0.4195 | 1.125 | 0.7085 | 1.0593 | 1.4878 |
| hsa-miR-421 | MI0003685 | AUCAACAGACAUUAAUUGGGCGC | 0.3245 | 0.2932 | 0.7558 | 0.7359 | 0.5162 | 0.3645 |
| hsa-miR-422a | MI0001444 | ACUGGACUUAGGGUCAGAAGGC | 1.2236 | 0.9395 | 1.0562 | 0.8587 | 0.5773 | 0.8644 |
| hsa-miR-422b | MI0000786 | ACUGGACUUGGAGUCAGAAGG | 0.8383 | 1.2228 | 0.5312 | 0.9185 | 0.6105 | 0.9082 |
| hsa-miR-423 | MI0001445 | AGCUCGGUCUGAGGCCCCUCAGU | 0.8765 | 1.1109 | 1.0183 | 1.1716 | 0.8196 | 2.3211 |
| hsa-miR-423-5p | MI0001445 | UGAGGGGCAGAGAGCGAGACUUU | 0.9639 | 0.8283 | 0.5233 | 1.1641 | 0.418 | 2.1111 |
| hsa-miR-424 | MI0001446 | CAGCAGCAAUUCAUGUUUUGAA | 0.8513 | 1.0055 | 0.5709 | 0.7485 | 0.4887 | 0.6624 |
| hsa-miR-424* | MI0001446 | CAAAACGUGAGGCGCUGCUAU | 0.654 | 0.6448 | 0.602 | 0.8813 | 0.7259 | 1.1135 |
| hsa-miR-425 | MI0001448 | AUCGGGAAUGUCGUGUCCGCCC | 0.6727 | 1.3147 | 0.5563 | 0.9334 | 0.8433 | 0.9403 |
| hsa-miR-425-5p | MI0001448 | AAUGACACGAUCACUCCCGUUGA | 0.5168 | 0.619 | 0.9114 | 0.8088 | 0.5317 | 0.6476 |
| hsa-miR-429 | MI0001641 | UAAUACUGUCUGGUAAAACCGU | 0.4992 | 0.5969 | 1.8009 | 1.5138 | 0.5275 | 0.6066 |
| hsa-miR-431 | MI0001721 | UGUCUUGCAGGCCGUCAUGCA | 0.1475 | 0.141 | 1.0792 | 0.8906 | 0.5801 | 1.5309 |
| hsa-miR-431* | MI0001721 | CAGGUCGUCUUGCAGGGCUUCU | 0.686 | 0.5726 | 0.5723 | 0.3992 | 0.4477 | 0.3844 |
| hsa-miR-432 | MI0003133 | UCUUGGAGUAGGUCAUUGGGUGG | 1.1707 | 1.2499 | 0.9384 | 0.7779 | 0.8519 | 0.9295 |
| hsa-miR-432* | MI0003133 | CUGGAUGGCUCCUCCAUGUCU | 0.9845 | 0.8776 | 0.8176 | 0.9918 | 0.6744 | 1.0528 |
| hsa-miR-433 | MI0001723 | AUCAUGAUGGGCUCCUCGGUGU | -0.1846 | 0.0614 | 0.9652 | 0.7592 | 1.1676 | 1.4909 |
| hsa-miR-448 | MI0001637 | UUGCAUAUGUAGGAUGUCCCAU | 1.0361 | 1.4287 | 0.5654 | 0.5519 | 0.8501 | 0.9369 |
| hsa-miR-449 | MI0001648 | UGGCAGUGUAUUGUUAGCUGGU | 0.6254 | 0.4057 | 1.0656 | 1.1776 | 0.5733 | 2.3643 |
| hsa-miR-449b | MI0003673 | AGGCAGUGUAUUGUUAGCUGGC | 0.3298 | 0.4003 | 0.5221 | 0.4886 | 0.4111 | 0.3 |
| hsa-miR-450 | MI0003187 | UUUUGCGAUGUGUUCCUAAUAU | 1.022 | 1.2399 | 0.5889 | 1.056 | 0.4828 | 1.2991 |
| hsa-miR-450 | MI0001652 | UUUUGCGAUGUGUUCCUAAUAU | 1.2212 | 2.0273 | 0.7545 | 1.14 | 0.9353 | 1.6066 |
| hsa-miR-450b-3p | MI0005531 | UUGGGAUCAUUUUGCAUCCAUA | 0.7368 | 1.0054 | 0.6873 | 1.1338 | 0.6503 | 0.607 |
| hsa-miR-450b-5p | MI0005531 | UUUUGCAAUAUGUUCCUGAAUA | 0.5802 | 0.9538 | 0.8171 | 1.1827 | 0.7886 | 0.6054 |
| hsa-miR-451 | MI0001729 | AAACCGUUACCAUUACUGAGUU | 0.9538 | 0.5741 | 1.5679 | 1.7709 | 2.3268 | 1.5503 |
| hsa-miR-452 | MI0001733 | AACUGUUUGCAGAGGAAACUGA | 1.1991 | 1.014 | 1.3452 | 0.6107 | 0.7299 | 0.8512 |
| hsa-miR-452* | MI0001733 | CUCAUCUGCAAAGAAGUAAGUG | 0.6639 | 0.705 | 1.174 | 0.7495 | 0.6905 | 1.1926 |
| hsa-miR-453 | MI0001727 | AGGUUGUCCGUGGUGAGUUCGCA | 1.1987 | 1.187 | 1.0484 | 0.8218 | 0.625 | 1.0453 |
| hsa-miR-454-3p | MI0003820 | UAGUGCAAUAUUGCUUAUAGGGU | 0.6302 | 0.7242 | 1.3894 | 0.5565 | 0.9263 | 0.6748 |
| hsa-miR-454-5p | MI0003820 | ACCCUAUCAAUAUUGUCUCUGC | 0.9269 | 1.3111 | 0.5002 | 0.3652 | 0.8857 | 2.6737 |
| hsa-miR-455 | MI0003513 | UAUGUGCCUUUGGACUACAUCG | 0.461 | 0.4058 | 9.19 | 5.0136 | 0.4175 | 0.3413 |
| hsa-miR-455-3p | MI0003513 | GCAGUCCAUGGGCAUAUACAC | 0.5414 | 0.6753 | 0.6301 | 0.858 | 0.7991 | 0.7281 |
| hsa-miR-483 | MI0002467 | UCACUCCUCUCCUCCCGUCUU | 0.8505 | 0.6845 | 0.8326 | 0.7863 | 0.7546 | 0.8899 |
| hsa-miR-483-5p | MI0002467 | AAGACGGGAGGAAAGAAGGGAG | 1.0169 | 1.1759 | 0.5364 | 1.4174 | 0.724 | 0.6337 |
| hsa-miR-484 | MI0002468 | UCAGGCUCAGUCCCCUCCCGAU | 1.2779 | 0.628 | 1.1151 | 0.965 | 1.191 | 0.8038 |
| hsa-miR-485-3p | MI0002469 | GUCAUACACGGCUCUCCUCUCU | 1.2707 | 0.7863 | 1.234 | 0.7976 | 0.8991 | 0.7214 |
| hsa-miR-485-5p | MI0002469 | AGAGGCUGGCCGUGAUGAAUUC | 0.1577 | 0.4798 | 0.948 | 0.7848 | 1.2293 | 1.5182 |
| hsa-miR-486 | MI0002470 | UCCUGUACUGAGCUGCCCCGAG | 0.5478 | 2.4938 | 0.6801 | 0.5921 | 0.7282 | 0.9809 |
| hsa-miR-486-3p | MI0002470 | CGGGGCAGCUCAGUACAGGAU | 0.2355 | 0.6713 | 0.4209 | 0.8231 | 0.1918 | 0.6721 |
| hsa-miR-487 | MI0002471 | AAUCAUACAGGGACAUCCAGUU | 0.6957 | 0.8069 | 1.085 | 0.9977 | 0.2766 | 0.6514 |
| hsa-miR-487b | MI0003530 | AAUCGUACAGGGUCAUCCACUU | 0.7798 | 0.7181 | 1.1768 | 1.0651 | 0.6353 | 0.8354 |
| hsa-miR-488 | MI0003123 | UUGAAAGGCUAUUUCUUGGUC | 0.7299 | 1.047 | 0.6883 | 0.9744 | 0.5698 | 0.537 |
| hsa-miR-488* | MI0003123 | CCCAGAUAAUGGCACUCUCAA | 0.2026 | 0.4169 | 0.3564 | 0.2098 | 0.3817 | 0.5345 |
| hsa-miR-489 | MI0003124 | GUGACAUCACAUAUACGGCAGC | 1.2112 | 1.0315 | 1.5535 | 0.7488 | 0.6456 | 0.457 |
| hsa-miR-490 | MI0003125 | CAACCUGGAGGACUCCAUGCUG | 0.6167 | 0.9446 | 0.4895 | 0.5465 | 0.5968 | 1.3968 |
| hsa-miR-490-5p | MI0003125 | CCAUGGAUCUCCAGGUGGGU | 0.3684 | 1.0595 | 0.621 | 0.8627 | 0.4224 | 0.6567 |
| hsa-miR-491 | MI0003126 | AGUGGGGAACCCUUCCAUGAGG | 0.4484 | 0.6766 | 0.5161 | 0.6801 | 0.3335 | 0.7533 |
| hsa-miR-491-3p | MI0003126 | CUUAUGCAAGAUUCCCUUCUAC | 0.9925 | 0.8164 | 0.7364 | 1.0714 | 0.6221 | 1.1869 |
| hsa-miR-492 | MI0003131 | AGGACCUGCGGGACAAGAUUCUU | 0.5314 | 0.7107 | 0.8552 | 0.551 | 1.356 | 1.6009 |
| hsa-miR-493 | MI0003132 | UUGUACAUGGUAGGCUUUCAUU | 0.8808 | 0.5584 | 1.0474 | 0.7923 | 0.8988 | 1.4376 |
| hsa-miR-493-3p | MI0003132 | UGAAGGUCUACUGUGUGCCAGG | 0.6517 | 0.9132 | 0.4162 | 0.7329 | 0.3855 | 0.7132 |
| hsa-miR-494 | MI0003134 | UGAAACAUACACGGGAAACCUC | 0.5879 | 0.5657 | 0.895 | 0.8429 | 0.619 | 1.2784 |
| hsa-miR-495 | MI0003135 | AAACAAACAUGGUGCACUUCUU | 0.8418 | 0.5575 | 1.0418 | 0.7829 | 0.6926 | 0.5677 |
| hsa-miR-496 | MI0003136 | UGAGUAUUACAUGGCCAAUCUC | 1.0179 | 1.0446 | 0.8798 | 0.6762 | 0.7853 | 0.7428 |
| hsa-miR-497 | MI0003138 | CAGCAGCACACUGUGGUUUGU | 0.9563 | 1.1785 | 1.183 | 1.1853 | 0.9874 | 1.3454 |
| hsa-miR-497* | MI0003138 | CAAACCACACUGUGGUGUUAGA | 0.734 | 0.4997 | 0.5008 | -0.5083 | 0.2824 | 0.1069 |
| hsa-miR-498 | MI0003142 | UUUCAAGCCAGGGGGCGUUUUUC | 0.7297 | 0.9147 | 0.893 | 1.0677 | 0.5654 | 1.0594 |
| hsa-miR-499 | MI0003183 | UUAAGACUUGCAGUGAUGUUU | 1.1807 | 3.2872 | 0.5256 | 0.8612 | 1.0374 | 1.4681 |
| hsa-miR-499-3p | MI0003183 | AACAUCACAGCAAGUCUGUGCU | 0.8937 | 1.6339 | 0.8608 | 1.6199 | 0.5107 | 0.7218 |
| hsa-miR-500 | MI0003184 | UAAUCCUUGCUACCUGGGUGAGA | 0.5204 | 0.4551 | 0.6934 | 1.4215 | 0.4832 | 0.5399 |
| hsa-miR-500* | MI0003184 | AUGCACCUGGGCAAGGAUUCUG | 0.6516 | 0.5696 | 1.5984 | 1.1691 | 0.6265 | 0.7173 |
| hsa-miR-501 | MI0003185 | AAUCCUUUGUCCCUGGGUGAGA | 0.5551 | 0.7325 | 0.9125 | 0.8858 | 0.4899 | 0.5774 |
| hsa-miR-501-3p | MI0003185 | AAUGCACCCGGGCAAGGAUUCU | 1.0722 | 1.0828 | 0.6936 | 1.5092 | -39.8347 | -43.9762 |
| hsa-miR-502 | MI0003186 | AUCCUUGCUAUCUGGGUGCUA | 0.4979 | 0.5397 | 1.0842 | 1.1385 | 0.4558 | 0.4367 |
| hsa-miR-502-3p | MI0003186 | AAUGCACCUGGGCAAGGAUUCA | 0.228 | 0.7408 | 0.8129 | 1.5955 | 0.7961 | 1.1996 |
| hsa-miR-503 | MI0003188 | UAGCAGCGGGAACAGUUCUGCAG | 0.4038 | 0.7172 | 0.8984 | 1.056 | 0.4047 | 1.0928 |
| hsa-miR-504 | MI0003189 | AGACCCUGGUCUGCACUCUAUC | 0.8452 | 0.9332 | 1.9191 | 0.8653 | 2.0221 | 1.0952 |
| hsa-miR-505 | MI0003190 | CGUCAACACUUGCUGGUUUCCU | 0.5995 | 0.4478 | 1.1979 | 0.5376 | 1.0493 | 1.3518 |
| hsa-miR-505* | MI0003190 | GGGAGCCAGGAAGUAUUGAUGU | 0.5429 | 0.8234 | 0.5883 | 1.0288 | 0.5537 | 0.6322 |
| hsa-miR-506 | MI0003193 | UAAGGCACCCUUCUGAGUAGA | 0.5791 | 0.9175 | 1.4802 | 1.3506 | 0.5282 | 0.6313 |
| hsa-miR-507 | MI0003194 | UUUUGCACCUUUUGGAGUGAA | 0.5243 | 0.8155 | 0.9878 | 0.7948 | 0.4388 | 0.6641 |
| hsa-miR-508 | MI0003195 | UGAUUGUAGCCUUUUGGAGUAGA | 0.3932 | 0.6326 | 1.0366 | 0.9309 | 0.7054 | 0.6741 |
| hsa-miR-508-5p | MI0003195 | UACUCCAGAGGGCGUCACUCAUG | 0.3677 | 0.8455 | 0.7067 | 1.0862 | 0.7359 | 0.9119 |
| hsa-miR-509 | MI0003196 | UGAUUGGUACGUCUGUGGGUAG | 0.7617 | 1.2343 | 1.8275 | 0.7715 | 0.5512 | 0.526 |
| hsa-miR-509-3-5p | MI0005717 | UACUGCAGACGUGGCAAUCAUG | 1.5536 | 1.5728 | 1.2367 | 0.7797 | 0.4454 | 1.7815 |
| hsa-miR-509-3p | MI0005530 | UGAUUGGUACGUCUGUGGGUAG | 0.5451 | 0.6557 | 0.654 | 0.6915 | 0.4439 | 0.3405 |
| hsa-miR-509-3p | MI0005717 | UGAUUGGUACGUCUGUGGGUAG | 0.5764 | 0.8666 | 1.3781 | 0.8562 | 0.5089 | 0.5004 |
| hsa-miR-509-5p | MI0003196 | UACUGCAGACAGUGGCAAUCA | 0.5659 | 0.7529 | 0.5536 | 0.9133 | 0.6878 | 0.5994 |
| hsa-miR-509-5p | MI0005530 | UACUGCAGACAGUGGCAAUCA | 0.7147 | 1.1323 | 0.6606 | 1.3278 | 0.6914 | 0.6801 |
| hsa-miR-510 | MI0003197 | UACUCAGGAGAGUGGCAAUCAC | 1.053 | 1.4145 | 0.3086 | 0.53 | 0.6659 | 0.7092 |
| hsa-miR-511 | MI0003127 | GUGUCUUUUGCUCUGCAGUCA | 0.775 | 0.6979 | 0.8538 | 0.8969 | 0.7422 | 1.1911 |
| hsa-miR-511 | MI0003128 | GUGUCUUUUGCUCUGCAGUCA | 0.972 | 2.8728 | 1.3464 | 1.2616 | 0.9185 | 1.5917 |
| hsa-miR-512-3p | MI0003140 | AAGUGCUGUCAUAGCUGAGGUC | 0.5313 | 0.5065 | 0.7658 | 0.7368 | 0.622 | 0.7895 |
| hsa-miR-512-3p | MI0003141 | AAGUGCUGUCAUAGCUGAGGUC | 0.5341 | 0.7101 | 0.8379 | 0.9597 | 0.7645 | 0.9411 |
| hsa-miR-512-5p | MI0003140 | CACUCAGCCUUGAGGGCACUUUC | 0.7126 | 0.8564 | 0.8168 | 0.5882 | 0.577 | 0.9468 |
| hsa-miR-512-5p | MI0003141 | CACUCAGCCUUGAGGGCACUUUC | 0.8173 | 1.2597 | 0.915 | 0.9231 | 0.886 | 1.1753 |
| hsa-miR-513 | MI0003191 | UUCACAGGGAGGUGUCAU | 0.8736 | 0.9959 | 0.6062 | 0.9676 | 0.4567 | 0.5866 |
| hsa-miR-513 | MI0003192 | UUCACAGGGAGGUGUCAU | 1.1446 | 1.5601 | 0.7083 | 0.9831 | 0.5575 | 0.7289 |
| hsa-miR-513a-3p | MI0003191 | UAAAUUUCACCUUUCUGAGAAGG | 0.8574 | 0.6479 | 0.6357 | 0.96 | 0.5133 | 0.609 |
| hsa-miR-513a-3p | MI0003192 | UAAAUUUCACCUUUCUGAGAAGG | 0.8834 | 0.7216 | 0.7774 | 1.0985 | 0.6302 | 0.7757 |
| hsa-miR-513b | MI0006648 | UUCACAAGGAGGUGUCAUUUAU | 0.4715 | -0.0413 | 0.4499 | 0.9367 | 1.0936 | 1.1524 |
| hsa-miR-513c | MI0006649 | UUCUCAAGGAGGUGUCGUUUAU | 0.8634 | 0.6718 | 0.4925 | 1.1573 | 1.8411 | 1.8495 |
| hsa-miR-514 | MI0003198 | AUUGACACUUCUGUGAGUAGA | 0.8743 | 1.1387 | 0.3516 | 0.5163 | 0.8245 | 0.7172 |
| hsa-miR-514 | MI0003199 | AUUGACACUUCUGUGAGUAGA | 0.9139 | 1.4068 | 0.505 | 0.6625 | 0.841 | 0.8183 |
| hsa-miR-514 | MI0003200 | AUUGACACUUCUGUGAGUAGA | 0.9362 | 1.7634 | 0.6599 | 0.8296 | 0.8996 | 0.8858 |
| hsa-miR-515-3p | MI0003144 | GAGUGCCUUCUUUUGGAGCGUU | 0.4579 | 0.0609 | 0.5008 | 0.4908 | 0.636 | 0.4933 |
| hsa-miR-515-3p | MI0003147 | GAGUGCCUUCUUUUGGAGCGUU | 0.8602 | 0.28 | 0.811 | 0.5014 | 0.927 | 0.6958 |
| hsa-miR-515-5p | MI0003144 | UUCUCCAAAAGAAAGCACUUUCUG | 0.6734 | 0.7841 | 0.9867 | 0.5658 | 0.5368 | 0.6664 |
| hsa-miR-515-5p | MI0003147 | UUCUCCAAAAGAAAGCACUUUCUG | 0.7923 | 1.129 | 1.3223 | 1.1 | 0.7573 | 1.0108 |
| hsa-miR-516-3p | MI0003167 | UGCUUCCUUUCAGAGGGU | 0.6003 | 0.5471 | 0.7669 | 0.5748 | 0.3962 | 0.5068 |
| hsa-miR-516-3p | MI0003172 | UGCUUCCUUUCAGAGGGU | 1.1876 | 0.8627 | 0.8015 | 0.7838 | 0.6107 | 0.6373 |
| hsa-miR-516-3p | MI0003180 | UGCUUCCUUUCAGAGGGU | 1.3035 | 0.9632 | 0.8978 | 0.9147 | 0.6418 | 0.9101 |
| hsa-miR-516-3p | MI0003181 | UGCUUCCUUUCAGAGGGU | 1.5087 | 1.153 | 1.0765 | 0.9295 | 0.7581 | 1.1235 |
| hsa-miR-516-5p | MI0003167 | AUCUGGAGGUAAGAAGCACUUU | 1.1172 | 0.9256 | 0.7371 | 0.8082 | 0.6633 | 0.9467 |
| hsa-miR-516-5p | MI0003172 | AUCUGGAGGUAAGAAGCACUUU | 1.4666 | 1.1922 | 1.0346 | 0.8751 | 0.7662 | 1.0508 |
| hsa-miR-516a-5p | MI0003180 | UUCUCGAGGAAAGAAGCACUUUC | 0.5262 | 0.8564 | 0.4055 | 0.7626 | 1.1946 | 1.4469 |
| hsa-miR-516a-5p | MI0003181 | UUCUCGAGGAAAGAAGCACUUUC | 0.8514 | 1.6827 | 0.5324 | 1.1135 | 1.67 | 1.5334 |
| hsa-miR-517 | MI0003161 | CCUCUAGAUGGAAGCACUGUCU | 0.7198 | 1.3347 | 0.9937 | 0.9657 | 0.4706 | 0.8023 |
| hsa-miR-517* | MI0003165 | CCUCUAGAUGGAAGCACUGUCU | 0.7158 | 0.9737 | 0.9906 | 0.9122 | 0.4321 | 0.7363 |
| hsa-miR-517* | MI0003174 | CCUCUAGAUGGAAGCACUGUCU | 0.7613 | 0.9964 | 1.0763 | 1.0461 | 0.5481 | 0.7735 |
| hsa-miR-517a | MI0003161 | AUCGUGCAUCCCUUUAGAGUGU | 0.6454 | 0.456 | 1.0555 | 0.8391 | 0.7558 | 0.7653 |
| hsa-miR-517b | MI0003165 | UCGUGCAUCCCUUUAGAGUGUU | 0.5409 | 0.7276 | 1.4743 | 1.2569 | 0.482 | 0.9143 |
| hsa-miR-517c | MI0003174 | AUCGUGCAUCCUUUUAGAGUGU | 0.8432 | 0.751 | 1.3378 | 1.1988 | 0.7432 | 0.7083 |
| hsa-miR-518a | MI0003170 | GAAAGCGCUUCCCUUUGCUGGA | 0.8083 | 0.9741 | 0.7594 | 0.8931 | 0.3992 | 0.4225 |
| hsa-miR-518a | MI0003173 | GAAAGCGCUUCCCUUUGCUGGA | 0.8094 | 0.9776 | 1.0779 | 0.9002 | 0.5161 | 0.5178 |
| hsa-miR-518a-5p | MI0003170 | CUGCAAAGGGAAGCCCUUUC | 0.8407 | 0.63 | 0.6571 | 1.497 | 0.5216 | 0.6879 |
| hsa-miR-518a-5p | MI0003173 | CUGCAAAGGGAAGCCCUUUC | 0.988 | 1.521 | 0.673 | 1.7037 | 0.7806 | 0.8546 |
| hsa-miR-518b | MI0003156 | CAAAGCGCUCCCCUUUAGAGGU | 0.6963 | 0.6078 | 0.985 | 0.9331 | 0.8646 | 1.1257 |
| hsa-miR-518c | MI0003159 | CAAAGCGCUUCUCUUUAGAGUGU | 0.9322 | 0.8854 | 0.7685 | 0.8552 | 0.54 | 0.6816 |
| hsa-miR-518c* | MI0003159 | UCUCUGGAGGGAAGCACUUUCUG | 0.785 | 0.6518 | 0.8402 | 1.1448 | 0.8 | 1.3855 |
| hsa-miR-518d | MI0003171 | CAAAGCGCUUCCCUUUGGAGC | 0.6089 | 0.7095 | 2.3663 | 1.295 | 0.6836 | 0.8962 |
| hsa-miR-518d-5p | MI0003171 | CUCUAGAGGGAAGCACUUUCUG | 1.0603 | 0.6455 | 0.6396 | 0.4327 | 1.8686 | 1.129 |
| hsa-miR-518e | MI0003169 | AAAGCGCUUCCCUUCAGAGUG | 0.8128 | 0.9566 | 0.664 | 0.7424 | 0.6739 | 1.1103 |
| hsa-miR-518e* | MI0003169 | CUCUAGAGGGAAGCGCUUUCUG | 0.6114 | 0.3016 | 0.4379 | 0.309 | 1.0942 | 0.8834 |
| hsa-miR-518f | MI0003154 | GAAAGCGCUUCUCUUUAGAGG | 0.7246 | 0.8649 | 1.0525 | 0.8509 | 0.4521 | 0.6355 |
| hsa-miR-518f* | MI0003154 | CUCUAGAGGGAAGCACUUUCUC | 0.5525 | 0.384 | 0.9983 | 0.8044 | 0.5115 | 0.4152 |
| hsa-miR-519a | MI0003178 | AAAGUGCAUCCUUUUAGAGUGU | 0.8644 | 1.2351 | 0.4075 | 0.4432 | 0.5979 | 0.5386 |
| hsa-miR-519a | MI0003182 | AAAGUGCAUCCUUUUAGAGUGU | 1.1438 | 1.444 | 0.5386 | 0.8174 | 1.0566 | 0.865 |
| hsa-miR-519a* | MI0003178 | CUCUAGAGGGAAGCGCUUUCUG | 0.7842 | 0.6954 | 0.6359 | 0.966 | 0.9945 | 1.2581 |
| hsa-miR-519b | MI0003151 | AAAGUGCAUCCUUUUAGAGGUU | 2.3823 | 2.2433 | 0.4962 | 0.5192 | 0.3867 | 0.819 |
| hsa-miR-519b-5p | MI0003151 | CUCUAGAGGGAAGCGCUUUCUG | 0.7179 | 0.4262 | 0.4899 | -0.0309 | 0.2359 | 0.1655 |
| hsa-miR-519c | MI0003148 | AAAGUGCAUCUUUUUAGAGGAU | 0.5995 | 0.7683 | 1.1786 | 0.9664 | 0.6503 | 1.1799 |
| hsa-miR-519d | MI0003162 | CAAAGUGCCUCCCUUUAGAGUG | 0.7466 | 0.8022 | 1.0195 | 0.7788 | 0.5168 | 0.7494 |
| hsa-miR-519e | MI0003145 | AAGUGCCUCCUUUUAGAGUGUU | 0.2619 | 0.6046 | 2.0933 | 1.3886 | 0.5563 | 0.3069 |
| hsa-miR-519e* | MI0003145 | UUCUCCAAAAGGGAGCACUUUC | 0.6978 | 0.7729 | 1.4554 | 1.2746 | 0.7568 | 1.0432 |
| hsa-miR-520a | MI0003149 | AAAGUGCUUCCCUUUGGACUGU | 0.9969 | 0.5788 | 0.8399 | 0.9807 | 0.8495 | 0.9037 |
| hsa-miR-520a* | MI0003149 | CUCCAGAGGGAAGUACUUUCU | 1.1099 | 1.1509 | 1.2039 | 0.7766 | 0.911 | 1.7384 |
| hsa-miR-520b | MI0003155 | AAAGUGCUUCCUUUUAGAGGG | 0.6687 | 0.5585 | 0.8544 | 0.9072 | 0.9681 | 1.1164 |
| hsa-miR-520c | MI0003158 | AAAGUGCUUCCUUUUAGAGGGU | 0.5084 | 0.4452 | 0.5192 | 0.6601 | 0.7753 | 0.7264 |
| hsa-miR-520c-5p | MI0003158 | CUCUAGAGGGAAGCACUUUCUG | 0.7326 | 0.5847 | 0.4486 | -0.0812 | 1.2598 | 0.6673 |
| hsa-miR-520d | MI0003164 | AAAGUGCUUCUCUUUGGUGGGU | 0.6433 | 0.6899 | 0.8354 | 0.599 | 0.7726 | 0.8739 |
| hsa-miR-520d* | MI0003164 | CUACAAAGGGAAGCCCUUUC | 0.8635 | 0.6045 | 4.1509 | 0.7025 | 1.0806 | 0.7104 |
| hsa-miR-520e | MI0003143 | AAAGUGCUUCCUUUUUGAGGG | 0.5826 | 0.653 | 0.8597 | 0.9861 | 0.6318 | 1.0633 |
| hsa-miR-520f | MI0003146 | AAGUGCUUCCUUUUAGAGGGUU | 0.2673 | 0.1638 | 0.4859 | 0.5668 | 0.4528 | 0.7328 |
| hsa-miR-520g | MI0003166 | ACAAAGUGCUUCCCUUUAGAGUGU | 0.8088 | 0.7973 | 1.177 | 0.8116 | 0.3742 | 0.5935 |
| hsa-miR-520h | MI0003175 | ACAAAGUGCUUCCCUUUAGAGU | 0.7729 | 0.5939 | 1.0851 | 0.917 | 0.6071 | 0.7926 |
| hsa-miR-521 | MI0003163 | AACGCACUUCCCUUUAGAGUGU | 0.7984 | 0.8621 | 1.133 | 0.3143 | 0.4801 | 0.8925 |
| hsa-miR-521 | MI0003176 | AACGCACUUCCCUUUAGAGUGU | 0.8204 | 1.1316 | 1.133 | 0.9609 | 0.5389 | 0.9528 |
| hsa-miR-522 | MI0003177 | AAAAUGGUUCCCUUUAGAGUGU | 0.4052 | 0.3067 | 0.5097 | 0.5952 | 0.4242 | 0.5435 |
| hsa-miR-522* | MI0003177 | CUCUAGAGGGAAGCGCUUUCUG | 0.5736 | 0.3193 | 0.5517 | 1.2761 | 0.538 | 0.5021 |
| hsa-miR-523 | MI0003153 | GAACGCGCUUCCCUAUAGAGGGU | 1.0289 | 1.1533 | 0.9044 | 0.6825 | 0.5207 | 0.9701 |
| hsa-miR-523* | MI0003153 | CUCUAGAGGGAAGCGCUUUCUG | 0.5353 | 0.2928 | 0.6687 | 0.5072 | 0.8483 | 0.5793 |
| hsa-miR-524 | MI0003160 | GAAGGCGCUUCCCUUUGGAGU | 0.8296 | 1.5723 | 1.1422 | 1.2141 | 0.8787 | 1.2628 |
| hsa-miR-524* | MI0003160 | CUACAAAGGGAAGCACUUUCUC | 0.6343 | 0.9047 | 0.8257 | 0.9614 | 0.9583 | 0.796 |
| hsa-miR-525 | MI0003152 | CUCCAGAGGGAUGCACUUUCU | 0.5353 | 0.5895 | 0.743 | 1.0079 | 0.8777 | 1.3692 |
| hsa-miR-525* | MI0003152 | GAAGGCGCUUCCCUUUAGAGCG | 1.4181 | 2.2961 | 0.8341 | 0.7504 | 0.8072 | 1.1132 |
| hsa-miR-526a | MI0003157 | CUCUAGAGGGAAGCACUUUCUG | 0.4164 | 0.3767 | 0.7962 | 0.6732 | 0.2724 | 0.3188 |
| hsa-miR-526a | MI0003168 | CUCUAGAGGGAAGCACUUUCUG | 0.9223 | 0.7757 | 1.0151 | 0.7325 | 0.4267 | 0.3505 |
| hsa-miR-526b | MI0003150 | CUCUUGAGGGAAGCACUUUCUGU | 1.3762 | 1.6432 | 0.8074 | 0.8844 | 0.3747 | 0.8227 |
| hsa-miR-526b* | MI0003150 | GAAAGUGCUUCCUUUUAGAGGC | 0.7409 | 1.1106 | 0.7867 | 0.7998 | 0.8413 | 0.9501 |
| hsa-miR-526c | MI0003148 | CUCUAGAGGGAAGCGCUUUCUG | 0.6759 | 0.7953 | 0.8913 | 0.7086 | 0.7216 | 0.4976 |
| hsa-miR-527 | MI0003179 | CUGCAAAGGGAAGCCCUUUC | 0.7424 | 1.8722 | 1.0187 | 0.9239 | 0.5949 | 0.5533 |
| hsa-miR-532 | MI0003205 | CAUGCCUUGAGUGUAGGACCGU | 0.8145 | 0.9978 | 1.6617 | 1.9121 | 0.781 | 1.1673 |
| hsa-miR-532-3p | MI0003205 | CCUCCCACACCCAAGGCUUGCA | 0.9597 | 0.4815 | 0.544 | 0.5428 | 1.0959 | 1.9786 |
| hsa-miR-539 | MI0003514 | GGAGAAAUUAUCCUUGGUGUGU | 0.3099 | 0.5893 | 0.9215 | 1.2411 | 0.3389 | 0.4894 |
| hsa-miR-541 | MI0005539 | UGGUGGGCACAGAAUCUGGACU | 0.324 | 0.5679 | 0.2712 | 0.4934 | 0.1956 | 0.3657 |
| hsa-miR-541* | MI0005539 | AAAGGAUUCUGCUGUCGGUCCCACU | 0.6262 | 0.9438 | 0.7089 | 1.115 | 1.0157 | 0.9664 |
| hsa-miR-542-3p | MI0003686 | UGUGACAGAUUGAUAACUGAAA | 0.7924 | 0.7313 | 1.0147 | 0.9923 | 1.3392 | 1.0987 |
| hsa-miR-542-5p | MI0003686 | UCGGGGAUCAUCAUGUCACGAGA | 1.4805 | 2.1348 | 1.7417 | 1.5573 | 0.531 | 0.8938 |
| hsa-miR-543 | MI0005565 | AAACAUUCGCGGUGCACUUCUU | 0.7256 | 1.4799 | 0.7137 | 0.8804 | 0.4116 | 0.4939 |
| hsa-miR-544 | MI0003515 | AUUCUGCAUUUUUAGCAAGUUC | 0.2997 | 1.4281 | 0.5072 | 0.5174 | 0.7367 | 0.678 |
| hsa-miR-545 | MI0003516 | UCAGCAAACAUUUAUUGUGUGC | 0.8781 | 1.5167 | 1.2495 | 0.7014 | 0.7923 | 1.2307 |
| hsa-miR-545* | MI0003516 | UCAGUAAAUGUUUAUUAGAUGA | 0.5611 | 1.3852 | 0.7679 | 1.0286 | 0.3241 | 0.4505 |
| hsa-miR-548a | MI0003593 | CAAAACUGGCAAUUACUUUUGC | 0.6803 | 0.7069 | 0.874 | 0.606 | 0.8934 | 1.2483 |
| hsa-miR-548a | MI0003598 | CAAAACUGGCAAUUACUUUUGC | 0.6995 | 1.181 | 1.0633 | 0.6553 | 0.9842 | 1.3431 |
| hsa-miR-548a | MI0003612 | CAAAACUGGCAAUUACUUUUGC | 0.9342 | 1.5704 | 1.2282 | 1.0682 | 1.1276 | 1.623 |
| hsa-miR-548a-5p | MI0003612 | AAAAGUAAUUGCGAGUUUUACC | 0.7918 | 1.2758 | 0.8144 | 1.1214 | 0.974 | 0.8719 |
| hsa-miR-548b | MI0003596 | CAAGAACCUCAGUUGCUUUUGU | 0.6428 | 0.7597 | 0.9283 | 0.5327 | 1.1889 | 1.5385 |
| hsa-miR-548b-5p | MI0003596 | AAAAGUAAUUGUGGUUUUGGCC | 1.0949 | 0.8226 | 0.5853 | 1.1226 | 1.1994 | 0.8473 |
| hsa-miR-548c | MI0003630 | CAAAAAUCUCAAUUACUUUUGC | 1.0625 | 0.7929 | 1.0891 | 0.9731 | 0.7856 | 1.0422 |
| hsa-miR-548c-5p | MI0003630 | AAAAGUAAUUGCGGUUUUUGCC | 0.8026 | 1.4955 | 0.7452 | 1.3815 | 0.9793 | 0.973 |
| hsa-miR-548d | MI0003668 | CAAAAACCACAGUUUCUUUUGC | 1.1184 | 0.759 | 0.6651 | 0.6991 | 0.4625 | 0.4545 |
| hsa-miR-548d | MI0003671 | CAAAAACCACAGUUUCUUUUGC | 0 | 2.2265 | 0.6917 | 0.8369 | 0.6775 | 0.8499 |
| hsa-miR-548d-5p | MI0003668 | AAAAGUAAUUGUGGUUUUUGCC | 0.5743 | 0.9488 | 0.5405 | 0.7726 | 0.7247 | 0.7335 |
| hsa-miR-548d-5p | MI0003671 | AAAAGUAAUUGUGGUUUUUGCC | 1.1136 | 1.0712 | 0.6915 | 0.7741 | 0.8475 | 1.0262 |
| hsa-miR-549 | MI0003679 | UGACAACUAUGGAUGAGCUCU | 0.0187 | 0.6451 | 1.0485 | 0.836 | 0.3808 | 0.4571 |
| hsa-miR-550 | MI0003600 | UGUCUUACUCCCUCAGGCACAU | 0.2729 | 0.2966 | 0.6385 | 0.3109 | 1.0583 | 0.8563 |
| hsa-miR-550 | MI0003601 | UGUCUUACUCCCUCAGGCACAU | 0.2866 | 0.874 | 0.6628 | 0.4899 | 1.1282 | 1.3241 |
| hsa-miR-550 | MI0003600 | AGUGCCUGAGGGAGUAAGAGCCC | 1.0953 | 0.9007 | 0.7244 | 0.6863 | 1.2503 | 1.3859 |
| hsa-miR-550 | MI0003601 | AGUGCCUGAGGGAGUAAGAGCCC | 1.1197 | 1.6545 | 1.0598 | 2.1809 | 1.3601 | 1.5216 |
| hsa-miR-551a | MI0003556 | GCGACCCACUCUUGGUUUCCA | 0.9632 | 1.098 | 1.1046 | 1.2187 | 0.8942 | 1.1088 |
| hsa-miR-551b | MI0003575 | GCGACCCAUACUUGGUUUCAG | 1.0058 | 0.8293 | 1.4249 | 1.3778 | 0.6199 | 1.0583 |
| hsa-miR-551b* | MI0003575 | GAAAUCAAGCGUGGGUGAGACC | 0.8547 | 0.7573 | 0.7205 | 1.2245 | 0.5531 | 0.74 |
| hsa-miR-552 | MI0003557 | AACAGGUGACUGGUUAGACAA | 1.3833 | 1.8443 | 2.581 | 3.0731 | 0.9615 | 1.2624 |
| hsa-miR-553 | MI0003558 | AAAACGGUGAGAUUUUGUUUU | 1.1705 | 2.1842 | 1.5306 | 1.4689 | 0.6909 | 1.0103 |
| hsa-miR-554 | MI0003559 | GCUAGUCCUGACUCAGCCAGU | 0.7104 | 1.2123 | 0.6989 | 1.0275 | 0.9232 | 1.1453 |
| hsa-miR-555 | MI0003561 | AGGGUAAGCUGAACCUCUGAU | 1.3506 | 2.3232 | 1.1219 | 2.0175 | 0.4614 | 1.4642 |
| hsa-miR-556 | MI0003562 | GAUGAGCUCAUUGUAAUAUGAG | 1.6505 | 2.9792 | 0.8294 | 0.6711 | 0.9704 | 1.3451 |
| hsa-miR-556-3p | MI0003562 | AUAUUACCAUUAGCUCAUCUUU | 1.0329 | 1.3382 | 0.5481 | 0.8684 | 0.6359 | 0.4787 |
| hsa-miR-557 | MI0003563 | GUUUGCACGGGUGGGCCUUGUCU | 1.5119 | 2.0594 | 0.2157 | 0.3786 | 0.7308 | 0.7004 |
| hsa-miR-558 | MI0003564 | UGAGCUGCUGUACCAAAAU | 0.3363 | 0.9335 | 0.9561 | 1.5769 | 0.4676 | 1.4332 |
| hsa-miR-559 | MI0003565 | UAAAGUAAAUAUGCACCAAAA | 1.7565 | 1.3094 | 0.6572 | 0.8421 | 0.696 | 0.9847 |
| hsa-miR-561 | MI0003567 | CAAAGUUUAAGAUCCUUGAAGU | 1.9034 | 0.104 | 1.1465 | 1.5155 | 0.7502 | 0.4939 |
| hsa-miR-562 | MI0003568 | AAAGUAGCUGUACCAUUUGC | 0.926 | 1.1439 | 0.6253 | 0.9031 | 0.6692 | 1.023 |
| hsa-miR-563 | MI0003569 | AGGUUGACAUACGUUUCCC | 1.2594 | 1.2374 | 0.9093 | 1.2179 | 0.5837 | 0.9391 |
| hsa-miR-564 | MI0003570 | AGGCACGGUGUCAGCAGGC | 0.9964 | 1.1483 | 1.0702 | 1.2991 | 0.7526 | 1.0774 |
| hsa-miR-566 | MI0003572 | GGGCGCCUGUGAUCCCAAC | 1.1547 | 1.1021 | 0.9898 | 1.585 | 0.71 | 1.0693 |
| hsa-miR-567 | MI0003573 | AGUAUGUUCUUCCAGGACAGAAC | 0.8597 | 0.6578 | 0.4809 | 0.7883 | 0.4003 | 0.6823 |
| hsa-miR-568 | MI0003574 | AUGUAUAAAUGUAUACACAC | 1.1547 | 0.9573 | 1.2292 | 1.2044 | 0.6998 | 1.0884 |
| hsa-miR-569 | MI0003576 | AGUUAAUGAAUCCUGGAAAGU | 0.956 | 1.1179 | 1.2737 | 1.1443 | 0.6738 | 0.3627 |
| hsa-miR-570 | MI0003577 | CGAAAACAGCAAUUACCUUUGC | 1.3797 | 1.5931 | 0.5927 | 0.8413 | 1.0793 | 1.4654 |
| hsa-miR-571 | MI0003578 | UGAGUUGGCCAUCUGAGUGAG | 1.0159 | 1.0764 | 0.6699 | 1.1434 | 0.7138 | 1.1106 |
| hsa-miR-572 | MI0003579 | GUCCGCUCGGCGGUGGCCCA | 0.7961 | 1.3973 | 1.0771 | 1.165 | 0.649 | 0.8814 |
| hsa-miR-573 | MI0003580 | CUGAAGUGAUGUGUAACUGAUCAG | 1.1488 | 0.6914 | 0.8997 | 0.956 | 1.0004 | 0.9996 |
| hsa-miR-574 | MI0003581 | CACGCUCAUGCACACACCCACA | 1.0255 | 1.9264 | 0.5346 | 0.8064 | 0.8542 | 1.3828 |
| hsa-miR-574-5p | MI0003581 | UGAGUGUGUGUGUGUGAGUGUGU | 0.9782 | -0.0185 | 0.5227 | 0.4667 | 0.6588 | 0.7074 |
| hsa-miR-575 | MI0003582 | GAGCCAGUUGGACAGGAGC | 0.4328 | 0.0096 | 1.2848 | 1.2683 | 0.9615 | 1.0885 |
| hsa-miR-576 | MI0003583 | AUUCUAAUUUCUCCACGUCUUU | 0.8914 | 1.2921 | 0.4659 | 0.7884 | 0.8297 | 1.2075 |
| hsa-miR-576-3p | MI0003583 | AAGAUGUGGAAAAAUUGGAAUC | 0.6664 | 0.8073 | 0.4996 | 0.5947 | 0.5302 | 0.7285 |
| hsa-miR-577 | MI0003584 | UAGAUAAAAUAUUGGUACCUG | 0.726 | 0.6829 | 0.9927 | 0.783 | 0.8982 | 0.5977 |
| hsa-miR-578 | MI0003585 | CUUCUUGUGCUCUAGGAUUGU | 0.8496 | 0.5119 | 0.9991 | 0.8389 | 0.8349 | 0.8669 |
| hsa-miR-579 | MI0003586 | UUCAUUUGGUAUAAACCGCGAUU | 0.6928 | 1.1438 | 0.6055 | 0.6742 | 0.5282 | 0.5694 |
| hsa-miR-580 | MI0003587 | UUGAGAAUGAUGAAUCAUUAGG | 0.8096 | 0.7775 | 0.7771 | 0.6725 | 0.9646 | 1.5306 |
| hsa-miR-581 | MI0003588 | UCUUGUGUUCUCUAGAUCAGU | 0.8697 | 0.5739 | 1.0848 | 1.0099 | 1.9537 | 1.5713 |
| hsa-miR-582 | MI0003589 | UUACAGUUGUUCAACCAGUUACU | 0.4938 | 0.6376 | 0.8304 | 0.5829 | 0.7474 | 0.5327 |
| hsa-miR-582-3p | MI0003589 | UAACUGGUUGAACAACUGAACC | 0.798 | 0.8796 | 0.675 | 1.5345 | 0.6069 | 0.8592 |
| hsa-miR-583 | MI0003590 | CAAAGAGGAAGGUCCCAUUAC | 0.3828 | 0.4694 | 0.9477 | 0.6864 | 0.7981 | 0.5306 |
| hsa-miR-584 | MI0003591 | UUAUGGUUUGCCUGGGACUGAG | 0.8106 | 1.0474 | 0.5427 | 0.5034 | 0.926 | 1.0852 |
| hsa-miR-585 | MI0003592 | UGGGCGUAUCUGUAUGCUA | 0.6337 | 0.7435 | 1.0612 | 0.8034 | 0.8083 | 1.3879 |
| hsa-miR-586 | MI0003594 | UAUGCAUUGUAUUUUUAGGUCC | 0.5844 | 1.2704 | 1.1893 | 0.727 | 1.3228 | 1.1379 |
| hsa-miR-587 | MI0003595 | UUUCCAUAGGUGAUGAGUCAC | 0.6989 | 0.7454 | 0.4015 | 0.7053 | 0.984 | 0.8962 |
| hsa-miR-588 | MI0003597 | UUGGCCACAAUGGGUUAGAAC | 1.0626 | 1.0188 | 0.8713 | 0.6798 | 0.901 | 0.9939 |
| hsa-miR-589 | MI0003599 | UGAGAACCACGUCUGCUCUGAG | 1.1018 | 0.8187 | 0.5415 | 0.8803 | 0.8233 | 0.8079 |
| hsa-miR-589* | MI0003599 | UCAGAACAAAUGCCGGUUCCCAGA | 1.102 | 1.0203 | 0.9998 | 0.7493 | 1.0745 | 1.1402 |
| hsa-miR-590 | MI0003602 | GAGCUUAUUCAUAAAAGUGCAG | 4.1597 | 2.491 | 1.1835 | 0.6536 | 0.9009 | 1.0432 |
| hsa-miR-590-3p | MI0003602 | UAAUUUUAUGUAUAAGCUAGU | -11.5418 | -30.4758 | 0.4321 | 0.5484 | 0.6425 | 0.7548 |
| hsa-miR-591 | MI0003603 | AGACCAUGGGUUCUCAUUGU | 9.7972 | -1.8175 | 0.6919 | 0.4686 | 0.7943 | 0.7399 |
| hsa-miR-592 | MI0003604 | UUGUGUCAAUAUGCGAUGAUGU | 0.6431 | 1.0789 | 0.7687 | 0.3868 | 0.983 | 0.9436 |
| hsa-miR-593 | MI0003605 | UGUCUCUGCUGGGGUUUCU | 1.0552 | 1.6929 | 0.713 | 2.7238 | 0.5669 | 1.1594 |
| hsa-miR-593* | MI0003605 | AGGCACCAGCCAGGCAUUGCUCAGC | 1.1933 | 1.4784 | 0.6041 | 0.6698 | 0.7771 | 0.705 |
| hsa-miR-595 | MI0003607 | GAAGUGUGCCGUGGUGUGUCU | 0.7867 | 0.6033 | 0.8481 | 0.9668 | 1.0111 | 0.8157 |
| hsa-miR-596 | MI0003608 | AAGCCUGCCCGGCUCCUCGGG | 0.669 | 0.9307 | 0.5481 | 0.7153 | 0.7467 | 0.9032 |
| hsa-miR-597 | MI0003609 | UGUGUCACUCGAUGACCACUGU | 0.9349 | 1.0753 | 0.8749 | 1.125 | 0.7833 | 0.7854 |
| hsa-miR-598 | MI0003610 | UACGUCAUCGUUGUCAUCGUCA | 1.0075 | 1.6027 | 0.8602 | 0.9289 | 0.8073 | 0.8691 |
| hsa-miR-599 | MI0003611 | GUUGUGUCAGUUUAUCAAAC | 0.9233 | 1.109 | 1.3017 | 1.0474 | 0.7304 | 0.7311 |
| hsa-miR-600 | MI0003613 | ACUUACAGACAAGAGCCUUGCUC | 0.9655 | 1.0515 | 1.2711 | 1.1273 | 0.5988 | 0.526 |
| hsa-miR-601 | MI0003614 | UGGUCUAGGAUUGUUGGAGGAG | 1.0694 | 1.1431 | 0.5112 | 0.8697 | 0.8496 | 1.2554 |
| hsa-miR-602 | MI0003615 | GACACGGGCGACAGCUGCGGCCC | 0.9722 | 0.7491 | 0.9896 | 0.7646 | 0.5156 | 0.8414 |
| hsa-miR-603 | MI0003616 | CACACACUGCAAUUACUUUUGC | 0.8857 | 0.8616 | 0.8379 | 0.8427 | 0.699 | 0.9602 |
| hsa-miR-604 | MI0003617 | AGGCUGCGGAAUUCAGGAC | 0.7193 | 0.8803 | 0.8882 | 0.9774 | 0.3784 | 0.7916 |
| hsa-miR-605 | MI0003618 | UAAAUCCCAUGGUGCCUUCUCCU | 0.8411 | 0.8046 | 0.7204 | 1.0051 | 0.738 | 0.7258 |
| hsa-miR-606 | MI0003619 | AAACUACUGAAAAUCAAAGAU | 0.8446 | 0.8183 | 0.9574 | 0.6312 | 0.508 | 0.9623 |
| hsa-miR-607 | MI0003620 | GUUCAAAUCCAGAUCUAUAAC | 0.7164 | 1.094 | 0.9846 | 0.7097 | 0.6599 | 0.6733 |
| hsa-miR-608 | MI0003621 | AGGGGUGGUGUUGGGACAGCUCCGU | 0.46 | 0.8326 | 0.8208 | 0.7446 | 0.5182 | 0.9579 |
| hsa-miR-609 | MI0003622 | AGGGUGUUUCUCUCAUCUCU | 0.8649 | 1.5451 | 0.7492 | 0.8007 | 0.6602 | 1.2428 |
| hsa-miR-610 | MI0003623 | UGAGCUAAAUGUGUGCUGGGA | 1.5302 | 1.3381 | 0.2952 | 0.8102 | 0.7003 | 0.7147 |
| hsa-miR-611 | MI0003624 | GCGAGGACCCCUCGGGGUCUGAC | 2.2805 | 4.0514 | 0.5456 | 1.0548 | 0.6126 | 0.7869 |
| hsa-miR-612 | MI0003625 | GCUGGGCAGGGCUUCUGAGCUCCUU | 1.3208 | 1.2231 | 0.6657 | 0.7584 | 0.7186 | 1.0439 |
| hsa-miR-613 | MI0003626 | AGGAAUGUUCCUUCUUUGCC | 1.1729 | 2.2034 | 0.5681 | 0.6466 | 0.4317 | 0.7991 |
| hsa-miR-614 | MI0003627 | GAACGCCUGUUCUUGCCAGGUGG | 0.8498 | 0.8499 | 0.4381 | 0.5866 | 0.7032 | 1.0711 |
| hsa-miR-615 | MI0003628 | UCCGAGCCUGGGUCUCCCUCUU | 0.868 | 1.2035 | 0.4199 | 0.59 | 0.7458 | 0.9311 |
| hsa-miR-615-5p | MI0003628 | GGGGGUCCCCGGUGCUCGGAUC | 1.066 | 1.7729 | 0.6162 | 0.9664 | 1.032 | 0.7852 |
| hsa-miR-616 | MI0003629 | AGUCAUUGGAGGGUUUGAGCAG | 1.27 | 1.0728 | 0.7155 | 0.6657 | 0.6235 | 0.9706 |
| hsa-miR-616* | MI0003629 | ACUCAAAACCCUUCAGUGACUU | 0.9796 | 0.8518 | 1.2216 | 1.2527 | 0.4764 | 0.8855 |
| hsa-miR-617 | MI0003631 | AGACUUCCCAUUUGAAGGUGGC | 0.8207 | 0.9226 | 1.1649 | 1.181 | 0.4517 | 1.3394 |
| hsa-miR-618 | MI0003632 | AAACUCUACUUGUCCUUCUGAGU | 0.9255 | 0.6993 | 0.861 | 0.9495 | 0.6205 | 0.8312 |
| hsa-miR-619 | MI0003633 | GACCUGGACAUGUUUGUGCCCAGU | 0.8709 | 0.6252 | 0.9119 | 0.9272 | 0.5414 | 0.843 |
| hsa-miR-620 | MI0003634 | AUGGAGAUAGAUAUAGAAAU | 0.7529 | 0.6918 | 1.0897 | 1.0176 | 0.419 | 0.6309 |
| hsa-miR-621 | MI0003635 | GGCUAGCAACAGCGCUUACCU | 0.8773 | 0.6269 | 1.1639 | 1.0224 | 0.6491 | 0.9499 |
| hsa-miR-622 | MI0003636 | ACAGUCUGCUGAGGUUGGAGC | 0.9433 | 0.8982 | 0.6993 | 0.6766 | 0.7723 | 1.0633 |
| hsa-miR-623 | MI0003637 | AUCCCUUGCAGGGGCUGUUGGGU | 0.8181 | 0.8004 | 0.9763 | 1.1619 | 0.5891 | 1.0207 |
| hsa-miR-624 | MI0003638 | CACAAGGUAUUGGUAUUACCU | 1.2926 | 1.2265 | 0.7422 | 1.439 | 1.2908 | 2.5144 |
| hsa-miR-624* | MI0003638 | UAGUACCAGUACCUUGUGUUCA | 0.7205 | 0.5803 | 0.7296 | 0.6551 | 0.4178 | 0.5452 |
| hsa-miR-625 | MI0003639 | AGGGGGAAAGUUCUAUAGUCC | 0.805 | 1.5733 | 0.506 | 0.6197 | 0.5342 | 0.7634 |
| hsa-miR-625* | MI0003639 | GACUAUAGAACUUUCCCCCUCA | 1.1573 | 1.5905 | 0.6488 | 1.1427 | 1.0881 | 0.7506 |
| hsa-miR-626 | MI0003640 | AGCUGUCUGAAAAUGUCUU | 0.6015 | 0.7041 | 0.9865 | 0.9452 | 0.6645 | 0.9154 |
| hsa-miR-627 | MI0003641 | GUGAGUCUCUAAGAAAAGAGGA | 0.7399 | 1.0435 | 0.8769 | 0.8477 | 0.6871 | 1.1088 |
| hsa-miR-628 | MI0003642 | UCUAGUAAGAGUGGCAGUCGA | 0.9767 | 1.2267 | 0.4606 | 0.7381 | 0.5485 | 0.8123 |
| hsa-miR-628-5p | MI0003642 | AUGCUGACAUAUUUACUAGAGG | 0.7826 | 0.4681 | 0.7173 | 0.8176 | 0.6522 | 0.4345 |
| hsa-miR-629 | MI0003643 | UGGGUUUACGUUGGGAGAACU | 0.5268 | 1.0126 | 0.2278 | 0.8978 | 0.7293 | 0.7955 |
| hsa-miR-629* | MI0003643 | GUUCUCCCAACGUAAGCCCAGC | 0.6596 | 1.0139 | 1.104 | 1.0524 | 0.4515 | 1.1582 |
| hsa-miR-630 | MI0003644 | AGUAUUCUGUACCAGGGAAGGU | 0.9182 | 1.0665 | 0.6083 | 0.4832 | 0.5972 | 0.7295 |
| hsa-miR-631 | MI0003645 | AGACCUGGCCCAGACCUCAGC | 0.6671 | 0.7641 | 0.993 | 0.9643 | 0.5399 | 0.7031 |
| hsa-miR-632 | MI0003647 | GUGUCUGCUUCCUGUGGGA | 0.6467 | 1.1361 | 0.7772 | 0.5416 | 0.8002 | 0.6967 |
| hsa-miR-633 | MI0003648 | CUAAUAGUAUCUACCACAAUAAA | 0.4052 | 0.7128 | 1.0494 | 0.6973 | 0.6234 | 0.9154 |
| hsa-miR-634 | MI0003649 | AACCAGCACCCCAACUUUGGAC | 0.4206 | 0.3636 | 0.341 | 0.3963 | 0.3118 | 0.4001 |
| hsa-miR-635 | MI0003650 | ACUUGGGCACUGAAACAAUGUCC | 0.1103 | 0.6389 | 0.513 | 0.3216 | 0.1984 | 0.3628 |
| hsa-miR-636 | MI0003651 | UGUGCUUGCUCGUCCCGCCCGCA | 0.9051 | 0.7179 | 0.4213 | 0.6442 | 0.8724 | 0.6703 |
| hsa-miR-637 | MI0003652 | ACUGGGGGCUUUCGGGCUCUGCGU | 0.6359 | 1.1843 | 0.7594 | 0.8295 | 0.296 | 0.2686 |
| hsa-miR-638 | MI0003653 | AGGGAUCGCGGGCGGGUGGCGGCCU | 0.8217 | 0.907 | 1.2731 | 0.9328 | 0.6813 | 1.128 |
| hsa-miR-639 | MI0003654 | AUCGCUGCGGUUGCGAGCGCUGU | 0.4345 | 0.6973 | 1.0301 | 0.8786 | 0.5725 | 0.8656 |
| hsa-miR-640 | MI0003655 | AUGAUCCAGGAACCUGCCUCU | 0.9205 | 0.5632 | 0.4615 | 0.5247 | 0.425 | 0.4473 |
| hsa-miR-641 | MI0003656 | AAAGACAUAGGAUAGAGUCACCUC | 0.8629 | 0.9165 | 0.8525 | 0.5158 | 0.6909 | 0.6333 |
| hsa-miR-642 | MI0003657 | GUCCCUCUCCAAAUGUGUCUUG | 0.9633 | 0.855 | 1.1817 | 0.6295 | 0.6319 | 0.5933 |
| hsa-miR-643 | MI0003658 | ACUUGUAUGCUAGCUCAGGUAG | 0.7809 | 0.62 | 1.0687 | 0.7748 | 0.6555 | 0.6022 |
| hsa-miR-644 | MI0003659 | AGUGUGGCUUUCUUAGAGC | 0.2434 | 0.5531 | 0.3263 | 0.5197 | 0.2563 | 0.5531 |
| hsa-miR-645 | MI0003660 | UCUAGGCUGGUACUGCUGA | 0.9381 | 0.6991 | 1.0215 | 0.6435 | 0.6687 | 0.6886 |
| hsa-miR-646 | MI0003661 | AAGCAGCUGCCUCUGAGGC | 0.531 | 0.5846 | 0.4512 | 0.6389 | 0.4413 | 0.5076 |
| hsa-miR-647 | MI0003662 | GUGGCUGCACUCACUUCCUUC | 0.2569 | 0.4894 | 0.5158 | 0.626 | 0.3848 | 0.5274 |
| hsa-miR-648 | MI0003663 | AAGUGUGCAGGGCACUGGU | 0.9919 | 0.7635 | 1.6215 | 1.1665 | 0.7428 | 0.9696 |
| hsa-miR-649 | MI0003664 | AAACCUGUGUUGUUCAAGAGUC | 0.3893 | 0.5333 | 1.0143 | 0.8151 | 0.4856 | 0.7951 |
| hsa-miR-650 | MI0003665 | AGGAGGCAGCGCUCUCAGGAC | 0.5315 | 0.6057 | 0.5005 | 0.4402 | 0.4466 | 0.5573 |
| hsa-miR-651 | MI0003666 | UUUAGGAUAAGCUUGACUUUUG | 0.3906 | 0.5538 | 0.8807 | 0.7232 | 0.4529 | 0.5089 |
| hsa-miR-652 | MI0003667 | AAUGGCGCCACUAGGGUUGUG | 1.0085 | 1.5543 | 0.3656 | 0.4708 | 0.6837 | 1.3401 |
| hsa-miR-653 | MI0003674 | GUGUUGAAACAAUCUCUACUG | 0.6964 | 0.918 | 0.6097 | 0.6692 | 0.6737 | 0.7948 |
| hsa-miR-654 | MI0003676 | UGGUGGGCCGCAGAACAUGUGC | 0.4129 | 0.5114 | 0.8285 | 0.7623 | 0.3559 | 0.1755 |
| hsa-miR-654-3p | MI0003676 | UAUGUCUGCUGACCAUCACCUU | 0.9099 | 0.8248 | 0.7201 | 2.142 | 1.7435 | 2.1779 |
| hsa-miR-655 | MI0003677 | AUAAUACAUGGUUAACCUCUUU | 0.3979 | 0.5066 | 0.745 | 0.5851 | 0.4686 | 0.7311 |
| hsa-miR-656 | MI0003678 | AAUAUUAUACAGUCAACCUCU | 0.5571 | 0.7642 | 1.1295 | 1.0083 | 0.5209 | 0.6585 |
| hsa-miR-657 | MI0003681 | GGCAGGUUCUCACCCUCUCUAGG | 0.4447 | 0.382 | 0.921 | 0.6671 | 0.5464 | 0.7527 |
| hsa-miR-658 | MI0003682 | GGCGGAGGGAAGUAGGUCCGUUGGU | 0.4219 | 0.3623 | 0.7473 | 0.6122 | 0.383 | 0.7314 |
| hsa-miR-659 | MI0003683 | CUUGGUUCAGGGAGGGUCCCCA | 0.3753 | 0.5075 | 0.9281 | 0.7744 | 0.6644 | 0.8154 |
| hsa-miR-660 | MI0003684 | UACCCAUUGCAUAUCGGAGUUG | 0.3655 | 0.5012 | 0.8739 | 0.589 | 0.3977 | 0.5418 |
| hsa-miR-661 | MI0003669 | UGCCUGGGUCUCUGGCCUGCGCGU | 0.5026 | 0.5695 | 0.7818 | 1.038 | 0.5123 | 0.641 |
| hsa-miR-662 | MI0003670 | UCCCACGUUGUGGCCCAGCAG | 0.8673 | 0.7764 | 0.8933 | 0.7702 | 0.2932 | 0.2888 |
| hsa-miR-663 | MI0003672 | AGGCGGGGCGCCGCGGGACCGC | 0.4986 | 0.8397 | 0.8013 | 0.7425 | 0.3516 | 0.2821 |
| hsa-miR-665 | MI0005563 | ACCAGGAGGCUGAGGCCCCU | 0.9862 | 2.5853 | 0.6078 | 0.887 | 0.6762 | 1.1922 |
| hsa-miR-668 | MI0003761 | UGUCACUCGGCUCGGCCCACUAC | 3.0521 | 3.9547 | -10.2152 | 3.9776 | 0.7352 | 0.9278 |
| hsa-miR-671 | MI0003760 | AGGAAGCCCUGGAGGGGCUGGAG | 1.1316 | 1.6304 | 0.7426 | 0.7278 | 0.5909 | 0.6461 |
| hsa-miR-671-3p | MI0003760 | UCCGGUUCUCAGGGCUCCACC | 0.9663 | 2.2825 | 0.5391 | 0.9316 | 0.5405 | 0.7445 |
| hsa-miR-675 | MI0005416 | UGGUGCGGAGAGGGCCCACAGUG | 1.4581 | 1.203 | 0.8188 | 1.1618 | 1.1162 | 1.1265 |
| hsa-miR-7 | MI0000264 | UGGAAGACUAGUGAUUUUGUUGU | 0.3365 | 1.3531 | 0.3216 | 0.6437 | 0.3937 | 0.4914 |
| hsa-miR-7 | MI0000265 | UGGAAGACUAGUGAUUUUGUUGU | 0.4703 | 1.5529 | 0.4496 | 0.7537 | 0.7981 | 1.2406 |
| hsa-miR-7 | MI0000263 | UGGAAGACUAGUGAUUUUGUUGU | 1.1625 | 2.3276 | 1.6392 | 1.8579 | 1.4266 | 1.4206 |
| hsa-miR-7-1* | MI0005543 | AAGGAGCUUACAAUCUAGCUGGG | 0.6252 | 0.8331 | 0.8125 | 2.0195 | 1.3822 | 1.5836 |
| hsa-miR-7-2* | MI0005543 | CAACUAGACUGUGAGCUUCUAG | 0.8985 | 1.0032 | 1.2183 | 0.7625 | 0.7017 | 1.2673 |
| hsa-miR-708 | MI0000263 | CAACAAAUCACAGUCUGCCAUA | 0.638 | 0.7857 | 0.6259 | 1.0875 | 0.4753 | 1.2124 |
| hsa-miR-708* | MI0000264 | CAACAAAUCCCAGUCUACCUAA | 0.7495 | 0.9884 | 0.646 | 1.2678 | 0.9009 | 0.8883 |
| hsa-miR-744 | MI0005559 | UGCGGGGCUAGGGCUAACAGCA | 0.7193 | 1.9372 | 0.6195 | 1.0571 | 0.6738 | 1.8027 |
| hsa-miR-744* | MI0005559 | CUGUUGCCACUAACCUCAACCU | 0.5718 | 2.0657 | 0.8279 | 1.1165 | 0.4959 | 0.5022 |
| hsa-miR-758 | MI0003757 | UUUGUGACCUGGUCCACUAACC | 0.8312 | 1.1045 | 0.6221 | 0.6005 | 1.3739 | 8.3073 |
| hsa-miR-760 | MI0005567 | CGGCUCUGGGUCUGUGGGGA | 0.5676 | 1 | 0.618 | 1.095 | 0.8716 | 0.9931 |
| hsa-miR-765 | MI0005116 | UGGAGGAGAAGGAAGGUGAUG | 0.4789 | 0.5821 | 0.4973 | 0.5846 | 0.3888 | 0.4466 |
| hsa-miR-766 | MI0003836 | ACUCCAGCCCCACAGCCUCAGC | 1.2505 | 1.8852 | 0.9373 | 1.1493 | 0.3266 | 0.505 |
| hsa-miR-767-3p | MI0003763 | UCUGCUCAUACCCCAUGGUUUCU | 0.4711 | 0.8434 | 0.5695 | 0.4677 | 0.508 | 0.5767 |
| hsa-miR-767-5p | MI0003763 | UGCACCAUGGUUGUCUGAGCAUG | 5.3755 | 8.0933 | 0.7228 | 0.6328 | 1.5207 | 4.4065 |
| hsa-miR-768-3p | MI0005117 | UCACAAUGCUGACACUCAAACUGCUGAC | 1.0302 | 1.1918 | 0.7896 | 0.6823 | 1.5137 | 1.1243 |
| hsa-miR-768-5p | MI0005117 | GUUGGAGGAUGAAAGUACGGAGUGAU | 1.8046 | 3.3005 | 0.6539 | 0.6166 | 0.7048 | 1.1047 |
| hsa-miR-769-3p | MI0003834 | CUGGGAUCUCCGGGGUCUUGGUU | 0.4051 | 0.6357 | 0.529 | 0.4699 | 0.6483 | 0.9582 |
| hsa-miR-769-5p | MI0003834 | UGAGACCUCUGGGUUCUGAGCU | 0.459 | 0.5354 | -12.5007 | 3.3262 | 1.5941 | 2.6387 |
| hsa-miR-770-5p | MI0005118 | UCCAGUACCACGUGUCAGGGCCA | 0.7863 | 0.9214 | 1.0305 | 1.1152 | 0.9464 | 0.9554 |
| hsa-miR-801 | MI0005202 | GAUUGCUCUGCGUGCGGAAUCGAC | 1.4021 | 2.5541 | 0.9949 | 0.8668 | 0.8744 | 1.8331 |
| hsa-miR-802 | MI0003906 | CAGUAACAAAGAUUCAUCCUUGU | 1.1662 | 2.4157 | 0.8236 | 0.7612 | 0.4056 | 0.6168 |
| hsa-miR-873 | MI0005564 | GCAGGAACUUGUGAGUCUCCU | 0.4318 | 0.5747 | 0.5229 | 0.5257 | 0.3259 | 0.5031 |
| hsa-miR-874 | MI0005532 | CUGCCCUGGCCCGAGGGACCGA | 1.2107 | 1.5919 | 0.6957 | 1.2636 | 0.529 | 0.4005 |
| hsa-miR-875-3p | MI0005541 | CCUGGAAACACUGAGGUUGUG | 0.816 | 1.137 | 0.6379 | 1.0565 | 0.6121 | 0.7978 |
| hsa-miR-875-5p | MI0005541 | UAUACCUCAGUUUUAUCAGGUG | 0.753 | 1.1426 | 0.7721 | 1.1088 | 0.9531 | 0.9795 |
| hsa-miR-876-3p | MI0005542 | UGGUGGUUUACAAAGUAAUUCA | 0.6904 | 1.8424 | 0.8115 | 1.4255 | 0.5981 | 1.0758 |
| hsa-miR-876-5p | MI0005542 | UGGAUUUCUUUGUGAAUCACCA | 0.7062 | 1.1368 | 0.7394 | 1.243 | 0.7187 | 0.6522 |
| hsa-miR-877 | MI0005561 | GUAGAGGAGAUGGCGCAGGG | 0.8934 | 0.914 | 1.0602 | 0.6586 | 0.9173 | 1.2899 |
| hsa-miR-877* | MI0005561 | UCCUCUUCUCCCUCCUCCCAG | 1.2836 | 1.3263 | 1.6822 | 0.7215 | 0.7713 | 1.2394 |
| hsa-miR-885-3p | MI0005560 | AGGCAGCGGGGUGUAGUGGAUA | 0.8842 | 2.5166 | 0.6017 | 1.1112 | 0.6986 | 0.7347 |
| hsa-miR-885-5p | MI0005560 | UCCAUUACACUACCCUGCCUCU | 0.7509 | 1.033 | 0.6038 | 0.9093 | 0.6253 | 0.446 |
| hsa-miR-886-3p | MI0005527 | CGCGGGUGCUUACUGACCCUU | 0.5246 | 1.4396 | 0.8347 | 1.2962 | 0.6629 | 3.2991 |
| hsa-miR-886-5p | MI0005527 | CGGGUCGGAGUUAGCUCAAGCGG | 0.7286 | -0.0107 | 0.5466 | 0.9299 | 0.6609 | 0.4606 |
| hsa-miR-887 | MI0005562 | GUGAACGGGCGCCAUCCCGAGG | 0.9219 | 0.631 | 0.7771 | 1.1979 | 0.7741 | 0.5645 |
| hsa-miR-888 | MI0005537 | UACUCAAAAAGCUGUCAGUCA | 0.4938 | 0.7993 | 0.6944 | 1.1383 | 0.5351 | 0.7304 |
| hsa-miR-888* | MI0005537 | GACUGACACCUCUUUGGGUGAA | 0.5682 | 0.7849 | 0.7802 | 1.2436 | 0.614 | 0.7652 |
| hsa-miR-889 | MI0005540 | UUAAUAUCGGACAACCAUUGU | 0.7059 | 0.7097 | 0.6278 | 1.0282 | 0.7104 | 0.4966 |
| hsa-miR-890 | MI0005533 | UACUUGGAAAGGCAUCAGUUG | 0.6248 | 0.7812 | 0.6852 | 0.9585 | 0.5395 | 0.4778 |
| hsa-miR-891a | MI0005524 | UGCAACGAACCUGAGCCACUGA | 0.6287 | 1.2755 | 0.5861 | 0.8655 | 0.8935 | 0.8621 |
| hsa-miR-891b | MI0005534 | UGCAACUUACCUGAGUCAUUGA | 0.7772 | 1.206 | 0.6144 | 1.0355 | 0.9409 | 0.9691 |
| hsa-miR-892a | MI0005528 | CACUGUGUCCUUUCUGCGUAG | 0.3989 | 0.7439 | 0.5522 | 0.7213 | 0.4755 | 0.3722 |
| hsa-miR-892b | MI0005538 | CACUGGCUCCUUUCUGGGUAGA | 0.6351 | 1.2547 | 0.5823 | 1.3373 | 0.6645 | 1.1816 |
| hsa-miR-9 | MI0000467 | UCUUUGGUUAUCUAGCUGUAUGA | 0.0237 | 0.6819 | 0.0682 | 0.3078 | 0.4601 | 0.276 |
| hsa-miR-9 | MI0000468 | UCUUUGGUUAUCUAGCUGUAUGA | 0.6017 | 0.5156 | 0.6869 | 0.6775 | 0.9128 | 0.8328 |
| hsa-miR-9 | MI0000466 | UCUUUGGUUAUCUAGCUGUAUGA | 0.625 | 0.8506 | 1.492 | 1.0416 | 1.4667 | 0.9819 |
| hsa-miR-9* | MI0000467 | AUAAAGCUAGAUAACCGAAAGU | 0.6837 | 0.4078 | 0.4586 | 0.5262 | 0.3964 | 0.4871 |
| hsa-miR-9* | MI0000468 | AUAAAGCUAGAUAACCGAAAGU | 0.7053 | 0.7476 | 0.7002 | 0.8746 | 0.4784 | 0.5819 |
| hsa-miR-9* | MI0000466 | AUAAAGCUAGAUAACCGAAAGU | 0.5123 | 0.4885 | 1.8572 | 0.9573 | 0.5738 | 0.713 |
| hsa-miR-92 | MI0000093 | UAUUGCACUUGUCCCGGCCUGU | 0.8987 | 1.1135 | 0.5227 | 0.6437 | 0.7047 | 0.5615 |
| hsa-miR-92 | MI0000094 | UAUUGCACUUGUCCCGGCCUGU | 1.1003 | 1.4573 | 0.7695 | 0.7331 | 0.8138 | 1.1814 |
| hsa-miR-920 | MI0005712 | GGGGAGCUGUGGAAGCAGUA | 0.9976 | 1.3688 | 0.8227 | 0.2991 | 0.5931 | 0.6309 |
| hsa-miR-921 | MI0005713 | CUAGUGAGGGACAGAACCAGGAUUC | 0.8915 | 1.0892 | 1.8798 | 1.0515 | 1.0187 | 1.9271 |
| hsa-miR-922 | MI0005714 | GCAGCAGAGAAUAGGACUACGUC | 1.3129 | 0.967 | 1.3852 | 0.962 | 1.3497 | 1.1396 |
| hsa-miR-923 | MI0005715 | GUCAGCGGAGGAAAAGAAACU | 0.809 | 1.0091 | 1.8035 | 1.0396 | 1.2517 | 1.1997 |
| hsa-miR-924 | MI0005716 | AGAGUCUUGUGAUGUCUUGC | 1.5347 | 1.4635 | 1.5541 | 0.6443 | 0.756 | 1.6394 |
| hsa-miR-92a-1* | MI0000093 | AGGUUGGGAUCGGUUGCAAUGCU | 0.6087 | 1.1016 | 0.4437 | 1.1604 | 0.5228 | 0.8763 |
| hsa-miR-92a-2* | MI0000094 | GGGUGGGGAUUUGUUGCAUUAC | 1.0035 | -0.0328 | 0.5379 | 0.6777 | 0.6609 | 0.6749 |
| hsa-miR-92b | MI0003560 | UAUUGCACUCGUCCCGGCCUCC | 0.9949 | 1.1468 | 0.5213 | 0.7885 | 0.6309 | 0.6919 |
| hsa-miR-92b* | MI0003560 | AGGGACGGGACGCGGUGCAGUG | 1.0027 | 1.0836 | 0.6088 | 0.9664 | 0.6053 | 0.5608 |
| hsa-miR-93 | MI0000095 | CAAAGUGCUGUUCGUGCAGGUAG | 0.7178 | 1.416 | 1.8447 | 0.8527 | 0.3835 | 0.616 |
| hsa-miR-93* | MI0000095 | ACUGCUGAGCUAGCACUUCCCG | 0.5928 | 0.5094 | 0.4423 | 0.6503 | 0.4255 | 0.3192 |
| hsa-miR-933 | MI0005755 | UGUGCGCAGGGAGACCUCUCCC | 0.8857 | 0.8649 | 2.255 | 0.9957 | 0.5116 | 2.1172 |
| hsa-miR-934 | MI0005756 | UGUCUACUACUGGAGACACUGG | 0.9002 | 0.8627 | 2.8517 | 0.6849 | 2.0086 | 2.1905 |
| hsa-miR-935 | MI0005757 | CCAGUUACCGCUUCCGCUACCGC | 0.8154 | 1.0265 | 1.4627 | 0.8758 | 1.7694 | 1.4542 |
| hsa-miR-936 | MI0005758 | ACAGUAGAGGGAGGAAUCGCAG | 0.8651 | 0.7265 | 1.7616 | 1.0792 | 1.54 | 0.7584 |
| hsa-miR-937 | MI0005759 | AUCCGCGCUCUGACUCUCUGCC | 0.9918 | 0.9104 | 2.8862 | 1.067 | 0.972 | 0.8242 |
| hsa-miR-938 | MI0005760 | UGCCCUUAAAGGUGAACCCAGU | 0.7841 | 0.8693 | 1.4743 | 2.0531 | 1.4114 | 1.6968 |
| hsa-miR-939 | MI0005761 | UGGGGAGCUGAGGCUCUGGGGGUG | 1.1978 | 1.2211 | 1.4721 | 0.6834 | 1.3956 | 0.9631 |
| hsa-miR-940 | MI0005762 | AAGGCAGGGCCCCCGCUCCCC | 0.9156 | 1.5687 | 1.5308 | 0.5473 | 1.5374 | 1.5653 |
| hsa-miR-941 | MI0005763 | CACCCGGCUGUGUGCACAUGUGC | 0.7369 | 1.4341 | 1.3691 | 0.6783 | -0.2144 | 0.5828 |
| hsa-miR-941 | MI0005764 | CACCCGGCUGUGUGCACAUGUGC | 0.9823 | 1.4542 | 1.4369 | 0.7038 | 1.2433 | 0.8168 |
| hsa-miR-941 | MI0005765 | CACCCGGCUGUGUGCACAUGUGC | 1.0094 | 1.5476 | 1.7156 | 0.7129 | 1.2753 | 0.94 |
| hsa-miR-941 | MI0005766 | CACCCGGCUGUGUGCACAUGUGC | 1.3246 | 1.5612 | 1.7834 | 0.7918 | 1.5491 | 2.1467 |
| hsa-miR-942 | MI0005767 | UCUUCUCUGUUUUGGCCAUGUG | 1.0997 | 1.7122 | 1.4012 | 0.8533 | 1.191 | 1.9589 |
| hsa-miR-943 | MI0005768 | CUGACUGUUGCCGUCCUCCAG | 1.0745 | 1.4384 | 1.307 | 1.0415 | 0.5618 | 1.177 |
| hsa-miR-944 | MI0005769 | AAAUUAUUGUACAUCGGAUGAG | 0.8207 | 1.6882 | 1.6385 | 0.378 | 1.9498 | 2.1106 |
| hsa-miR-95 | MI0000097 | UUCAACGGGUAUUUAUUGAGCA | 0.6252 | 1.1763 | 0.9322 | 0.9466 | 0.8772 | 1.1216 |
| hsa-miR-96 | MI0000098 | UUUGGCACUAGCACAUUUUUGCU | 0.9282 | 1.6958 | 1.2601 | 0.7194 | 0.5916 | 0.619 |
| hsa-miR-96* | MI0000098 | AAUCAUGUGCAGUGCCAAUAUG | 0.5488 | 1.0613 | 0.5764 | 0.8011 | 0.6662 | 0.5732 |
| hsa-miR-98 | MI0000100 | UGAGGUAGUAAGUUGUAUUGUU | 1.8941 | 0.8537 | 1.0929 | 1.5305 | 0.3764 | 0.9286 |
| hsa-miR-99a | MI0000101 | AACCCGUAGAUCCGAUCUUGUG | 1.2818 | 0.8317 | -0.8103 | 0.3848 | 0.0795 | 0.6082 |
| hsa-miR-99a* | MI0000101 | CAAGCUCGCUUCUAUGGGUCUG | 1.0766 | 1.1974 | 0.0431 | 0.2712 | 0.7924 | 0.6564 |
| hsa-miR-99b | MI0000746 | CACCCGUAGAACCGACCUUGCG | 0.3848 | 0.7181 | 0.6162 | 0.6934 | 1.2598 | 1.2854 |
| hsa-miR-99b* | MI0000746 | CAAGCUCGUGUCUGUGGGUCCG | 0.58 | 0.7349 | 0.7256 | 0.77 | 1.3354 | 0.598 |

| Supplementary Table S2. Normalized AR transcriptional activity, relative to control miRNA, as determined by PSE-PBN luciferase activity. Sample transfection efficiency normalized by Renilla luciferase. | | | |
| --- | --- | --- | --- |
|  |  |  |  |
|  |  |  |  |
| miRNA | miRBase | Sequence | Relative value |
| hsa-let-7a | MI0000061 | UGAGGUAGUAGGUUGUAUAGUU | 0.2685 |
| hsa-let-7a | MI0000062 | UGAGGUAGUAGGUUGUAUAGUU | 0.2702 |
| hsa-let-7a | MI0000060 | UGAGGUAGUAGGUUGUAUAGUU | 0.5146 |
| hsa-let-7a* | MI0000062 | CUAUACAAUCUACUGUCUUUC | 0.9731 |
| hsa-let-7a* | MI0000060 | CUAUACAAUCUACUGUCUUUC | 1.1648 |
| hsa-let-7b | MI0000063 | UGAGGUAGUAGGUUGUGUGGUU | 0.9531 |
| hsa-let-7b* | MI0000063 | CUAUACAACCUACUGCCUUCCC | 2.6219 |
| hsa-let-7c | MI0000064 | UGAGGUAGUAGGUUGUAUGGUU | 0.8136 |
| hsa-let-7c* | MI0000064 | UAGAGUUACACCCUGGGAGUUA | 0.5802 |
| hsa-let-7d | MI0000065 | AGAGGUAGUAGGUUGCAUAGUU | 0.7593 |
| hsa-let-7d* | MI0000065 | CUAUACGACCUGCUGCCUUUCU | 2.5455 |
| hsa-let-7e | MI0000066 | UGAGGUAGGAGGUUGUAUAGUU | 0.8917 |
| hsa-let-7e* | MI0000066 | CUAUACGGCCUCCUAGCUUUCC | 0.9428 |
| hsa-let-7f | MI0000068 | UGAGGUAGUAGAUUGUAUAGUU | 0.1226 |
| hsa-let-7f | MI0000067 | UGAGGUAGUAGAUUGUAUAGUU | 0.7241 |
| hsa-let-7f-1* | MI0000067 | CUAUACAAUCUAUUGCCUUCCC | 0.3192 |
| hsa-let-7f-2* | MI0000068 | CUAUACAGUCUACUGUCUUUCC | 0.8038 |
| hsa-let-7g | MI0000433 | UGAGGUAGUAGUUUGUACAGUU | 1.1658 |
| hsa-let-7g* | MI0000433 | CUGUACAGGCCACUGCCUUGC | 0.3249 |
| hsa-let-7i | MI0000434 | UGAGGUAGUAGUUUGUGCUGUU | 0.404 |
| hsa-let-7i* | MI0000434 | CUGCGCAAGCUACUGCCUUGCU | 0.3098 |
| hsa-miR-1 | MI0000437 | UGGAAUGUAAAGAAGUAUGUAU | 0.1766 |
| hsa-miR-1 | MI0000651 | UGGAAUGUAAAGAAGUAUGUAU | 0.2391 |
| hsa-miR-100 | MI0000102 | AACCCGUAGAUCCGAACUUGUG | 0.3875 |
| hsa-miR-100* | MI0000102 | CAAGCUUGUAUCUAUAGGUAUG | 1.6567 |
| hsa-miR-101 | MI0000739 | UACAGUACUGUGAUAACUGAA | 0.0619 |
| hsa-miR-101 | MI0000103 | UACAGUACUGUGAUAACUGAA | 0.3235 |
| hsa-miR-101* | MI0000103 | CAGUUAUCACAGUGCUGAUGCU | 1.9931 |
| hsa-miR-103 | MI0000108 | AGCAGCAUUGUACAGGGCUAUGA | 1.6335 |
| hsa-miR-103 | MI0000109 | AGCAGCAUUGUACAGGGCUAUGA | 1.8484 |
| hsa-miR-105 | MI0000111 | UCAAAUGCUCAGACUCCUGUGGU | 3.1243 |
| hsa-miR-105 | MI0000112 | UCAAAUGCUCAGACUCCUGUGGU | 4.1512 |
| hsa-miR-105* | MI0000111 | ACGGAUGUUUGAGCAUGUGCUA | 0.5831 |
| hsa-miR-105* | MI0000112 | ACGGAUGUUUGAGCAUGUGCUA | 0.9289 |
| hsa-miR-106a | MI0000113 | AAAAGUGCUUACAGUGCAGGUAG | 0.4214 |
| hsa-miR-106a* | MI0000113 | CUGCAAUGUAAGCACUUCUUAC | 0.4677 |
| hsa-miR-106b | MI0000734 | UAAAGUGCUGACAGUGCAGAU | 1.207 |
| hsa-miR-106b* | MI0000734 | CCGCACUGUGGGUACUUGCUGC | 1.5704 |
| hsa-miR-107 | MI0000114 | AGCAGCAUUGUACAGGGCUAUCA | 0.4191 |
| hsa-miR-10a | MI0000266 | UACCCUGUAGAUCCGAAUUUGUG | 0.4464 |
| hsa-miR-10a* | MI0000266 | CAAAUUCGUAUCUAGGGGAAUA | 1.4999 |
| hsa-miR-10b | MI0000267 | UACCCUGUAGAACCGAAUUUGUG | 1.0713 |
| hsa-miR-10b* | MI0000267 | ACAGAUUCGAUUCUAGGGGAAU | 1.083 |
| hsa-miR-122* | MI0000442 | AACGCCAUUAUCACACUAAAUA | 0.1777 |
| hsa-miR-1224-3p | MI0003764 | CCCCACCUCCUCUCUCCUCAG | 1.1267 |
| hsa-miR-1224-5p | MI0003764 | GUGAGGACUCGGGAGGUGG | 0.8077 |
| hsa-miR-1225-3p | MI0006311 | UGAGCCCCUGUGCCGCCCCCAG | 1.1758 |
| hsa-miR-1225-5p | MI0006311 | GUGGGUACGGCCCAGUGGGGGG | 1.4804 |
| hsa-miR-1226 | MI0006313 | UCACCAGCCCUGUGUUCCCUAG | 0.6429 |
| hsa-miR-1226* | MI0006313 | GUGAGGGCAUGCAGGCCUGGAUGGGG | 0.658 |
| hsa-miR-1227 | MI0006316 | CGUGCCACCCUUUUCCCCAG | 0.3853 |
| hsa-miR-1228 | MI0006318 | UCACACCUGCCUCGCCCCCC | 0.897 |
| hsa-miR-1228* | MI0006318 | GUGGGCGGGGGCAGGUGUGUG | 0.5335 |
| hsa-miR-1229 | MI0006319 | CUCUCACCACUGCCCUCCCACAG | 0.3808 |
| hsa-miR-122a | MI0000442 | UGGAGUGUGACAAUGGUGUUUG | 0.1003 |
| hsa-miR-1231 | MI0006321 | GUGUCUGGGCGGACAGCUGC | 0.5525 |
| hsa-miR-1233 | MI0006323 | UGAGCCCUGUCCUCCCGCAG | 1.0076 |
| hsa-miR-1234 | MI0006324 | UCGGCCUGACCACCCACCCCAC | 0.8953 |
| hsa-miR-1236 | MI0006326 | CCUCUUCCCCUUGUCUCUCCAG | 2.3422 |
| hsa-miR-1237 | MI0006327 | UCCUUCUGCUCCGUCCCCCAG | 1.1091 |
| hsa-miR-1238 | MI0006328 | CUUCCUCGUCUGUCUGCCCC | 1.7443 |
| hsa-miR-124* | MI0000443 | CGUGUUCACAGCGGACCUUGAU | 0.4295 |
| hsa-miR-124* | MI0000445 | CGUGUUCACAGCGGACCUUGAU | 0.6828 |
| hsa-miR-124* | MI0000444 | CGUGUUCACAGCGGACCUUGAU | 1.4496 |
| hsa-miR-124a | MI0000443 | UAAGGCACGCGGUGAAUGCC | 0.6113 |
| hsa-miR-124a | MI0000444 | UAAGGCACGCGGUGAAUGCC | 0.806 |
| hsa-miR-124a | MI0000445 | UAAGGCACGCGGUGAAUGCC | 1.1946 |
| hsa-miR-125a | MI0000469 | UCCCUGAGACCCUUUAACCUGUGA | 0.9553 |
| hsa-miR-125a-3p | MI0000469 | ACAGGUGAGGUUCUUGGGAGCC | 1.4527 |
| hsa-miR-125b | MI0000446 | UCCCUGAGACCCUAACUUGUGA | 0.878 |
| hsa-miR-125b | MI0000470 | UCCCUGAGACCCUAACUUGUGA | 0.8955 |
| hsa-miR-125b-1* | MI0000446 | ACGGGUUAGGCUCUUGGGAGCU | 0.4493 |
| hsa-miR-125b-2* | MI0000470 | UCACAAGUCAGGCUCUUGGGAC | 1.3745 |
| hsa-miR-126 | MI0000471 | UCGUACCGUGAGUAAUAAUGCG | 0.6729 |
| hsa-miR-126* | MI0000471 | CAUUAUUACUUUUGGUACGCG | 0.1964 |
| hsa-miR-127 | MI0000472 | UCGGAUCCGUCUGAGCUUGGCU | 0.3613 |
| hsa-miR-127-5p | MI0000472 | CUGAAGCUCAGAGGGCUCUGAU | 0.8976 |
| hsa-miR-128 | MI0000727 | UCACAGUGAACCGGUCUCUUU | 1.5991 |
| hsa-miR-128a | MI0000447 | UCACAGUGAACCGGUCUCUUU | 1.2812 |
| hsa-miR-129 | MI0000473 | CUUUUUGCGGUCUGGGCUUGC | 0.4688 |
| hsa-miR-129 | MI0000252 | CUUUUUGCGGUCUGGGCUUGC | 1.0393 |
| hsa-miR-129* | MI0000252 | AAGCCCUUACCCCAAAAAGUAU | 0.9351 |
| hsa-miR-129-3p | MI0000473 | AAGCCCUUACCCCAAAAAGCAU | 0.2851 |
| hsa-miR-130a | MI0000448 | CAGUGCAAUGUUAAAAGGGCAU | 0.4103 |
| hsa-miR-130a* | MI0000448 | UUCACAUUGUGCUACUGUCUGC | 0.5787 |
| hsa-miR-130b | MI0000748 | CAGUGCAAUGAUGAAAGGGCAU | 0.5201 |
| hsa-miR-130b* | MI0000748 | ACUCUUUCCCUGUUGCACUAC | 0.7167 |
| hsa-miR-132 | MI0000449 | UAACAGUCUACAGCCAUGGUCG | 1.3496 |
| hsa-miR-132* | MI0000449 | ACCGUGGCUUUCGAUUGUUACU | 0.3744 |
| hsa-miR-133a | MI0000450 | UUUGGUCCCCUUCAACCAGCUG | 1.0229 |
| hsa-miR-133a | MI0000451 | UUUGGUCCCCUUCAACCAGCUG | 1.2429 |
| hsa-miR-133b | MI0000822 | UUUGGUCCCCUUCAACCAGCUA | 1.3265 |
| hsa-miR-134 | MI0000474 | UGUGACUGGUUGACCAGAGGGG | 0.8183 |
| hsa-miR-135a | MI0000452 | UAUGGCUUUUUAUUCCUAUGUGA | 0.2241 |
| hsa-miR-135a | MI0000453 | UAUGGCUUUUUAUUCCUAUGUGA | 0.2554 |
| hsa-miR-135a* | MI0000452 | UAUAGGGAUUGGAGCCGUGGCG | 0.8304 |
| hsa-miR-135b | MI0000810 | UAUGGCUUUUCAUUCCUAUGUGA | 0.7443 |
| hsa-miR-135b* | MI0000810 | AUGUAGGGCUAAAAGCCAUGGG | 0.5285 |
| hsa-miR-136 | MI0000475 | ACUCCAUUUGUUUUGAUGAUGGA | 1.5803 |
| hsa-miR-136* | MI0000475 | CAUCAUCGUCUCAAAUGAGUCU | 3.6905 |
| hsa-miR-137 | MI0000454 | UUAUUGCUUAAGAAUACGCGUAG | 1.3629 |
| hsa-miR-138 | MI0000455 | AGCUGGUGUUGUGAAUCAGGCCG | 0.2062 |
| hsa-miR-138 | MI0000476 | AGCUGGUGUUGUGAAUCAGGCCG | 0.489 |
| hsa-miR-138-1* | MI0000476 | GCUACUUCACAACACCAGGGCC | 1.5916 |
| hsa-miR-138-2* | MI0000455 | GCUAUUUCACGACACCAGGGUU | 0.9446 |
| hsa-miR-139 | MI0000261 | UCUACAGUGCACGUGUCUCCAG | 0.7215 |
| hsa-miR-139-3p | MI0000261 | GGAGACGCGGCCCUGUUGGAGU | 1.929 |
| hsa-miR-140 | MI0000456 | CAGUGGUUUUACCCUAUGGUAG | 1.5963 |
| hsa-miR-140-3p | MI0000456 | UACCACAGGGUAGAACCACGG | 0.304 |
| hsa-miR-141 | MI0000457 | UAACACUGUCUGGUAAAGAUGG | 0.2889 |
| hsa-miR-141* | MI0000457 | CAUCUUCCAGUACAGUGUUGGA | 2.9506 |
| hsa-miR-142-3p | MI0000458 | UGUAGUGUUUCCUACUUUAUGGA | 0.3309 |
| hsa-miR-142-5p | MI0000458 | CAUAAAGUAGAAAGCACUACU | 0.5831 |
| hsa-miR-143 | MI0000459 | UGAGAUGAAGCACUGUAGCUC | 0.1365 |
| hsa-miR-143* | MI0000459 | GGUGCAGUGCUGCAUCUCUGGU | 0.8432 |
| hsa-miR-144 | MI0000460 | UACAGUAUAGAUGAUGUACU | 0.3368 |
| hsa-miR-144* | MI0000460 | GGAUAUCAUCAUAUACUGUAAG | 0.3206 |
| hsa-miR-145 | MI0000461 | GUCCAGUUUUCCCAGGAAUCCCU | 0.312 |
| hsa-miR-145* | MI0000461 | GGAUUCCUGGAAAUACUGUUCU | 0.9477 |
| hsa-miR-146a | MI0000477 | UGAGAACUGAAUUCCAUGGGUU | 0.3001 |
| hsa-miR-146a* | MI0000477 | CCUCUGAAAUUCAGUUCUUCAG | 1.4263 |
| hsa-miR-146b | MI0003129 | UGAGAACUGAAUUCCAUAGGCU | 0.2711 |
| hsa-miR-146b-3p | MI0003129 | UGCCCUGUGGACUCAGUUCUGG | 0.5268 |
| hsa-miR-147 | MI0000262 | GUGUGUGGAAAUGCUUCUGC | 0.0857 |
| hsa-miR-147b | MI0005544 | GUGUGCGGAAAUGCUUCUGCUA | 0.251 |
| hsa-miR-148a | MI0000253 | UCAGUGCACUACAGAACUUUGU | 1.1723 |
| hsa-miR-148a* | MI0000253 | AAAGUUCUGAGACACUCCGACU | 2.6453 |
| hsa-miR-148b | MI0000811 | UCAGUGCAUCACAGAACUUUGU | 0.549 |
| hsa-miR-148b* | MI0000811 | AAGUUCUGUUAUACACUCAGGC | 3.5768 |
| hsa-miR-149 | MI0000478 | UCUGGCUCCGUGUCUUCACUCCC | 1.0017 |
| hsa-miR-149* | MI0000478 | AGGGAGGGACGGGGGCUGUGC | 0.1701 |
| hsa-miR-150 | MI0000479 | UCUCCCAACCCUUGUACCAGUG | 0.6679 |
| hsa-miR-150* | MI0000479 | CUGGUACAGGCCUGGGGGACAG | 0.9116 |
| hsa-miR-151 | MI0000809 | CUAGACUGAAGCUCCUUGAGG | 0.1455 |
| hsa-miR-151-5p | MI0000809 | UCGAGGAGCUCACAGUCUAGU | 0.8719 |
| hsa-miR-152 | MI0000462 | UCAGUGCAUGACAGAACUUGG | 0.3627 |
| hsa-miR-153 | MI0000463 | UUGCAUAGUCACAAAAGUGAUC | 1.0688 |
| hsa-miR-153 | MI0000464 | UUGCAUAGUCACAAAAGUGAUC | 2.4259 |
| hsa-miR-154 | MI0000480 | UAGGUUAUCCGUGUUGCCUUCG | 0.7185 |
| hsa-miR-154* | MI0000480 | AAUCAUACACGGUUGACCUAUU | 1.1337 |
| hsa-miR-155 | MI0000681 | UUAAUGCUAAUCGUGAUAGGGGU | 0.3958 |
| hsa-miR-155* | MI0000681 | CUCCUACAUAUUAGCAUUAACA | 1.1835 |
| hsa-miR-15a | MI0000069 | UAGCAGCACAUAAUGGUUUGUG | 0.5517 |
| hsa-miR-15a* | MI0000069 | CAGGCCAUAUUGUGCUGCCUCA | 0.7991 |
| hsa-miR-15b | MI0000438 | UAGCAGCACAUCAUGGUUUACA | 0.2791 |
| hsa-miR-15b* | MI0000438 | CGAAUCAUUAUUUGCUGCUCUA | 3.1261 |
| hsa-miR-16 | MI0000070 | UAGCAGCACGUAAAUAUUGGCG | 0.2404 |
| hsa-miR-16 | MI0000115 | UAGCAGCACGUAAAUAUUGGCG | 0.6306 |
| hsa-miR-16-1* | MI0000070 | CCAGUAUUAACUGUGCUGCUGA | 5.0261 |
| hsa-miR-16-2* | MI0000115 | CCAAUAUUACUGUGCUGCUUUA | 1.2959 |
| hsa-miR-17-3p | MI0000071 | ACUGCAGUGAAGGCACUUGUAG | 1.2685 |
| hsa-miR-17-5p | MI0000071 | CAAAGUGCUUACAGUGCAGGUAG | 0.5777 |
| hsa-miR-181a | MI0000269 | AACAUUCAACGCUGUCGGUGAGU | 0.4611 |
| hsa-miR-181a | MI0000289 | AACAUUCAACGCUGUCGGUGAGU | 3.1915 |
| hsa-miR-181a-2* | MI0000269 | ACCACUGACCGUUGACUGUACC | 0.8272 |
| hsa-miR-181b | MI0000270 | AACAUUCAUUGCUGUCGGUGGGU | 2.3323 |
| hsa-miR-181b | MI0000683 | AACAUUCAUUGCUGUCGGUGGGU | 2.9342 |
| hsa-miR-181c | MI0000271 | AACAUUCAACCUGUCGGUGAGU | 0.8695 |
| hsa-miR-181c* | MI0000271 | AACCAUCGACCGUUGAGUGGAC | 0.4901 |
| hsa-miR-181d | MI0003139 | AACAUUCAUUGUUGUCGGUGGGU | 1.5892 |
| hsa-miR-182 | MI0000272 | UUUGGCAAUGGUAGAACUCACACU | 3.1969 |
| hsa-miR-182* | MI0000272 | UGGUUCUAGACUUGCCAACUA | 1.0621 |
| hsa-miR-183 | MI0000273 | UAUGGCACUGGUAGAAUUCACU | 1.3893 |
| hsa-miR-183* | MI0000273 | GUGAAUUACCGAAGGGCCAUAA | 1.1327 |
| hsa-miR-184 | MI0000481 | UGGACGGAGAACUGAUAAGGGU | 0.2515 |
| hsa-miR-185 | MI0000482 | UGGAGAGAAAGGCAGUUCCUGA | 0.2711 |
| hsa-miR-185* | MI0000482 | AGGGGCUGGCUUUCCUCUGGUC | 0.29 |
| hsa-miR-186 | MI0000483 | CAAAGAAUUCUCCUUUUGGGCU | 1.137 |
| hsa-miR-186* | MI0000483 | GCCCAAAGGUGAAUUUUUUGGG | 1.223 |
| hsa-miR-187 | MI0000274 | UCGUGUCUUGUGUUGCAGCCGG | 0.5475 |
| hsa-miR-187* | MI0000274 | GGCUACAACACAGGACCCGGGC | 0.6398 |
| hsa-miR-188 | MI0000484 | CAUCCCUUGCAUGGUGGAGGG | 1.0348 |
| hsa-miR-188-3p | MI0000484 | CUCCCACAUGCAGGGUUUGCA | 0.5149 |
| hsa-miR-189 | MI0000080 | UGCCUACUGAGCUGAUAUCAGU | 0.8458 |
| hsa-miR-18a | MI0000072 | UAAGGUGCAUCUAGUGCAGAUAG | 1.6062 |
| hsa-miR-18a* | MI0000072 | ACUGCCCUAAGUGCUCCUUCUGG | 1.7208 |
| hsa-miR-18b | MI0001518 | UAAGGUGCAUCUAGUGCAGUUAG | 1.6298 |
| hsa-miR-18b* | MI0001518 | UGCCCUAAAUGCCCCUUCUGGC | 1.6782 |
| hsa-miR-190 | MI0000486 | UGAUAUGUUUGAUAUAUUAGGU | 0.2043 |
| hsa-miR-190b | MI0005545 | UGAUAUGUUUGAUAUUGGGUU | 0.918 |
| hsa-miR-191 | MI0000465 | CAACGGAAUCCCAAAAGCAGCUG | 0.3045 |
| hsa-miR-191* | MI0000465 | GCUGCGCUUGGAUUUCGUCCCC | 0.1535 |
| hsa-miR-192 | MI0000234 | CUGACCUAUGAAUUGACAGCC | 0.2842 |
| hsa-miR-192* | MI0000234 | CUGCCAAUUCCAUAGGUCACAG | 3.0648 |
| hsa-miR-193a | MI0000487 | AACUGGCCUACAAAGUCCCAGU | 0.276 |
| hsa-miR-193a-5p | MI0000487 | UGGGUCUUUGCGGGCGAGAUGA | 0.5447 |
| hsa-miR-193b | MI0003137 | AACUGGCCCUCAAAGUCCCGCU | 0.4416 |
| hsa-miR-193b* | MI0003137 | CGGGGUUUUGAGGGCGAGAUGA | 0.6161 |
| hsa-miR-194 | MI0000732 | UGUAACAGCAACUCCAUGUGGA | 0.253 |
| hsa-miR-194 | MI0000488 | UGUAACAGCAACUCCAUGUGGA | 0.4279 |
| hsa-miR-194* | MI0000732 | CCAGUGGGGCUGCUGUUAUCUG | 2.3634 |
| hsa-miR-195 | MI0000489 | UAGCAGCACAGAAAUAUUGGC | 2.9945 |
| hsa-miR-195* | MI0000489 | CCAAUAUUGGCUGUGCUGCUCC | 1.2911 |
| hsa-miR-196a | MI0000238 | UAGGUAGUUUCAUGUUGUUGGG | 0.7918 |
| hsa-miR-196a | MI0000279 | UAGGUAGUUUCAUGUUGUUGGG | 1.2782 |
| hsa-miR-196a* | MI0000279 | CGGCAACAAGAAACUGCCUGAG | 0.1545 |
| hsa-miR-196b | MI0001150 | UAGGUAGUUUCCUGUUGUUGGG | 1.3925 |
| hsa-miR-197 | MI0000239 | UUCACCACCUUCUCCACCCAGC | 0.3877 |
| hsa-miR-198 | MI0000240 | GGUCCAGAGGGGAGAUAGGUUC | 0.6258 |
| hsa-miR-199a | MI0000242 | CCCAGUGUUCAGACUACCUGUUC | 0.4666 |
| hsa-miR-199a | MI0000281 | CCCAGUGUUCAGACUACCUGUUC | 0.5895 |
| hsa-miR-199a* | MI0000242 | ACAGUAGUCUGCACAUUGGUUA | 0.1293 |
| hsa-miR-199a* | MI0000281 | ACAGUAGUCUGCACAUUGGUUA | 0.4623 |
| hsa-miR-199b | MI0000282 | CCCAGUGUUUAGACUAUCUGUUC | 0.7212 |
| hsa-miR-199b-3p | MI0000282 | ACAGUAGUCUGCACAUUGGUUA | 0.3208 |
| hsa-miR-19a | MI0000073 | UGUGCAAAUCUAUGCAAAACUGA | 0.9704 |
| hsa-miR-19a* | MI0000073 | AGUUUUGCAUAGUUGCACUACA | 0.7182 |
| hsa-miR-19b | MI0000074 | UGUGCAAAUCCAUGCAAAACUGA | 0.274 |
| hsa-miR-19b | MI0000075 | UGUGCAAAUCCAUGCAAAACUGA | 0.5181 |
| hsa-miR-19b-1* | MI0000074 | AGUUUUGCAGGUUUGCAUCCAGC | 1.2192 |
| hsa-miR-19b-2* | MI0000075 | AGUUUUGCAGGUUUGCAUUUCA | 0.5199 |
| hsa-miR-200a | MI0000737 | UAACACUGUCUGGUAACGAUGU | 0.141 |
| hsa-miR-200a* | MI0000737 | CAUCUUACCGGACAGUGCUGGA | 0.4693 |
| hsa-miR-200b | MI0000342 | UAAUACUGCCUGGUAAUGAUGA | 2.2445 |
| hsa-miR-200b* | MI0000342 | CAUCUUACUGGGCAGCAUUGGA | 1.2628 |
| hsa-miR-200c | MI0000650 | UAAUACUGCCGGGUAAUGAUGGA | 0.3473 |
| hsa-miR-200c* | MI0000650 | CGUCUUACCCAGCAGUGUUUGG | 0.3909 |
| hsa-miR-202 | MI0003130 | AGAGGUAUAGGGCAUGGGAA | 0.5815 |
| hsa-miR-202* | MI0003130 | UUCCUAUGCAUAUACUUCUUUG | 0.3983 |
| hsa-miR-203 | MI0000283 | GUGAAAUGUUUAGGACCACUAG | 0.539 |
| hsa-miR-204 | MI0000284 | UUCCCUUUGUCAUCCUAUGCCU | 0.1199 |
| hsa-miR-205 | MI0000285 | UCCUUCAUUCCACCGGAGUCUG | 0.1727 |
| hsa-miR-206 | MI0000490 | UGGAAUGUAAGGAAGUGUGUGG | 0.2655 |
| hsa-miR-208 | MI0000251 | AUAAGACGAGCAAAAAGCUUGU | 0.6652 |
| hsa-miR-208b | MI0005570 | AUAAGACGAACAAAAGGUUUGU | 1.1728 |
| hsa-miR-20a | MI0000076 | UAAAGUGCUUAUAGUGCAGGUAG | 0.2592 |
| hsa-miR-20a* | MI0000076 | ACUGCAUUAUGAGCACUUAAAG | 0.8867 |
| hsa-miR-20b | MI0001519 | CAAAGUGCUCAUAGUGCAGGUAG | 0.3002 |
| hsa-miR-20b* | MI0001519 | ACUGUAGUAUGGGCACUUCCAG | 0.5507 |
| hsa-miR-21 | MI0000077 | UAGCUUAUCAGACUGAUGUUGA | 0.6311 |
| hsa-miR-21* | MI0000077 | CAACACCAGUCGAUGGGCUGU | 1.7428 |
| hsa-miR-210 | MI0000286 | CUGUGCGUGUGACAGCGGCUGA | 0.4394 |
| hsa-miR-211 | MI0000287 | UUCCCUUUGUCAUCCUUCGCCU | 0.6936 |
| hsa-miR-212 | MI0000288 | UAACAGUCUCCAGUCACGGCC | 0.8193 |
| hsa-miR-213 | MI0000289 | ACCAUCGACCGUUGAUUGUACC | 0.7784 |
| hsa-miR-214 | MI0000290 | ACAGCAGGCACAGACAGGCAGU | 0.7388 |
| hsa-miR-214* | MI0000290 | UGCCUGUCUACACUUGCUGUGC | 0.5388 |
| hsa-miR-215 | MI0000291 | AUGACCUAUGAAUUGACAGAC | 0.178 |
| hsa-miR-216 | MI0000292 | UAAUCUCAGCUGGCAACUGUGA | 1.3764 |
| hsa-miR-216b | MI0005569 | AAAUCUCUGCAGGCAAAUGUGA | 0.8591 |
| hsa-miR-217 | MI0000293 | UACUGCAUCAGGAACUGAUUGGA | 1.5674 |
| hsa-miR-218 | MI0000295 | UUGUGCUUGAUCUAACCAUGU | 0.1266 |
| hsa-miR-218 | MI0000294 | UUGUGCUUGAUCUAACCAUGU | 1.0069 |
| hsa-miR-218-1* | MI0000294 | AUGGUUCCGUCAAGCACCAUGG | 0.8199 |
| hsa-miR-218-2* | MI0000295 | CAUGGUUCUGUCAAGCACCGCG | 0.6699 |
| hsa-miR-219 | MI0000740 | UGAUUGUCCAAACGCAAUUCU | 0.2152 |
| hsa-miR-219 | MI0000296 | UGAUUGUCCAAACGCAAUUCU | 0.9417 |
| hsa-miR-219-1-3p | MI0000296 | AGAGUUGAGUCUGGACGUCCCG | 0.4758 |
| hsa-miR-219-2-3p | MI0000740 | AGAAUUGUGGCUGGACAUCUGU | 1.0618 |
| hsa-miR-22 | MI0000078 | AAGCUGCCAGUUGAAGAACUGU | 0.715 |
| hsa-miR-22* | MI0000078 | AGUUCUUCAGUGGCAAGCUUUA | 1.0852 |
| hsa-miR-220 | MI0000297 | CCACACCGUAUCUGACACUUU | 0.7196 |
| hsa-miR-220b | MI0005529 | CCACCACCGUGUCUGACACUU | 5.1475 |
| hsa-miR-220c | MI0005536 | ACACAGGGCUGUUGUGAAGACU | 0.5059 |
| hsa-miR-221 | MI0000298 | AGCUACAUUGUCUGCUGGGUUUC | 0.1732 |
| hsa-miR-221* | MI0000298 | ACCUGGCAUACAAUGUAGAUUU | 0.3182 |
| hsa-miR-222 | MI0000299 | AGCUACAUCUGGCUACUGGGU | 1.4747 |
| hsa-miR-222* | MI0000299 | CUCAGUAGCCAGUGUAGAUCCU | 2.0043 |
| hsa-miR-223 | MI0000300 | UGUCAGUUUGUCAAAUACCCCA | 0.7392 |
| hsa-miR-223* | MI0000300 | CGUGUAUUUGACAAGCUGAGUU | 0.3457 |
| hsa-miR-224 | MI0000301 | CAAGUCACUAGUGGUUCCGUU | 3.3945 |
| hsa-miR-23a | MI0000079 | AUCACAUUGCCAGGGAUUUCC | 0.4001 |
| hsa-miR-23a* | MI0000079 | GGGGUUCCUGGGGAUGGGAUUU | 0.7267 |
| hsa-miR-23b | MI0000439 | AUCACAUUGCCAGGGAUUACC | 0.2383 |
| hsa-miR-23b* | MI0000439 | UGGGUUCCUGGCAUGCUGAUUU | 0.7913 |
| hsa-miR-24 | MI0000081 | UGGCUCAGUUCAGCAGGAACAG | 0.1121 |
| hsa-miR-24 | MI0000080 | UGGCUCAGUUCAGCAGGAACAG | 0.1438 |
| hsa-miR-24-2* | MI0000081 | UGCCUACUGAGCUGAAACACAG | 1.0825 |
| hsa-miR-25 | MI0000082 | CAUUGCACUUGUCUCGGUCUGA | 0.8404 |
| hsa-miR-25* | MI0000082 | AGGCGGAGACUUGGGCAAUUG | 0.2084 |
| hsa-miR-26a | MI0000083 | UUCAAGUAAUCCAGGAUAGGCU | 0.5584 |
| hsa-miR-26a | MI0000750 | UUCAAGUAAUCCAGGAUAGGCU | 0.8945 |
| hsa-miR-26a-1* | MI0000083 | CCUAUUCUUGGUUACUUGCACG | 2.454 |
| hsa-miR-26a-2* | MI0000750 | CCUAUUCUUGAUUACUUGUUUC | 1.1862 |
| hsa-miR-26b | MI0000084 | UUCAAGUAAUUCAGGAUAGGU | 1.3548 |
| hsa-miR-26b* | MI0000084 | CCUGUUCUCCAUUACUUGGCUC | 2.0577 |
| hsa-miR-27a | MI0000085 | UUCACAGUGGCUAAGUUCCGC | 1.4361 |
| hsa-miR-27a* | MI0000085 | AGGGCUUAGCUGCUUGUGAGCA | 1.0885 |
| hsa-miR-27b | MI0000440 | UUCACAGUGGCUAAGUUCUGC | 0.2209 |
| hsa-miR-27b* | MI0000440 | AGAGCUUAGCUGAUUGGUGAAC | 1.2718 |
| hsa-miR-28 | MI0000086 | AAGGAGCUCACAGUCUAUUGAG | 0.2962 |
| hsa-miR-28-3p | MI0000086 | CACUAGAUUGUGAGCUCCUGGA | 1.3575 |
| hsa-miR-296 | MI0000747 | AGGGCCCCCCCUCAAUCCUGU | 0.468 |
| hsa-miR-296-3p | MI0000747 | GAGGGUUGGGUGGAGGCUCUCC | 0.4003 |
| hsa-miR-297 | MI0005775 | AUGUAUGUGUGCAUGUGCAUG | 0.3602 |
| hsa-miR-298 | MI0005523 | AGCAGAAGCAGGGAGGUUCUCCCA | 2.2498 |
| hsa-miR-299-3p | MI0000744 | UAUGUGGGAUGGUAAACCGCUU | 0.1129 |
| hsa-miR-299-5p | MI0000744 | UGGUUUACCGUCCCACAUACAU | 0.0375 |
| hsa-miR-29a | MI0000087 | UAGCACCAUCUGAAAUCGGUUA | 0.6955 |
| hsa-miR-29a* | MI0000087 | ACUGAUUUCUUUUGGUGUUCAG | 1.5688 |
| hsa-miR-29b | MI0000107 | UAGCACCAUUUGAAAUCAGUGUU | 0.1514 |
| hsa-miR-29b | MI0000105 | UAGCACCAUUUGAAAUCAGUGUU | 0.189 |
| hsa-miR-29b-1* | MI0000105 | GCUGGUUUCAUAUGGUGGUUUAGA | 0.9643 |
| hsa-miR-29b-2* | MI0000107 | CUGGUUUCACAUGGUGGCUUAG | 0.5133 |
| hsa-miR-29c | MI0000735 | UAGCACCAUUUGAAAUCGGUUA | 0.5924 |
| hsa-miR-29c* | MI0000735 | UGACCGAUUUCUCCUGGUGUUC | 1.3899 |
| hsa-miR-300 | MI0005525 | UAUACAAGGGCAGACUCUCUCU | 1.4341 |
| hsa-miR-301 | MI0000745 | CAGUGCAAUAGUAUUGUCAAAGC | 0.5244 |
| hsa-miR-301b | MI0005568 | CAGUGCAAUGAUAUUGUCAAAGC | 0.4615 |
| hsa-miR-302a | MI0000738 | UAAGUGCUUCCAUGUUUUGGUGA | 0.2926 |
| hsa-miR-302a* | MI0000738 | ACUUAAACGUGGAUGUACUUGCU | 0.8957 |
| hsa-miR-302b | MI0000772 | UAAGUGCUUCCAUGUUUUAGUAG | 0.2462 |
| hsa-miR-302b* | MI0000772 | ACUUUAACAUGGAAGUGCUUUC | 0.9072 |
| hsa-miR-302c | MI0000773 | UAAGUGCUUCCAUGUUUCAGUGG | 0.1121 |
| hsa-miR-302c* | MI0000773 | UUUAACAUGGGGGUACCUGCUG | 0.3659 |
| hsa-miR-302d | MI0000774 | UAAGUGCUUCCAUGUUUGAGUGU | 0.2156 |
| hsa-miR-302d* | MI0000774 | ACUUUAACAUGGAGGCACUUGC | 1.1706 |
| hsa-miR-30a-3p | MI0000088 | CUUUCAGUCGGAUGUUUGCAGC | 0.3783 |
| hsa-miR-30a-5p | MI0000088 | UGUAAACAUCCUCGACUGGAAG | 0.2376 |
| hsa-miR-30b | MI0000441 | UGUAAACAUCCUACACUCAGCU | 0.3016 |
| hsa-miR-30b* | MI0000441 | CUGGGAGGUGGAUGUUUACUUC | 0.1449 |
| hsa-miR-30c | MI0000254 | UGUAAACAUCCUACACUCUCAGC | 0.1329 |
| hsa-miR-30c | MI0000736 | UGUAAACAUCCUACACUCUCAGC | 1.9346 |
| hsa-miR-30c-1* | MI0000736 | CUGGGAGAGGGUUGUUUACUCC | 1.4644 |
| hsa-miR-30c-2* | MI0000254 | CUGGGAGAAGGCUGUUUACUCU | 0.6497 |
| hsa-miR-30d | MI0000255 | UGUAAACAUCCCCGACUGGAAG | 0.2421 |
| hsa-miR-30d* | MI0000255 | CUUUCAGUCAGAUGUUUGCUGC | 0.6382 |
| hsa-miR-30e-3p | MI0000749 | CUUUCAGUCGGAUGUUUACAGC | 0.145 |
| hsa-miR-30e-5p | MI0000749 | UGUAAACAUCCUUGACUGGAAG | 0.8789 |
| hsa-miR-31 | MI0000089 | AGGCAAGAUGCUGGCAUAGCU | 1.1235 |
| hsa-miR-31* | MI0000089 | UGCUAUGCCAACAUAUUGCCAU | 0.9146 |
| hsa-miR-32 | MI0000090 | UAUUGCACAUUACUAAGUUGCA | 1.0091 |
| hsa-miR-32* | MI0000090 | CAAUUUAGUGUGUGUGAUAUUU | 0.7352 |
| hsa-miR-320 | MI0000542 | AAAAGCUGGGUUGAGAGGGCGA | 0.4731 |
| hsa-miR-323 | MI0000807 | CACAUUACACGGUCGACCUCU | 0.1985 |
| hsa-miR-323-5p | MI0000807 | AGGUGGUCCGUGGCGCGUUCGC | 0.9099 |
| hsa-miR-324-3p | MI0000813 | ACUGCCCCAGGUGCUGCUGG | 0.2373 |
| hsa-miR-324-5p | MI0000813 | CGCAUCCCCUAGGGCAUUGGUGU | 1.7439 |
| hsa-miR-325 | MI0000824 | CCUAGUAGGUGUCCAGUAAGUGU | 0.5482 |
| hsa-miR-326 | MI0000808 | CCUCUGGGCCCUUCCUCCAG | 0.1666 |
| hsa-miR-328 | MI0000804 | CUGGCCCUCUCUGCCCUUCCGU | 0.6937 |
| hsa-miR-329 | MI0001725 | AACACACCUGGUUAACCUCUUU | 0.1952 |
| hsa-miR-329 | MI0001726 | AACACACCUGGUUAACCUCUUU | 0.9158 |
| hsa-miR-33 | MI0000091 | GUGCAUUGUAGUUGCAUUGCA | 1.295 |
| hsa-miR-330 | MI0000803 | GCAAAGCACACGGCCUGCAGAGA | 0.7747 |
| hsa-miR-330-5p | MI0000803 | UCUCUGGGCCUGUGUCUUAGGC | 0.3439 |
| hsa-miR-331 | MI0000812 | GCCCCUGGGCCUAUCCUAGAA | 0.1529 |
| hsa-miR-331-5p | MI0000812 | CUAGGUAUGGUCCCAGGGAUCC | 1.5186 |
| hsa-miR-335 | MI0000816 | UCAAGAGCAAUAACGAAAAAUGU | 0.0561 |
| hsa-miR-335* | MI0000816 | UUUUUCAUUAUUGCUCCUGACC | 1.2045 |
| hsa-miR-337 | MI0000806 | CUCCUAUAUGAUGCCUUUCUUC | 0.3153 |
| hsa-miR-337-5p | MI0000806 | GAACGGCUUCAUACAGGAGUU | 0.203 |
| hsa-miR-338 | MI0000814 | UCCAGCAUCAGUGAUUUUGUUG | 0.4719 |
| hsa-miR-338-5p | MI0000814 | AACAAUAUCCUGGUGCUGAGUG | 2.8275 |
| hsa-miR-339 | MI0000815 | UCCCUGUCCUCCAGGAGCUCACG | 1.3757 |
| hsa-miR-339-3p | MI0000815 | UGAGCGCCUCGACGACAGAGCCG | 0.6825 |
| hsa-miR-33a* | MI0000091 | CAAUGUUUCCACAGUGCAUCAC | 1.2823 |
| hsa-miR-33b | MI0003646 | GUGCAUUGCUGUUGCAUUGC | 1.1985 |
| hsa-miR-33b* | MI0003646 | CAGUGCCUCGGCAGUGCAGCCC | 1.2118 |
| hsa-miR-340 | MI0000802 | UUAUAAAGCAAUGAGACUGAUU | 0.551 |
| hsa-miR-340* | MI0000802 | UCCGUCUCAGUUACUUUAUAGC | 0.2584 |
| hsa-miR-342 | MI0000805 | UCUCACACAGAAAUCGCACCCGU | 0.4397 |
| hsa-miR-342-5p | MI0000805 | AGGGGUGCUAUCUGUGAUUGA | 0.4801 |
| hsa-miR-345 | MI0000825 | GCUGACUCCUAGUCCAGGGCUC | 3.3676 |
| hsa-miR-346 | MI0000826 | UGUCUGCCCGCAUGCCUGCCUCU | 1.5423 |
| hsa-miR-34a | MI0000268 | UGGCAGUGUCUUAGCUGGUUGU | 1.7215 |
| hsa-miR-34a* | MI0000268 | CAAUCAGCAAGUAUACUGCCCU | 2.9785 |
| hsa-miR-34b | MI0000742 | CAAUCACUAACUCCACUGCCAU | 0.8549 |
| hsa-miR-34b* | MI0000742 | UAGGCAGUGUCAUUAGCUGAUUG | 0.5944 |
| hsa-miR-34c | MI0000743 | AGGCAGUGUAGUUAGCUGAUUGC | 0.1471 |
| hsa-miR-34c-3p | MI0000743 | AAUCACUAACCACACGGCCAGG | 3.2661 |
| hsa-miR-361 | MI0000760 | UUAUCAGAAUCUCCAGGGGUAC | 0.1627 |
| hsa-miR-361-3p | MI0000760 | UCCCCCAGGUGUGAUUCUGAUUU | 0.8851 |
| hsa-miR-362 | MI0000762 | AAUCCUUGGAACCUAGGUGUGAGU | 0.4791 |
| hsa-miR-362-3p | MI0000762 | AACACACCUAUUCAAGGAUUCA | 1.8606 |
| hsa-miR-363 | MI0000764 | AAUUGCACGGUAUCCAUCUGUA | 0.4653 |
| hsa-miR-363* | MI0000764 | CGGGUGGAUCACGAUGCAAUUU | 0.1801 |
| hsa-miR-365 | MI0000767 | UAAUGCCCCUAAAAAUCCUUAU | 1.5767 |
| hsa-miR-365 | MI0000769 | UAAUGCCCCUAAAAAUCCUUAU | 3.0907 |
| hsa-miR-367 | MI0000775 | AAUUGCACUUUAGCAAUGGUGA | 0.3978 |
| hsa-miR-367* | MI0000775 | ACUGUUGCUAAUAUGCAACUCU | 1.0428 |
| hsa-miR-368 | MI0000776 | AACAUAGAGGAAAUUCCACGU | 1.2513 |
| hsa-miR-369-3p | MI0000777 | AAUAAUACAUGGUUGAUCUUU | 0.9272 |
| hsa-miR-369-5p | MI0000777 | AGAUCGACCGUGUUAUAUUCGC | 0.1764 |
| hsa-miR-370 | MI0000778 | GCCUGCUGGGGUGGAACCUGGU | 0.2701 |
| hsa-miR-371 | MI0000779 | AAGUGCCGCCAUCUUUUGAGUGU | 0.2456 |
| hsa-miR-371-5p | MI0000779 | ACUCAAACUGUGGGGGCACU | 1.0882 |
| hsa-miR-372 | MI0000780 | AAAGUGCUGCGACAUUUGAGCGU | 1.5108 |
| hsa-miR-373 | MI0000781 | GAAGUGCUUCGAUUUUGGGGUGU | 1.2175 |
| hsa-miR-373* | MI0000781 | ACUCAAAAUGGGGGCGCUUUCC | 0.505 |
| hsa-miR-374 | MI0000782 | UUAUAAUACAACCUGAUAAGUG | 0.0848 |
| hsa-miR-374a* | MI0000782 | CUUAUCAGAUUGUAUUGUAAUU | 1.5158 |
| hsa-miR-374b | MI0005566 | AUAUAAUACAACCUGCUAAGUG | 0.4687 |
| hsa-miR-374b* | MI0005566 | CUUAGCAGGUUGUAUUAUCAUU | 0.5509 |
| hsa-miR-375 | MI0000783 | UUUGUUCGUUCGGCUCGCGUGA | 0.4523 |
| hsa-miR-376a | MI0000784 | AUCAUAGAGGAAAAUCCACGU | 0.7081 |
| hsa-miR-376a | MI0003529 | AUCAUAGAGGAAAAUCCACGU | 0.8018 |
| hsa-miR-376a* | MI0000784 | GUAGAUUCUCCUUCUAUGAGUA | 0.6277 |
| hsa-miR-376b | MI0002466 | AUCAUAGAGGAAAAUCCAUGUU | 0.1445 |
| hsa-miR-377 | MI0000785 | AUCACACAAAGGCAACUUUUGU | 0.2879 |
| hsa-miR-377* | MI0000785 | AGAGGUUGCCCUUGGUGAAUUC | 1.2544 |
| hsa-miR-378 | MI0000786 | CUCCUGACUCCAGGUCCUGUGU | 0.6073 |
| hsa-miR-379 | MI0000787 | UGGUAGACUAUGGAACGUAGG | 0.8652 |
| hsa-miR-379* | MI0000787 | UAUGUAACAUGGUCCACUAACU | 0.7711 |
| hsa-miR-380-3p | MI0000788 | UAUGUAAUAUGGUCCACAUCUU | 0.2156 |
| hsa-miR-380-5p | MI0000788 | UGGUUGACCAUAGAACAUGCGC | 0.3932 |
| hsa-miR-381 | MI0000789 | UAUACAAGGGCAAGCUCUCUGU | 0.6291 |
| hsa-miR-382 | MI0000790 | GAAGUUGUUCGUGGUGGAUUCG | 1.1576 |
| hsa-miR-383 | MI0000791 | AGAUCAGAAGGUGAUUGUGGCU | 0.5024 |
| hsa-miR-384 | MI0001145 | AUUCCUAGAAAUUGUUCAUA | 0.7143 |
| hsa-miR-409-3p | MI0001735 | GAAUGUUGCUCGGUGAACCCCU | 0.5631 |
| hsa-miR-409-5p | MI0001735 | AGGUUACCCGAGCAACUUUGCAU | 0.4581 |
| hsa-miR-410 | MI0002465 | AAUAUAACACAGAUGGCCUGU | 0.328 |
| hsa-miR-411 | MI0003675 | UAGUAGACCGUAUAGCGUACG | 0.096 |
| hsa-miR-411* | MI0003675 | UAUGUAACACGGUCCACUAACC | 0.5844 |
| hsa-miR-412 | MI0002464 | ACUUCACCUGGUCCACUAGCCGU | 0.1215 |
| hsa-miR-421 | MI0003685 | AUCAACAGACAUUAAUUGGGCGC | 0.1717 |
| hsa-miR-422a | MI0001444 | ACUGGACUUAGGGUCAGAAGGC | 0.7769 |
| hsa-miR-422b | MI0000786 | ACUGGACUUGGAGUCAGAAGG | 0.7837 |
| hsa-miR-423 | MI0001445 | AGCUCGGUCUGAGGCCCCUCAGU | 0.5183 |
| hsa-miR-423-5p | MI0001445 | UGAGGGGCAGAGAGCGAGACUUU | 0.47 |
| hsa-miR-424 | MI0001446 | CAGCAGCAAUUCAUGUUUUGAA | 0.0598 |
| hsa-miR-424* | MI0001446 | CAAAACGUGAGGCGCUGCUAU | 0.8775 |
| hsa-miR-425 | MI0001448 | AUCGGGAAUGUCGUGUCCGCCC | 1.1361 |
| hsa-miR-425-5p | MI0001448 | AAUGACACGAUCACUCCCGUUGA | 0.0362 |
| hsa-miR-429 | MI0001641 | UAAUACUGUCUGGUAAAACCGU | 0.3209 |
| hsa-miR-431 | MI0001721 | UGUCUUGCAGGCCGUCAUGCA | 0.1811 |
| hsa-miR-431* | MI0001721 | CAGGUCGUCUUGCAGGGCUUCU | 0.6158 |
| hsa-miR-432 | MI0003133 | UCUUGGAGUAGGUCAUUGGGUGG | 0.419 |
| hsa-miR-432* | MI0003133 | CUGGAUGGCUCCUCCAUGUCU | 0.8279 |
| hsa-miR-433 | MI0001723 | AUCAUGAUGGGCUCCUCGGUGU | 0.2777 |
| hsa-miR-448 | MI0001637 | UUGCAUAUGUAGGAUGUCCCAU | 1.4687 |
| hsa-miR-449 | MI0001648 | UGGCAGUGUAUUGUUAGCUGGU | 0.2771 |
| hsa-miR-449b | MI0003673 | AGGCAGUGUAUUGUUAGCUGGC | 0.0832 |
| hsa-miR-450 | MI0003187 | UUUUGCGAUGUGUUCCUAAUAU | 1.5577 |
| hsa-miR-450 | MI0001652 | UUUUGCGAUGUGUUCCUAAUAU | 1.5971 |
| hsa-miR-450b-3p | MI0005531 | UUGGGAUCAUUUUGCAUCCAUA | 0.208 |
| hsa-miR-450b-5p | MI0005531 | UUUUGCAAUAUGUUCCUGAAUA | 1.03 |
| hsa-miR-451 | MI0001729 | AAACCGUUACCAUUACUGAGUU | 0.3698 |
| hsa-miR-452 | MI0001733 | AACUGUUUGCAGAGGAAACUGA | 1.739 |
| hsa-miR-452* | MI0001733 | CUCAUCUGCAAAGAAGUAAGUG | 0.9151 |
| hsa-miR-453 | MI0001727 | AGGUUGUCCGUGGUGAGUUCGCA | 0.8573 |
| hsa-miR-454-3p | MI0003820 | UAGUGCAAUAUUGCUUAUAGGGU | 0.5849 |
| hsa-miR-454-5p | MI0003820 | ACCCUAUCAAUAUUGUCUCUGC | 0.4129 |
| hsa-miR-455 | MI0003513 | UAUGUGCCUUUGGACUACAUCG | 0.3983 |
| hsa-miR-455-3p | MI0003513 | GCAGUCCAUGGGCAUAUACAC | 1.3999 |
| hsa-miR-483 | MI0002467 | UCACUCCUCUCCUCCCGUCUU | 0.9017 |
| hsa-miR-483-5p | MI0002467 | AAGACGGGAGGAAAGAAGGGAG | 2.7952 |
| hsa-miR-484 | MI0002468 | UCAGGCUCAGUCCCCUCCCGAU | 0.7085 |
| hsa-miR-485-3p | MI0002469 | GUCAUACACGGCUCUCCUCUCU | 0.9595 |
| hsa-miR-485-5p | MI0002469 | AGAGGCUGGCCGUGAUGAAUUC | 0.2654 |
| hsa-miR-486 | MI0002470 | UCCUGUACUGAGCUGCCCCGAG | 0.5796 |
| hsa-miR-486-3p | MI0002470 | CGGGGCAGCUCAGUACAGGAU | 0.2227 |
| hsa-miR-487 | MI0002471 | AAUCAUACAGGGACAUCCAGUU | 0.3021 |
| hsa-miR-487b | MI0003530 | AAUCGUACAGGGUCAUCCACUU | 0.1318 |
| hsa-miR-488 | MI0003123 | UUGAAAGGCUAUUUCUUGGUC | 0.6974 |
| hsa-miR-488* | MI0003123 | CCCAGAUAAUGGCACUCUCAA | 0.0491 |
| hsa-miR-489 | MI0003124 | GUGACAUCACAUAUACGGCAGC | 1.345 |
| hsa-miR-490 | MI0003125 | CAACCUGGAGGACUCCAUGCUG | 0.8377 |
| hsa-miR-490-5p | MI0003125 | CCAUGGAUCUCCAGGUGGGU | 0.6037 |
| hsa-miR-491 | MI0003126 | AGUGGGGAACCCUUCCAUGAGG | 0.1059 |
| hsa-miR-491-3p | MI0003126 | CUUAUGCAAGAUUCCCUUCUAC | 0.4335 |
| hsa-miR-492 | MI0003131 | AGGACCUGCGGGACAAGAUUCUU | 0.6291 |
| hsa-miR-493 | MI0003132 | UUGUACAUGGUAGGCUUUCAUU | 0.0808 |
| hsa-miR-493-3p | MI0003132 | UGAAGGUCUACUGUGUGCCAGG | 3.6504 |
| hsa-miR-494 | MI0003134 | UGAAACAUACACGGGAAACCUC | 0.6502 |
| hsa-miR-495 | MI0003135 | AAACAAACAUGGUGCACUUCUU | 1.7241 |
| hsa-miR-496 | MI0003136 | UGAGUAUUACAUGGCCAAUCUC | 1.003 |
| hsa-miR-497 | MI0003138 | CAGCAGCACACUGUGGUUUGU | 0.8885 |
| hsa-miR-497* | MI0003138 | CAAACCACACUGUGGUGUUAGA | 0.8929 |
| hsa-miR-498 | MI0003142 | UUUCAAGCCAGGGGGCGUUUUUC | 0.4643 |
| hsa-miR-499 | MI0003183 | UUAAGACUUGCAGUGAUGUUU | 0.7577 |
| hsa-miR-499-3p | MI0003183 | AACAUCACAGCAAGUCUGUGCU | 2.291 |
| hsa-miR-500 | MI0003184 | UAAUCCUUGCUACCUGGGUGAGA | 0.4134 |
| hsa-miR-500* | MI0003184 | AUGCACCUGGGCAAGGAUUCUG | 0.2677 |
| hsa-miR-501 | MI0003185 | AAUCCUUUGUCCCUGGGUGAGA | 0.171 |
| hsa-miR-501-3p | MI0003185 | AAUGCACCCGGGCAAGGAUUCU | 0.5495 |
| hsa-miR-502 | MI0003186 | AUCCUUGCUAUCUGGGUGCUA | 0.2371 |
| hsa-miR-502-3p | MI0003186 | AAUGCACCUGGGCAAGGAUUCA | 1.2455 |
| hsa-miR-503 | MI0003188 | UAGCAGCGGGAACAGUUCUGCAG | 0.3636 |
| hsa-miR-504 | MI0003189 | AGACCCUGGUCUGCACUCUAUC | 1.6662 |
| hsa-miR-505 | MI0003190 | CGUCAACACUUGCUGGUUUCCU | 1.4422 |
| hsa-miR-505* | MI0003190 | GGGAGCCAGGAAGUAUUGAUGU | 0.452 |
| hsa-miR-506 | MI0003193 | UAAGGCACCCUUCUGAGUAGA | 0.1881 |
| hsa-miR-507 | MI0003194 | UUUUGCACCUUUUGGAGUGAA | 0.121 |
| hsa-miR-508 | MI0003195 | UGAUUGUAGCCUUUUGGAGUAGA | 0.2785 |
| hsa-miR-508-5p | MI0003195 | UACUCCAGAGGGCGUCACUCAUG | 1.3985 |
| hsa-miR-509 | MI0003196 | UGAUUGGUACGUCUGUGGGUAG | 0.8842 |
| hsa-miR-509-3-5p | MI0005717 | UACUGCAGACGUGGCAAUCAUG | 2.6973 |
| hsa-miR-509-3p | MI0005530 | UGAUUGGUACGUCUGUGGGUAG | 0.898 |
| hsa-miR-509-3p | MI0005717 | UGAUUGGUACGUCUGUGGGUAG | 0.9896 |
| hsa-miR-509-5p | MI0003196 | UACUGCAGACAGUGGCAAUCA | 1.9922 |
| hsa-miR-509-5p | MI0005530 | UACUGCAGACAGUGGCAAUCA | 2.2415 |
| hsa-miR-510 | MI0003197 | UACUCAGGAGAGUGGCAAUCAC | 1.0654 |
| hsa-miR-511 | MI0003127 | GUGUCUUUUGCUCUGCAGUCA | 0.2297 |
| hsa-miR-511 | MI0003128 | GUGUCUUUUGCUCUGCAGUCA | 0.2928 |
| hsa-miR-512-3p | MI0003140 | AAGUGCUGUCAUAGCUGAGGUC | 0.2394 |
| hsa-miR-512-3p | MI0003141 | AAGUGCUGUCAUAGCUGAGGUC | 0.2679 |
| hsa-miR-512-5p | MI0003140 | CACUCAGCCUUGAGGGCACUUUC | 0.2892 |
| hsa-miR-512-5p | MI0003141 | CACUCAGCCUUGAGGGCACUUUC | 0.7693 |
| hsa-miR-513 | MI0003191 | UUCACAGGGAGGUGUCAU | 1.941 |
| hsa-miR-513 | MI0003192 | UUCACAGGGAGGUGUCAU | 2.8236 |
| hsa-miR-513a-3p | MI0003191 | UAAAUUUCACCUUUCUGAGAAGG | 1.672 |
| hsa-miR-513a-3p | MI0003192 | UAAAUUUCACCUUUCUGAGAAGG | 1.9696 |
| hsa-miR-513b | MI0006648 | UUCACAAGGAGGUGUCAUUUAU | 1.2838 |
| hsa-miR-513c | MI0006649 | UUCUCAAGGAGGUGUCGUUUAU | 1.5284 |
| hsa-miR-514 | MI0003198 | AUUGACACUUCUGUGAGUAGA | 0.6886 |
| hsa-miR-514 | MI0003199 | AUUGACACUUCUGUGAGUAGA | 0.738 |
| hsa-miR-514 | MI0003200 | AUUGACACUUCUGUGAGUAGA | 1.1284 |
| hsa-miR-515-3p | MI0003144 | GAGUGCCUUCUUUUGGAGCGUU | 0.2529 |
| hsa-miR-515-3p | MI0003147 | GAGUGCCUUCUUUUGGAGCGUU | 0.5335 |
| hsa-miR-515-5p | MI0003144 | UUCUCCAAAAGAAAGCACUUUCUG | 0.4636 |
| hsa-miR-515-5p | MI0003147 | UUCUCCAAAAGAAAGCACUUUCUG | 0.5093 |
| hsa-miR-516-3p | MI0003167 | UGCUUCCUUUCAGAGGGU | 0.1355 |
| hsa-miR-516-3p | MI0003172 | UGCUUCCUUUCAGAGGGU | 0.2409 |
| hsa-miR-516-3p | MI0003180 | UGCUUCCUUUCAGAGGGU | 0.6185 |
| hsa-miR-516-3p | MI0003181 | UGCUUCCUUUCAGAGGGU | 0.8135 |
| hsa-miR-516-5p | MI0003167 | AUCUGGAGGUAAGAAGCACUUU | 0.2777 |
| hsa-miR-516-5p | MI0003172 | AUCUGGAGGUAAGAAGCACUUU | 0.5347 |
| hsa-miR-516a-5p | MI0003180 | UUCUCGAGGAAAGAAGCACUUUC | 1.0699 |
| hsa-miR-516a-5p | MI0003181 | UUCUCGAGGAAAGAAGCACUUUC | 1.2766 |
| hsa-miR-517* | MI0003161 | CCUCUAGAUGGAAGCACUGUCU | 0.2852 |
| hsa-miR-517* | MI0003165 | CCUCUAGAUGGAAGCACUGUCU | 0.3115 |
| hsa-miR-517* | MI0003174 | CCUCUAGAUGGAAGCACUGUCU | 0.4384 |
| hsa-miR-517a | MI0003161 | AUCGUGCAUCCCUUUAGAGUGU | 0.8355 |
| hsa-miR-517b | MI0003165 | UCGUGCAUCCCUUUAGAGUGUU | 0.5555 |
| hsa-miR-517c | MI0003174 | AUCGUGCAUCCUUUUAGAGUGU | 0.5394 |
| hsa-miR-518a | MI0003170 | GAAAGCGCUUCCCUUUGCUGGA | 0.4209 |
| hsa-miR-518a | MI0003173 | GAAAGCGCUUCCCUUUGCUGGA | 0.6749 |
| hsa-miR-518a-5p | MI0003170 | CUGCAAAGGGAAGCCCUUUC | 0.9107 |
| hsa-miR-518a-5p | MI0003173 | CUGCAAAGGGAAGCCCUUUC | 1.0011 |
| hsa-miR-518b | MI0003156 | CAAAGCGCUCCCCUUUAGAGGU | 0.0707 |
| hsa-miR-518c | MI0003159 | CAAAGCGCUUCUCUUUAGAGUGU | 0.373 |
| hsa-miR-518c* | MI0003159 | UCUCUGGAGGGAAGCACUUUCUG | 0.1897 |
| hsa-miR-518d | MI0003171 | CAAAGCGCUUCCCUUUGGAGC | 0.335 |
| hsa-miR-518d-5p | MI0003171 | CUCUAGAGGGAAGCACUUUCUG | 0.3718 |
| hsa-miR-518e | MI0003169 | AAAGCGCUUCCCUUCAGAGUG | 0.2518 |
| hsa-miR-518e* | MI0003169 | CUCUAGAGGGAAGCGCUUUCUG | 1.6856 |
| hsa-miR-518f | MI0003154 | GAAAGCGCUUCUCUUUAGAGG | 0.4877 |
| hsa-miR-518f* | MI0003154 | CUCUAGAGGGAAGCACUUUCUC | 0.3031 |
| hsa-miR-519a | MI0003178 | AAAGUGCAUCCUUUUAGAGUGU | 2.249 |
| hsa-miR-519a | MI0003182 | AAAGUGCAUCCUUUUAGAGUGU | 2.3529 |
| hsa-miR-519a* | MI0003178 | CUCUAGAGGGAAGCGCUUUCUG | 0.3551 |
| hsa-miR-519b | MI0003151 | AAAGUGCAUCCUUUUAGAGGUU | 1.0374 |
| hsa-miR-519b-5p | MI0003151 | CUCUAGAGGGAAGCGCUUUCUG | 0.5355 |
| hsa-miR-519c | MI0003148 | AAAGUGCAUCUUUUUAGAGGAU | 0.8077 |
| hsa-miR-519d | MI0003162 | CAAAGUGCCUCCCUUUAGAGUG | 1.0619 |
| hsa-miR-519e | MI0003145 | AAGUGCCUCCUUUUAGAGUGUU | 0.8291 |
| hsa-miR-519e* | MI0003145 | UUCUCCAAAAGGGAGCACUUUC | 0.5519 |
| hsa-miR-520a | MI0003149 | AAAGUGCUUCCCUUUGGACUGU | 0.1369 |
| hsa-miR-520a* | MI0003149 | CUCCAGAGGGAAGUACUUUCU | 0.169 |
| hsa-miR-520b | MI0003155 | AAAGUGCUUCCUUUUAGAGGG | 0.2861 |
| hsa-miR-520c | MI0003158 | AAAGUGCUUCCUUUUAGAGGGU | 1.3027 |
| hsa-miR-520c-5p | MI0003158 | CUCUAGAGGGAAGCACUUUCUG | 0.3491 |
| hsa-miR-520d | MI0003164 | AAAGUGCUUCUCUUUGGUGGGU | 1.0146 |
| hsa-miR-520d* | MI0003164 | CUACAAAGGGAAGCCCUUUC | 2.3419 |
| hsa-miR-520e | MI0003143 | AAAGUGCUUCCUUUUUGAGGG | 0.7123 |
| hsa-miR-520f | MI0003146 | AAGUGCUUCCUUUUAGAGGGUU | 0.3633 |
| hsa-miR-520g | MI0003166 | ACAAAGUGCUUCCCUUUAGAGUGU | 1.6768 |
| hsa-miR-520h | MI0003175 | ACAAAGUGCUUCCCUUUAGAGU | 0.9084 |
| hsa-miR-521 | MI0003163 | AACGCACUUCCCUUUAGAGUGU | 0.3146 |
| hsa-miR-521 | MI0003176 | AACGCACUUCCCUUUAGAGUGU | 0.6733 |
| hsa-miR-522 | MI0003177 | AAAAUGGUUCCCUUUAGAGUGU | 0.1149 |
| hsa-miR-522* | MI0003177 | CUCUAGAGGGAAGCGCUUUCUG | 0.4163 |
| hsa-miR-523 | MI0003153 | GAACGCGCUUCCCUAUAGAGGGU | 0.4816 |
| hsa-miR-523* | MI0003153 | CUCUAGAGGGAAGCGCUUUCUG | 0.5249 |
| hsa-miR-524 | MI0003160 | GAAGGCGCUUCCCUUUGGAGU | 1.2428 |
| hsa-miR-524* | MI0003160 | CUACAAAGGGAAGCACUUUCUC | 0.532 |
| hsa-miR-525 | MI0003152 | CUCCAGAGGGAUGCACUUUCU | 0.0971 |
| hsa-miR-525* | MI0003152 | GAAGGCGCUUCCCUUUAGAGCG | 1.2364 |
| hsa-miR-526a | MI0003157 | CUCUAGAGGGAAGCACUUUCUG | 0.4148 |
| hsa-miR-526a | MI0003168 | CUCUAGAGGGAAGCACUUUCUG | 0.5483 |
| hsa-miR-526b | MI0003150 | CUCUUGAGGGAAGCACUUUCUGU | 1.5491 |
| hsa-miR-526b* | MI0003150 | GAAAGUGCUUCCUUUUAGAGGC | 0.7186 |
| hsa-miR-526c | MI0003148 | CUCUAGAGGGAAGCGCUUUCUG | 0.2523 |
| hsa-miR-527 | MI0003179 | CUGCAAAGGGAAGCCCUUUC | 0.4396 |
| hsa-miR-532 | MI0003205 | CAUGCCUUGAGUGUAGGACCGU | 0.1508 |
| hsa-miR-532-3p | MI0003205 | CCUCCCACACCCAAGGCUUGCA | 0.9601 |
| hsa-miR-539 | MI0003514 | GGAGAAAUUAUCCUUGGUGUGU | 0.1021 |
| hsa-miR-541 | MI0005539 | UGGUGGGCACAGAAUCUGGACU | 0.076 |
| hsa-miR-541* | MI0005539 | AAAGGAUUCUGCUGUCGGUCCCACU | 0.5432 |
| hsa-miR-542-3p | MI0003686 | UGUGACAGAUUGAUAACUGAAA | 0.7193 |
| hsa-miR-542-5p | MI0003686 | UCGGGGAUCAUCAUGUCACGAGA | 0.8553 |
| hsa-miR-543 | MI0005565 | AAACAUUCGCGGUGCACUUCUU | 0.4684 |
| hsa-miR-544 | MI0003515 | AUUCUGCAUUUUUAGCAAGUUC | 1.3156 |
| hsa-miR-545 | MI0003516 | UCAGCAAACAUUUAUUGUGUGC | 1.4146 |
| hsa-miR-545* | MI0003516 | UCAGUAAAUGUUUAUUAGAUGA | 0.5769 |
| hsa-miR-548a | MI0003593 | CAAAACUGGCAAUUACUUUUGC | 0.6365 |
| hsa-miR-548a | MI0003598 | CAAAACUGGCAAUUACUUUUGC | 1.1899 |
| hsa-miR-548a | MI0003612 | CAAAACUGGCAAUUACUUUUGC | 1.3664 |
| hsa-miR-548a-5p | MI0003612 | AAAAGUAAUUGCGAGUUUUACC | 0.3298 |
| hsa-miR-548b | MI0003596 | CAAGAACCUCAGUUGCUUUUGU | 1.035 |
| hsa-miR-548b-5p | MI0003596 | AAAAGUAAUUGUGGUUUUGGCC | 0.427 |
| hsa-miR-548c | MI0003630 | CAAAAAUCUCAAUUACUUUUGC | 1.9961 |
| hsa-miR-548c-5p | MI0003630 | AAAAGUAAUUGCGGUUUUUGCC | 0.4975 |
| hsa-miR-548d | MI0003668 | CAAAAACCACAGUUUCUUUUGC | 0.0998 |
| hsa-miR-548d | MI0003671 | CAAAAACCACAGUUUCUUUUGC | 0.1505 |
| hsa-miR-548d-5p | MI0003668 | AAAAGUAAUUGUGGUUUUUGCC | 0.7169 |
| hsa-miR-548d-5p | MI0003671 | AAAAGUAAUUGUGGUUUUUGCC | 0.7583 |
| hsa-miR-549 | MI0003679 | UGACAACUAUGGAUGAGCUCU | 0.2582 |
| hsa-miR-550 | MI0003600 | UGUCUUACUCCCUCAGGCACAU | 0.4543 |
| hsa-miR-550 | MI0003601 | UGUCUUACUCCCUCAGGCACAU | 0.7735 |
| hsa-miR-550 | MI0003600 | AGUGCCUGAGGGAGUAAGAGCCC | 1.1116 |
| hsa-miR-550 | MI0003601 | AGUGCCUGAGGGAGUAAGAGCCC | 2.3153 |
| hsa-miR-551a | MI0003556 | GCGACCCACUCUUGGUUUCCA | 0.5904 |
| hsa-miR-551b | MI0003575 | GCGACCCAUACUUGGUUUCAG | 0.3558 |
| hsa-miR-551b* | MI0003575 | GAAAUCAAGCGUGGGUGAGACC | 0.6727 |
| hsa-miR-552 | MI0003557 | AACAGGUGACUGGUUAGACAA | 0.4781 |
| hsa-miR-553 | MI0003558 | AAAACGGUGAGAUUUUGUUUU | 0.2496 |
| hsa-miR-554 | MI0003559 | GCUAGUCCUGACUCAGCCAGU | 0.4317 |
| hsa-miR-555 | MI0003561 | AGGGUAAGCUGAACCUCUGAU | 0.4303 |
| hsa-miR-556 | MI0003562 | GAUGAGCUCAUUGUAAUAUGAG | 0.5204 |
| hsa-miR-556-3p | MI0003562 | AUAUUACCAUUAGCUCAUCUUU | 1.3814 |
| hsa-miR-557 | MI0003563 | GUUUGCACGGGUGGGCCUUGUCU | 0.2016 |
| hsa-miR-558 | MI0003564 | UGAGCUGCUGUACCAAAAU | 0.2559 |
| hsa-miR-559 | MI0003565 | UAAAGUAAAUAUGCACCAAAA | 0.1898 |
| hsa-miR-561 | MI0003567 | CAAAGUUUAAGAUCCUUGAAGU | 0.1864 |
| hsa-miR-562 | MI0003568 | AAAGUAGCUGUACCAUUUGC | 0.1389 |
| hsa-miR-563 | MI0003569 | AGGUUGACAUACGUUUCCC | 0.369 |
| hsa-miR-564 | MI0003570 | AGGCACGGUGUCAGCAGGC | 0.5016 |
| hsa-miR-566 | MI0003572 | GGGCGCCUGUGAUCCCAAC | 0.2969 |
| hsa-miR-567 | MI0003573 | AGUAUGUUCUUCCAGGACAGAAC | 0.1914 |
| hsa-miR-568 | MI0003574 | AUGUAUAAAUGUAUACACAC | 0.4066 |
| hsa-miR-569 | MI0003576 | AGUUAAUGAAUCCUGGAAAGU | 0.2262 |
| hsa-miR-570 | MI0003577 | CGAAAACAGCAAUUACCUUUGC | 1.5745 |
| hsa-miR-571 | MI0003578 | UGAGUUGGCCAUCUGAGUGAG | 0.2247 |
| hsa-miR-572 | MI0003579 | GUCCGCUCGGCGGUGGCCCA | 0.2628 |
| hsa-miR-573 | MI0003580 | CUGAAGUGAUGUGUAACUGAUCAG | 0.3834 |
| hsa-miR-574 | MI0003581 | CACGCUCAUGCACACACCCACA | 1.0681 |
| hsa-miR-574-5p | MI0003581 | UGAGUGUGUGUGUGUGAGUGUGU | 0.1579 |
| hsa-miR-575 | MI0003582 | GAGCCAGUUGGACAGGAGC | 0.1407 |
| hsa-miR-576 | MI0003583 | AUUCUAAUUUCUCCACGUCUUU | 1.4364 |
| hsa-miR-576-3p | MI0003583 | AAGAUGUGGAAAAAUUGGAAUC | 2.9457 |
| hsa-miR-577 | MI0003584 | UAGAUAAAAUAUUGGUACCUG | 1.473 |
| hsa-miR-578 | MI0003585 | CUUCUUGUGCUCUAGGAUUGU | 3.5754 |
| hsa-miR-579 | MI0003586 | UUCAUUUGGUAUAAACCGCGAUU | 0.5812 |
| hsa-miR-580 | MI0003587 | UUGAGAAUGAUGAAUCAUUAGG | 2.5665 |
| hsa-miR-581 | MI0003588 | UCUUGUGUUCUCUAGAUCAGU | 4.2371 |
| hsa-miR-582 | MI0003589 | UUACAGUUGUUCAACCAGUUACU | 0.7155 |
| hsa-miR-582-3p | MI0003589 | UAACUGGUUGAACAACUGAACC | 1.2109 |
| hsa-miR-583 | MI0003590 | CAAAGAGGAAGGUCCCAUUAC | 1.6362 |
| hsa-miR-584 | MI0003591 | UUAUGGUUUGCCUGGGACUGAG | 0.6301 |
| hsa-miR-585 | MI0003592 | UGGGCGUAUCUGUAUGCUA | 0.5556 |
| hsa-miR-586 | MI0003594 | UAUGCAUUGUAUUUUUAGGUCC | 2.4598 |
| hsa-miR-587 | MI0003595 | UUUCCAUAGGUGAUGAGUCAC | 0.8758 |
| hsa-miR-588 | MI0003597 | UUGGCCACAAUGGGUUAGAAC | 0.5614 |
| hsa-miR-589 | MI0003599 | UGAGAACCACGUCUGCUCUGAG | 0.6356 |
| hsa-miR-589* | MI0003599 | UCAGAACAAAUGCCGGUUCCCAGA | 1.2816 |
| hsa-miR-590 | MI0003602 | GAGCUUAUUCAUAAAAGUGCAG | 2.2598 |
| hsa-miR-590-3p | MI0003602 | UAAUUUUAUGUAUAAGCUAGU | 0.6473 |
| hsa-miR-591 | MI0003603 | AGACCAUGGGUUCUCAUUGU | 0.561 |
| hsa-miR-592 | MI0003604 | UUGUGUCAAUAUGCGAUGAUGU | 1.9267 |
| hsa-miR-593 | MI0003605 | UGUCUCUGCUGGGGUUUCU | 0.2032 |
| hsa-miR-593* | MI0003605 | AGGCACCAGCCAGGCAUUGCUCAGC | 2.4967 |
| hsa-miR-595 | MI0003607 | GAAGUGUGCCGUGGUGUGUCU | 1.6342 |
| hsa-miR-596 | MI0003608 | AAGCCUGCCCGGCUCCUCGGG | 1.0716 |
| hsa-miR-597 | MI0003609 | UGUGUCACUCGAUGACCACUGU | 2.1446 |
| hsa-miR-598 | MI0003610 | UACGUCAUCGUUGUCAUCGUCA | 0.7798 |
| hsa-miR-599 | MI0003611 | GUUGUGUCAGUUUAUCAAAC | 1.5129 |
| hsa-miR-600 | MI0003613 | ACUUACAGACAAGAGCCUUGCUC | 2.4235 |
| hsa-miR-601 | MI0003614 | UGGUCUAGGAUUGUUGGAGGAG | 1.6903 |
| hsa-miR-602 | MI0003615 | GACACGGGCGACAGCUGCGGCCC | 2.2336 |
| hsa-miR-603 | MI0003616 | CACACACUGCAAUUACUUUUGC | 2.1803 |
| hsa-miR-604 | MI0003617 | AGGCUGCGGAAUUCAGGAC | 0.321 |
| hsa-miR-605 | MI0003618 | UAAAUCCCAUGGUGCCUUCUCCU | 1.4013 |
| hsa-miR-606 | MI0003619 | AAACUACUGAAAAUCAAAGAU | 0.6186 |
| hsa-miR-607 | MI0003620 | GUUCAAAUCCAGAUCUAUAAC | 1.6514 |
| hsa-miR-608 | MI0003621 | AGGGGUGGUGUUGGGACAGCUCCGU | 0.5797 |
| hsa-miR-609 | MI0003622 | AGGGUGUUUCUCUCAUCUCU | 0.2082 |
| hsa-miR-610 | MI0003623 | UGAGCUAAAUGUGUGCUGGGA | 1.4505 |
| hsa-miR-611 | MI0003624 | GCGAGGACCCCUCGGGGUCUGAC | 0.6427 |
| hsa-miR-612 | MI0003625 | GCUGGGCAGGGCUUCUGAGCUCCUU | 1.1897 |
| hsa-miR-613 | MI0003626 | AGGAAUGUUCCUUCUUUGCC | 0.2971 |
| hsa-miR-614 | MI0003627 | GAACGCCUGUUCUUGCCAGGUGG | 1.5716 |
| hsa-miR-615 | MI0003628 | UCCGAGCCUGGGUCUCCCUCUU | 0.9694 |
| hsa-miR-615-5p | MI0003628 | GGGGGUCCCCGGUGCUCGGAUC | 0.8642 |
| hsa-miR-616 | MI0003629 | AGUCAUUGGAGGGUUUGAGCAG | 0.5182 |
| hsa-miR-616* | MI0003629 | ACUCAAAACCCUUCAGUGACUU | 1.2561 |
| hsa-miR-617 | MI0003631 | AGACUUCCCAUUUGAAGGUGGC | 2.1857 |
| hsa-miR-618 | MI0003632 | AAACUCUACUUGUCCUUCUGAGU | 0.6543 |
| hsa-miR-619 | MI0003633 | GACCUGGACAUGUUUGUGCCCAGU | 1.1474 |
| hsa-miR-620 | MI0003634 | AUGGAGAUAGAUAUAGAAAU | 1.7664 |
| hsa-miR-621 | MI0003635 | GGCUAGCAACAGCGCUUACCU | 2.4721 |
| hsa-miR-622 | MI0003636 | ACAGUCUGCUGAGGUUGGAGC | 4.189 |
| hsa-miR-623 | MI0003637 | AUCCCUUGCAGGGGCUGUUGGGU | 0.3836 |
| hsa-miR-624 | MI0003638 | CACAAGGUAUUGGUAUUACCU | 14.2983 |
| hsa-miR-624* | MI0003638 | UAGUACCAGUACCUUGUGUUCA | 0.8819 |
| hsa-miR-625 | MI0003639 | AGGGGGAAAGUUCUAUAGUCC | 0.1564 |
| hsa-miR-625* | MI0003639 | GACUAUAGAACUUUCCCCCUCA | 0.5812 |
| hsa-miR-626 | MI0003640 | AGCUGUCUGAAAAUGUCUU | 1.7065 |
| hsa-miR-627 | MI0003641 | GUGAGUCUCUAAGAAAAGAGGA | 1.8108 |
| hsa-miR-628 | MI0003642 | UCUAGUAAGAGUGGCAGUCGA | 1.2186 |
| hsa-miR-628-5p | MI0003642 | AUGCUGACAUAUUUACUAGAGG | 2.1363 |
| hsa-miR-629 | MI0003643 | UGGGUUUACGUUGGGAGAACU | 0.6274 |
| hsa-miR-629* | MI0003643 | GUUCUCCCAACGUAAGCCCAGC | 3.9995 |
| hsa-miR-630 | MI0003644 | AGUAUUCUGUACCAGGGAAGGU | 1.6478 |
| hsa-miR-631 | MI0003645 | AGACCUGGCCCAGACCUCAGC | 1.2183 |
| hsa-miR-632 | MI0003647 | GUGUCUGCUUCCUGUGGGA | 1.8149 |
| hsa-miR-633 | MI0003648 | CUAAUAGUAUCUACCACAAUAAA | 0.288 |
| hsa-miR-634 | MI0003649 | AACCAGCACCCCAACUUUGGAC | 0.1216 |
| hsa-miR-635 | MI0003650 | ACUUGGGCACUGAAACAAUGUCC | 0.0231 |
| hsa-miR-636 | MI0003651 | UGUGCUUGCUCGUCCCGCCCGCA | 1.2514 |
| hsa-miR-637 | MI0003652 | ACUGGGGGCUUUCGGGCUCUGCGU | 0.0672 |
| hsa-miR-638 | MI0003653 | AGGGAUCGCGGGCGGGUGGCGGCCU | 0.1158 |
| hsa-miR-639 | MI0003654 | AUCGCUGCGGUUGCGAGCGCUGU | 0.1653 |
| hsa-miR-640 | MI0003655 | AUGAUCCAGGAACCUGCCUCU | 0.1621 |
| hsa-miR-641 | MI0003656 | AAAGACAUAGGAUAGAGUCACCUC | 0.3116 |
| hsa-miR-642 | MI0003657 | GUCCCUCUCCAAAUGUGUCUUG | 0.1778 |
| hsa-miR-643 | MI0003658 | ACUUGUAUGCUAGCUCAGGUAG | 0.3276 |
| hsa-miR-644 | MI0003659 | AGUGUGGCUUUCUUAGAGC | 0.1091 |
| hsa-miR-645 | MI0003660 | UCUAGGCUGGUACUGCUGA | 0.1376 |
| hsa-miR-646 | MI0003661 | AAGCAGCUGCCUCUGAGGC | 0.068 |
| hsa-miR-647 | MI0003662 | GUGGCUGCACUCACUUCCUUC | 0.2096 |
| hsa-miR-648 | MI0003663 | AAGUGUGCAGGGCACUGGU | 0.0649 |
| hsa-miR-649 | MI0003664 | AAACCUGUGUUGUUCAAGAGUC | 0.1049 |
| hsa-miR-650 | MI0003665 | AGGAGGCAGCGCUCUCAGGAC | 0.0641 |
| hsa-miR-651 | MI0003666 | UUUAGGAUAAGCUUGACUUUUG | 0.2073 |
| hsa-miR-652 | MI0003667 | AAUGGCGCCACUAGGGUUGUG | 1.5249 |
| hsa-miR-653 | MI0003674 | GUGUUGAAACAAUCUCUACUG | 0.9408 |
| hsa-miR-654 | MI0003676 | UGGUGGGCCGCAGAACAUGUGC | 0.073 |
| hsa-miR-654-3p | MI0003676 | UAUGUCUGCUGACCAUCACCUU | 3.2914 |
| hsa-miR-655 | MI0003677 | AUAAUACAUGGUUAACCUCUUU | 0.3587 |
| hsa-miR-656 | MI0003678 | AAUAUUAUACAGUCAACCUCU | 0.2071 |
| hsa-miR-657 | MI0003681 | GGCAGGUUCUCACCCUCUCUAGG | 0.3942 |
| hsa-miR-658 | MI0003682 | GGCGGAGGGAAGUAGGUCCGUUGGU | 0.157 |
| hsa-miR-659 | MI0003683 | CUUGGUUCAGGGAGGGUCCCCA | 0.344 |
| hsa-miR-660 | MI0003684 | UACCCAUUGCAUAUCGGAGUUG | 0.1496 |
| hsa-miR-661 | MI0003669 | UGCCUGGGUCUCUGGCCUGCGCGU | 0.2311 |
| hsa-miR-662 | MI0003670 | UCCCACGUUGUGGCCCAGCAG | 0.5284 |
| hsa-miR-663 | MI0003672 | AGGCGGGGCGCCGCGGGACCGC | 0.2378 |
| hsa-miR-665 | MI0005563 | ACCAGGAGGCUGAGGCCCCU | 1.0529 |
| hsa-miR-668 | MI0003761 | UGUCACUCGGCUCGGCCCACUAC | 0.3272 |
| hsa-miR-671 | MI0003760 | AGGAAGCCCUGGAGGGGCUGGAG | 0.8527 |
| hsa-miR-671-3p | MI0003760 | UCCGGUUCUCAGGGCUCCACC | 0.1421 |
| hsa-miR-675 | MI0005416 | UGGUGCGGAGAGGGCCCACAGUG | 1.2081 |
| hsa-miR-7 | MI0000264 | UGGAAGACUAGUGAUUUUGUUGU | 0.1317 |
| hsa-miR-7 | MI0000265 | UGGAAGACUAGUGAUUUUGUUGU | 0.1759 |
| hsa-miR-7 | MI0000263 | UGGAAGACUAGUGAUUUUGUUGU | 0.9273 |
| hsa-miR-708 | MI0005543 | AAGGAGCUUACAAUCUAGCUGGG | 0.4271 |
| hsa-miR-708* | MI0005543 | CAACUAGACUGUGAGCUUCUAG | 0.4997 |
| hsa-miR-7-1* | MI0000263 | CAACAAAUCACAGUCUGCCAUA | 0.8284 |
| hsa-miR-7-2* | MI0000264 | CAACAAAUCCCAGUCUACCUAA | 1.7203 |
| hsa-miR-744 | MI0005559 | UGCGGGGCUAGGGCUAACAGCA | 0.2672 |
| hsa-miR-744* | MI0005559 | CUGUUGCCACUAACCUCAACCU | 0.7627 |
| hsa-miR-758 | MI0003757 | UUUGUGACCUGGUCCACUAACC | 3.2587 |
| hsa-miR-760 | MI0005567 | CGGCUCUGGGUCUGUGGGGA | 0.4031 |
| hsa-miR-765 | MI0005116 | UGGAGGAGAAGGAAGGUGAUG | 0.18 |
| hsa-miR-766 | MI0003836 | ACUCCAGCCCCACAGCCUCAGC | 2.2333 |
| hsa-miR-767-3p | MI0003763 | UCUGCUCAUACCCCAUGGUUUCU | 0.5216 |
| hsa-miR-767-5p | MI0003763 | UGCACCAUGGUUGUCUGAGCAUG | 0.4651 |
| hsa-miR-768-3p | MI0005117 | UCACAAUGCUGACACUCAAACUGCUGAC | 0.7059 |
| hsa-miR-768-5p | MI0005117 | GUUGGAGGAUGAAAGUACGGAGUGAU | 0.4194 |
| hsa-miR-769-3p | MI0003834 | CUGGGAUCUCCGGGGUCUUGGUU | 0.4755 |
| hsa-miR-769-5p | MI0003834 | UGAGACCUCUGGGUUCUGAGCU | 0.8035 |
| hsa-miR-770-5p | MI0005118 | UCCAGUACCACGUGUCAGGGCCA | 0.2244 |
| hsa-miR-801 | MI0005202 | GAUUGCUCUGCGUGCGGAAUCGAC | 0.4528 |
| hsa-miR-802 | MI0003906 | CAGUAACAAAGAUUCAUCCUUGU | 0.5195 |
| hsa-miR-873 | MI0005564 | GCAGGAACUUGUGAGUCUCCU | 0.2929 |
| hsa-miR-874 | MI0005532 | CUGCCCUGGCCCGAGGGACCGA | 0.2212 |
| hsa-miR-875-3p | MI0005541 | CCUGGAAACACUGAGGUUGUG | 0.5157 |
| hsa-miR-875-5p | MI0005541 | UAUACCUCAGUUUUAUCAGGUG | 0.7139 |
| hsa-miR-876-3p | MI0005542 | UGGUGGUUUACAAAGUAAUUCA | 1.2368 |
| hsa-miR-876-5p | MI0005542 | UGGAUUUCUUUGUGAAUCACCA | 0.2755 |
| hsa-miR-877 | MI0005561 | GUAGAGGAGAUGGCGCAGGG | 1.0741 |
| hsa-miR-877* | MI0005561 | UCCUCUUCUCCCUCCUCCCAG | 5.5371 |
| hsa-miR-885-3p | MI0005560 | AGGCAGCGGGGUGUAGUGGAUA | 0.143 |
| hsa-miR-885-5p | MI0005560 | UCCAUUACACUACCCUGCCUCU | 0.3652 |
| hsa-miR-886-3p | MI0005527 | CGCGGGUGCUUACUGACCCUU | 0.4031 |
| hsa-miR-886-5p | MI0005527 | CGGGUCGGAGUUAGCUCAAGCGG | 0.6642 |
| hsa-miR-887 | MI0005562 | GUGAACGGGCGCCAUCCCGAGG | 0.2253 |
| hsa-miR-888 | MI0005537 | UACUCAAAAAGCUGUCAGUCA | 0.2891 |
| hsa-miR-888* | MI0005537 | GACUGACACCUCUUUGGGUGAA | 1.4846 |
| hsa-miR-889 | MI0005540 | UUAAUAUCGGACAACCAUUGU | 0.3708 |
| hsa-miR-890 | MI0005533 | UACUUGGAAAGGCAUCAGUUG | 0.8842 |
| hsa-miR-891a | MI0005524 | UGCAACGAACCUGAGCCACUGA | 0.7112 |
| hsa-miR-891b | MI0005534 | UGCAACUUACCUGAGUCAUUGA | 0.2594 |
| hsa-miR-892a | MI0005528 | CACUGUGUCCUUUCUGCGUAG | 0.2299 |
| hsa-miR-892b | MI0005538 | CACUGGCUCCUUUCUGGGUAGA | 0.1974 |
| hsa-miR-9 | MI0000467 | UCUUUGGUUAUCUAGCUGUAUGA | 0.1513 |
| hsa-miR-9 | MI0000468 | UCUUUGGUUAUCUAGCUGUAUGA | 0.1668 |
| hsa-miR-9 | MI0000466 | UCUUUGGUUAUCUAGCUGUAUGA | 0.3244 |
| hsa-miR-9* | MI0000467 | AUAAAGCUAGAUAACCGAAAGU | 0.2472 |
| hsa-miR-9* | MI0000468 | AUAAAGCUAGAUAACCGAAAGU | 0.5728 |
| hsa-miR-9* | MI0000466 | AUAAAGCUAGAUAACCGAAAGU | 0.1582 |
| hsa-miR-92 | MI0000093 | UAUUGCACUUGUCCCGGCCUGU | 0.5086 |
| hsa-miR-92 | MI0000094 | UAUUGCACUUGUCCCGGCCUGU | 0.8224 |
| hsa-miR-920 | MI0005712 | GGGGAGCUGUGGAAGCAGUA | 1.152 |
| hsa-miR-921 | MI0005713 | CUAGUGAGGGACAGAACCAGGAUUC | 1.2732 |
| hsa-miR-922 | MI0005714 | GCAGCAGAGAAUAGGACUACGUC | 0.6656 |
| hsa-miR-923 | MI0005715 | GUCAGCGGAGGAAAAGAAACU | 1.8359 |
| hsa-miR-924 | MI0005716 | AGAGUCUUGUGAUGUCUUGC | 0.8629 |
| hsa-miR-92a-1* | MI0000093 | AGGUUGGGAUCGGUUGCAAUGCU | 0.3026 |
| hsa-miR-92a-2* | MI0000094 | GGGUGGGGAUUUGUUGCAUUAC | 0.4226 |
| hsa-miR-92b | MI0003560 | UAUUGCACUCGUCCCGGCCUCC | 0.8826 |
| hsa-miR-92b* | MI0003560 | AGGGACGGGACGCGGUGCAGUG | 0.6102 |
| hsa-miR-93 | MI0000095 | CAAAGUGCUGUUCGUGCAGGUAG | 0.9644 |
| hsa-miR-93* | MI0000095 | ACUGCUGAGCUAGCACUUCCCG | 0.671 |
| hsa-miR-933 | MI0005755 | UGUGCGCAGGGAGACCUCUCCC | 1.2413 |
| hsa-miR-934 | MI0005756 | UGUCUACUACUGGAGACACUGG | 1.2466 |
| hsa-miR-935 | MI0005757 | CCAGUUACCGCUUCCGCUACCGC | 2.2225 |
| hsa-miR-936 | MI0005758 | ACAGUAGAGGGAGGAAUCGCAG | 0.7755 |
| hsa-miR-937 | MI0005759 | AUCCGCGCUCUGACUCUCUGCC | 0.6455 |
| hsa-miR-938 | MI0005760 | UGCCCUUAAAGGUGAACCCAGU | 1.2339 |
| hsa-miR-939 | MI0005761 | UGGGGAGCUGAGGCUCUGGGGGUG | 0.8362 |
| hsa-miR-940 | MI0005762 | AAGGCAGGGCCCCCGCUCCCC | 1.3128 |
| hsa-miR-941 | MI0005763 | CACCCGGCUGUGUGCACAUGUGC | 0.9112 |
| hsa-miR-941 | MI0005764 | CACCCGGCUGUGUGCACAUGUGC | 0.9885 |
| hsa-miR-941 | MI0005765 | CACCCGGCUGUGUGCACAUGUGC | 1.037 |
| hsa-miR-941 | MI0005766 | CACCCGGCUGUGUGCACAUGUGC | 1.0989 |
| hsa-miR-942 | MI0005767 | UCUUCUCUGUUUUGGCCAUGUG | 1.3339 |
| hsa-miR-943 | MI0005768 | CUGACUGUUGCCGUCCUCCAG | 2.9533 |
| hsa-miR-944 | MI0005769 | AAAUUAUUGUACAUCGGAUGAG | 0.6726 |
| hsa-miR-95 | MI0000097 | UUCAACGGGUAUUUAUUGAGCA | 1.8489 |
| hsa-miR-96 | MI0000098 | UUUGGCACUAGCACAUUUUUGCU | 2.7004 |
| hsa-miR-96* | MI0000098 | AAUCAUGUGCAGUGCCAAUAUG | 0.8428 |
| hsa-miR-98 | MI0000100 | UGAGGUAGUAAGUUGUAUUGUU | 0.2142 |
| hsa-miR-99a | MI0000101 | AACCCGUAGAUCCGAUCUUGUG | 0.5767 |
| hsa-miR-99a* | MI0000101 | CAAGCUCGCUUCUAUGGGUCUG | 1.3548 |
| hsa-miR-99b | MI0000746 | CACCCGUAGAACCGACCUUGCG | 0.5141 |
| hsa-miR-99b* | MI0000746 | CAAGCUCGUGUCUGUGGGUCCG | 0.7024 |

| Supplementary Table S3. Cell proliferation and viability 6 days post miRNA transfection, relative to control miRNA, as determined by LNCaP MLuc cell viability assay. | | | | |
| --- | --- | --- | --- | --- |
|  |  |  |  |  |
|  |  |  |  |  |
| miroRNA | miRBase | Sequence | Mean | SEM |
| hsa-let-7a | MI0000061 | UGAGGUAGUAGGUUGUAUAGUU | 0.8152 | 0.0723 |
| hsa-let-7a | MI0000062 | UGAGGUAGUAGGUUGUAUAGUU | 0.9819 | 0.1634 |
| hsa-let-7a | MI0000060 | UGAGGUAGUAGGUUGUAUAGUU | 1.0963 | 0.2121 |
| hsa-let-7a* | MI0000062 | CUAUACAAUCUACUGUCUUUC | 0.7956 | 0.2056 |
| hsa-let-7a* | MI0000060 | CUAUACAAUCUACUGUCUUUC | 0.8348 | 0.2611 |
| hsa-let-7b | MI0000063 | UGAGGUAGUAGGUUGUGUGGUU | 0.7797 | 0.1433 |
| hsa-let-7b* | MI0000063 | CUAUACAACCUACUGCCUUCCC | 0.4228 | 0.0216 |
| hsa-let-7c | MI0000064 | UGAGGUAGUAGGUUGUAUGGUU | 0.819 | 0.0812 |
| hsa-let-7c* | MI0000064 | UAGAGUUACACCCUGGGAGUUA | 0.6112 | 0.2903 |
| hsa-let-7d | MI0000065 | AGAGGUAGUAGGUUGCAUAGUU | 0.3279 | 0.0717 |
| hsa-let-7d* | MI0000065 | CUAUACGACCUGCUGCCUUUCU | 0.8327 | 0.1955 |
| hsa-let-7e | MI0000066 | UGAGGUAGGAGGUUGUAUAGUU | 0.5115 | 0.1033 |
| hsa-let-7e* | MI0000066 | CUAUACGGCCUCCUAGCUUUCC | 0.6428 | 0.1337 |
| hsa-let-7f | MI0000068 | UGAGGUAGUAGAUUGUAUAGUU | 0.4352 | 0.1049 |
| hsa-let-7f | MI0000067 | UGAGGUAGUAGAUUGUAUAGUU | 0.6376 | 0.3845 |
| hsa-let-7f-1* | MI0000067 | CUAUACAAUCUAUUGCCUUCCC | 0.1722 | 0.0168 |
| hsa-let-7f-2* | MI0000068 | CUAUACAGUCUACUGUCUUUCC | 0.5005 | 0.0334 |
| hsa-let-7g | MI0000433 | UGAGGUAGUAGUUUGUACAGUU | 0.4762 | 0.0701 |
| hsa-let-7g* | MI0000433 | CUGUACAGGCCACUGCCUUGC | 0.1363 | 0.0098 |
| hsa-let-7i | MI0000434 | UGAGGUAGUAGUUUGUGCUGUU | 0.3621 | 0.0533 |
| hsa-let-7i* | MI0000434 | CUGCGCAAGCUACUGCCUUGCU | 0.7649 | 0.073 |
| hsa-miR-1 | MI0000437 | UGGAAUGUAAAGAAGUAUGUAU | 0.1937 | 0.0171 |
| hsa-miR-1 | MI0000651 | UGGAAUGUAAAGAAGUAUGUAU | 0.2062 | 0.0347 |
| hsa-miR-100 | MI0000102 | AACCCGUAGAUCCGAACUUGUG | 0.5132 | 0.0759 |
| hsa-miR-100* | MI0000102 | CAAGCUUGUAUCUAUAGGUAUG | 1.6167 | 0.3697 |
| hsa-miR-101 | MI0000739 | UACAGUACUGUGAUAACUGAA | 0.2485 | 0.0399 |
| hsa-miR-101 | MI0000103 | UACAGUACUGUGAUAACUGAA | 0.5491 | 0.1928 |
| hsa-miR-101* | MI0000103 | CAGUUAUCACAGUGCUGAUGCU | 0.101 | 0.0174 |
| hsa-miR-103 | MI0000108 | AGCAGCAUUGUACAGGGCUAUGA | 0.3449 | 0.0011 |
| hsa-miR-103 | MI0000109 | AGCAGCAUUGUACAGGGCUAUGA | 0.371 | 0.0358 |
| hsa-miR-105 | MI0000111 | UCAAAUGCUCAGACUCCUGUGGU | 0.4449 | 0.0278 |
| hsa-miR-105 | MI0000112 | UCAAAUGCUCAGACUCCUGUGGU | 0.5113 | 0.066 |
| hsa-miR-105* | MI0000111 | ACGGAUGUUUGAGCAUGUGCUA | 0.2006 | 0.0478 |
| hsa-miR-105* | MI0000112 | ACGGAUGUUUGAGCAUGUGCUA | 0.2058 | 0.0404 |
| hsa-miR-106a | MI0000113 | AAAAGUGCUUACAGUGCAGGUAG | 1.2391 | 0.1785 |
| hsa-miR-106a* | MI0000113 | CUGCAAUGUAAGCACUUCUUAC | 0.123 | 0.011 |
| hsa-miR-106b | MI0000734 | UAAAGUGCUGACAGUGCAGAU | 2.4041 | 0.6091 |
| hsa-miR-106b* | MI0000734 | CCGCACUGUGGGUACUUGCUGC | 0.4378 | 0.0774 |
| hsa-miR-107 | MI0000114 | AGCAGCAUUGUACAGGGCUAUCA | 0.4703 | 0.0346 |
| hsa-miR-10a | MI0000266 | UACCCUGUAGAUCCGAAUUUGUG | 0.2237 | 0.0306 |
| hsa-miR-10a* | MI0000266 | CAAAUUCGUAUCUAGGGGAAUA | 0.4239 | 0.0024 |
| hsa-miR-10b | MI0000267 | UACCCUGUAGAACCGAAUUUGUG | 0.277 | 0.1469 |
| hsa-miR-10b* | MI0000267 | ACAGAUUCGAUUCUAGGGGAAU | 0.3621 | 0.0098 |
| hsa-miR-122* | MI0000442 | AACGCCAUUAUCACACUAAAUA | 0.394 | 0.118 |
| hsa-miR-1224-3p | MI0003764 | CCCCACCUCCUCUCUCCUCAG | 2.0573 | 0.3099 |
| hsa-miR-1224-5p | MI0003764 | GUGAGGACUCGGGAGGUGG | 0.6318 | 0.1033 |
| hsa-miR-1225-3p | MI0006311 | UGAGCCCCUGUGCCGCCCCCAG | 0.9562 | 0.0504 |
| hsa-miR-1225-5p | MI0006311 | GUGGGUACGGCCCAGUGGGGGG | 0.976 | 0.0202 |
| hsa-miR-1226 | MI0006313 | UCACCAGCCCUGUGUUCCCUAG | 0.2444 | 0.0405 |
| hsa-miR-1226* | MI0006313 | GUGAGGGCAUGCAGGCCUGGAUGGGG | 0.4549 | 0.0537 |
| hsa-miR-1227 | MI0006316 | CGUGCCACCCUUUUCCCCAG | 0.7512 | 0.0125 |
| hsa-miR-1228 | MI0006318 | UCACACCUGCCUCGCCCCCC | 0.292 | 0.0243 |
| hsa-miR-1228* | MI0006318 | GUGGGCGGGGGCAGGUGUGUG | 0.7775 | 0.049 |
| hsa-miR-1229 | MI0006319 | CUCUCACCACUGCCCUCCCACAG | 1.0098 | 0.583 |
| hsa-miR-122a | MI0000442 | UGGAGUGUGACAAUGGUGUUUG | 0.1617 | 0.0287 |
| hsa-miR-1231 | MI0006321 | GUGUCUGGGCGGACAGCUGC | 0.1323 | 0.055 |
| hsa-miR-1233 | MI0006323 | UGAGCCCUGUCCUCCCGCAG | 0.3528 | 0.1916 |
| hsa-miR-1234 | MI0006324 | UCGGCCUGACCACCCACCCCAC | 0.5824 | 0.2372 |
| hsa-miR-1236 | MI0006326 | CCUCUUCCCCUUGUCUCUCCAG | 0.8391 | 0.1377 |
| hsa-miR-1237 | MI0006327 | UCCUUCUGCUCCGUCCCCCAG | 0.2133 | 0.0479 |
| hsa-miR-1238 | MI0006328 | CUUCCUCGUCUGUCUGCCCC | 0.2088 | 0.0077 |
| hsa-miR-124* | MI0000443 | CGUGUUCACAGCGGACCUUGAU | 0.1931 | 0.0197 |
| hsa-miR-124* | MI0000445 | CGUGUUCACAGCGGACCUUGAU | 0.2521 | 0.0645 |
| hsa-miR-124* | MI0000444 | CGUGUUCACAGCGGACCUUGAU | 0.3108 | 0.0184 |
| hsa-miR-124a | MI0000443 | UAAGGCACGCGGUGAAUGCC | 0.103 | 0.0162 |
| hsa-miR-124a | MI0000444 | UAAGGCACGCGGUGAAUGCC | 0.104 | 0.0085 |
| hsa-miR-124a | MI0000445 | UAAGGCACGCGGUGAAUGCC | 0.1123 | 0.0031 |
| hsa-miR-125a | MI0000469 | UCCCUGAGACCCUUUAACCUGUGA | 1.004 | 0.1314 |
| hsa-miR-125a-3p | MI0000469 | ACAGGUGAGGUUCUUGGGAGCC | 0.5154 | 0.4322 |
| hsa-miR-125b | MI0000446 | UCCCUGAGACCCUAACUUGUGA | 0.5191 | 0.0712 |
| hsa-miR-125b | MI0000470 | UCCCUGAGACCCUAACUUGUGA | 0.667 | 0.1378 |
| hsa-miR-125b-1* | MI0000446 | ACGGGUUAGGCUCUUGGGAGCU | 0.4023 | 0.0393 |
| hsa-miR-125b-2* | MI0000470 | UCACAAGUCAGGCUCUUGGGAC | 2.0544 | 0.0991 |
| hsa-miR-126 | MI0000471 | UCGUACCGUGAGUAAUAAUGCG | 0.7136 | 0.3024 |
| hsa-miR-126* | MI0000471 | CAUUAUUACUUUUGGUACGCG | 0.4906 | 0.0614 |
| hsa-miR-127 | MI0000472 | UCGGAUCCGUCUGAGCUUGGCU | 0.3257 | 0.0275 |
| hsa-miR-127-5p | MI0000472 | CUGAAGCUCAGAGGGCUCUGAU | 0.2503 | 0.034 |
| hsa-miR-128 | MI0000727 | UCACAGUGAACCGGUCUCUUU | 0.8333 | 0.1206 |
| hsa-miR-128a | MI0000447 | UCACAGUGAACCGGUCUCUUU | 0.5645 | 0.042 |
| hsa-miR-129 | MI0000473 | CUUUUUGCGGUCUGGGCUUGC | 0.2003 | 0.0935 |
| hsa-miR-129 | MI0000252 | CUUUUUGCGGUCUGGGCUUGC | 0.2283 | 0.0833 |
| hsa-miR-129* | MI0000252 | AAGCCCUUACCCCAAAAAGUAU | 0.1462 | 0.0054 |
| hsa-miR-129-3p | MI0000473 | AAGCCCUUACCCCAAAAAGCAU | 0.1695 | 0.0271 |
| hsa-miR-130a | MI0000448 | CAGUGCAAUGUUAAAAGGGCAU | 1.0433 | 0.2531 |
| hsa-miR-130a* | MI0000448 | UUCACAUUGUGCUACUGUCUGC | 0.4121 | 0.0147 |
| hsa-miR-130b | MI0000748 | CAGUGCAAUGAUGAAAGGGCAU | 0.8339 | 0.2289 |
| hsa-miR-130b* | MI0000748 | ACUCUUUCCCUGUUGCACUAC | 0.1555 | 0.0191 |
| hsa-miR-132 | MI0000449 | UAACAGUCUACAGCCAUGGUCG | 0.7556 | 0.1162 |
| hsa-miR-132* | MI0000449 | ACCGUGGCUUUCGAUUGUUACU | 0.2955 | 0.1702 |
| hsa-miR-133a | MI0000450 | UUUGGUCCCCUUCAACCAGCUG | 0.2066 | 0.0686 |
| hsa-miR-133a | MI0000451 | UUUGGUCCCCUUCAACCAGCUG | 0.2073 | 0.0676 |
| hsa-miR-133b | MI0000822 | UUUGGUCCCCUUCAACCAGCUA | 0.1641 | 0.0207 |
| hsa-miR-134 | MI0000474 | UGUGACUGGUUGACCAGAGGGG | 0.2384 | 0.0684 |
| hsa-miR-135a | MI0000452 | UAUGGCUUUUUAUUCCUAUGUGA | 0.1319 | 0.0205 |
| hsa-miR-135a | MI0000453 | UAUGGCUUUUUAUUCCUAUGUGA | 0.1642 | 0.0516 |
| hsa-miR-135a* | MI0000452 | UAUAGGGAUUGGAGCCGUGGCG | 0.2558 | 0.0549 |
| hsa-miR-135b | MI0000810 | UAUGGCUUUUCAUUCCUAUGUGA | 0.0901 | 0.0023 |
| hsa-miR-135b* | MI0000810 | AUGUAGGGCUAAAAGCCAUGGG | 0.1455 | 0.0037 |
| hsa-miR-136 | MI0000475 | ACUCCAUUUGUUUUGAUGAUGGA | 0.4971 | 0.2645 |
| hsa-miR-136* | MI0000475 | CAUCAUCGUCUCAAAUGAGUCU | 0.117 | 0.0173 |
| hsa-miR-137 | MI0000454 | UUAUUGCUUAAGAAUACGCGUAG | 0.2419 | 0.0842 |
| hsa-miR-138 | MI0000455 | AGCUGGUGUUGUGAAUCAGGCCG | 0.3205 | 0.0368 |
| hsa-miR-138 | MI0000476 | AGCUGGUGUUGUGAAUCAGGCCG | 0.3646 | 0.0256 |
| hsa-miR-138-1* | MI0000476 | GCUACUUCACAACACCAGGGCC | 1.3971 | 0.1712 |
| hsa-miR-138-2* | MI0000455 | GCUAUUUCACGACACCAGGGUU | 0.7195 | 0.1506 |
| hsa-miR-139 | MI0000261 | UCUACAGUGCACGUGUCUCCAG | 0.8646 | 0.1525 |
| hsa-miR-139-3p | MI0000261 | GGAGACGCGGCCCUGUUGGAGU | 0.4568 | 0.0776 |
| hsa-miR-140 | MI0000456 | CAGUGGUUUUACCCUAUGGUAG | 0.5884 | 0.0707 |
| hsa-miR-140-3p | MI0000456 | UACCACAGGGUAGAACCACGG | 0.9923 | 0.107 |
| hsa-miR-141 | MI0000457 | UAACACUGUCUGGUAAAGAUGG | 0.4039 | 0.0778 |
| hsa-miR-141* | MI0000457 | CAUCUUCCAGUACAGUGUUGGA | 0.2503 | 0.0223 |
| hsa-miR-142-3p | MI0000458 | UGUAGUGUUUCCUACUUUAUGGA | 0.6694 | 0.1127 |
| hsa-miR-142-5p | MI0000458 | CAUAAAGUAGAAAGCACUACU | 1.4147 | 0.2088 |
| hsa-miR-143 | MI0000459 | UGAGAUGAAGCACUGUAGCUC | 0.2236 | 0.0322 |
| hsa-miR-143* | MI0000459 | GGUGCAGUGCUGCAUCUCUGGU | 1.105 | 0.1227 |
| hsa-miR-144 | MI0000460 | UACAGUAUAGAUGAUGUACU | 0.4987 | 0.0506 |
| hsa-miR-144* | MI0000460 | GGAUAUCAUCAUAUACUGUAAG | 0.1903 | 0.0107 |
| hsa-miR-145 | MI0000461 | GUCCAGUUUUCCCAGGAAUCCCU | 0.3059 | 0.0065 |
| hsa-miR-145* | MI0000461 | GGAUUCCUGGAAAUACUGUUCU | 1.0312 | 0.2506 |
| hsa-miR-146a | MI0000477 | UGAGAACUGAAUUCCAUGGGUU | 0.3316 | 0.0724 |
| hsa-miR-146a* | MI0000477 | CCUCUGAAAUUCAGUUCUUCAG | 1.3323 | 0.3541 |
| hsa-miR-146b | MI0003129 | UGAGAACUGAAUUCCAUAGGCU | 0.327 | 0.0019 |
| hsa-miR-146b-3p | MI0003129 | UGCCCUGUGGACUCAGUUCUGG | 0.6786 | 0.1043 |
| hsa-miR-147 | MI0000262 | GUGUGUGGAAAUGCUUCUGC | 0.6073 | 0.2427 |
| hsa-miR-147b | MI0005544 | GUGUGCGGAAAUGCUUCUGCUA | 0.6178 | 0.0306 |
| hsa-miR-148a | MI0000253 | UCAGUGCACUACAGAACUUUGU | 0.9098 | 0.2372 |
| hsa-miR-148a* | MI0000253 | AAAGUUCUGAGACACUCCGACU | 0.7261 | 0.174 |
| hsa-miR-148b | MI0000811 | UCAGUGCAUCACAGAACUUUGU | 0.6849 | 0.0633 |
| hsa-miR-148b* | MI0000811 | AAGUUCUGUUAUACACUCAGGC | 0.1291 | 0.0115 |
| hsa-miR-149 | MI0000478 | UCUGGCUCCGUGUCUUCACUCCC | 0.6359 | 0.2522 |
| hsa-miR-149* | MI0000478 | AGGGAGGGACGGGGGCUGUGC | 0.1963 | 0.0388 |
| hsa-miR-150 | MI0000479 | UCUCCCAACCCUUGUACCAGUG | 0.6432 | 0.2115 |
| hsa-miR-150* | MI0000479 | CUGGUACAGGCCUGGGGGACAG | 0.9967 | 0.1042 |
| hsa-miR-151 | MI0000809 | CUAGACUGAAGCUCCUUGAGG | 0.3485 | 0.0539 |
| hsa-miR-151-5p | MI0000809 | UCGAGGAGCUCACAGUCUAGU | 0.2451 | 0.0046 |
| hsa-miR-152 | MI0000462 | UCAGUGCAUGACAGAACUUGG | 0.6613 | 0.086 |
| hsa-miR-153 | MI0000463 | UUGCAUAGUCACAAAAGUGAUC | 0.1019 | 0.0018 |
| hsa-miR-153 | MI0000464 | UUGCAUAGUCACAAAAGUGAUC | 0.1215 | 0.0294 |
| hsa-miR-154 | MI0000480 | UAGGUUAUCCGUGUUGCCUUCG | 0.2367 | 0.0497 |
| hsa-miR-154* | MI0000480 | AAUCAUACACGGUUGACCUAUU | 0.45 | 0.0812 |
| hsa-miR-155 | MI0000681 | UUAAUGCUAAUCGUGAUAGGGGU | 0.5478 | 0.0607 |
| hsa-miR-155* | MI0000681 | CUCCUACAUAUUAGCAUUAACA | 0.9802 | 0.0315 |
| hsa-miR-15a | MI0000069 | UAGCAGCACAUAAUGGUUUGUG | 0.184 | 0.0823 |
| hsa-miR-15a* | MI0000069 | CAGGCCAUAUUGUGCUGCCUCA | 0.3795 | 0.0934 |
| hsa-miR-15b | MI0000438 | UAGCAGCACAUCAUGGUUUACA | 0.1999 | 0.0532 |
| hsa-miR-15b* | MI0000438 | CGAAUCAUUAUUUGCUGCUCUA | 1.1132 | 0.0587 |
| hsa-miR-16 | MI0000070 | UAGCAGCACGUAAAUAUUGGCG | 0.1876 | 0.0995 |
| hsa-miR-16 | MI0000115 | UAGCAGCACGUAAAUAUUGGCG | 0.3088 | 0.1467 |
| hsa-miR-16-1* | MI0000070 | CCAGUAUUAACUGUGCUGCUGA | 0.9442 | 0.1372 |
| hsa-miR-16-2* | MI0000115 | CCAAUAUUACUGUGCUGCUUUA | 0.4332 | 0.0366 |
| hsa-miR-17-3p | MI0000071 | ACUGCAGUGAAGGCACUUGUAG | 0.3981 | 0.1112 |
| hsa-miR-17-5p | MI0000071 | CAAAGUGCUUACAGUGCAGGUAG | 0.9347 | 0.3069 |
| hsa-miR-181a | MI0000269 | AACAUUCAACGCUGUCGGUGAGU | 0.6436 | 0.0163 |
| hsa-miR-181a | MI0000289 | AACAUUCAACGCUGUCGGUGAGU | 0.7769 | 0.1722 |
| hsa-miR-181a-2* | MI0000269 | ACCACUGACCGUUGACUGUACC | 0.9399 | 0.073 |
| hsa-miR-181b | MI0000270 | AACAUUCAUUGCUGUCGGUGGGU | 0.6857 | 0.123 |
| hsa-miR-181b | MI0000683 | AACAUUCAUUGCUGUCGGUGGGU | 0.8013 | 0.0404 |
| hsa-miR-181c | MI0000271 | AACAUUCAACCUGUCGGUGAGU | 0.5523 | 0.0258 |
| hsa-miR-181c* | MI0000271 | AACCAUCGACCGUUGAGUGGAC | 0.2652 | 0.0518 |
| hsa-miR-181d | MI0003139 | AACAUUCAUUGUUGUCGGUGGGU | 1.3085 | 0.3547 |
| hsa-miR-182 | MI0000272 | UUUGGCAAUGGUAGAACUCACACU | 0.5013 | 0.17 |
| hsa-miR-182* | MI0000272 | UGGUUCUAGACUUGCCAACUA | 0.1435 | 0.0194 |
| hsa-miR-183 | MI0000273 | UAUGGCACUGGUAGAAUUCACU | 0.1098 | 0.0146 |
| hsa-miR-183* | MI0000273 | GUGAAUUACCGAAGGGCCAUAA | 0.3526 | 0.0354 |
| hsa-miR-184 | MI0000481 | UGGACGGAGAACUGAUAAGGGU | 0.1365 | 0.0325 |
| hsa-miR-185 | MI0000482 | UGGAGAGAAAGGCAGUUCCUGA | 0.1035 | 0.0284 |
| hsa-miR-185* | MI0000482 | AGGGGCUGGCUUUCCUCUGGUC | 0.1151 | 0.0122 |
| hsa-miR-186 | MI0000483 | CAAAGAAUUCUCCUUUUGGGCU | 0.4177 | 0.102 |
| hsa-miR-186* | MI0000483 | GCCCAAAGGUGAAUUUUUUGGG | 1.2398 | 0.2357 |
| hsa-miR-187 | MI0000274 | UCGUGUCUUGUGUUGCAGCCGG | 0.6976 | 0.0151 |
| hsa-miR-187* | MI0000274 | GGCUACAACACAGGACCCGGGC | 1.7665 | 0.0887 |
| hsa-miR-188 | MI0000484 | CAUCCCUUGCAUGGUGGAGGG | 0.4852 | 0.0852 |
| hsa-miR-188-3p | MI0000484 | CUCCCACAUGCAGGGUUUGCA | 0.8694 | 0.0805 |
| hsa-miR-189 | MI0000080 | UGCCUACUGAGCUGAUAUCAGU | 0.4792 | 0.2065 |
| hsa-miR-18a | MI0000072 | UAAGGUGCAUCUAGUGCAGAUAG | 0.6399 | 0.0682 |
| hsa-miR-18a* | MI0000072 | ACUGCCCUAAGUGCUCCUUCUGG | 0.6925 | 0.0112 |
| hsa-miR-18b | MI0001518 | UAAGGUGCAUCUAGUGCAGUUAG | 0.4067 | 0.0481 |
| hsa-miR-18b* | MI0001518 | UGCCCUAAAUGCCCCUUCUGGC | 0.3996 | 0.0279 |
| hsa-miR-190 | MI0000486 | UGAUAUGUUUGAUAUAUUAGGU | 0.2586 | 0.0561 |
| hsa-miR-190b | MI0005545 | UGAUAUGUUUGAUAUUGGGUU | 0.3515 | 0.0992 |
| hsa-miR-191 | MI0000465 | CAACGGAAUCCCAAAAGCAGCUG | 0.4863 | 0.034 |
| hsa-miR-191* | MI0000465 | GCUGCGCUUGGAUUUCGUCCCC | 2.1111 | 0.0758 |
| hsa-miR-192 | MI0000234 | CUGACCUAUGAAUUGACAGCC | 0.4265 | 0.0384 |
| hsa-miR-192* | MI0000234 | CUGCCAAUUCCAUAGGUCACAG | 0.4259 | 0.1623 |
| hsa-miR-193a | MI0000487 | AACUGGCCUACAAAGUCCCAGU | 0.0831 | 0.0087 |
| hsa-miR-193a-5p | MI0000487 | UGGGUCUUUGCGGGCGAGAUGA | 0.2692 | 0.0342 |
| hsa-miR-193b | MI0003137 | AACUGGCCCUCAAAGUCCCGCU | 0.2348 | 0.0624 |
| hsa-miR-193b* | MI0003137 | CGGGGUUUUGAGGGCGAGAUGA | 0.1579 | 0.0006 |
| hsa-miR-194 | MI0000732 | UGUAACAGCAACUCCAUGUGGA | 0.3329 | 0.017 |
| hsa-miR-194 | MI0000488 | UGUAACAGCAACUCCAUGUGGA | 0.4857 | 0.1167 |
| hsa-miR-194* | MI0000732 | CCAGUGGGGCUGCUGUUAUCUG | 0.3506 | 0.0858 |
| hsa-miR-195 | MI0000489 | UAGCAGCACAGAAAUAUUGGC | 0.1613 | 0.0287 |
| hsa-miR-195* | MI0000489 | CCAAUAUUGGCUGUGCUGCUCC | 0.9212 | 0.053 |
| hsa-miR-196a | MI0000238 | UAGGUAGUUUCAUGUUGUUGGG | 0.1471 | 0.0166 |
| hsa-miR-196a | MI0000279 | UAGGUAGUUUCAUGUUGUUGGG | 0.221 | 0.0383 |
| hsa-miR-196a* | MI0000279 | CGGCAACAAGAAACUGCCUGAG | 0.4361 | 0.1962 |
| hsa-miR-196b | MI0001150 | UAGGUAGUUUCCUGUUGUUGGG | 0.1616 | 0.0379 |
| hsa-miR-197 | MI0000239 | UUCACCACCUUCUCCACCCAGC | 0.2054 | 0.095 |
| hsa-miR-198 | MI0000240 | GGUCCAGAGGGGAGAUAGGUUC | 0.5281 | 0.1271 |
| hsa-miR-199a | MI0000242 | CCCAGUGUUCAGACUACCUGUUC | 0.3201 | 0.0816 |
| hsa-miR-199a | MI0000281 | CCCAGUGUUCAGACUACCUGUUC | 0.3661 | 0.0655 |
| hsa-miR-199a* | MI0000242 | ACAGUAGUCUGCACAUUGGUUA | 0.1358 | 0.0233 |
| hsa-miR-199a* | MI0000281 | ACAGUAGUCUGCACAUUGGUUA | 0.1756 | 0.0055 |
| hsa-miR-199b | MI0000282 | CCCAGUGUUUAGACUAUCUGUUC | 0.5623 | 0.0299 |
| hsa-miR-199b-3p | MI0000282 | ACAGUAGUCUGCACAUUGGUUA | 0.201 | 0.0189 |
| hsa-miR-19a | MI0000073 | UGUGCAAAUCUAUGCAAAACUGA | 2.0011 | 0.9839 |
| hsa-miR-19a* | MI0000073 | AGUUUUGCAUAGUUGCACUACA | 0.3032 | 0.146 |
| hsa-miR-19b | MI0000074 | UGUGCAAAUCCAUGCAAAACUGA | 0.7522 | 0.0416 |
| hsa-miR-19b | MI0000075 | UGUGCAAAUCCAUGCAAAACUGA | 1.7644 | 0.8805 |
| hsa-miR-19b-1* | MI0000074 | AGUUUUGCAGGUUUGCAUCCAGC | 0.1425 | 0.0689 |
| hsa-miR-19b-2* | MI0000075 | AGUUUUGCAGGUUUGCAUUUCA | 0.2658 | 0.0027 |
| hsa-miR-200a | MI0000737 | UAACACUGUCUGGUAACGAUGU | 0.5927 | 0.1247 |
| hsa-miR-200a* | MI0000737 | CAUCUUACCGGACAGUGCUGGA | 0.381 | 0.0898 |
| hsa-miR-200b | MI0000342 | UAAUACUGCCUGGUAAUGAUGA | 0.473 | 0.2436 |
| hsa-miR-200b* | MI0000342 | CAUCUUACUGGGCAGCAUUGGA | 1.074 | 0.065 |
| hsa-miR-200c | MI0000650 | UAAUACUGCCGGGUAAUGAUGGA | 0.4909 | 0.0337 |
| hsa-miR-200c* | MI0000650 | CGUCUUACCCAGCAGUGUUUGG | 0.3317 | 0.3224 |
| hsa-miR-202 | MI0003130 | AGAGGUAUAGGGCAUGGGAA | 0.5425 | 0.2501 |
| hsa-miR-202* | MI0003130 | UUCCUAUGCAUAUACUUCUUUG | 1.1625 | 0.6686 |
| hsa-miR-203 | MI0000283 | GUGAAAUGUUUAGGACCACUAG | 0.4363 | 0.0308 |
| hsa-miR-204 | MI0000284 | UUCCCUUUGUCAUCCUAUGCCU | 0.3748 | 0.0365 |
| hsa-miR-205 | MI0000285 | UCCUUCAUUCCACCGGAGUCUG | 1.2005 | 0.0486 |
| hsa-miR-206 | MI0000490 | UGGAAUGUAAGGAAGUGUGUGG | 0.3407 | 0.0011 |
| hsa-miR-208 | MI0000251 | AUAAGACGAGCAAAAAGCUUGU | 0.2178 | 0.0083 |
| hsa-miR-208b | MI0005570 | AUAAGACGAACAAAAGGUUUGU | 0.0815 | 0.0205 |
| hsa-miR-20a | MI0000076 | UAAAGUGCUUAUAGUGCAGGUAG | 2.4843 | 1.1369 |
| hsa-miR-20a* | MI0000076 | ACUGCAUUAUGAGCACUUAAAG | 0.7781 | 0.3161 |
| hsa-miR-20b | MI0001519 | CAAAGUGCUCAUAGUGCAGGUAG | 2.4243 | 0.465 |
| hsa-miR-20b* | MI0001519 | ACUGUAGUAUGGGCACUUCCAG | 0.2433 | 0.0175 |
| hsa-miR-21 | MI0000077 | UAGCUUAUCAGACUGAUGUUGA | 2.7443 | 0.7704 |
| hsa-miR-21* | MI0000077 | CAACACCAGUCGAUGGGCUGU | 0.2565 | 0.0373 |
| hsa-miR-210 | MI0000286 | CUGUGCGUGUGACAGCGGCUGA | 0.5606 | 0.0071 |
| hsa-miR-211 | MI0000287 | UUCCCUUUGUCAUCCUUCGCCU | 0.4704 | 0.0326 |
| hsa-miR-212 | MI0000288 | UAACAGUCUCCAGUCACGGCC | 0.519 | 0.2204 |
| hsa-miR-213 | MI0000289 | ACCAUCGACCGUUGAUUGUACC | 1.2142 | 0.2456 |
| hsa-miR-214 | MI0000290 | ACAGCAGGCACAGACAGGCAGU | 0.4922 | 0.2861 |
| hsa-miR-214* | MI0000290 | UGCCUGUCUACACUUGCUGUGC | 0.091 | 0.0165 |
| hsa-miR-215 | MI0000291 | AUGACCUAUGAAUUGACAGAC | 0.4358 | 0.1684 |
| hsa-miR-216 | MI0000292 | UAAUCUCAGCUGGCAACUGUGA | 0.5158 | 0.1159 |
| hsa-miR-216b | MI0005569 | AAAUCUCUGCAGGCAAAUGUGA | 0.4992 | 0.016 |
| hsa-miR-217 | MI0000293 | UACUGCAUCAGGAACUGAUUGGA | 0.1541 | 0.0572 |
| hsa-miR-218 | MI0000295 | UUGUGCUUGAUCUAACCAUGU | 0.37 | 0.0401 |
| hsa-miR-218 | MI0000294 | UUGUGCUUGAUCUAACCAUGU | 0.5356 | 0.0758 |
| hsa-miR-218-1* | MI0000294 | AUGGUUCCGUCAAGCACCAUGG | 0.3057 | 0.0613 |
| hsa-miR-218-2* | MI0000295 | CAUGGUUCUGUCAAGCACCGCG | 0.2941 | 0.0034 |
| hsa-miR-219 | MI0000740 | UGAUUGUCCAAACGCAAUUCU | 0.3092 | 0.0693 |
| hsa-miR-219 | MI0000296 | UGAUUGUCCAAACGCAAUUCU | 0.3565 | 0.068 |
| hsa-miR-219-1-3p | MI0000296 | AGAGUUGAGUCUGGACGUCCCG | 0.1082 | 0.0032 |
| hsa-miR-219-2-3p | MI0000740 | AGAAUUGUGGCUGGACAUCUGU | 0.3628 | 0.0247 |
| hsa-miR-22 | MI0000078 | AAGCUGCCAGUUGAAGAACUGU | 0.306 | 0.1525 |
| hsa-miR-22* | MI0000078 | AGUUCUUCAGUGGCAAGCUUUA | 0.248 | 0.1197 |
| hsa-miR-220 | MI0000297 | CCACACCGUAUCUGACACUUU | 1.8504 | 0.5268 |
| hsa-miR-220b | MI0005529 | CCACCACCGUGUCUGACACUU | 1.3159 | 0.1034 |
| hsa-miR-220c | MI0005536 | ACACAGGGCUGUUGUGAAGACU | 0.1492 | 0.0237 |
| hsa-miR-221 | MI0000298 | AGCUACAUUGUCUGCUGGGUUUC | 1.329 | 0.367 |
| hsa-miR-221* | MI0000298 | ACCUGGCAUACAAUGUAGAUUU | 0.2238 | 0.0026 |
| hsa-miR-222 | MI0000299 | AGCUACAUCUGGCUACUGGGU | 0.9132 | 0.0089 |
| hsa-miR-222* | MI0000299 | CUCAGUAGCCAGUGUAGAUCCU | 0.1809 | 0.0005 |
| hsa-miR-223 | MI0000300 | UGUCAGUUUGUCAAAUACCCCA | 0.6998 | 0.0785 |
| hsa-miR-223* | MI0000300 | CGUGUAUUUGACAAGCUGAGUU | 0.1915 | 0.0012 |
| hsa-miR-224 | MI0000301 | CAAGUCACUAGUGGUUCCGUU | 0.2164 | 0.065 |
| hsa-miR-23a | MI0000079 | AUCACAUUGCCAGGGAUUUCC | 1.993 | 0.7532 |
| hsa-miR-23a* | MI0000079 | GGGGUUCCUGGGGAUGGGAUUU | 0.8609 | 0.4194 |
| hsa-miR-23b | MI0000439 | AUCACAUUGCCAGGGAUUACC | 0.5648 | 0.1943 |
| hsa-miR-23b* | MI0000439 | UGGGUUCCUGGCAUGCUGAUUU | 0.1566 | 0.0163 |
| hsa-miR-24 | MI0000081 | UGGCUCAGUUCAGCAGGAACAG | 0.3173 | 0.0763 |
| hsa-miR-24 | MI0000080 | UGGCUCAGUUCAGCAGGAACAG | 0.4899 | 0.4747 |
| hsa-miR-24-2* | MI0000081 | UGCCUACUGAGCUGAAACACAG | 0.4738 | 0.089 |
| hsa-miR-25 | MI0000082 | CAUUGCACUUGUCUCGGUCUGA | 0.8621 | 0.1944 |
| hsa-miR-25* | MI0000082 | AGGCGGAGACUUGGGCAAUUG | 0.0685 | 0.0078 |
| hsa-miR-26a | MI0000083 | UUCAAGUAAUCCAGGAUAGGCU | 0.0859 | 0.016 |
| hsa-miR-26a | MI0000750 | UUCAAGUAAUCCAGGAUAGGCU | 0.1596 | 0.025 |
| hsa-miR-26a-1* | MI0000083 | CCUAUUCUUGGUUACUUGCACG | 1.8728 | 0.5805 |
| hsa-miR-26a-2* | MI0000750 | CCUAUUCUUGAUUACUUGUUUC | 1.3553 | 0.2738 |
| hsa-miR-26b | MI0000084 | UUCAAGUAAUUCAGGAUAGGU | 0.254 | 0.0301 |
| hsa-miR-26b* | MI0000084 | CCUGUUCUCCAUUACUUGGCUC | 0.4898 | 0.0627 |
| hsa-miR-27a | MI0000085 | UUCACAGUGGCUAAGUUCCGC | 0.53 | 0.155 |
| hsa-miR-27a* | MI0000085 | AGGGCUUAGCUGCUUGUGAGCA | 0.1884 | 0.0451 |
| hsa-miR-27b | MI0000440 | UUCACAGUGGCUAAGUUCUGC | 0.4505 | 0.108 |
| hsa-miR-27b* | MI0000440 | AGAGCUUAGCUGAUUGGUGAAC | 0.9146 | 0.0843 |
| hsa-miR-28 | MI0000086 | AAGGAGCUCACAGUCUAUUGAG | 0.227 | 0.04 |
| hsa-miR-28-3p | MI0000086 | CACUAGAUUGUGAGCUCCUGGA | 0.2578 | 0.0497 |
| hsa-miR-296 | MI0000747 | AGGGCCCCCCCUCAAUCCUGU | 0.2495 | 0.0126 |
| hsa-miR-296-3p | MI0000747 | GAGGGUUGGGUGGAGGCUCUCC | 0.4951 | 0.0307 |
| hsa-miR-297 | MI0005775 | AUGUAUGUGUGCAUGUGCAUG | 0.1794 | 0.0017 |
| hsa-miR-298 | MI0005523 | AGCAGAAGCAGGGAGGUUCUCCCA | 0.7182 | 0.1496 |
| hsa-miR-299-3p | MI0000744 | UAUGUGGGAUGGUAAACCGCUU | 0.1448 | 0.034 |
| hsa-miR-299-5p | MI0000744 | UGGUUUACCGUCCCACAUACAU | 0.1394 | 0.0254 |
| hsa-miR-29a | MI0000087 | UAGCACCAUCUGAAAUCGGUUA | 0.1693 | 0.0044 |
| hsa-miR-29a* | MI0000087 | ACUGAUUUCUUUUGGUGUUCAG | 0.7536 | 0.0236 |
| hsa-miR-29b | MI0000107 | UAGCACCAUUUGAAAUCAGUGUU | 1.2051 | 0.2478 |
| hsa-miR-29b | MI0000105 | UAGCACCAUUUGAAAUCAGUGUU | 1.3038 | 0.1081 |
| hsa-miR-29b-1* | MI0000105 | GCUGGUUUCAUAUGGUGGUUUAGA | 0.3049 | 0.0703 |
| hsa-miR-29b-2* | MI0000107 | CUGGUUUCACAUGGUGGCUUAG | 0.33 | 0.0479 |
| hsa-miR-29c | MI0000735 | UAGCACCAUUUGAAAUCGGUUA | 0.1432 | 0.0192 |
| hsa-miR-29c* | MI0000735 | UGACCGAUUUCUCCUGGUGUUC | 0.323 | 0.0051 |
| hsa-miR-300 | MI0005525 | UAUACAAGGGCAGACUCUCUCU | 0.7927 | 0.0522 |
| hsa-miR-301 | MI0000745 | CAGUGCAAUAGUAUUGUCAAAGC | 0.4396 | 0.0204 |
| hsa-miR-301b | MI0005568 | CAGUGCAAUGAUAUUGUCAAAGC | 1.007 | 0.0266 |
| hsa-miR-302a | MI0000738 | UAAGUGCUUCCAUGUUUUGGUGA | 1.1323 | 0.6167 |
| hsa-miR-302a* | MI0000738 | ACUUAAACGUGGAUGUACUUGCU | 0.919 | 0.1388 |
| hsa-miR-302b | MI0000772 | UAAGUGCUUCCAUGUUUUAGUAG | 0.8329 | 0.0144 |
| hsa-miR-302b* | MI0000772 | ACUUUAACAUGGAAGUGCUUUC | 1.3759 | 0.3204 |
| hsa-miR-302c | MI0000773 | UAAGUGCUUCCAUGUUUCAGUGG | 1.1707 | 0.5781 |
| hsa-miR-302c* | MI0000773 | UUUAACAUGGGGGUACCUGCUG | 0.8209 | 0.2473 |
| hsa-miR-302d | MI0000774 | UAAGUGCUUCCAUGUUUGAGUGU | 0.7703 | 0.1707 |
| hsa-miR-302d* | MI0000774 | ACUUUAACAUGGAGGCACUUGC | 0.6651 | 0.0441 |
| hsa-miR-30a-3p | MI0000088 | CUUUCAGUCGGAUGUUUGCAGC | 0.5094 | 0.0516 |
| hsa-miR-30a-5p | MI0000088 | UGUAAACAUCCUCGACUGGAAG | 0.2187 | 0.0766 |
| hsa-miR-30b | MI0000441 | UGUAAACAUCCUACACUCAGCU | 0.249 | 0.0218 |
| hsa-miR-30b* | MI0000441 | CUGGGAGGUGGAUGUUUACUUC | 0.0778 | 0.0074 |
| hsa-miR-30c | MI0000254 | UGUAAACAUCCUACACUCUCAGC | 0.2719 | 0.0139 |
| hsa-miR-30c | MI0000736 | UGUAAACAUCCUACACUCUCAGC | 1.9641 | 0.0081 |
| hsa-miR-30c-1* | MI0000736 | CUGGGAGAGGGUUGUUUACUCC | 0.0829 | 0.0013 |
| hsa-miR-30c-2* | MI0000254 | CUGGGAGAAGGCUGUUUACUCU | 0.0938 | 0.0251 |
| hsa-miR-30d | MI0000255 | UGUAAACAUCCCCGACUGGAAG | 0.265 | 0.0062 |
| hsa-miR-30d* | MI0000255 | CUUUCAGUCAGAUGUUUGCUGC | 0.4337 | 0.0843 |
| hsa-miR-30e-3p | MI0000749 | CUUUCAGUCGGAUGUUUACAGC | 0.6673 | 0.2234 |
| hsa-miR-30e-5p | MI0000749 | UGUAAACAUCCUUGACUGGAAG | 0.2084 | 0.0056 |
| hsa-miR-31 | MI0000089 | AGGCAAGAUGCUGGCAUAGCU | 0.4773 | 0.0454 |
| hsa-miR-31* | MI0000089 | UGCUAUGCCAACAUAUUGCCAU | 1.3142 | 0.4881 |
| hsa-miR-32 | MI0000090 | UAUUGCACAUUACUAAGUUGCA | 0.5704 | 0.1087 |
| hsa-miR-32* | MI0000090 | CAAUUUAGUGUGUGUGAUAUUU | 0.1593 | 0.0051 |
| hsa-miR-320 | MI0000542 | AAAAGCUGGGUUGAGAGGGCGA | 0.9374 | 0.0604 |
| hsa-miR-323 | MI0000807 | CACAUUACACGGUCGACCUCU | 0.1433 | 0.0435 |
| hsa-miR-323-5p | MI0000807 | AGGUGGUCCGUGGCGCGUUCGC | 0.1283 | 0.006 |
| hsa-miR-324-3p | MI0000813 | ACUGCCCCAGGUGCUGCUGG | 0.743 | 0.0866 |
| hsa-miR-324-5p | MI0000813 | CGCAUCCCCUAGGGCAUUGGUGU | 1.2788 | 0.4387 |
| hsa-miR-325 | MI0000824 | CCUAGUAGGUGUCCAGUAAGUGU | 1.4794 | 0.1723 |
| hsa-miR-326 | MI0000808 | CCUCUGGGCCCUUCCUCCAG | 0.4147 | 0.0356 |
| hsa-miR-328 | MI0000804 | CUGGCCCUCUCUGCCCUUCCGU | 0.4309 | 0.0926 |
| hsa-miR-329 | MI0001725 | AACACACCUGGUUAACCUCUUU | 0.334 | 0.094 |
| hsa-miR-329 | MI0001726 | AACACACCUGGUUAACCUCUUU | 0.3908 | 0.0137 |
| hsa-miR-33 | MI0000091 | GUGCAUUGUAGUUGCAUUGCA | 0.3432 | 0.1361 |
| hsa-miR-330 | MI0000803 | GCAAAGCACACGGCCUGCAGAGA | 0.9215 | 0.1115 |
| hsa-miR-330-5p | MI0000803 | UCUCUGGGCCUGUGUCUUAGGC | 0.2342 | 0.0057 |
| hsa-miR-331 | MI0000812 | GCCCCUGGGCCUAUCCUAGAA | 0.7921 | 0.235 |
| hsa-miR-331-5p | MI0000812 | CUAGGUAUGGUCCCAGGGAUCC | 0.7886 | 0.0834 |
| hsa-miR-335 | MI0000816 | UCAAGAGCAAUAACGAAAAAUGU | 1.2408 | 0.4396 |
| hsa-miR-335* | MI0000816 | UUUUUCAUUAUUGCUCCUGACC | 0.5039 | 0.1429 |
| hsa-miR-337 | MI0000806 | CUCCUAUAUGAUGCCUUUCUUC | 0.6061 | 0.0765 |
| hsa-miR-337-5p | MI0000806 | GAACGGCUUCAUACAGGAGUU | 0.6446 | 0.0521 |
| hsa-miR-338 | MI0000814 | UCCAGCAUCAGUGAUUUUGUUG | 0.4524 | 0.0346 |
| hsa-miR-338-5p | MI0000814 | AACAAUAUCCUGGUGCUGAGUG | 1.0061 | 0.0616 |
| hsa-miR-339 | MI0000815 | UCCCUGUCCUCCAGGAGCUCACG | 0.2596 | 0.0541 |
| hsa-miR-339-3p | MI0000815 | UGAGCGCCUCGACGACAGAGCCG | 0.1937 | 0.0054 |
| hsa-miR-33a* | MI0000091 | CAAUGUUUCCACAGUGCAUCAC | 0.2938 | 0.0401 |
| hsa-miR-33b | MI0003646 | GUGCAUUGCUGUUGCAUUGC | 0.2982 | 0.1328 |
| hsa-miR-33b* | MI0003646 | CAGUGCCUCGGCAGUGCAGCCC | 0.8175 | 0.9408 |
| hsa-miR-340 | MI0000802 | UUAUAAAGCAAUGAGACUGAUU | 0.1694 | 0.0116 |
| hsa-miR-340* | MI0000802 | UCCGUCUCAGUUACUUUAUAGC | 0.228 | 0.0424 |
| hsa-miR-342 | MI0000805 | UCUCACACAGAAAUCGCACCCGU | 0.9723 | 0.2784 |
| hsa-miR-342-5p | MI0000805 | AGGGGUGCUAUCUGUGAUUGA | 0.0987 | 0.0151 |
| hsa-miR-345 | MI0000825 | GCUGACUCCUAGUCCAGGGCUC | 2.7413 | 0.7766 |
| hsa-miR-346 | MI0000826 | UGUCUGCCCGCAUGCCUGCCUCU | 0.576 | 0.1304 |
| hsa-miR-34a | MI0000268 | UGGCAGUGUCUUAGCUGGUUGU | 0.1426 | 0.0903 |
| hsa-miR-34a* | MI0000268 | CAAUCAGCAAGUAUACUGCCCU | 0.1737 | 0.0066 |
| hsa-miR-34b | MI0000742 | CAAUCACUAACUCCACUGCCAU | 0.5574 | 0.1264 |
| hsa-miR-34b* | MI0000742 | UAGGCAGUGUCAUUAGCUGAUUG | 0.1115 | 0.0225 |
| hsa-miR-34c | MI0000743 | AGGCAGUGUAGUUAGCUGAUUGC | 0.0949 | 0.0166 |
| hsa-miR-34c-3p | MI0000743 | AAUCACUAACCACACGGCCAGG | 0.8032 | 0.0635 |
| hsa-miR-361 | MI0000760 | UUAUCAGAAUCUCCAGGGGUAC | 0.7173 | 0.0517 |
| hsa-miR-361-3p | MI0000760 | UCCCCCAGGUGUGAUUCUGAUUU | 0.5828 | 0.041 |
| hsa-miR-362 | MI0000762 | AAUCCUUGGAACCUAGGUGUGAGU | 0.4209 | 0.0005 |
| hsa-miR-362-3p | MI0000762 | AACACACCUAUUCAAGGAUUCA | 0.1577 | 0.0099 |
| hsa-miR-363 | MI0000764 | AAUUGCACGGUAUCCAUCUGUA | 1.3278 | 0.4332 |
| hsa-miR-363* | MI0000764 | CGGGUGGAUCACGAUGCAAUUU | 0.1248 | 0.0688 |
| hsa-miR-365 | MI0000767 | UAAUGCCCCUAAAAAUCCUUAU | 0.2679 | 0.0331 |
| hsa-miR-365 | MI0000769 | UAAUGCCCCUAAAAAUCCUUAU | 0.3414 | 0.1369 |
| hsa-miR-367 | MI0000775 | AAUUGCACUUUAGCAAUGGUGA | 0.9608 | 0.1385 |
| hsa-miR-367* | MI0000775 | ACUGUUGCUAAUAUGCAACUCU | 0.1538 | 0.0036 |
| hsa-miR-368 | MI0000776 | AACAUAGAGGAAAUUCCACGU | 0.108 | 0.0209 |
| hsa-miR-369-3p | MI0000777 | AAUAAUACAUGGUUGAUCUUU | 0.6863 | 0.2575 |
| hsa-miR-369-5p | MI0000777 | AGAUCGACCGUGUUAUAUUCGC | 1.6786 | 0.0245 |
| hsa-miR-370 | MI0000778 | GCCUGCUGGGGUGGAACCUGGU | 0.5156 | 0.1003 |
| hsa-miR-371 | MI0000779 | AAGUGCCGCCAUCUUUUGAGUGU | 0.3413 | 0.1253 |
| hsa-miR-371-5p | MI0000779 | ACUCAAACUGUGGGGGCACU | 1.1668 | 0.2361 |
| hsa-miR-372 | MI0000780 | AAAGUGCUGCGACAUUUGAGCGU | 0.858 | 0.0173 |
| hsa-miR-373 | MI0000781 | GAAGUGCUUCGAUUUUGGGGUGU | 0.6894 | 0.1566 |
| hsa-miR-373* | MI0000781 | ACUCAAAAUGGGGGCGCUUUCC | 0.9863 | 0.3594 |
| hsa-miR-374 | MI0000782 | UUAUAAUACAACCUGAUAAGUG | 0.4019 | 0.0861 |
| hsa-miR-374a* | MI0000782 | CUUAUCAGAUUGUAUUGUAAUU | 0.7951 | 0.0484 |
| hsa-miR-374b | MI0005566 | AUAUAAUACAACCUGCUAAGUG | 0.2587 | 0.0711 |
| hsa-miR-374b* | MI0005566 | CUUAGCAGGUUGUAUUAUCAUU | 0.2803 | 0.0003 |
| hsa-miR-375 | MI0000783 | UUUGUUCGUUCGGCUCGCGUGA | 0.4931 | 0.1343 |
| hsa-miR-376a | MI0000784 | AUCAUAGAGGAAAAUCCACGU | 0.3053 | 0.0914 |
| hsa-miR-376a | MI0003529 | AUCAUAGAGGAAAAUCCACGU | 0.3481 | 0.0309 |
| hsa-miR-376a* | MI0000784 | GUAGAUUCUCCUUCUAUGAGUA | 0.2435 | 0.1356 |
| hsa-miR-376b | MI0002466 | AUCAUAGAGGAAAAUCCAUGUU | 0.2247 | 0.3106 |
| hsa-miR-377 | MI0000785 | AUCACACAAAGGCAACUUUUGU | 0.6156 | 0.171 |
| hsa-miR-377* | MI0000785 | AGAGGUUGCCCUUGGUGAAUUC | 0.1556 | 0.0165 |
| hsa-miR-378 | MI0000786 | CUCCUGACUCCAGGUCCUGUGU | 0.1788 | 0.0077 |
| hsa-miR-379 | MI0000787 | UGGUAGACUAUGGAACGUAGG | 0.22 | 0.026 |
| hsa-miR-379* | MI0000787 | UAUGUAACAUGGUCCACUAACU | 0.2942 | 0.2168 |
| hsa-miR-380-3p | MI0000788 | UAUGUAAUAUGGUCCACAUCUU | 0.3707 | 0.0412 |
| hsa-miR-380-5p | MI0000788 | UGGUUGACCAUAGAACAUGCGC | 0.2004 | 0.0095 |
| hsa-miR-381 | MI0000789 | UAUACAAGGGCAAGCUCUCUGU | 2.7113 | 0.7712 |
| hsa-miR-382 | MI0000790 | GAAGUUGUUCGUGGUGGAUUCG | 0.6518 | 0.0227 |
| hsa-miR-383 | MI0000791 | AGAUCAGAAGGUGAUUGUGGCU | 0.615 | 0.2484 |
| hsa-miR-384 | MI0001145 | AUUCCUAGAAAUUGUUCAUA | 0.9046 | 0.2784 |
| hsa-miR-409-3p | MI0001735 | GAAUGUUGCUCGGUGAACCCCU | 0.4155 | 0.147 |
| hsa-miR-409-5p | MI0001735 | AGGUUACCCGAGCAACUUUGCAU | 0.7637 | 0.2695 |
| hsa-miR-410 | MI0002465 | AAUAUAACACAGAUGGCCUGU | 1.1196 | 0.4515 |
| hsa-miR-411 | MI0003675 | UAGUAGACCGUAUAGCGUACG | 0.3883 | 0.0397 |
| hsa-miR-411* | MI0003675 | UAUGUAACACGGUCCACUAACC | 0.3832 | 0.0473 |
| hsa-miR-412 | MI0002464 | ACUUCACCUGGUCCACUAGCCGU | 1.9228 | 0.1319 |
| hsa-miR-421 | MI0003685 | AUCAACAGACAUUAAUUGGGCGC | 0.4464 | 0.0874 |
| hsa-miR-422a | MI0001444 | ACUGGACUUAGGGUCAGAAGGC | 0.4437 | 0.1225 |
| hsa-miR-422b | MI0000786 | ACUGGACUUGGAGUCAGAAGG | 0.4188 | 0.2381 |
| hsa-miR-423 | MI0001445 | AGCUCGGUCUGAGGCCCCUCAGU | 0.377 | 0.0817 |
| hsa-miR-423-5p | MI0001445 | UGAGGGGCAGAGAGCGAGACUUU | 0.3725 | 0.0291 |
| hsa-miR-424 | MI0001446 | CAGCAGCAAUUCAUGUUUUGAA | 0.1496 | 0.009 |
| hsa-miR-424* | MI0001446 | CAAAACGUGAGGCGCUGCUAU | 0.2311 | 0.0417 |
| hsa-miR-425 | MI0001448 | AUCGGGAAUGUCGUGUCCGCCC | 0.6994 | 0.2785 |
| hsa-miR-425-5p | MI0001448 | AAUGACACGAUCACUCCCGUUGA | 0.294 | 0.0265 |
| hsa-miR-429 | MI0001641 | UAAUACUGUCUGGUAAAACCGU | 1.181 | 0.2903 |
| hsa-miR-431 | MI0001721 | UGUCUUGCAGGCCGUCAUGCA | 0.7446 | 0.2177 |
| hsa-miR-431* | MI0001721 | CAGGUCGUCUUGCAGGGCUUCU | 0.3943 | 0.0196 |
| hsa-miR-432 | MI0003133 | UCUUGGAGUAGGUCAUUGGGUGG | 0.3091 | 0.4301 |
| hsa-miR-432* | MI0003133 | CUGGAUGGCUCCUCCAUGUCU | 2.5292 | 0.1031 |
| hsa-miR-433 | MI0001723 | AUCAUGAUGGGCUCCUCGGUGU | 1.1481 | 0.2361 |
| hsa-miR-448 | MI0001637 | UUGCAUAUGUAGGAUGUCCCAU | 0.3684 | 0.1446 |
| hsa-miR-449 | MI0001648 | UGGCAGUGUAUUGUUAGCUGGU | 0.13 | 0.0396 |
| hsa-miR-449b | MI0003673 | AGGCAGUGUAUUGUUAGCUGGC | 0.1291 | 0.012 |
| hsa-miR-450 | MI0003187 | UUUUGCGAUGUGUUCCUAAUAU | 0.4284 | 0.0203 |
| hsa-miR-450 | MI0001652 | UUUUGCGAUGUGUUCCUAAUAU | 0.6854 | 0.3838 |
| hsa-miR-450b-3p | MI0005531 | UUGGGAUCAUUUUGCAUCCAUA | 0.146 | 0.0022 |
| hsa-miR-450b-5p | MI0005531 | UUUUGCAAUAUGUUCCUGAAUA | 0.3999 | 0.0015 |
| hsa-miR-451 | MI0001729 | AAACCGUUACCAUUACUGAGUU | 0.8864 | 0.201 |
| hsa-miR-452 | MI0001733 | AACUGUUUGCAGAGGAAACUGA | 0.6571 | 0.0409 |
| hsa-miR-452* | MI0001733 | CUCAUCUGCAAAGAAGUAAGUG | 0.2778 | 0.0488 |
| hsa-miR-453 | MI0001727 | AGGUUGUCCGUGGUGAGUUCGCA | 0.2291 | 0.1329 |
| hsa-miR-454-3p | MI0003820 | UAGUGCAAUAUUGCUUAUAGGGU | 0.4689 | 0.0054 |
| hsa-miR-454-5p | MI0003820 | ACCCUAUCAAUAUUGUCUCUGC | 0.5851 | 0.1356 |
| hsa-miR-455 | MI0003513 | UAUGUGCCUUUGGACUACAUCG | 2.3175 | 1.0497 |
| hsa-miR-455-3p | MI0003513 | GCAGUCCAUGGGCAUAUACAC | 1.0476 | 0.1832 |
| hsa-miR-483 | MI0002467 | UCACUCCUCUCCUCCCGUCUU | 0.8641 | 0.4857 |
| hsa-miR-483-5p | MI0002467 | AAGACGGGAGGAAAGAAGGGAG | 0.4443 | 0.0158 |
| hsa-miR-484 | MI0002468 | UCAGGCUCAGUCCCCUCCCGAU | 1.4347 | 0.3388 |
| hsa-miR-485-3p | MI0002469 | GUCAUACACGGCUCUCCUCUCU | 1.0682 | 0.1286 |
| hsa-miR-485-5p | MI0002469 | AGAGGCUGGCCGUGAUGAAUUC | 0.2519 | 0.0075 |
| hsa-miR-486 | MI0002470 | UCCUGUACUGAGCUGCCCCGAG | 0.9849 | 0.0568 |
| hsa-miR-486-3p | MI0002470 | CGGGGCAGCUCAGUACAGGAU | 0.2473 | 0.0015 |
| hsa-miR-487 | MI0002471 | AAUCAUACAGGGACAUCCAGUU | 0.5187 | 0.1022 |
| hsa-miR-487b | MI0003530 | AAUCGUACAGGGUCAUCCACUU | 0.3751 | 0.056 |
| hsa-miR-488 | MI0003123 | UUGAAAGGCUAUUUCUUGGUC | 0.2244 | 0.0381 |
| hsa-miR-488* | MI0003123 | CCCAGAUAAUGGCACUCUCAA | 0.2477 | 0.0635 |
| hsa-miR-489 | MI0003124 | GUGACAUCACAUAUACGGCAGC | 0.088 | 0.0168 |
| hsa-miR-490 | MI0003125 | CAACCUGGAGGACUCCAUGCUG | 1.1432 | 0.493 |
| hsa-miR-490-5p | MI0003125 | CCAUGGAUCUCCAGGUGGGU | 0.281 | 0.0207 |
| hsa-miR-491 | MI0003126 | AGUGGGGAACCCUUCCAUGAGG | 0.2151 | 0.1127 |
| hsa-miR-491-3p | MI0003126 | CUUAUGCAAGAUUCCCUUCUAC | 0.3037 | 0.0182 |
| hsa-miR-492 | MI0003131 | AGGACCUGCGGGACAAGAUUCUU | 0.6161 | 0.1149 |
| hsa-miR-493 | MI0003132 | UUGUACAUGGUAGGCUUUCAUU | 0.2877 | 0.0315 |
| hsa-miR-493-3p | MI0003132 | UGAAGGUCUACUGUGUGCCAGG | 1.3226 | 0.5189 |
| hsa-miR-494 | MI0003134 | UGAAACAUACACGGGAAACCUC | 0.2324 | 0.0808 |
| hsa-miR-495 | MI0003135 | AAACAAACAUGGUGCACUUCUU | 1.7524 | 0.6524 |
| hsa-miR-496 | MI0003136 | UGAGUAUUACAUGGCCAAUCUC | 0.3236 | 0.0701 |
| hsa-miR-497 | MI0003138 | CAGCAGCACACUGUGGUUUGU | 0.1418 | 0.004 |
| hsa-miR-497* | MI0003138 | CAAACCACACUGUGGUGUUAGA | 0.5316 | 0.0485 |
| hsa-miR-498 | MI0003142 | UUUCAAGCCAGGGGGCGUUUUUC | 0.3869 | 0.0351 |
| hsa-miR-499 | MI0003183 | UUAAGACUUGCAGUGAUGUUU | 0.3716 | 0.0987 |
| hsa-miR-499-3p | MI0003183 | AACAUCACAGCAAGUCUGUGCU | 0.1436 | 0.0201 |
| hsa-miR-500 | MI0003184 | UAAUCCUUGCUACCUGGGUGAGA | 0.4813 | 0.0012 |
| hsa-miR-500* | MI0003184 | AUGCACCUGGGCAAGGAUUCUG | 0.6646 | 0.0251 |
| hsa-miR-501 | MI0003185 | AAUCCUUUGUCCCUGGGUGAGA | 3.0075 | 0.5541 |
| hsa-miR-501-3p | MI0003185 | AAUGCACCCGGGCAAGGAUUCU | 0.373 | 0.0085 |
| hsa-miR-502 | MI0003186 | AUCCUUGCUAUCUGGGUGCUA | 1.4643 | 0.1613 |
| hsa-miR-502-3p | MI0003186 | AAUGCACCUGGGCAAGGAUUCA | 0.254 | 0.0194 |
| hsa-miR-503 | MI0003188 | UAGCAGCGGGAACAGUUCUGCAG | 2.9368 | 0.7522 |
| hsa-miR-504 | MI0003189 | AGACCCUGGUCUGCACUCUAUC | 0.9782 | 0.1317 |
| hsa-miR-505 | MI0003190 | CGUCAACACUUGCUGGUUUCCU | 0.8659 | 0.0794 |
| hsa-miR-505* | MI0003190 | GGGAGCCAGGAAGUAUUGAUGU | 0.6778 | 0.0874 |
| hsa-miR-506 | MI0003193 | UAAGGCACCCUUCUGAGUAGA | 0.2518 | 0.1094 |
| hsa-miR-507 | MI0003194 | UUUUGCACCUUUUGGAGUGAA | 0.5606 | 0.0934 |
| hsa-miR-508 | MI0003195 | UGAUUGUAGCCUUUUGGAGUAGA | 0.7816 | 0.0427 |
| hsa-miR-508-5p | MI0003195 | UACUCCAGAGGGCGUCACUCAUG | 0.452 | 0.0103 |
| hsa-miR-509 | MI0003196 | UGAUUGGUACGUCUGUGGGUAG | 0.2068 | 0.0212 |
| hsa-miR-509-3-5p | MI0005717 | UACUGCAGACGUGGCAAUCAUG | 0.1124 | 0.0126 |
| hsa-miR-509-3p | MI0005530 | UGAUUGGUACGUCUGUGGGUAG | 0.1697 | 0.0018 |
| hsa-miR-509-3p | MI0005717 | UGAUUGGUACGUCUGUGGGUAG | 0.2357 | 0.0808 |
| hsa-miR-509-5p | MI0003196 | UACUGCAGACAGUGGCAAUCA | 0.209 | 0.0616 |
| hsa-miR-509-5p | MI0005530 | UACUGCAGACAGUGGCAAUCA | 0.2117 | 0.0577 |
| hsa-miR-510 | MI0003197 | UACUCAGGAGAGUGGCAAUCAC | 0.1731 | 0.1183 |
| hsa-miR-511 | MI0003127 | GUGUCUUUUGCUCUGCAGUCA | 0.8434 | 0.6051 |
| hsa-miR-511 | MI0003128 | GUGUCUUUUGCUCUGCAGUCA | 0.9157 | 0.7074 |
| hsa-miR-512-3p | MI0003140 | AAGUGCUGUCAUAGCUGAGGUC | 0.6081 | 0.0676 |
| hsa-miR-512-3p | MI0003141 | AAGUGCUGUCAUAGCUGAGGUC | 0.6787 | 0.1675 |
| hsa-miR-512-5p | MI0003140 | CACUCAGCCUUGAGGGCACUUUC | 0.9802 | 1.1307 |
| hsa-miR-512-5p | MI0003141 | CACUCAGCCUUGAGGGCACUUUC | 1.0269 | 1.0647 |
| hsa-miR-513 | MI0003191 | UUCACAGGGAGGUGUCAU | 0.7004 | 0.1379 |
| hsa-miR-513 | MI0003192 | UUCACAGGGAGGUGUCAU | 0.7277 | 0.1764 |
| hsa-miR-513a-3p | MI0003191 | UAAAUUUCACCUUUCUGAGAAGG | 0.1258 | 0.0063 |
| hsa-miR-513a-3p | MI0003192 | UAAAUUUCACCUUUCUGAGAAGG | 0.1322 | 0.0026 |
| hsa-miR-513b | MI0006648 | UUCACAAGGAGGUGUCAUUUAU | 1.1569 | 0.2837 |
| hsa-miR-513c | MI0006649 | UUCUCAAGGAGGUGUCGUUUAU | 0.494 | 0.1305 |
| hsa-miR-514 | MI0003198 | AUUGACACUUCUGUGAGUAGA | 0.8166 | 0.1481 |
| hsa-miR-514 | MI0003199 | AUUGACACUUCUGUGAGUAGA | 1.0327 | 0.1574 |
| hsa-miR-514 | MI0003200 | AUUGACACUUCUGUGAGUAGA | 1.5161 | 1.1373 |
| hsa-miR-515-3p | MI0003144 | GAGUGCCUUCUUUUGGAGCGUU | 0.5429 | 0.057 |
| hsa-miR-515-3p | MI0003147 | GAGUGCCUUCUUUUGGAGCGUU | 0.7006 | 0.28 |
| hsa-miR-515-5p | MI0003144 | UUCUCCAAAAGAAAGCACUUUCUG | 1.1354 | 0.3732 |
| hsa-miR-515-5p | MI0003147 | UUCUCCAAAAGAAAGCACUUUCUG | 1.1575 | 0.342 |
| hsa-miR-516-3p | MI0003167 | UGCUUCCUUUCAGAGGGU | 0.3772 | 0.0002 |
| hsa-miR-516-3p | MI0003172 | UGCUUCCUUUCAGAGGGU | 0.5794 | 0.2856 |
| hsa-miR-516-3p | MI0003180 | UGCUUCCUUUCAGAGGGU | 1.0536 | 0.8101 |
| hsa-miR-516-3p | MI0003181 | UGCUUCCUUUCAGAGGGU | 1.2039 | 0.5975 |
| hsa-miR-516-5p | MI0003167 | AUCUGGAGGUAAGAAGCACUUU | 0.2167 | 0.0517 |
| hsa-miR-516-5p | MI0003172 | AUCUGGAGGUAAGAAGCACUUU | 0.2479 | 0.0959 |
| hsa-miR-516a-5p | MI0003180 | UUCUCGAGGAAAGAAGCACUUUC | 0.4813 | 0.2025 |
| hsa-miR-516a-5p | MI0003181 | UUCUCGAGGAAAGAAGCACUUUC | 0.5086 | 0.2411 |
| hsa-miR-517* | MI0003161 | CCUCUAGAUGGAAGCACUGUCU | 1.0497 | 0.1457 |
| hsa-miR-517* | MI0003165 | CCUCUAGAUGGAAGCACUGUCU | 1.0634 | 0.165 |
| hsa-miR-517* | MI0003174 | CCUCUAGAUGGAAGCACUGUCU | 1.1257 | 0.0382 |
| hsa-miR-517a | MI0003161 | AUCGUGCAUCCCUUUAGAGUGU | 0.4409 | 0.0615 |
| hsa-miR-517b | MI0003165 | UCGUGCAUCCCUUUAGAGUGUU | 0.4823 | 0.0277 |
| hsa-miR-517c | MI0003174 | AUCGUGCAUCCUUUUAGAGUGU | 0.3239 | 0.1458 |
| hsa-miR-518a | MI0003170 | GAAAGCGCUUCCCUUUGCUGGA | 0.1861 | 0.0222 |
| hsa-miR-518a | MI0003173 | GAAAGCGCUUCCCUUUGCUGGA | 0.2786 | 0.153 |
| hsa-miR-518a-5p | MI0003170 | CUGCAAAGGGAAGCCCUUUC | 0.1154 | 0.0054 |
| hsa-miR-518a-5p | MI0003173 | CUGCAAAGGGAAGCCCUUUC | 0.1235 | 0.0168 |
| hsa-miR-518b | MI0003156 | CAAAGCGCUCCCCUUUAGAGGU | 0.1628 | 0.013 |
| hsa-miR-518c | MI0003159 | CAAAGCGCUUCUCUUUAGAGUGU | 0.3495 | 0.182 |
| hsa-miR-518c* | MI0003159 | UCUCUGGAGGGAAGCACUUUCUG | 0.6023 | 0.0713 |
| hsa-miR-518d | MI0003171 | CAAAGCGCUUCCCUUUGGAGC | 0.1665 | 0.0118 |
| hsa-miR-518d-5p | MI0003171 | CUCUAGAGGGAAGCACUUUCUG | 0.6836 | 0.7841 |
| hsa-miR-518e | MI0003169 | AAAGCGCUUCCCUUCAGAGUG | 0.5827 | 0.1987 |
| hsa-miR-518e* | MI0003169 | CUCUAGAGGGAAGCGCUUUCUG | 0.6794 | 0.0125 |
| hsa-miR-518f | MI0003154 | GAAAGCGCUUCUCUUUAGAGG | 0.7424 | 0.3709 |
| hsa-miR-518f* | MI0003154 | CUCUAGAGGGAAGCACUUUCUC | 1.2981 | 0.3006 |
| hsa-miR-519a | MI0003178 | AAAGUGCAUCCUUUUAGAGUGU | 0.8176 | 0.3271 |
| hsa-miR-519a | MI0003182 | AAAGUGCAUCCUUUUAGAGUGU | 1.2284 | 0.2537 |
| hsa-miR-519a* | MI0003178 | CUCUAGAGGGAAGCGCUUUCUG | 0.5545 | 0.0824 |
| hsa-miR-519b | MI0003151 | AAAGUGCAUCCUUUUAGAGGUU | 1.0877 | 0.5933 |
| hsa-miR-519b-5p | MI0003151 | CUCUAGAGGGAAGCGCUUUCUG | 0.6109 | 0.0679 |
| hsa-miR-519c | MI0003148 | AAAGUGCAUCUUUUUAGAGGAU | 1.047 | 0.1061 |
| hsa-miR-519d | MI0003162 | CAAAGUGCCUCCCUUUAGAGUG | 1.3952 | 0.7243 |
| hsa-miR-519e | MI0003145 | AAGUGCCUCCUUUUAGAGUGUU | 0.4321 | 0.151 |
| hsa-miR-519e* | MI0003145 | UUCUCCAAAAGGGAGCACUUUC | 0.8423 | 0.1732 |
| hsa-miR-520a | MI0003149 | AAAGUGCUUCCCUUUGGACUGU | 0.6985 | 0.0632 |
| hsa-miR-520a* | MI0003149 | CUCCAGAGGGAAGUACUUUCU | 0.428 | 0.0919 |
| hsa-miR-520b | MI0003155 | AAAGUGCUUCCUUUUAGAGGG | 0.8332 | 0.1202 |
| hsa-miR-520c | MI0003158 | AAAGUGCUUCCUUUUAGAGGGU | 0.7911 | 0.4114 |
| hsa-miR-520c-5p | MI0003158 | CUCUAGAGGGAAGCACUUUCUG | 0.9328 | 0.0525 |
| hsa-miR-520d | MI0003164 | AAAGUGCUUCUCUUUGGUGGGU | 1.1439 | 0.1855 |
| hsa-miR-520d* | MI0003164 | CUACAAAGGGAAGCCCUUUC | 0.633 | 0.2098 |
| hsa-miR-520e | MI0003143 | AAAGUGCUUCCUUUUUGAGGG | 0.8057 | 0.1805 |
| hsa-miR-520f | MI0003146 | AAGUGCUUCCUUUUAGAGGGUU | 0.718 | 0.0254 |
| hsa-miR-520g | MI0003166 | ACAAAGUGCUUCCCUUUAGAGUGU | 1.3553 | 0.0346 |
| hsa-miR-520h | MI0003175 | ACAAAGUGCUUCCCUUUAGAGU | 1.3289 | 0.2966 |
| hsa-miR-521 | MI0003163 | AACGCACUUCCCUUUAGAGUGU | 0.3097 | 0.0162 |
| hsa-miR-521 | MI0003176 | AACGCACUUCCCUUUAGAGUGU | 0.3632 | 0.0593 |
| hsa-miR-522 | MI0003177 | AAAAUGGUUCCCUUUAGAGUGU | 0.0692 | 0.0291 |
| hsa-miR-522* | MI0003177 | CUCUAGAGGGAAGCGCUUUCUG | 0.573 | 0.114 |
| hsa-miR-523 | MI0003153 | GAACGCGCUUCCCUAUAGAGGGU | 1.6484 | 0.6577 |
| hsa-miR-523* | MI0003153 | CUCUAGAGGGAAGCGCUUUCUG | 0.5991 | 0.1244 |
| hsa-miR-524 | MI0003160 | GAAGGCGCUUCCCUUUGGAGU | 0.6029 | 0.1676 |
| hsa-miR-524* | MI0003160 | CUACAAAGGGAAGCACUUUCUC | 0.2082 | 0.2875 |
| hsa-miR-525 | MI0003152 | CUCCAGAGGGAUGCACUUUCU | 0.3466 | 0.0485 |
| hsa-miR-525* | MI0003152 | GAAGGCGCUUCCCUUUAGAGCG | 0.9428 | 0.429 |
| hsa-miR-526a | MI0003157 | CUCUAGAGGGAAGCACUUUCUG | 0.9205 | 0.0668 |
| hsa-miR-526a | MI0003168 | CUCUAGAGGGAAGCACUUUCUG | 1.2111 | 0.4778 |
| hsa-miR-526b | MI0003150 | CUCUUGAGGGAAGCACUUUCUGU | 0.2488 | 0.1013 |
| hsa-miR-526b* | MI0003150 | GAAAGUGCUUCCUUUUAGAGGC | 0.9913 | 0.3819 |
| hsa-miR-526c | MI0003148 | CUCUAGAGGGAAGCGCUUUCUG | 0.8964 | 0.3463 |
| hsa-miR-527 | MI0003179 | CUGCAAAGGGAAGCCCUUUC | 0.1343 | 0.0288 |
| hsa-miR-532 | MI0003205 | CAUGCCUUGAGUGUAGGACCGU | 0.1929 | 0.0152 |
| hsa-miR-532-3p | MI0003205 | CCUCCCACACCCAAGGCUUGCA | 0.6777 | 0.0594 |
| hsa-miR-539 | MI0003514 | GGAGAAAUUAUCCUUGGUGUGU | 0.848 | 0.0054 |
| hsa-miR-541 | MI0005539 | UGGUGGGCACAGAAUCUGGACU | 0.0624 | 0.0006 |
| hsa-miR-541* | MI0005539 | AAAGGAUUCUGCUGUCGGUCCCACU | 0.763 | 0.1417 |
| hsa-miR-542-3p | MI0003686 | UGUGACAGAUUGAUAACUGAAA | 0.3562 | 0.0278 |
| hsa-miR-542-5p | MI0003686 | UCGGGGAUCAUCAUGUCACGAGA | 0.4466 | 0.0653 |
| hsa-miR-543 | MI0005565 | AAACAUUCGCGGUGCACUUCUU | 0.7538 | 0.0263 |
| hsa-miR-544 | MI0003515 | AUUCUGCAUUUUUAGCAAGUUC | 0.2346 | 0.1385 |
| hsa-miR-545 | MI0003516 | UCAGCAAACAUUUAUUGUGUGC | 0.8985 | 0.1759 |
| hsa-miR-545* | MI0003516 | UCAGUAAAUGUUUAUUAGAUGA | 1.2125 | 0.0602 |
| hsa-miR-548a | MI0003593 | CAAAACUGGCAAUUACUUUUGC | 0.3061 | 0.1166 |
| hsa-miR-548a | MI0003598 | CAAAACUGGCAAUUACUUUUGC | 0.3151 | 0.1294 |
| hsa-miR-548a | MI0003612 | CAAAACUGGCAAUUACUUUUGC | 0.5185 | 0.1583 |
| hsa-miR-548a-5p | MI0003612 | AAAAGUAAUUGCGAGUUUUACC | 1.3073 | 0.2119 |
| hsa-miR-548b | MI0003596 | CAAGAACCUCAGUUGCUUUUGU | 1.1479 | 0.1886 |
| hsa-miR-548b-5p | MI0003596 | AAAAGUAAUUGUGGUUUUGGCC | 0.8871 | 0.0005 |
| hsa-miR-548c | MI0003630 | CAAAAAUCUCAAUUACUUUUGC | 0.9981 | 0.3908 |
| hsa-miR-548c-5p | MI0003630 | AAAAGUAAUUGCGGUUUUUGCC | 1.1931 | 0.0713 |
| hsa-miR-548d | MI0003668 | CAAAAACCACAGUUUCUUUUGC | 0.7525 | 0.2965 |
| hsa-miR-548d | MI0003671 | CAAAAACCACAGUUUCUUUUGC | 1.0003 | 0.0539 |
| hsa-miR-548d-5p | MI0003668 | AAAAGUAAUUGUGGUUUUUGCC | 2.3348 | 0.4806 |
| hsa-miR-548d-5p | MI0003671 | AAAAGUAAUUGUGGUUUUUGCC | 2.5291 | 0.2058 |
| hsa-miR-549 | MI0003679 | UGACAACUAUGGAUGAGCUCU | 1.1718 | 0.1411 |
| hsa-miR-550 | MI0003600 | UGUCUUACUCCCUCAGGCACAU | 0.3702 | 0.0385 |
| hsa-miR-550 | MI0003601 | UGUCUUACUCCCUCAGGCACAU | 0.4224 | 0.0352 |
| hsa-miR-550 | MI0003600 | AGUGCCUGAGGGAGUAAGAGCCC | 0.6411 | 0.1777 |
| hsa-miR-550 | MI0003601 | AGUGCCUGAGGGAGUAAGAGCCC | 0.6489 | 0.1667 |
| hsa-miR-551a | MI0003556 | GCGACCCACUCUUGGUUUCCA | 0.5489 | 0.7694 |
| hsa-miR-551b | MI0003575 | GCGACCCAUACUUGGUUUCAG | 1.6407 | 0.2598 |
| hsa-miR-551b* | MI0003575 | GAAAUCAAGCGUGGGUGAGACC | 1.1129 | 0.1244 |
| hsa-miR-552 | MI0003557 | AACAGGUGACUGGUUAGACAA | 0.1423 | 0.0123 |
| hsa-miR-553 | MI0003558 | AAAACGGUGAGAUUUUGUUUU | 0.8464 | 0.1747 |
| hsa-miR-554 | MI0003559 | GCUAGUCCUGACUCAGCCAGU | 0.954 | 0.035 |
| hsa-miR-555 | MI0003561 | AGGGUAAGCUGAACCUCUGAU | 0.1481 | 0.0171 |
| hsa-miR-556 | MI0003562 | GAUGAGCUCAUUGUAAUAUGAG | 1.1704 | 0.5141 |
| hsa-miR-556-3p | MI0003562 | AUAUUACCAUUAGCUCAUCUUU | 0.4775 | 0.2473 |
| hsa-miR-557 | MI0003563 | GUUUGCACGGGUGGGCCUUGUCU | 0.3681 | 0.0742 |
| hsa-miR-558 | MI0003564 | UGAGCUGCUGUACCAAAAU | 0.3047 | 0.0532 |
| hsa-miR-559 | MI0003565 | UAAAGUAAAUAUGCACCAAAA | 1.0623 | 0.4212 |
| hsa-miR-561 | MI0003567 | CAAAGUUUAAGAUCCUUGAAGU | 0.6369 | 0.0626 |
| hsa-miR-562 | MI0003568 | AAAGUAGCUGUACCAUUUGC | 0.3047 | 0.0678 |
| hsa-miR-563 | MI0003569 | AGGUUGACAUACGUUUCCC | 0.1268 | 0.1724 |
| hsa-miR-564 | MI0003570 | AGGCACGGUGUCAGCAGGC | 0.475 | 0.0428 |
| hsa-miR-566 | MI0003572 | GGGCGCCUGUGAUCCCAAC | 1.516 | 0.1086 |
| hsa-miR-567 | MI0003573 | AGUAUGUUCUUCCAGGACAGAAC | 0.4129 | 0.0005 |
| hsa-miR-568 | MI0003574 | AUGUAUAAAUGUAUACACAC | 1.3692 | 0.1559 |
| hsa-miR-569 | MI0003576 | AGUUAAUGAAUCCUGGAAAGU | 0.9075 | 0.1647 |
| hsa-miR-570 | MI0003577 | CGAAAACAGCAAUUACCUUUGC | 0.5763 | 0.1782 |
| hsa-miR-571 | MI0003578 | UGAGUUGGCCAUCUGAGUGAG | 0.2537 | 0.0126 |
| hsa-miR-572 | MI0003579 | GUCCGCUCGGCGGUGGCCCA | 1.2415 | 0.3197 |
| hsa-miR-573 | MI0003580 | CUGAAGUGAUGUGUAACUGAUCAG | 0.8689 | 0.1453 |
| hsa-miR-574 | MI0003581 | CACGCUCAUGCACACACCCACA | 0.7929 | 0.2309 |
| hsa-miR-574-5p | MI0003581 | UGAGUGUGUGUGUGUGAGUGUGU | 0.3496 | 0.0411 |
| hsa-miR-575 | MI0003582 | GAGCCAGUUGGACAGGAGC | 0.3012 | 0.4186 |
| hsa-miR-576 | MI0003583 | AUUCUAAUUUCUCCACGUCUUU | 0.928 | 0.2498 |
| hsa-miR-576-3p | MI0003583 | AAGAUGUGGAAAAAUUGGAAUC | 0.1277 | 0.0133 |
| hsa-miR-577 | MI0003584 | UAGAUAAAAUAUUGGUACCUG | 2.7008 | 0.1797 |
| hsa-miR-578 | MI0003585 | CUUCUUGUGCUCUAGGAUUGU | 0.2719 | 0.0383 |
| hsa-miR-579 | MI0003586 | UUCAUUUGGUAUAAACCGCGAUU | 0.3209 | 0.1023 |
| hsa-miR-580 | MI0003587 | UUGAGAAUGAUGAAUCAUUAGG | 0.6049 | 0.016 |
| hsa-miR-581 | MI0003588 | UCUUGUGUUCUCUAGAUCAGU | 0.9186 | 0.0773 |
| hsa-miR-582 | MI0003589 | UUACAGUUGUUCAACCAGUUACU | 0.5184 | 0.0253 |
| hsa-miR-582-3p | MI0003589 | UAACUGGUUGAACAACUGAACC | 0.3706 | 0.0178 |
| hsa-miR-583 | MI0003590 | CAAAGAGGAAGGUCCCAUUAC | 0.6594 | 0.0907 |
| hsa-miR-584 | MI0003591 | UUAUGGUUUGCCUGGGACUGAG | 0.8115 | 0.0869 |
| hsa-miR-585 | MI0003592 | UGGGCGUAUCUGUAUGCUA | 0.2489 | 0.1649 |
| hsa-miR-586 | MI0003594 | UAUGCAUUGUAUUUUUAGGUCC | 0.7329 | 0.2088 |
| hsa-miR-587 | MI0003595 | UUUCCAUAGGUGAUGAGUCAC | 1.4893 | 0.1944 |
| hsa-miR-588 | MI0003597 | UUGGCCACAAUGGGUUAGAAC | 0.31 | 0.0598 |
| hsa-miR-589 | MI0003599 | UGAGAACCACGUCUGCUCUGAG | 0.2739 | 0.0299 |
| hsa-miR-589* | MI0003599 | UCAGAACAAAUGCCGGUUCCCAGA | 1.0017 | 0.1097 |
| hsa-miR-590 | MI0003602 | GAGCUUAUUCAUAAAAGUGCAG | 1.1345 | 0.0923 |
| hsa-miR-590-3p | MI0003602 | UAAUUUUAUGUAUAAGCUAGU | 0.8691 | 0.004 |
| hsa-miR-591 | MI0003603 | AGACCAUGGGUUCUCAUUGU | 0.2665 | 0.0935 |
| hsa-miR-592 | MI0003604 | UUGUGUCAAUAUGCGAUGAUGU | 0.4337 | 0.0789 |
| hsa-miR-593 | MI0003605 | UGUCUCUGCUGGGGUUUCU | 0.6185 | 0.3368 |
| hsa-miR-593* | MI0003605 | AGGCACCAGCCAGGCAUUGCUCAGC | 0.3059 | 0.0916 |
| hsa-miR-595 | MI0003607 | GAAGUGUGCCGUGGUGUGUCU | 0.7901 | 0.2883 |
| hsa-miR-596 | MI0003608 | AAGCCUGCCCGGCUCCUCGGG | 0.4125 | 0.2546 |
| hsa-miR-597 | MI0003609 | UGUGUCACUCGAUGACCACUGU | 0.7892 | 0.1268 |
| hsa-miR-598 | MI0003610 | UACGUCAUCGUUGUCAUCGUCA | 1.0992 | 0.2759 |
| hsa-miR-599 | MI0003611 | GUUGUGUCAGUUUAUCAAAC | 0.6531 | 0.2141 |
| hsa-miR-600 | MI0003613 | ACUUACAGACAAGAGCCUUGCUC | 0.4571 | 0.153 |
| hsa-miR-601 | MI0003614 | UGGUCUAGGAUUGUUGGAGGAG | 0.2949 | 0.0354 |
| hsa-miR-602 | MI0003615 | GACACGGGCGACAGCUGCGGCCC | 1.0751 | 0.1889 |
| hsa-miR-603 | MI0003616 | CACACACUGCAAUUACUUUUGC | 0.3123 | 0.0689 |
| hsa-miR-604 | MI0003617 | AGGCUGCGGAAUUCAGGAC | 0.1927 | 0.0527 |
| hsa-miR-605 | MI0003618 | UAAAUCCCAUGGUGCCUUCUCCU | 0.4768 | 0.1915 |
| hsa-miR-606 | MI0003619 | AAACUACUGAAAAUCAAAGAU | 0.7084 | 0.4157 |
| hsa-miR-607 | MI0003620 | GUUCAAAUCCAGAUCUAUAAC | 0.8008 | 0.2185 |
| hsa-miR-608 | MI0003621 | AGGGGUGGUGUUGGGACAGCUCCGU | 0.1398 | 0.0374 |
| hsa-miR-609 | MI0003622 | AGGGUGUUUCUCUCAUCUCU | 0.3011 | 0.0974 |
| hsa-miR-610 | MI0003623 | UGAGCUAAAUGUGUGCUGGGA | 1.5087 | 0.4501 |
| hsa-miR-611 | MI0003624 | GCGAGGACCCCUCGGGGUCUGAC | 1.4257 | 0.3272 |
| hsa-miR-612 | MI0003625 | GCUGGGCAGGGCUUCUGAGCUCCUU | 0.4547 | 0.1694 |
| hsa-miR-613 | MI0003626 | AGGAAUGUUCCUUCUUUGCC | 0.2859 | 0.1262 |
| hsa-miR-614 | MI0003627 | GAACGCCUGUUCUUGCCAGGUGG | 0.6717 | 0.3606 |
| hsa-miR-615 | MI0003628 | UCCGAGCCUGGGUCUCCCUCUU | 0.8101 | 0.3442 |
| hsa-miR-615-5p | MI0003628 | GGGGGUCCCCGGUGCUCGGAUC | 1.266 | 0.0411 |
| hsa-miR-616 | MI0003629 | AGUCAUUGGAGGGUUUGAGCAG | 0.7116 | 0.1492 |
| hsa-miR-616* | MI0003629 | ACUCAAAACCCUUCAGUGACUU | 1.0413 | 0.3889 |
| hsa-miR-617 | MI0003631 | AGACUUCCCAUUUGAAGGUGGC | 0.34 | 0.0802 |
| hsa-miR-618 | MI0003632 | AAACUCUACUUGUCCUUCUGAGU | 0.4952 | 0.0883 |
| hsa-miR-619 | MI0003633 | GACCUGGACAUGUUUGUGCCCAGU | 0.4288 | 0.0531 |
| hsa-miR-620 | MI0003634 | AUGGAGAUAGAUAUAGAAAU | 0.5135 | 0.1821 |
| hsa-miR-621 | MI0003635 | GGCUAGCAACAGCGCUUACCU | 0.5389 | 0.0199 |
| hsa-miR-622 | MI0003636 | ACAGUCUGCUGAGGUUGGAGC | 1.4738 | 0.4459 |
| hsa-miR-623 | MI0003637 | AUCCCUUGCAGGGGCUGUUGGGU | 0.84 | 0.0307 |
| hsa-miR-624 | MI0003638 | CACAAGGUAUUGGUAUUACCU | 0.3782 | 0.031 |
| hsa-miR-624* | MI0003638 | UAGUACCAGUACCUUGUGUUCA | 0.5871 | 0.1818 |
| hsa-miR-625 | MI0003639 | AGGGGGAAAGUUCUAUAGUCC | 0.1213 | 0.0531 |
| hsa-miR-625* | MI0003639 | GACUAUAGAACUUUCCCCCUCA | 0.249 | 0.0411 |
| hsa-miR-626 | MI0003640 | AGCUGUCUGAAAAUGUCUU | 2.2236 | 0.6498 |
| hsa-miR-627 | MI0003641 | GUGAGUCUCUAAGAAAAGAGGA | 0.9769 | 0.1801 |
| hsa-miR-628 | MI0003642 | UCUAGUAAGAGUGGCAGUCGA | 0.5782 | 0.2092 |
| hsa-miR-628-5p | MI0003642 | AUGCUGACAUAUUUACUAGAGG | 0.2957 | 0.0523 |
| hsa-miR-629 | MI0003643 | UGGGUUUACGUUGGGAGAACU | 0.1617 | 0.0121 |
| hsa-miR-629* | MI0003643 | GUUCUCCCAACGUAAGCCCAGC | 0.2919 | 0.1369 |
| hsa-miR-630 | MI0003644 | AGUAUUCUGUACCAGGGAAGGU | 0.4505 | 0.1352 |
| hsa-miR-631 | MI0003645 | AGACCUGGCCCAGACCUCAGC | 0.1216 | 0.0358 |
| hsa-miR-632 | MI0003647 | GUGUCUGCUUCCUGUGGGA | 0.8065 | 0.0707 |
| hsa-miR-633 | MI0003648 | CUAAUAGUAUCUACCACAAUAAA | 0.6925 | 0.1402 |
| hsa-miR-634 | MI0003649 | AACCAGCACCCCAACUUUGGAC | 0.209 | 0.0134 |
| hsa-miR-635 | MI0003650 | ACUUGGGCACUGAAACAAUGUCC | 0.5985 | 0.1015 |
| hsa-miR-636 | MI0003651 | UGUGCUUGCUCGUCCCGCCCGCA | 1.0122 | 0.2429 |
| hsa-miR-637 | MI0003652 | ACUGGGGGCUUUCGGGCUCUGCGU | 0.2198 | 0.0695 |
| hsa-miR-638 | MI0003653 | AGGGAUCGCGGGCGGGUGGCGGCCU | 0.889 | 0.246 |
| hsa-miR-639 | MI0003654 | AUCGCUGCGGUUGCGAGCGCUGU | 0.9401 | 0.0816 |
| hsa-miR-640 | MI0003655 | AUGAUCCAGGAACCUGCCUCU | 0.261 | 0.0508 |
| hsa-miR-641 | MI0003656 | AAAGACAUAGGAUAGAGUCACCUC | 1.3941 | 0.1014 |
| hsa-miR-642 | MI0003657 | GUCCCUCUCCAAAUGUGUCUUG | 1.9661 | 0.3624 |
| hsa-miR-643 | MI0003658 | ACUUGUAUGCUAGCUCAGGUAG | 1.0455 | 0.0762 |
| hsa-miR-644 | MI0003659 | AGUGUGGCUUUCUUAGAGC | 0.1293 | 0.0227 |
| hsa-miR-645 | MI0003660 | UCUAGGCUGGUACUGCUGA | 0.614 | 0.0393 |
| hsa-miR-646 | MI0003661 | AAGCAGCUGCCUCUGAGGC | 0.1545 | 0.0116 |
| hsa-miR-647 | MI0003662 | GUGGCUGCACUCACUUCCUUC | 0.3025 | 0.0149 |
| hsa-miR-648 | MI0003663 | AAGUGUGCAGGGCACUGGU | 0.3125 | 0.0705 |
| hsa-miR-649 | MI0003664 | AAACCUGUGUUGUUCAAGAGUC | 1.0183 | 0.2277 |
| hsa-miR-650 | MI0003665 | AGGAGGCAGCGCUCUCAGGAC | 0.2292 | 0.0487 |
| hsa-miR-651 | MI0003666 | UUUAGGAUAAGCUUGACUUUUG | 0.8615 | 0.1583 |
| hsa-miR-652 | MI0003667 | AAUGGCGCCACUAGGGUUGUG | 0.7119 | 0.3692 |
| hsa-miR-653 | MI0003674 | GUGUUGAAACAAUCUCUACUG | 0.7321 | 0.2289 |
| hsa-miR-654 | MI0003676 | UGGUGGGCCGCAGAACAUGUGC | 0.1602 | 0.0044 |
| hsa-miR-654-3p | MI0003676 | UAUGUCUGCUGACCAUCACCUU | 0.9814 | 0.0961 |
| hsa-miR-655 | MI0003677 | AUAAUACAUGGUUAACCUCUUU | 0.0946 | 0.0211 |
| hsa-miR-656 | MI0003678 | AAUAUUAUACAGUCAACCUCU | 0.3236 | 0.0146 |
| hsa-miR-657 | MI0003681 | GGCAGGUUCUCACCCUCUCUAGG | 0.3062 | 0.0274 |
| hsa-miR-658 | MI0003682 | GGCGGAGGGAAGUAGGUCCGUUGGU | 1.0709 | 0.2048 |
| hsa-miR-659 | MI0003683 | CUUGGUUCAGGGAGGGUCCCCA | 0.5538 | 0.1578 |
| hsa-miR-660 | MI0003684 | UACCCAUUGCAUAUCGGAGUUG | 0.5045 | 0.0832 |
| hsa-miR-661 | MI0003669 | UGCCUGGGUCUCUGGCCUGCGCGU | 0.3174 | 0.0365 |
| hsa-miR-662 | MI0003670 | UCCCACGUUGUGGCCCAGCAG | 1.1288 | 0.243 |
| hsa-miR-663 | MI0003672 | AGGCGGGGCGCCGCGGGACCGC | 0.4037 | 0.0807 |
| hsa-miR-665 | MI0005563 | ACCAGGAGGCUGAGGCCCCU | 0.118 | 0.0099 |
| hsa-miR-668 | MI0003761 | UGUCACUCGGCUCGGCCCACUAC | 0.6337 | 0.1719 |
| hsa-miR-671 | MI0003760 | AGGAAGCCCUGGAGGGGCUGGAG | 0.3449 | 0.0158 |
| hsa-miR-671-3p | MI0003760 | UCCGGUUCUCAGGGCUCCACC | 0.073 | 0.0075 |
| hsa-miR-675 | MI0005416 | UGGUGCGGAGAGGGCCCACAGUG | 0.6369 | 0.1046 |
| hsa-miR-7 | MI0000264 | UGGAAGACUAGUGAUUUUGUUGU | 0.1304 | 0.0411 |
| hsa-miR-7 | MI0000265 | UGGAAGACUAGUGAUUUUGUUGU | 0.1634 | 0.0055 |
| hsa-miR-7 | MI0000263 | UGGAAGACUAGUGAUUUUGUUGU | 0.2436 | 0.1204 |
| hsa-miR-708 | MI0005543 | AAGGAGCUUACAAUCUAGCUGGG | 0.076 | 0.0025 |
| hsa-miR-708* | MI0005543 | CAACUAGACUGUGAGCUUCUAG | 0.3967 | 0.1011 |
| hsa-miR-7-1* | MI0000263 | CAACAAAUCACAGUCUGCCAUA | 1.1431 | 0.1062 |
| hsa-miR-7-2* | MI0000264 | CAACAAAUCCCAGUCUACCUAA | 0.9854 | 0.2479 |
| hsa-miR-744 | MI0005559 | UGCGGGGCUAGGGCUAACAGCA | 0.0479 | 0.0037 |
| hsa-miR-744* | MI0005559 | CUGUUGCCACUAACCUCAACCU | 0.1043 | 0.0172 |
| hsa-miR-758 | MI0003757 | UUUGUGACCUGGUCCACUAACC | 0.5459 | 0.0981 |
| hsa-miR-760 | MI0005567 | CGGCUCUGGGUCUGUGGGGA | 0.702 | 0.0718 |
| hsa-miR-765 | MI0005116 | UGGAGGAGAAGGAAGGUGAUG | 0.1095 | 0.0046 |
| hsa-miR-766 | MI0003836 | ACUCCAGCCCCACAGCCUCAGC | 0.1945 | 0.016 |
| hsa-miR-767-3p | MI0003763 | UCUGCUCAUACCCCAUGGUUUCU | 0.3494 | 0.0094 |
| hsa-miR-767-5p | MI0003763 | UGCACCAUGGUUGUCUGAGCAUG | 0.3221 | 0.0803 |
| hsa-miR-768-3p | MI0005117 | UCACAAUGCUGACACUCAAACUGCUGAC | 0.8368 | 0.3417 |
| hsa-miR-768-5p | MI0005117 | GUUGGAGGAUGAAAGUACGGAGUGAU | 0.2011 | 0.0548 |
| hsa-miR-769-3p | MI0003834 | CUGGGAUCUCCGGGGUCUUGGUU | 0.4339 | 0.223 |
| hsa-miR-769-5p | MI0003834 | UGAGACCUCUGGGUUCUGAGCU | 0.3543 | 0.0858 |
| hsa-miR-770-5p | MI0005118 | UCCAGUACCACGUGUCAGGGCCA | 0.2527 | 0.098 |
| hsa-miR-801 | MI0005202 | GAUUGCUCUGCGUGCGGAAUCGAC | 0.2724 | 0.0052 |
| hsa-miR-802 | MI0003906 | CAGUAACAAAGAUUCAUCCUUGU | 0.5253 | 0.0675 |
| hsa-miR-873 | MI0005564 | GCAGGAACUUGUGAGUCUCCU | 0.2555 | 0.0462 |
| hsa-miR-874 | MI0005532 | CUGCCCUGGCCCGAGGGACCGA | 2.0532 | 0.1008 |
| hsa-miR-875-3p | MI0005541 | CCUGGAAACACUGAGGUUGUG | 0.9303 | 0.0679 |
| hsa-miR-875-5p | MI0005541 | UAUACCUCAGUUUUAUCAGGUG | 0.4428 | 0.1395 |
| hsa-miR-876-3p | MI0005542 | UGGUGGUUUACAAAGUAAUUCA | 0.0701 | 0.0006 |
| hsa-miR-876-5p | MI0005542 | UGGAUUUCUUUGUGAAUCACCA | 0.4303 | 0.0311 |
| hsa-miR-877 | MI0005561 | GUAGAGGAGAUGGCGCAGGG | 1.4909 | 0.0534 |
| hsa-miR-877* | MI0005561 | UCCUCUUCUCCCUCCUCCCAG | 0.471 | 0.1331 |
| hsa-miR-885-3p | MI0005560 | AGGCAGCGGGGUGUAGUGGAUA | 0.0819 | 0.0081 |
| hsa-miR-885-5p | MI0005560 | UCCAUUACACUACCCUGCCUCU | 0.2931 | 0.0781 |
| hsa-miR-886-3p | MI0005527 | CGCGGGUGCUUACUGACCCUU | 0.1257 | 0.0051 |
| hsa-miR-886-5p | MI0005527 | CGGGUCGGAGUUAGCUCAAGCGG | 1.42 | 0.084 |
| hsa-miR-887 | MI0005562 | GUGAACGGGCGCCAUCCCGAGG | 0.379 | 0.0521 |
| hsa-miR-888 | MI0005537 | UACUCAAAAAGCUGUCAGUCA | 1.5155 | 0.116 |
| hsa-miR-888* | MI0005537 | GACUGACACCUCUUUGGGUGAA | 0.3822 | 0.0395 |
| hsa-miR-889 | MI0005540 | UUAAUAUCGGACAACCAUUGU | 0.17 | 0.0036 |
| hsa-miR-890 | MI0005533 | UACUUGGAAAGGCAUCAGUUG | 0.2546 | 0.0373 |
| hsa-miR-891a | MI0005524 | UGCAACGAACCUGAGCCACUGA | 0.5129 | 0.0106 |
| hsa-miR-891b | MI0005534 | UGCAACUUACCUGAGUCAUUGA | 0.0671 | 0.0053 |
| hsa-miR-892a | MI0005528 | CACUGUGUCCUUUCUGCGUAG | 0.315 | 0.026 |
| hsa-miR-892b | MI0005538 | CACUGGCUCCUUUCUGGGUAGA | 0.1954 | 0.0136 |
| hsa-miR-9 | MI0000467 | UCUUUGGUUAUCUAGCUGUAUGA | 0.3391 | 0.0521 |
| hsa-miR-9 | MI0000468 | UCUUUGGUUAUCUAGCUGUAUGA | 0.3637 | 0.2151 |
| hsa-miR-9 | MI0000466 | UCUUUGGUUAUCUAGCUGUAUGA | 0.4367 | 0.1118 |
| hsa-miR-9* | MI0000467 | AUAAAGCUAGAUAACCGAAAGU | 0.1091 | 0.048 |
| hsa-miR-9* | MI0000468 | AUAAAGCUAGAUAACCGAAAGU | 0.1247 | 0.0128 |
| hsa-miR-9* | MI0000466 | AUAAAGCUAGAUAACCGAAAGU | 0.1269 | 0.0229 |
| hsa-miR-92 | MI0000093 | UAUUGCACUUGUCCCGGCCUGU | 1.1106 | 0.5174 |
| hsa-miR-92 | MI0000094 | UAUUGCACUUGUCCCGGCCUGU | 1.3308 | 0.5716 |
| hsa-miR-920 | MI0005712 | GGGGAGCUGUGGAAGCAGUA | 0.4038 | 0.0817 |
| hsa-miR-921 | MI0005713 | CUAGUGAGGGACAGAACCAGGAUUC | 0.2349 | 0.0246 |
| hsa-miR-922 | MI0005714 | GCAGCAGAGAAUAGGACUACGUC | 0.3529 | 0.1104 |
| hsa-miR-923 | MI0005715 | GUCAGCGGAGGAAAAGAAACU | 0.8073 | 0.0563 |
| hsa-miR-924 | MI0005716 | AGAGUCUUGUGAUGUCUUGC | 0.241 | 0.0246 |
| hsa-miR-92a-1* | MI0000093 | AGGUUGGGAUCGGUUGCAAUGCU | 0.1227 | 0.0535 |
| hsa-miR-92a-2* | MI0000094 | GGGUGGGGAUUUGUUGCAUUAC | 0.3491 | 0.0401 |
| hsa-miR-92b | MI0003560 | UAUUGCACUCGUCCCGGCCUCC | 0.8792 | 0.4298 |
| hsa-miR-92b* | MI0003560 | AGGGACGGGACGCGGUGCAGUG | 0.3282 | 0.0611 |
| hsa-miR-93 | MI0000095 | CAAAGUGCUGUUCGUGCAGGUAG | 0.7293 | 0.0976 |
| hsa-miR-93* | MI0000095 | ACUGCUGAGCUAGCACUUCCCG | 0.1727 | 0.0248 |
| hsa-miR-933 | MI0005755 | UGUGCGCAGGGAGACCUCUCCC | 0.5851 | 0.0604 |
| hsa-miR-934 | MI0005756 | UGUCUACUACUGGAGACACUGG | 0.1192 | 0.026 |
| hsa-miR-935 | MI0005757 | CCAGUUACCGCUUCCGCUACCGC | 0.405 | 0.0576 |
| hsa-miR-936 | MI0005758 | ACAGUAGAGGGAGGAAUCGCAG | 0.2488 | 0.0172 |
| hsa-miR-937 | MI0005759 | AUCCGCGCUCUGACUCUCUGCC | 0.5233 | 0.0283 |
| hsa-miR-938 | MI0005760 | UGCCCUUAAAGGUGAACCCAGU | 0.6375 | 0.0874 |
| hsa-miR-939 | MI0005761 | UGGGGAGCUGAGGCUCUGGGGGUG | 0.6692 | 0.0533 |
| hsa-miR-940 | MI0005762 | AAGGCAGGGCCCCCGCUCCCC | 0.1075 | 0.0042 |
| hsa-miR-941 | MI0005763 | CACCCGGCUGUGUGCACAUGUGC | 0.5703 | 0.0587 |
| hsa-miR-941 | MI0005764 | CACCCGGCUGUGUGCACAUGUGC | 0.6789 | 0.2122 |
| hsa-miR-941 | MI0005765 | CACCCGGCUGUGUGCACAUGUGC | 0.6902 | 0.1106 |
| hsa-miR-941 | MI0005766 | CACCCGGCUGUGUGCACAUGUGC | 0.7211 | 0.1524 |
| hsa-miR-942 | MI0005767 | UCUUCUCUGUUUUGGCCAUGUG | 0.4111 | 0.0396 |
| hsa-miR-943 | MI0005768 | CUGACUGUUGCCGUCCUCCAG | 0.1329 | 0.0256 |
| hsa-miR-944 | MI0005769 | AAAUUAUUGUACAUCGGAUGAG | 0.0983 | 0.0046 |
| hsa-miR-95 | MI0000097 | UUCAACGGGUAUUUAUUGAGCA | 1.0638 | 0.1228 |
| hsa-miR-96 | MI0000098 | UUUGGCACUAGCACAUUUUUGCU | 0.5602 | 0.0531 |
| hsa-miR-96* | MI0000098 | AAUCAUGUGCAGUGCCAAUAUG | 0.3204 | 0.0075 |
| hsa-miR-98 | MI0000100 | UGAGGUAGUAAGUUGUAUUGUU | 0.5297 | 0.1638 |
| hsa-miR-99a | MI0000101 | AACCCGUAGAUCCGAUCUUGUG | 0.2992 | 0.0057 |
| hsa-miR-99a* | MI0000101 | CAAGCUCGCUUCUAUGGGUCUG | 0.9375 | 0.2274 |
| hsa-miR-99b | MI0000746 | CACCCGUAGAACCGACCUUGCG | 0.3068 | 0.0973 |
| hsa-miR-99b* | MI0000746 | CAAGCUCGUGUCUGUGGGUCCG | 0.6699 | 0.1676 |

Supplementary Table S4. The sequence of primers used in this study.

| **Primer** | **Sequence** |
| --- | --- |
| AR#1 US | CATT**ACTAGT**CCUGCUAAUCAAGUCACACA |
| AR#1 DS | CATT**AAGCTT**AGGUACUGAUGCUCCAUUGC |
| AR#2 US | CATT**ACTAGT**GGGGCTAGACTGCTCAACTG |
| AR#2 DS | CATT**AAGCTT**TTCTGCAGGTGGATATGCTG |
| AR#3 US | CATT**ACTAGT**TCCCAGCAAGTGGAGAAGTT |
| AR#3 DS | CATT**AAGCTT**TCACAATTGGGAAACCCATAA |
| AR#4 US | CATT**ACTAGT**GGTCCTTTACATCTGTTTTGGAA |
| AR#4 DS | CATT**AAGCTT**GGGACTCTAGCATCAGCACA |
| AR#5 US | CATT**ACTAGT**CCTTTGGGTAGTTGCTGAGG |
| AR#5 DS | CATT**AAGCTT**CATTTCCAACCATGCAATGA |
| AR#6 US | CATT**ACTAGT**CCTAGGACCTCCCAACTCAA |
| AR#6 DS | CATT**AAGCTT**CCCAGGCACTTCACTGTAGG |
| AR#7 US | CATT**ACTAGT**GCCCTTGTGACCTGAAACAC |
| AR#7 DS | CATT**AAGCTT**GAGCTAATTAAAAGAACTCTGATGACA |
| AR-1-30d-M-F | GTCAAGTTGTGCTTGTTTGAAGCACTACTCTGTGCCAG |
| AR-1-30d-M-R | CTGGCACAGAGTAGTGCTTCAAACAAGCACAACTTGAC |
| AR-1 Mut-F (30b*-M) | CCCTCCCTATCTAACCCTCAAATGGCACCTTCAGACTTTG |
| AR-1 Mut-R (30b*-M) | CAAAGTCTGAAGGTGCCATTTGAGGGTTAGATAGGGAGGG |
| AR-2 Mut-F (30b*-M) | CCCCAGAGATGATACCCTCAAAGCAAGTGGAGAAGTTCTC |
| AR-2 Mut-R (30b*-M) | GAGAACTTCTCCACTTGCTTTGAGGGTATCATCTCTGGGG |
| PSA-F | ATCCTGTCTCGGATTGTGGG |
| PSA-R | GAGTCATCACCTGGCTTCCT |
| Actin-F | AGGCACCAGGGCGTGAT |
| Actin-R | GCCCACATAGGAATCCTTCTGAC |

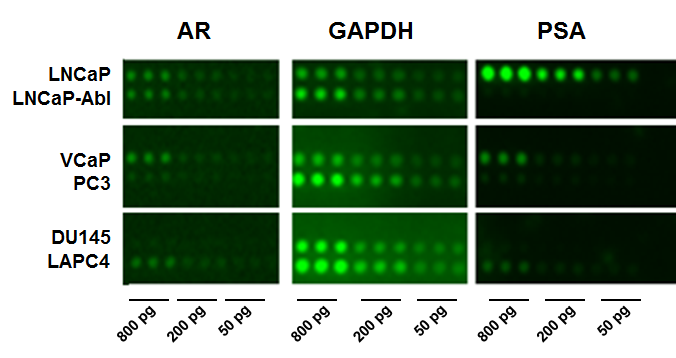


**Supplementary Figure S1. Protein LMA Specificity and Controls.** Positive and negative control cell lysate signals for AR, GAPDH and PSA on protein LMA. Lysates are spotted in triplicate for each concentration (800, 200, and 50 pg). Cell lines are indicated. Applied dilutions for each antibody are as follows: Anti Androgen receptor antibody; 1:2000, anti PSA antibody; 1:5000, anti GAPDH; 1:5000.


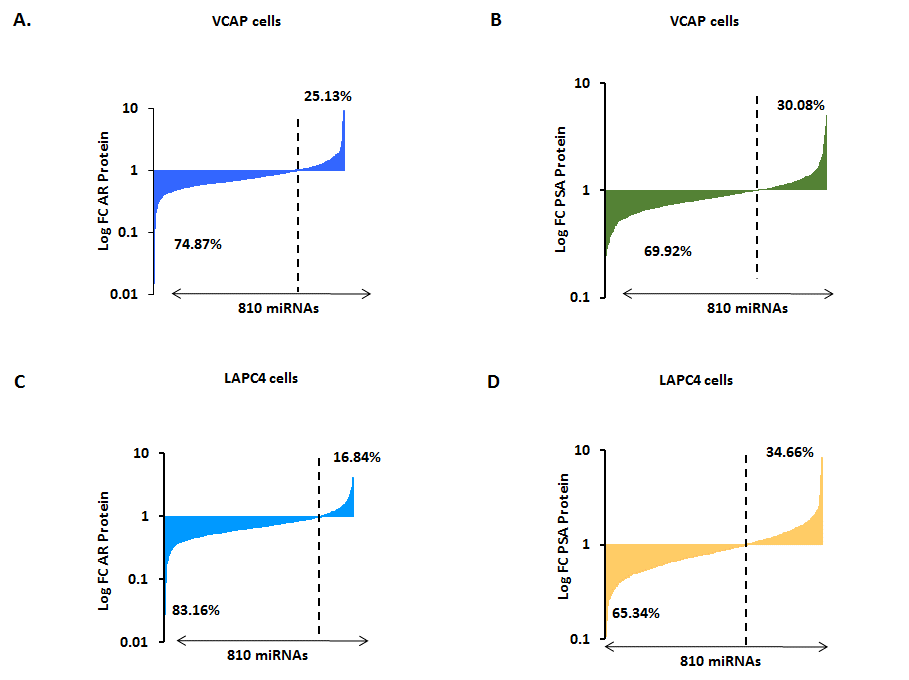


**Supplementary Figure S2**. **High-throughput miRNA mimic library screen for miRNAs that modulate AR and PSA protein levels**. A, Log transformed Fold Change (FC) in AR protein level, relative to control miRNA (dashed line), in LAPC4 cells. 48 hr post transfection. Protein level, normalized to GAPDH, is organized as a bar graph waterfall plot for each mimic, ranked by FC in AR expression. B, Log transformed waterfall plot for FC in PSA protein level, relative to control miRNA (dashed line), for each mimic in LAPC4 cells. 48 hr post transfection. C, Log transformed waterfall plot for FC in AR protein level, relative to control miRNA (dashed line), for each mimic in VCaP cells. 48 hr post transfection. D, Log transformed waterfall plot for FC in PSA protein level, relative to control miRNA (dashed line), for each mimic in VCaP cells. 48 hr post transfection. Each waterfall plot indicates percentage of library with FC signal below and above miRNA controls. Mimics with negative values in LMAs have been removed from analyses.


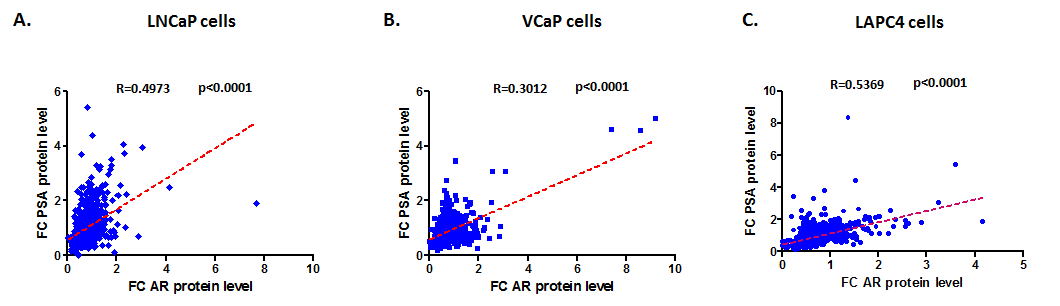


**Supplementary Figure S3.** **Correlation of PSA and AR protein levels for each miRNA mimic in protein LMA analyses.** A, LNCaP; B, VCaP; and C, LAPC4 prostate cancer cell lines. Signals represent FC in protein signal, relative to control miRNA, for each miRNA. Correlation determined by Pearson correlation calculation. R – Pearson r, p value indicated.


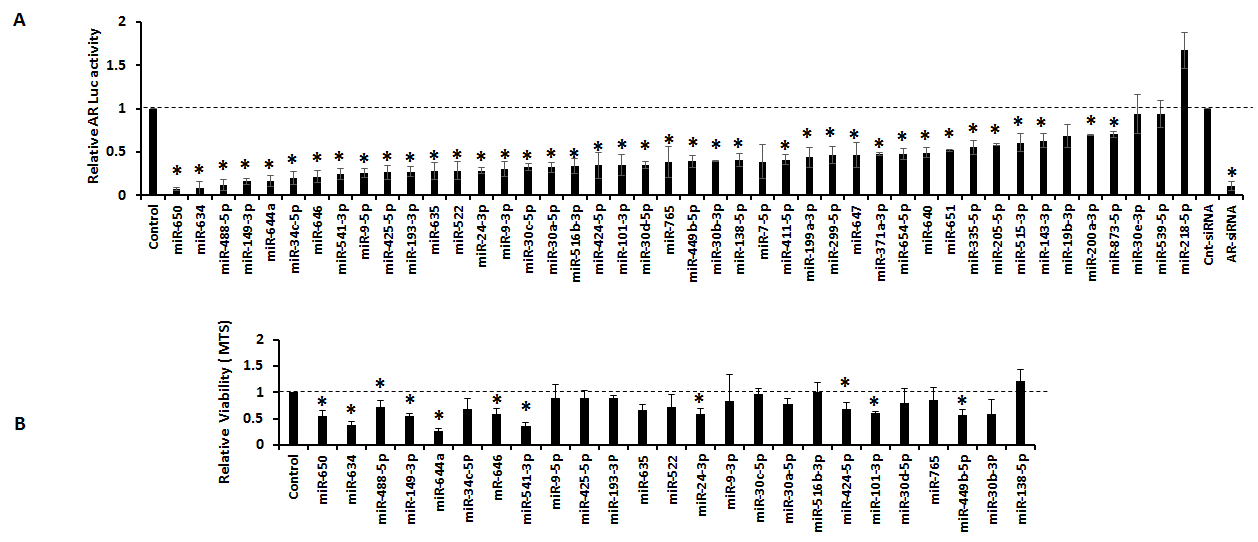


**Supplementary Figure S4**. **Additional verification analyses for candidate AR-regulating miRNAs.** A, MiRNA induced fold changes in AR transcriptional activity as measured by *Renilla* normalized Firefly luciferase activity from the AR-responsive pBK-PSE-PBN-Luc vector. Activity determined 48 hr post transfection and 24 hr after stimulation with 5 nM R1881. Results are representative of minimum two independent experiments for each miRNAs in triplicates. B, MiRNA induced fold changes in LNCaP-95 cell viability, 6 days post miRNA transfection, as determined by Cell Titer aqueous one solution Cell Proliferation Assay. Results are representative of two independent experiments for each miRNA. Each graph represents average FC in signal and error bars represent standard deviation. Dashed line represent control miRNA treated cell activity or viability. Cnt-siRNA, Control siRNA, AR-siRNA.


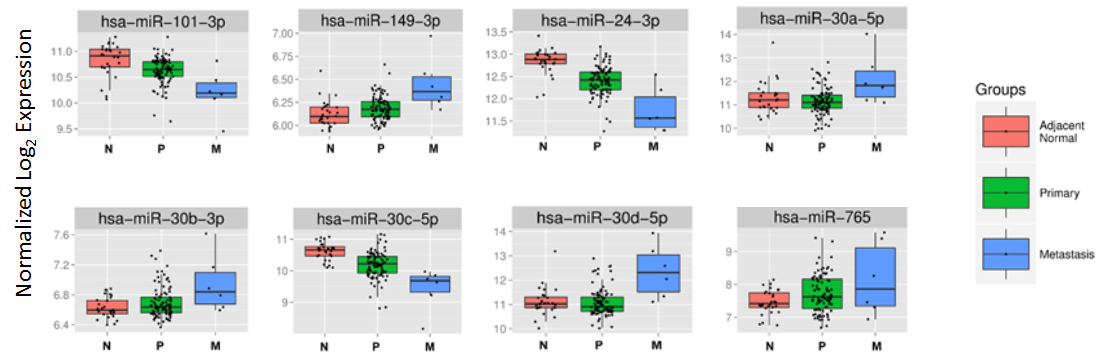


**Supplementary Figure S5. Differentially expressed AR regulatory miRNAs in Taylor dataset**. Log2 expression level of miRNAs showing significant (FDR < 0.05) and concordant differential expression with progression from normal to localized prostate cancer (PCa) and from localized PCa to metastasis (GSE21036). Boxplots represent the 25-75 quantiles. Horizontal line is median, vertical line indicates the 5-95 quantiles. All points are shown for complete data representation. N = Normal, P = Primary Prostate Cancer, M = Metastatic Prostate Cancer.


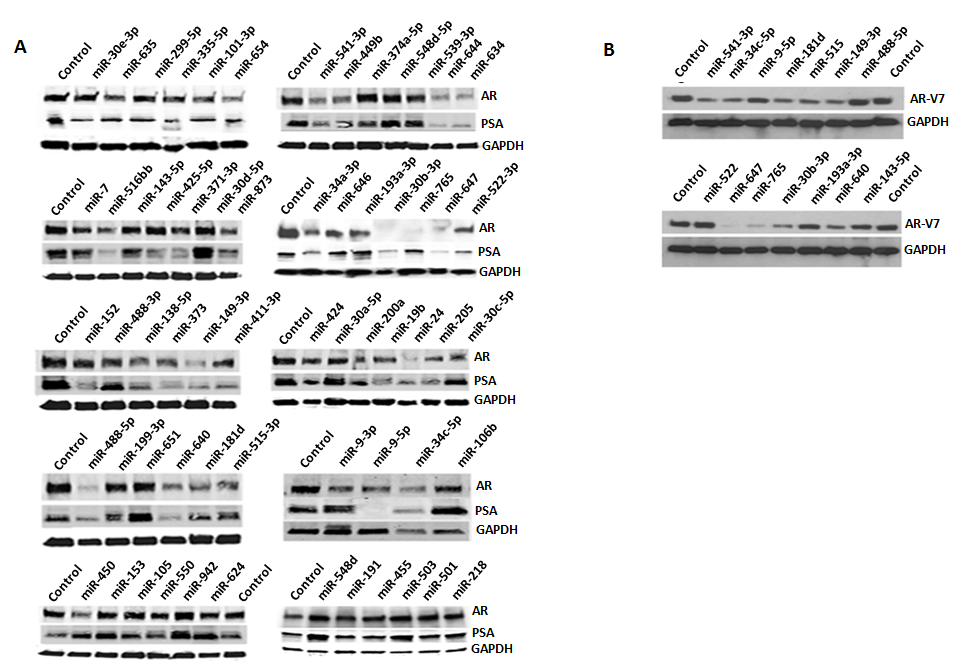


**Supplementary Figure S6. MiRNA effects on AR, AR-V7 and PSA protein levels.** A, Western blot for AR and PSA from LNCaP cells 48 hr post transfection with miRNA mimics (20 nM) obtained for verification assays. B, Western blot for AR-V7 from miRNA-transfected VCAP cells.


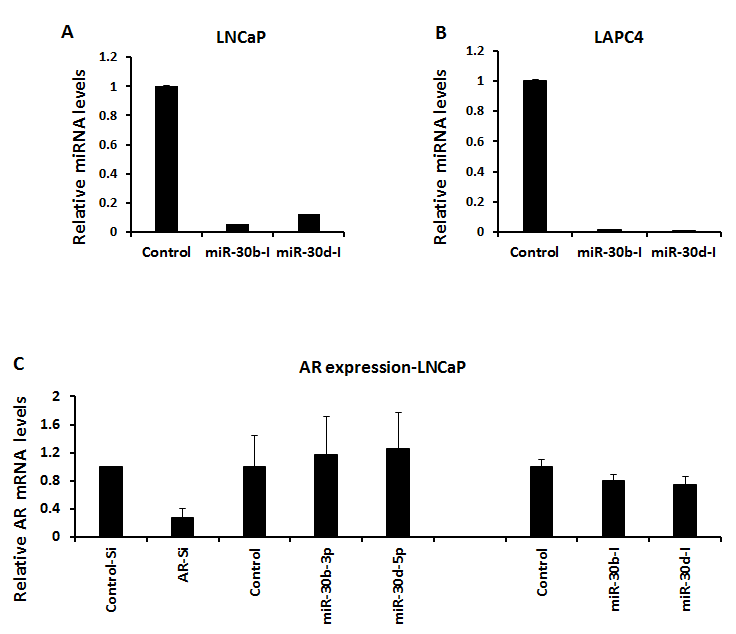


**Supplementary Figure S7. Evaluation of miR-30 mimic and antagomir transfections.** A&B, LNCaP and LAPC4 cells were transfected with 20 nM of control, miR-30b-3p or miR-30d-5p antagomirs (miR-30b-I and miR-30d-I). 48 hr after transfection, RNA was isolated and cDNA generated for respective miRNAs. Relative miRNA levels were quantified by droplet digital PCR, and normalized to RNU6B. Data presents mean and standard deviation of at least two independent ddPCR experiments. C, Effect of mimic and antagomir transfection on AR mRNA levels. MiR-30 mimics, inhibitors, or AR siRNA (20 nM) were transfected into LNCaP cells for 48h followed by RNA isolation. Following reverse transcription, AR mRNA levels were quantified by ddPCR. β-actin mRNA was used for normalization. Data presents mean and standard deviation of at least two independent ddPCR experiments from each cell lines.

**Supplementary Figure S8. Evaluation of miR-30d-5p activity with AR1 3’UTR in LNCaP Cells.** LNCaP cells were co-transfected with miR-30d-5p mimics, the AR-1 3’UTR reporter with intact (AR1-WT) and mutated (AR1-M) miR-30d-5p seed sequence binding sites, and a constitutively active *Renilla* luciferase vector for normalization. After 48 hours, reporter activity was assessed by dual luciferase enzyme assay and normalized to Renilla luciferase.


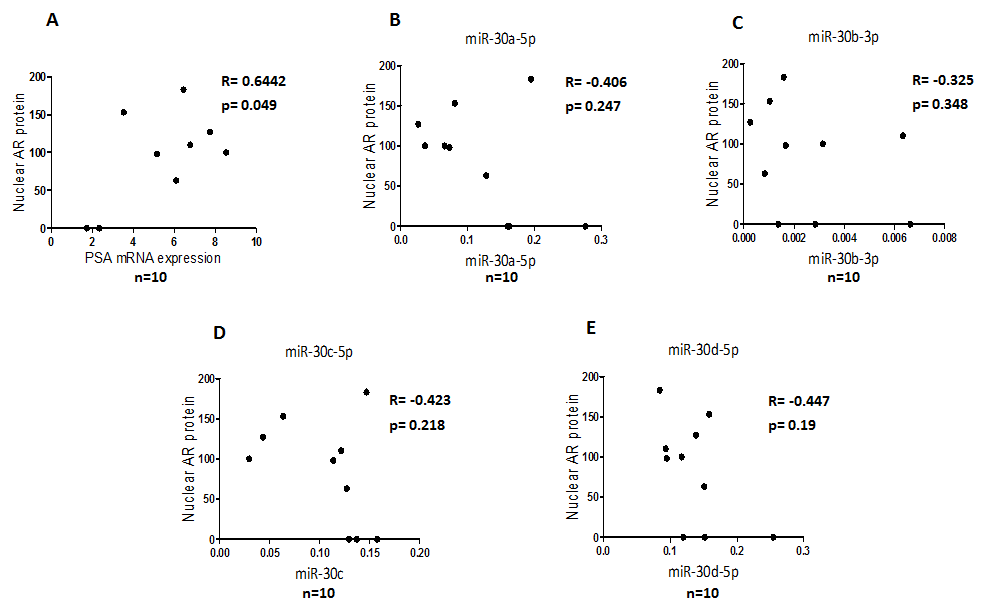


**Supplementary Figure S9. Correlation of nuclear AR protein staining with PSA mRNA and miR-30 miRNA levels.** A, Correlation between PSA mRNA levels, as quantified by qRT-PCR and normalized to GAPDH, and nuclear AR protein staining, as determined by immunohistochemistry, in CRPC samples (N = 10). A direct correlation was determined by Spearman Correlation calculation (p = 0.049). B-E, Inverse trend between nuclear AR protein expression and miR-30a-5p, miR-30b-3p, miR-30c-5p, and miR-30d-5p (N = 10). Data for nuclear AR expression were obtained through the PCBN, through Correlation determined by Pearson correlation calculation. R – Pearson r.
